# Screening and Functional Analysis of Hub MicroRNAs Related to Tumor Development in Colon Cancer

**DOI:** 10.1155/2020/3981931

**Published:** 2020-01-23

**Authors:** Dong-Hu Yu, Wei Li, Jing-Yu Huang, Xiao-Ping Liu, Chi Zhang, Xiao-Lan Ruan, Sheng Li

**Affiliations:** ^1^Department of Biological Repositories, Human Genetics Resource Preservation Center of Hubei Province, Zhongnan Hospital of Wuhan University, Wuhan 430071, China; ^2^The Second Clinical College, Wuhan University, Wuhan 430071, China; ^3^Department of Oncology, The First People's Hospital of Tianmen, Tianmen 431700, Hubei Province, China; ^4^Department of Thoracic Surgery, Zhongnan Hospital of Wuhan University, Wuhan 430071, China; ^5^Hubei Key Laboratory of Tumor Biological Behaviors & Hubei Cancer Clinical Study Center, Wuhan 430071, China; ^6^Department of Gastrointestinal Surgery, Hubei Cancer Hospital, Tongji Medical College, Huazhong University of Science and Technology, Wuhan 430071, China; ^7^Department of Hematology, Renmin Hospital of Wuhan University, Wuhan 430071, China

## Abstract

Various microRNAs (miRNAs) are of importance in the development of colon cancer, but most of the mechanisms of the miRNAs are still unclear. In order to clarify the hub miRNAs and their roles in colon cancer development, GSE98406 was used to screen hub miRNAs by bioinformatics analysis. 46 DE-miRNAs (14 were upregulated and 32 were downregulated) and 1738 target genes of DE-miRNAs were ascertained. miRNAs-gene-networks and miRNAs-GO-networks were built to get more knowledge about the function of candidate miRNAs. After validation, three miRNAs (miR-17-5p, miR-182-5p and miR-200a-3p) were recognized to be hub miRNAs associated with the progression of colon cancer. More importantly, the hub miRNAs and the putative targets genes might be new diagnostic and therapeutic targets for colon cancer in the future.

## 1. Introduction

Colon cancer is a common malignancy that affects more than 130,000 people each year, causing about 60,000 deaths [1, 2]. Although the overall five-year survival of patients with colon cancer is generally high with proper treatment, the complex unknown pathogenesis limits the further improvement in colon cancer treatment [3, 4]. Therefore, there is an urgent need for more insights into the pathogenesis of colon cancer. In recent years, miRNAs' role in cancer research has received increasing attention. miRNA is an important factor in tumorigenesis and metastasis, and its expression characteristics are closely related to the occurrence, progression, and prognosis of various tumors [5, 6]. Previous studies have identified some important miRNAs impairing the development of cancers by miRNA expression profiles [7–9]. Besides, bioinformatics analysis was widely used for the identification of novel biomarkers and mechanism studies [10, 11]. In this study, we aimed to search and confirm hub miRNAs that play important parts in the development of colon cancer, thus providing more information for the mechanism research and clinical application of colon cancer.

## 2. Materials and Methods

### 2.1. Data Collection and Processing

**Figure 1:**
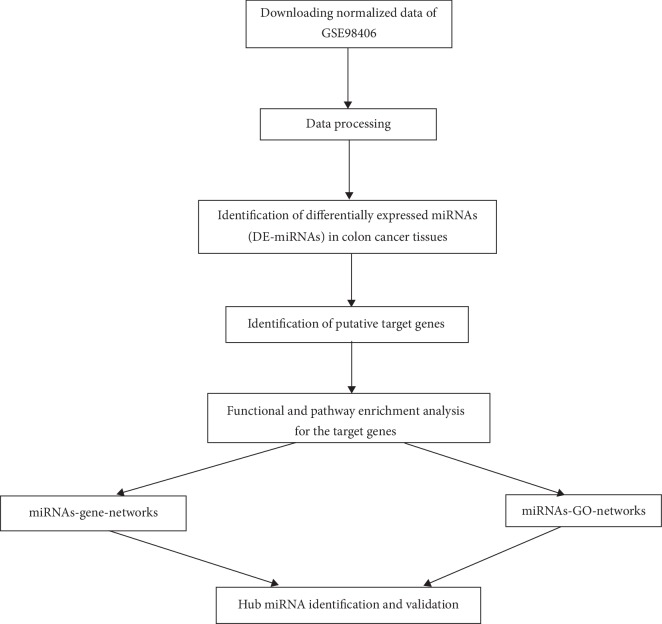
Flow chart of data preparation, processing, analysis, and validation.

The brief workflow of this study is shown in Figure 1. The microRNA expression profiles of GSE98406, GSE83924, GSE48267, and GSE35834 were downloaded from the Gene Expression Omnibus (GEO) database (https://www.ncbi.nlm.nih.gov/geo/). Quantile normalization was performed to normalize all datasets. [Supplementary-material supplementary-material-1] lists the details of these datasets. GSE98406 was used as a training dataset for screening DE-microRNAs. GSE83924 and GSE48267 were used as independent sample *T* test sets for verification, respectively. In addition, the clinicopathological correlation analysis for the colon cancer samples in GSE35834 was performed.

### 2.2. Screening of DE-miRNAs in Colon Cancer Tissues

In this study, the “limma” package in R [12] was used to screen DE-miRNAs between normal colon tissues and tumor tissues. The cutoff criteria were FDR < 0.05 and |Log_2_FC| > 1.5.

### 2.3. Functional Enrichment Analysis of Putative Target Genes

In order to get more knowledge about the candidate miRNAs function, we submitted the selected miRNAs to GCBI to screen their target genes. GCBI is an online tool, which can be used to predict miRNA target genes based on miRanda and TargetScan. Gene Ontology (GO) enrichment analysis and Kyoto Encyclopedia of Genes and Genomes (KEGG) pathway enrichment analysis for target genes were performed. The cut-off criterion is FDR < 0.05. Also, we drew an interactive network by pathway network (path-net) analysis in GCBI, which covered the significant KEGG pathway to find the hub pathways. The degrees of each pathway in this path-net were calculated, and the top 5 pathways with highest degrees were selected as hub pathways.

### 2.4. Identification and Validation of HUB miRNAs

After understanding the target genes and the GOs for DE-miRNAs, two important networks for this study (miRNA-gene-network and miRNA-GO-network) were built. Based on the ordering of the number of microRNAs in the two networks, we selected the overlapping hub miRNAs and the key regulatory functions of these miRNA. Two datasets (GSE83924 and GSE48267) were used to verify differential expression levels of these miRNAs between normal colon tissues and tumor tissues by independent sample *T* test, respectively. *P* < 0.05 was considered statistically significant. Meanwhile, the datasets of GSE83924 and GSE48267 were used to perform ROC curve analysis, and the AUC for each hub miRNA was calculated to distinguish the tumor tissues from the normal tissues.

### 2.5. The Clinical Significance of HUB miRNAs in Colon Cancer

Based on 52 cases of colon cancer samples with complete clinical information in GSE35834, the relationship between the expression of hub miRNAs and clinicopathological parameters was evaluated by clinicopathological correlation analysis. According to the amount of hub miRNA expression, 52 cases were classified into high expression group and low expression group according to the hub miRNA expression (high group, *n* = 26; low group, *n* = 26). Chi-square tests were used to assess the relationship between the expression of hub miRNA and gender, age, tumor grade, TNM stage, and metastasis of colon cancer patients. A value of *P* <0.05 was considered as statistically significant.

## 3. Results

### 3.1. DE-miRNAs and Target Genes in Colon Tumor Tissues

**Figure 2:**
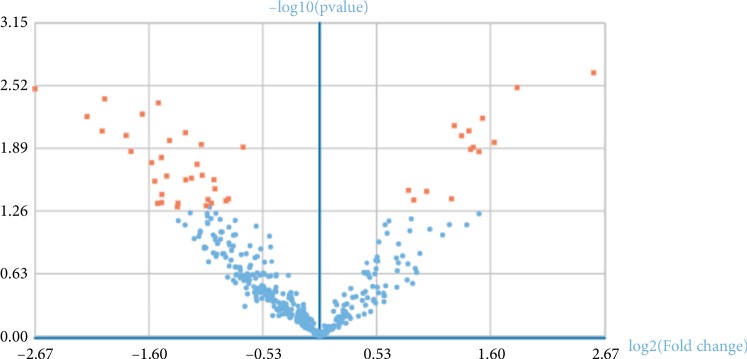
The volcano plot for DE-miRNAs.

Under the thresholds of FDR < 0.05 and |Log_2_FC| > 1.5, a total of 46 DE-miRNAs (14 up-regulated and 32 down-regulated in colon cancer samples) were selected from 7 control samples and 14 colon samples in GSE98406. The volcano plot for DE-miRNAs was taken (Figure 2) and the characteristics of the dysregulated miRNAs are listed in Table 1. Based on miRanda and TargetScan, 1738 putative miRNA target genes were identified using GCBI ([Supplementary-material supplementary-material-1]).

### 3.2. Functional Enrichment Analysis of Target Genes

**Figure 3:**
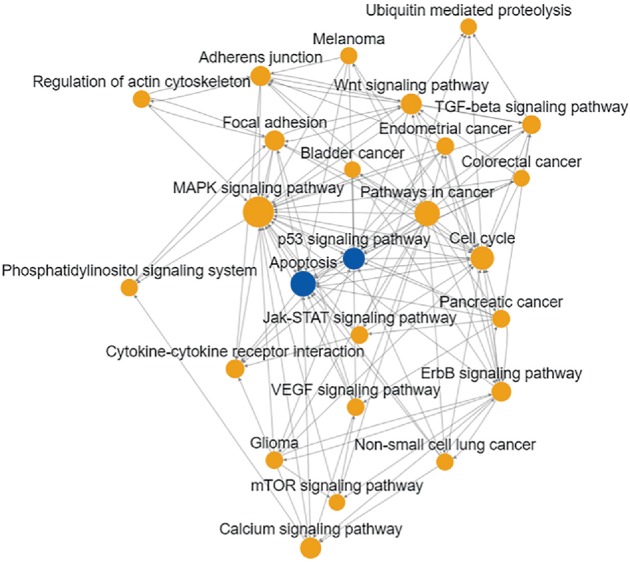
Pathway network (Path-net). Significantly changed pathways were connected in a Path-net to show the interaction network among these pathways. Each pathway in the network was measured by counting the upstream and downstream pathways. The blue circle represents pathways involving upregulated miRNAs, while the yellow circle represents pathways involving both upregulated and downregulated miRNAs. The size of the circle represents the degree value and the lines show the interaction between pathways. A higher degree of pathway indicates that it plays a more important role in the signaling network.

To study the roles of DE-miRNAs in mediating colon cancer progression, we performed GO analysis and KEGG pathway enrichment analysis for target genes. The data in Table 2 indicate that top 10 GOs were “transcription, DNA-dependent”, ‘‘regulation of transcription, DNA-dependent”, ‘‘signal transduction”, ‘‘positive regulation of transcription from RNA polymerase II promoter”, ‘‘apoptotic process”, ‘‘positive regulation of transcription, DNA-dependent”, ‘‘negative regulation of transcription from RNA polymerase II promoter”, ‘‘nervous system development”, ‘‘axon guidance” and ‘‘protein phosphorylation”. According to the KEGG database, the main pathways involving the target genes were demonstrated. As shown in Table 3, the top 10 pathways were “MAPK signaling pathway”, “pathways in cancer”, “PI3K-Akt signaling pathway, proteoglycans in cancer”, “HTLV-I infection”, “endocytosis”, “transcriptional mis-regulation in cancer”, “neurotrophin signaling pathway”, “axon guidance and GnRH signaling pathway”. What is more, a pathway network was shown, which covers 25 significantly changed pathways (Figure 3). Moreover, the MAPK signaling pathway (degrees:41), apoptosis (degrees:27), pathways in cancer (degree:27), cell cycle (degrees:23) and p53 signaling pathway (degrees:23) showed highest connectivity degrees in path-net, which demonstrated these 5 pathways would play a central role in tumor development.

### 3.3. Hub miRNAs Identification and Validation

**Figure 4:**
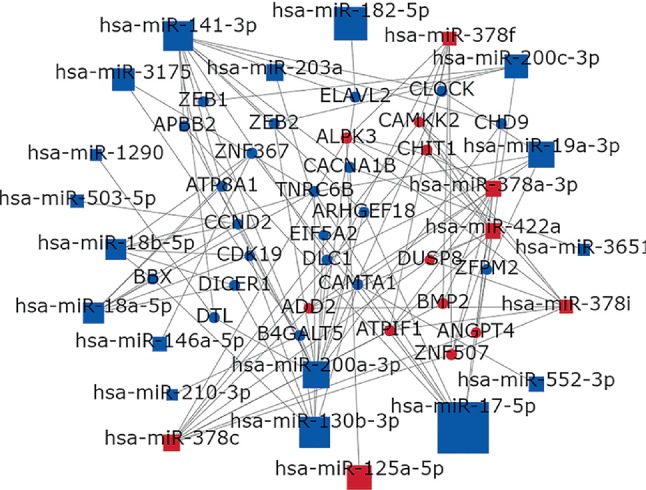
miRNAs-gene-network. According to the interactions between miRNAs and the intersected target genes, miRNAs-gene-network was constructed. The blue circles represent genes, while blue square nodes represent downregulated miRNAs. The size of the circle or square node represents the degree value. A higher degree of gene/miRNAs indicates that it plays a more important role in the signaling network.

**Figure 5:**
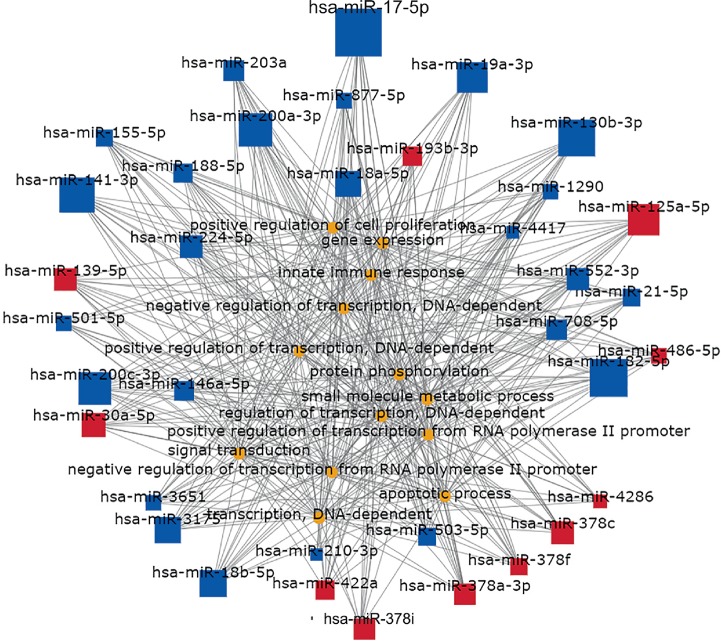
miRNAs-GO-network. The miRNAs-GO-network was generated according to the relationship of significant biological functions and miRNAs. The yellow and blue circles represent GOs, red square nodes represent upregulated miRNAs, and blue square nodes represent downregulated miRNAs. The size of the circle or square node represents the degree value. A higher degree of GO/miRNAs indicates that it plays a more important role in the signaling network.

**Figure 6:**
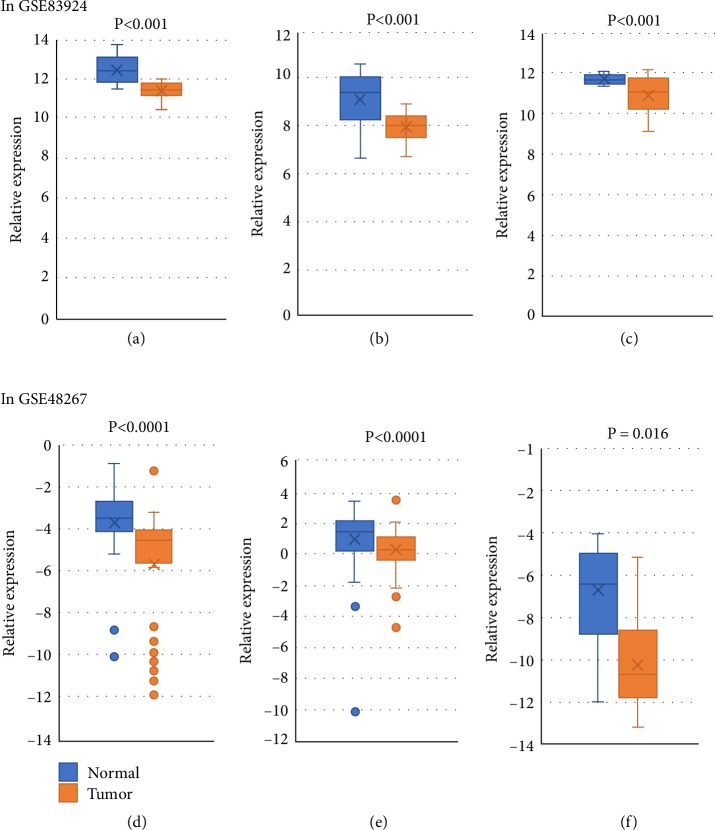
In GSE83924, miRNAs expression levels of miR-17-5p (a), miR-182-5p (b), and miR-200a-3p (c) between normal colon and tumor samples. In GSE48267, miRNAs expression levels of miR-17-5p (d), miR-182-5p (e), and miR-200a-3p (f) between normal colon and tumor samples. Independent sample *T* test was used to evaluate the statistical significance of differences.

**Figure 7:**
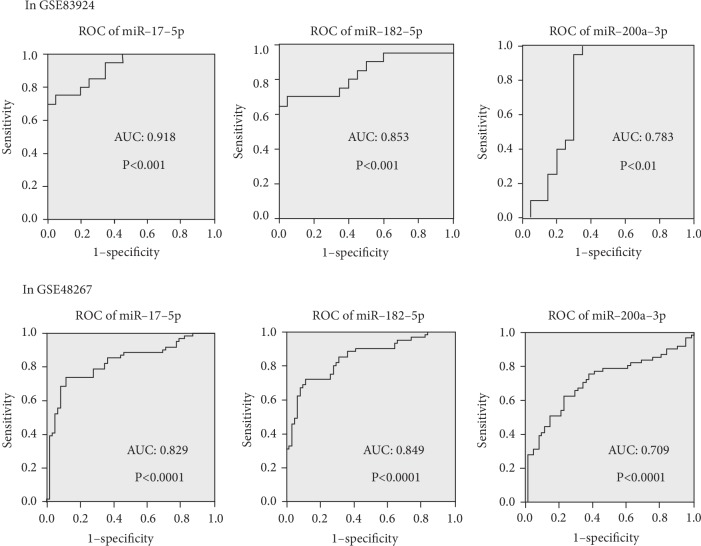
ROC curve of miR-17-5p, miR-182-5p and miR-200a-3p in the datasets of GSE83924 and GSE48267.

To identify hub miRNAs and their main functions in the development of colon cancer, we selected target genes and DE-miRNAs to construct miRNAs-gene-networks (Figure 4) and miRNAs-GO-networks (Figure 5) according to the significant regulation of GOs and pathways. According to the rank of degrees of miRNAs in two networks, the top rated three miRNAs (miR-17-5p, miR-182-5p, and miR-200a-3p) were determined to be hub miRNAs (Table 4). The bioinformatic analysis showed the hub miRNAs were lowly expressed in colon cancer tissues compared with normal colon tissues. And dysregulated miRNAs play important roles in signal transduction, apoptotic process, and pathways in cancer. GSE83924 and GSE48267 were used to make validation. Further proved by the datasets of GSE83924 and GSE48267, miR-17-5p, miR-182-5p and miR-200a-3p were lowly expressed in tumor tissues (Figure 6). Besides, ROC curve for GSE83924 indicated that miR-17-5p (AUC = 0.918), miR-182-5p (AUC = 0.853) and miR-200a-3p (AUC = 0.783) exhibited excellent diagnostic efficiency for tumor and normal tissues. And ROC curve for GSE48267 also demonstrated that miR-17-5p (AUC = 0.829), miR-182-5p (AUC = 0.849) and miR-200a-3p (AUC = 0.709) exhibited diagnostic efficiency for tumor and normal tissues (Figure 7).

### 3.4. Association of Hub miRNAs Expression with Clinical Significance

Chi-square analysis for GSE35834 showed that miR-17-5p expression was associated with tumor grade significantly (*P* = 0.02), and miR-182-5p expression group was associated with advanced TNM stage (III/IV) (*P* = 0.019). No other significant difference was observed in other clinicopathological features (age, gender, and metastasis) ([Supplementary-material supplementary-material-1]).

## 4. Discussion

In this study, bioinformatics analysis of GSE98406 revealed 46 DE-miRNAs (down-regulated 32 and up-regulated 14). According to the miRNAs-gene-networks and miRNAs-GO-networks, miR-17-5p, miR-182-5p, and miR-200a-3p were considered to be hub miRNAs. They play an important role in tumor development as tumor suppressor genes and oncogenes. Although miR-182-5p and miR-200a-3p have been found to be associated with colorectal cancer [13–15], there is still a lack of relevant studies exploring its regulatory mechanisms in colon cancer. As for miR-17-5p, it is the first time to discover it was negatively related to tumor progression.

Then, using the target prediction method in the GCBI online tool, 1738 genes were selected as target genes for these DE-miRNAs. The target genes predicted by GO analysis were enriched in “transcription, DNA-dependent”, “transcriptional regulation, DNA-dependent”, “signal transduction” and “positive regulation of transcription from RNA polymerase II promoter”. Interestingly, we noticed the opposite GOs (negative regulation of transcription from RNA polymerase II promoter and Positive regulation of transcription from RNA polymerase II promoter). Taken together, the hub miRNAs (hsa-miR-17-5p, hsa-miR-182-5p and hsa-miR-200a-3p) that we identified were reliable, which may be candidate biomarkers for colon cancer.

As for the 3 hub miRNAs, we conducted a literature review of these miRNAs. miR-17-5p is an important regulator, which has a strong effect on the G1/S phase of cell cycle transition [16]. MiR-17 has been found to target certain genes in some cancers, such as bladder cancer and oral squamous cell carcinoma [17, 18]. miR-182-5p is a member of the miR-183/96/182 cluster. Previous studies have identified its important role in breast cancer, glioma, prostate cancer, prostate cancer and renal cell carcinoma [19–23]. Generally, miR-182-5p regulates the apoptosis of tumor cells by targeting certain special genes, such as FOXO1, MTSS1, HMGA2, CASP9, and FOXO3 [24, 25], and these target genes were also predicted by our experiment. miR-200a-3p has been found to play important roles in the epithelial to the mesenchymal transition process in the development of cancer [26, 27]. miR-200a-3p plays a role like a tumor suppressor and its target genes are enriched in signal transmission and cell apoptosis control. miR-200a-3p is rarely used as a research focus and related regulatory mechanisms remain to be clarified.

In summary, we identified three hub miRNAs (hsa-miR-17-5p, hsa-miR-182-5p, and hsa-miR-200a-3p), which were closely related to the development of colon cancer. The hub miRNAs we identified might provide references for the functional study of downstream proteins in other study and some clinical targeted treatments in the future. However, due to the small sample size of this study, these results still have certain limitations. Further, in vivo and in vitro studies are needed to understand the exact molecular mechanisms that influence the development of colon cancer.

## Figures and Tables

**Table 1:** The characteristics of the dysregulated miRNAs.

Rank	miRNAs	FC	FDR	Feature
1	miR-486-5p	5.903792	0.002243	up
2	miR-451a	3.594096	0.003163	up
3	miR-378i	2.871432	0.006424	up
4	miR-126-3p	2.390553	0.007611	up
5	miR-378a-3p	2.628745	0.008606	up
6	miR-378c	2.505525	0.009621	up
7	miR-30a-5p	3.096433	0.011253	up
8	miR-378f	2.70463	0.012609	up
9	miR-422a	2.655992	0.013226	up
10	miR-139-5p	2.804786	0.013957	up
11	miR-1280	1.776806	0.034109	up
12	miR-4286	1.997162	0.034955	up
13	miR-193b-3p	2.347341	0.041491	up
14	miR-125a-5p	1.83864	0.042671	up
15	miR-182-5p	−6.34791	0.003257	down
16	miR-3687	−4.03913	0.004112	down
17	miR-503-5p	−2.85125	0.004506	down
18	miR-18b-5p	−3.16775	0.005848	down
19	miR-4417	−4.53068	0.006188	down
20	miR-1246	−4.10737	0.00864	down
21	miR-224-5p	−2.39326	0.008975	down
22	miR-200c-3p	−3.51686	0.009565	down
23	miR-552-3p	−2.65361	0.010763	down
24	miR-877-5p	−2.15946	0.011788	down
25	miR-501-5p	−1.64608	0.012554	down
26	miR-203a	−3.40787	0.013864	down
27	miR-146a-5p	−2.79816	0.015957	down
28	miR-18a-5p	−2.98152	0.017964	down
29	miR-210-3p	−2.21905	0.018626	down
30	miR-424-3p	−2.14658	0.024091	down
31	miR-1290	−2.70448	0.024521	down
32	miR-25-5p	−2.3023	0.025739	down
33	miR-4449	−1.98777	0.026635	down
34	miR-3651	−2.38984	0.026706	down
35	miR-141-3p	−2.92073	0.027618	down
36	miR-17-5p	−1.97557	0.032908	down
37	miR-200a-3p	−2.79035	0.037529	down
38	miR-188-5p	−1.81097	0.04154	down
39	miR-106b-3p	−2.06644	0.042212	down
40	miR-130b-3p	−1.84017	0.043567	down
41	miR-21-5p	−2.79495	0.045308	down
42	miR-19a-3p	−2.02304	0.045882	down
43	miR-3648	−2.51032	0.045897	down
44	miR-708-5p	−2.86582	0.046121	down
45	miR-155-5p	−2.09222	0.048755	down
46	miR-3175	−2.52047	0.04994	down

**Table 2:** The top 10 dysregulated GOs.

Rank	GO ID	GO name	Count	FDR
1	GO:0006351	Transcription, DNA-dependent	278	1.54E−60
2	GO:0006355	Regulation of transcription, DNA-dependent	169	3.34E−27
3	GO:0007165	Signal transduction	165	1.81E−37
4	GO:0045944	Positive regulation of transcription from RNA polymerase II promoter	154	5.61E−52
5	GO:0006915	Apoptotic process	111	4.75E−27
6	GO:0045893	Positive regulation of transcription, DNA-dependent	103	1.14E−32
7	GO:0000122	Negative regulation of transcription from RNA polymerase II promoter	103	2.41E−31
8	GO:0007399	Nervous system development	75	1.70E−30
9	GO:0007411	Axon guidance	75	4.27E−27
10	GO:0006468	Protein phosphorylation	74	1.61E−24

**Table 3:** The top ten dysregulated pathways of the target genes of DE-miRNAs.

Rank	Pathway ID	Pathway name	Count	FDR
1	04010	MAPK signaling pathway	69	2.00E−28
2	05200	Pathways in cancer	64	8.44E−19
3	04151	PI3K-Akt signaling pathway	61	1.17E−15
4	05205	Proteoglycans in cancer	47	5.80E−15
5	05166	HTLV-I infection	47	2.77E−12
6	04144	Endocytosis	45	2.58E−15
7	05202	Transcriptional misregulation in cancer	38	1.97E−12
8	04722	Neurotrophin signaling pathway	33	3.08E−14
9	04360	Axon guidance	31	1.38E−11
10	04912	GnRH signaling pathway	24	6.40E−10

**Table 4:** Hub miRNAs in miRNAs-gene-networks and miRNAs-GO-networks.

Rank	miRNAs	Feature	miRNA-gene-networks degree	miRNA-GO-networks degree
1	miR-17-5p	down	277	449
2	miR-182-5p	down	177	405
3	miR-200a-3p	down	126	342

## Data Availability

The data supporting the results reported in this article can be available by contacting the corresponding author.

## Supplementary Materials

Supplementary 1 Table S1: the details of GSE98406, GSE83924, GSE48267 and GSE35834.

Supplementary 2 Table S2: putative target genes of DE-miRNAs.

Supplementary 3 Table S3: clinicopathological correlation analysis for GSE35834.
