# Supplementary material for: Screening and Functional Analysis of Hub MicroRNAs Related to Tumor Development in Colon Cancer
Source: Biomed Res Int. 2020 Jan 23;2020:3981931. doi: 10.1155/2020/3981931 (PMC6998761; doi:10.1155/2020/3981931)
Supplement: Supplementary 1 — Table S1: the details of GSE98406, GSE83924, GSE48267 and GSE35834. [file 3981931.f1.docx]

**Table S1:** **The details of GSE98406, GSE83924, GSE48267 and GSE35834.**

| Accession number of the dataset | Platform | Organism | Disease type | |
| --- | --- | --- | --- | --- |
|  |  |  | Control | Colon cancer |
| GSE98406 | GPL16384 | Homo sapiens | 7 | 14 |
| GSE83924 | GPL16384 | Homo sapiens | 20 | 20 |
| GSE48267 | GPL10850 | Homo sapiens | 82 | 61 |
| GSE35834 | GPL6955 | Homo sapiens | 23 | 55 |
