# Supplementary material for: Screening and Functional Analysis of Hub MicroRNAs Related to Tumor Development in Colon Cancer
Source: Biomed Res Int. 2020 Jan 23;2020:3981931. doi: 10.1155/2020/3981931 (PMC6998761; doi:10.1155/2020/3981931)
Supplement: Supplementary 2 — Table S2: putative target genes of DE-miRNAs. [file 3981931.f2.docx]

| Transcript ID | Gene Symbol | Accession Number |
| --- | --- | --- |
| hsa-miR-106b-3p | NCDN | NM_001014839 |
| hsa-miR-106b-3p | NCDN | NM_001014841 |
| hsa-miR-106b-3p | NCDN | NM_014284 |
| hsa-miR-106b-3p | ZNF827 | NM_178835 |
| hsa-miR-1246 | ANKFY1 | NM_016376 |
| hsa-miR-1246 | BEND4 | NM_001159547 |
| hsa-miR-1246 | BEND4 | NM_207406 |
| hsa-miR-1246 | CALM2 | NM_001743 |
| hsa-miR-1246 | CCNG2 | NM_004354 |
| hsa-miR-1246 | CLLU1 | NM_001025233 |
| hsa-miR-1246 | GNRHR | NM_000406 |
| hsa-miR-1246 | GNRHR | NM_001012763 |
| hsa-miR-1246 | GRIA1 | NM_000827 |
| hsa-miR-1246 | GRIA1 | NM_001114183 |
| hsa-miR-1246 | MC2R | NM_000529 |
| hsa-miR-1246 | METAP2 | NM_006838 |
| hsa-miR-1246 | PSD3 | NM_015310 |
| hsa-miR-1246 | PSD3 | NM_206909 |
| hsa-miR-1246 | QTRTD1 | NM_024638 |
| hsa-miR-1246 | REPS2 | NM_001080975 |
| hsa-miR-1246 | REPS2 | NM_004726 |
| hsa-miR-1246 | RTKN2 | NM_145307 |
| hsa-miR-1246 | TMEM132D | NM_133448 |
| hsa-miR-1246 | TMTC3 | NM_181783 |
| hsa-miR-1246 | UNC5B | NM_170744 |
| hsa-miR-1246 | ZC3H10 | NM_032786 |
| hsa-miR-125a-5p | ABR | NM_001092 |
| hsa-miR-125a-5p | ABR | NM_001159746 |
| hsa-miR-125a-5p | ABR | NM_021962 |
| hsa-miR-125a-5p | AGGF1 | NM_018046 |
| hsa-miR-125a-5p | ALPK3 | NM_020778 |
| hsa-miR-125a-5p | ANKRD13B | NM_152345 |
| hsa-miR-125a-5p | ANKRD33B | NM_001164440 |
| hsa-miR-125a-5p | ANKRD44 | NM_001195144 |
| hsa-miR-125a-5p | ANKRD50 | NM_001167882 |
| hsa-miR-125a-5p | ANKRD50 | NM_020337 |
| hsa-miR-125a-5p | ARHGEF1 | NM_004706 |
| hsa-miR-125a-5p | ARHGEF1 | NM_198977 |
| hsa-miR-125a-5p | ARHGEF1 | NM_199002 |
| hsa-miR-125a-5p | ARID3B | NM_006465 |
| hsa-miR-125a-5p | ATOH8 | NM_032827 |
| hsa-miR-125a-5p | BAK1 | NM_001188 |
| hsa-miR-125a-5p | BAP1 | NM_004656 |
| hsa-miR-125a-5p | BCL2L2 | NM_001199839 |
| hsa-miR-125a-5p | BCL2L2 | NM_004050 |
| hsa-miR-125a-5p | BMF | NM_001003940 |
| hsa-miR-125a-5p | BMF | NM_001003942 |
| hsa-miR-125a-5p | BMF | NM_001003943 |
| hsa-miR-125a-5p | BMF | NM_033503 |
| hsa-miR-125a-5p | BRPF1 | NM_001003694 |
| hsa-miR-125a-5p | BRPF1 | NM_004634 |
| hsa-miR-125a-5p | C10orf54 | NM_022153 |
| hsa-miR-125a-5p | C11orf57 | NM_001082969 |
| hsa-miR-125a-5p | C11orf57 | NM_001082970 |
| hsa-miR-125a-5p | C11orf57 | NM_018195 |
| hsa-miR-125a-5p | C15orf39 | NM_015492 |
| hsa-miR-125a-5p | C17orf103 | NM_152914 |
| hsa-miR-125a-5p | C19orf54 | NM_198476 |
| hsa-miR-125a-5p | CD300LF | NM_139018 |
| hsa-miR-125a-5p | CDC42BPG | NM_017525 |
| hsa-miR-125a-5p | CDKN2B | NM_004936 |
| hsa-miR-125a-5p | CLEC16A | NM_015226 |
| hsa-miR-125a-5p | CSNK2A1 | NM_001895 |
| hsa-miR-125a-5p | CSNK2A1 | NM_177559 |
| hsa-miR-125a-5p | CSNK2A1 | NM_177560 |
| hsa-miR-125a-5p | CYTH1 | NM_017456 |
| hsa-miR-125a-5p | DCTN1 | NM_001135040 |
| hsa-miR-125a-5p | DCTN1 | NM_001135041 |
| hsa-miR-125a-5p | DCTN1 | NM_001190836 |
| hsa-miR-125a-5p | DCTN1 | NM_001190837 |
| hsa-miR-125a-5p | DCTN1 | NM_004082 |
| hsa-miR-125a-5p | DCTN1 | NM_023019 |
| hsa-miR-125a-5p | DDX42 | NM_007372 |
| hsa-miR-125a-5p | DDX42 | NM_203499 |
| hsa-miR-125a-5p | DHX33 | NM_001199699 |
| hsa-miR-125a-5p | DHX33 | NM_020162 |
| hsa-miR-125a-5p | DNAJB2 | NM_006736 |
| hsa-miR-125a-5p | DNAL4 | NM_005740 |
| hsa-miR-125a-5p | DPH2 | NM_001039589 |
| hsa-miR-125a-5p | DPH2 | NM_001384 |
| hsa-miR-125a-5p | DRAM2 | NM_178454 |
| hsa-miR-125a-5p | DTX4 | NM_015177 |
| hsa-miR-125a-5p | DUSP3 | NM_004090 |
| hsa-miR-125a-5p | E2F2 | NM_004091 |
| hsa-miR-125a-5p | EDEM1 | NM_014674 |
| hsa-miR-125a-5p | ELAVL1 | NM_001419 |
| hsa-miR-125a-5p | ENTPD3 | NM_001248 |
| hsa-miR-125a-5p | FAM134C | NM_178126 |
| hsa-miR-125a-5p | FAM169B | NM_182562 |
| hsa-miR-125a-5p | FAM78A | NM_033387 |
| hsa-miR-125a-5p | FAM92B | NM_198491 |
| hsa-miR-125a-5p | FBXW4 | NM_022039 |
| hsa-miR-125a-5p | FGF9 | NM_002010 |
| hsa-miR-125a-5p | FOXS1 | NM_004118 |
| hsa-miR-125a-5p | FURIN | NM_002569 |
| hsa-miR-125a-5p | FUT4 | NM_002033 |
| hsa-miR-125a-5p | GALNT14 | NM_024572 |
| hsa-miR-125a-5p | GCNT1 | NM_001097633 |
| hsa-miR-125a-5p | GCNT1 | NM_001097634 |
| hsa-miR-125a-5p | GCNT1 | NM_001097635 |
| hsa-miR-125a-5p | GCNT1 | NM_001097636 |
| hsa-miR-125a-5p | GCNT1 | NM_001490 |
| hsa-miR-125a-5p | GGA2 | NM_015044 |
| hsa-miR-125a-5p | GGT7 | NM_178026 |
| hsa-miR-125a-5p | GK5 | NM_001039547 |
| hsa-miR-125a-5p | GMIP | NM_016573 |
| hsa-miR-125a-5p | GPC4 | NM_001448 |
| hsa-miR-125a-5p | GPR107 | NM_001136557 |
| hsa-miR-125a-5p | GPR107 | NM_001136558 |
| hsa-miR-125a-5p | GPR107 | NM_020960 |
| hsa-miR-125a-5p | GPR153 | NM_207370 |
| hsa-miR-125a-5p | GRB10 | NM_001001549 |
| hsa-miR-125a-5p | GRB10 | NM_001001550 |
| hsa-miR-125a-5p | GRB10 | NM_001001555 |
| hsa-miR-125a-5p | GRB10 | NM_005311 |
| hsa-miR-125a-5p | GTPBP2 | NM_019096 |
| hsa-miR-125a-5p | HDDC3 | NM_198527 |
| hsa-miR-125a-5p | HIF1AN | NM_017902 |
| hsa-miR-125a-5p | HNRNPUL2 | NM_001079559 |
| hsa-miR-125a-5p | HOXB3 | NM_002146 |
| hsa-miR-125a-5p | IER2 | NM_004907 |
| hsa-miR-125a-5p | IFFO1 | NM_001039670 |
| hsa-miR-125a-5p | IFFO1 | NM_001193457 |
| hsa-miR-125a-5p | IFFO1 | NM_080730 |
| hsa-miR-125a-5p | IKZF4 | NM_022465 |
| hsa-miR-125a-5p | IL6R | NM_000565 |
| hsa-miR-125a-5p | IL6R | NM_181359 |
| hsa-miR-125a-5p | IMPAD1 | NM_017813 |
| hsa-miR-125a-5p | INO80D | NM_017759 |
| hsa-miR-125a-5p | INTS7 | NM_001199809 |
| hsa-miR-125a-5p | INTS7 | NM_001199811 |
| hsa-miR-125a-5p | INTS7 | NM_001199812 |
| hsa-miR-125a-5p | INTS7 | NM_015434 |
| hsa-miR-125a-5p | IQSEC2 | NM_001111125 |
| hsa-miR-125a-5p | KCNJ12 | NM_021012 |
| hsa-miR-125a-5p | KCNK10 | NM_021161 |
| hsa-miR-125a-5p | KCNK10 | NM_138317 |
| hsa-miR-125a-5p | KCNK10 | NM_138318 |
| hsa-miR-125a-5p | KCTD15 | NM_001129994 |
| hsa-miR-125a-5p | KCTD15 | NM_001129995 |
| hsa-miR-125a-5p | KCTD15 | NM_024076 |
| hsa-miR-125a-5p | KCTD21 | NM_001029859 |
| hsa-miR-125a-5p | KHNYN | NM_015299 |
| hsa-miR-125a-5p | KIAA1244 | NM_020340 |
| hsa-miR-125a-5p | KIAA1841 | NM_001129993 |
| hsa-miR-125a-5p | KLF13 | NM_015995 |
| hsa-miR-125a-5p | KPNA6 | NM_012316 |
| hsa-miR-125a-5p | LCLAT1 | NM_001002257 |
| hsa-miR-125a-5p | LCLAT1 | NM_182551 |
| hsa-miR-125a-5p | LFNG | NM_001040167 |
| hsa-miR-125a-5p | LFNG | NM_001166355 |
| hsa-miR-125a-5p | LFNG | NM_002304 |
| hsa-miR-125a-5p | LIF | NM_002309 |
| hsa-miR-125a-5p | LIMK1 | NM_001204426 |
| hsa-miR-125a-5p | LIMK1 | NM_002314 |
| hsa-miR-125a-5p | LIN28A | NM_024674 |
| hsa-miR-125a-5p | LONRF2 | NM_198461 |
| hsa-miR-125a-5p | LRFN2 | NM_020737 |
| hsa-miR-125a-5p | LRRC10B | NM_001145077 |
| hsa-miR-125a-5p | LRRC8A | NM_001127244 |
| hsa-miR-125a-5p | LRRC8A | NM_001127245 |
| hsa-miR-125a-5p | LRRC8A | NM_019594 |
| hsa-miR-125a-5p | MAN1B1 | NM_016219 |
| hsa-miR-125a-5p | MAP2K7 | NM_145185 |
| hsa-miR-125a-5p | MAP3K10 | NM_002446 |
| hsa-miR-125a-5p | MAPK14 | NM_001315 |
| hsa-miR-125a-5p | MAPK14 | NM_139012 |
| hsa-miR-125a-5p | MAPK14 | NM_139014 |
| hsa-miR-125a-5p | MAPKAPK2 | NM_004759 |
| hsa-miR-125a-5p | MAVS | NM_001206491 |
| hsa-miR-125a-5p | MAVS | NM_020746 |
| hsa-miR-125a-5p | MED15 | NM_001003891 |
| hsa-miR-125a-5p | MED15 | NM_015889 |
| hsa-miR-125a-5p | MEGF9 | NM_001080497 |
| hsa-miR-125a-5p | MFHAS1 | NM_004225 |
| hsa-miR-125a-5p | MFN1 | NM_033540 |
| hsa-miR-125a-5p | MGAT4A | NM_012214 |
| hsa-miR-125a-5p | MLF2 | NM_005439 |
| hsa-miR-125a-5p | MMP11 | NM_005940 |
| hsa-miR-125a-5p | MSI1 | NM_002442 |
| hsa-miR-125a-5p | MTF1 | NM_005955 |
| hsa-miR-125a-5p | MTMR3 | NM_021090 |
| hsa-miR-125a-5p | MTMR3 | NM_153050 |
| hsa-miR-125a-5p | MTMR3 | NM_153051 |
| hsa-miR-125a-5p | MTUS1 | NM_001001924 |
| hsa-miR-125a-5p | MTUS1 | NM_001001925 |
| hsa-miR-125a-5p | MTUS1 | NM_001001931 |
| hsa-miR-125a-5p | MTUS1 | NM_001166393 |
| hsa-miR-125a-5p | MTUS1 | NM_020749 |
| hsa-miR-125a-5p | MUL1 | NM_024544 |
| hsa-miR-125a-5p | MYT1 | NM_004535 |
| hsa-miR-125a-5p | NIPAL4 | NM_001099287 |
| hsa-miR-125a-5p | NIPAL4 | NM_001172292 |
| hsa-miR-125a-5p | NT5DC1 | NM_152729 |
| hsa-miR-125a-5p | NUP210 | NM_024923 |
| hsa-miR-125a-5p | NXF1 | NM_001081491 |
| hsa-miR-125a-5p | ORC2 | NM_006190 |
| hsa-miR-125a-5p | PCTP | NM_001102402 |
| hsa-miR-125a-5p | PCTP | NM_021213 |
| hsa-miR-125a-5p | PDPR | NM_017990 |
| hsa-miR-125a-5p | PELI2 | NM_021255 |
| hsa-miR-125a-5p | PHC2 | NM_004427 |
| hsa-miR-125a-5p | PHC2 | NM_198040 |
| hsa-miR-125a-5p | PIP5KL1 | NM_001135219 |
| hsa-miR-125a-5p | PIP5KL1 | NM_173492 |
| hsa-miR-125a-5p | PODXL | NM_001018111 |
| hsa-miR-125a-5p | PODXL | NM_005397 |
| hsa-miR-125a-5p | PPAT | NM_002703 |
| hsa-miR-125a-5p | PPP1R12B | NM_001197131 |
| hsa-miR-125a-5p | PPP1R12B | NM_002481 |
| hsa-miR-125a-5p | PPP1R12B | NM_032103 |
| hsa-miR-125a-5p | PPP1R12B | NM_032104 |
| hsa-miR-125a-5p | PRDM1 | NM_001198 |
| hsa-miR-125a-5p | PRDM1 | NM_182907 |
| hsa-miR-125a-5p | PRRC1 | NM_130809 |
| hsa-miR-125a-5p | PSMB8 | NM_004159 |
| hsa-miR-125a-5p | PSMB8 | NM_148919 |
| hsa-miR-125a-5p | PTPN18 | NM_001142370 |
| hsa-miR-125a-5p | PTPN18 | NM_014369 |
| hsa-miR-125a-5p | PVRL2 | NM_001042724 |
| hsa-miR-125a-5p | RAB3D | NM_004283 |
| hsa-miR-125a-5p | RAB8B | NM_016530 |
| hsa-miR-125a-5p | RAPGEFL1 | NM_016339 |
| hsa-miR-125a-5p | RET | NM_020630 |
| hsa-miR-125a-5p | RHOQ | NM_012249 |
| hsa-miR-125a-5p | RIMKLA | NM_173642 |
| hsa-miR-125a-5p | RS1 | NM_000330 |
| hsa-miR-125a-5p | RUSC2 | NM_001135999 |
| hsa-miR-125a-5p | RUSC2 | NM_014806 |
| hsa-miR-125a-5p | RYBP | NM_012234 |
| hsa-miR-125a-5p | SAR1B | NM_001033503 |
| hsa-miR-125a-5p | SAR1B | NM_016103 |
| hsa-miR-125a-5p | SBNO1 | NM_001167856 |
| hsa-miR-125a-5p | SBNO1 | NM_018183 |
| hsa-miR-125a-5p | SEMA4D | NM_006378 |
| hsa-miR-125a-5p | SERTAD3 | NM_013368 |
| hsa-miR-125a-5p | SERTAD3 | NM_203344 |
| hsa-miR-125a-5p | SGSM2 | NM_001098509 |
| hsa-miR-125a-5p | SGSM2 | NM_014853 |
| hsa-miR-125a-5p | SLC24A2 | NM_001193288 |
| hsa-miR-125a-5p | SLC24A2 | NM_020344 |
| hsa-miR-125a-5p | SLC27A4 | NM_005094 |
| hsa-miR-125a-5p | SLC35A4 | NM_080670 |
| hsa-miR-125a-5p | SLC35C1 | NM_001145265 |
| hsa-miR-125a-5p | SLC35C1 | NM_001145266 |
| hsa-miR-125a-5p | SLC35C1 | NM_018389 |
| hsa-miR-125a-5p | SLC38A9 | NM_173514 |
| hsa-miR-125a-5p | SLC46A3 | NM_181785 |
| hsa-miR-125a-5p | SLC4A10 | NM_001178015 |
| hsa-miR-125a-5p | SLC4A10 | NM_001178016 |
| hsa-miR-125a-5p | SLC4A10 | NM_022058 |
| hsa-miR-125a-5p | SLC6A17 | NM_001010898 |
| hsa-miR-125a-5p | SLC7A1 | NM_003045 |
| hsa-miR-125a-5p | SLC7A6 | NM_001076785 |
| hsa-miR-125a-5p | SLC7A6 | NM_003983 |
| hsa-miR-125a-5p | SLITRK6 | NM_032229 |
| hsa-miR-125a-5p | SMAD2 | NM_001003652 |
| hsa-miR-125a-5p | SMAD2 | NM_001135937 |
| hsa-miR-125a-5p | SMAD2 | NM_005901 |
| hsa-miR-125a-5p | SMARCD2 | NM_001098426 |
| hsa-miR-125a-5p | SMCR8 | NM_144775 |
| hsa-miR-125a-5p | SMG1 | NM_015092 |
| hsa-miR-125a-5p | SMG5 | NM_015327 |
| hsa-miR-125a-5p | SOX11 | NM_003108 |
| hsa-miR-125a-5p | SPEG | NM_005876 |
| hsa-miR-125a-5p | SRRM3 | NM_001110199 |
| hsa-miR-125a-5p | SSTR3 | NM_001051 |
| hsa-miR-125a-5p | ST6GAL1 | NM_003032 |
| hsa-miR-125a-5p | ST6GAL1 | NM_173216 |
| hsa-miR-125a-5p | ST6GAL1 | NM_173217 |
| hsa-miR-125a-5p | STARD13 | NM_052851 |
| hsa-miR-125a-5p | STARD13 | NM_178006 |
| hsa-miR-125a-5p | STARD13 | NM_178007 |
| hsa-miR-125a-5p | STMN3 | NM_015894 |
| hsa-miR-125a-5p | SULT4A1 | NM_014351 |
| hsa-miR-125a-5p | SUV39H1 | NM_003173 |
| hsa-miR-125a-5p | SUV420H2 | NM_032701 |
| hsa-miR-125a-5p | SYN2 | NM_133625 |
| hsa-miR-125a-5p | TAF9B | NM_015975 |
| hsa-miR-125a-5p | TBC1D8B | NM_017752 |
| hsa-miR-125a-5p | TDG | NM_003211 |
| hsa-miR-125a-5p | TMEM101 | NM_032376 |
| hsa-miR-125a-5p | TMEM120B | NM_001080825 |
| hsa-miR-125a-5p | TMEM198 | NM_001005209 |
| hsa-miR-125a-5p | TMEM86A | NM_153347 |
| hsa-miR-125a-5p | TNFAIP3 | NM_006290 |
| hsa-miR-125a-5p | TNFSF4 | NM_003326 |
| hsa-miR-125a-5p | TRIL | NM_014817 |
| hsa-miR-125a-5p | TRIM71 | NM_001039111 |
| hsa-miR-125a-5p | TSTD2 | NM_139246 |
| hsa-miR-125a-5p | TTC7A | NM_020458 |
| hsa-miR-125a-5p | TYSND1 | NM_001040273 |
| hsa-miR-125a-5p | TYSND1 | NM_173555 |
| hsa-miR-125a-5p | USP2 | NM_004205 |
| hsa-miR-125a-5p | USP2 | NM_171997 |
| hsa-miR-125a-5p | USP45 | NM_001080481 |
| hsa-miR-125a-5p | USP46 | NM_001134223 |
| hsa-miR-125a-5p | USP46 | NM_022832 |
| hsa-miR-125a-5p | USP8 | NM_001128610 |
| hsa-miR-125a-5p | USP8 | NM_005154 |
| hsa-miR-125a-5p | VAX1 | NM_199131 |
| hsa-miR-125a-5p | VEGFA | NM_001171623 |
| hsa-miR-125a-5p | VEGFA | NM_001171625 |
| hsa-miR-125a-5p | VEGFA | NM_001171626 |
| hsa-miR-125a-5p | VEGFA | NM_001171627 |
| hsa-miR-125a-5p | VEGFA | NM_001171628 |
| hsa-miR-125a-5p | VEGFA | NM_001171630 |
| hsa-miR-125a-5p | VEGFA | NM_001204385 |
| hsa-miR-125a-5p | VEGFA | NM_003376 |
| hsa-miR-125a-5p | VPS37B | NM_024667 |
| hsa-miR-125a-5p | WIPF2 | NM_133264 |
| hsa-miR-125a-5p | ZBTB33 | NM_001184742 |
| hsa-miR-125a-5p | ZBTB33 | NM_006777 |
| hsa-miR-125a-5p | ZC3H7B | NM_017590 |
| hsa-miR-125a-5p | ZFYVE1 | NM_021260 |
| hsa-miR-125a-5p | ZFYVE1 | NM_178441 |
| hsa-miR-125a-5p | ZMYM2 | NM_001190964 |
| hsa-miR-125a-5p | ZMYM2 | NM_001190965 |
| hsa-miR-125a-5p | ZMYM2 | NM_003453 |
| hsa-miR-125a-5p | ZMYM2 | NM_197968 |
| hsa-miR-125a-5p | ZNF460 | NM_006635 |
| hsa-miR-125a-5p | ZNF624 | NM_020787 |
| hsa-miR-125a-5p | ZNF704 | NM_001033723 |
| hsa-miR-125a-5p | ZNRF3 | NM_001206998 |
| hsa-miR-125a-5p | ZNRF3 | NM_032173 |
| hsa-miR-125a-5p | ZSWIM4 | NM_023072 |
| hsa-miR-126-3p | KANK2 | NM_001136191 |
| hsa-miR-126-3p | KANK2 | NM_015493 |
| hsa-miR-1290 | ABCC6 | NM_001079528 |
| hsa-miR-1290 | ANKRD13C | NM_030816 |
| hsa-miR-1290 | ANTXR1 | NM_032208 |
| hsa-miR-1290 | BBS9 | NM_001033604 |
| hsa-miR-1290 | BBS9 | NM_001033605 |
| hsa-miR-1290 | BBS9 | NM_014451 |
| hsa-miR-1290 | BBS9 | NM_198428 |
| hsa-miR-1290 | C11orf54 | NM_014039 |
| hsa-miR-1290 | DTL | NM_016448 |
| hsa-miR-1290 | EIF4B | NM_001417 |
| hsa-miR-1290 | GOLGA1 | NM_002077 |
| hsa-miR-1290 | MTMR4 | NM_004687 |
| hsa-miR-1290 | NEK11 | NM_001146003 |
| hsa-miR-1290 | NEK11 | NM_024800 |
| hsa-miR-1290 | NOTCH1 | NM_017617 |
| hsa-miR-1290 | PICALM | NM_001008660 |
| hsa-miR-1290 | PICALM | NM_001206946 |
| hsa-miR-1290 | PICALM | NM_001206947 |
| hsa-miR-1290 | PICALM | NM_007166 |
| hsa-miR-1290 | RTKN2 | NM_145307 |
| hsa-miR-1290 | STXBP5L | NM_014980 |
| hsa-miR-1290 | SYNPO2 | NM_001128933 |
| hsa-miR-1290 | TMEM155 | NM_152399 |
| hsa-miR-1290 | TMEM161B | NM_153354 |
| hsa-miR-1290 | UNC5B | NM_170744 |
| hsa-miR-1290 | ZNF24 | NM_006965 |
| hsa-miR-130b-3p | AAK1 | NM_014911 |
| hsa-miR-130b-3p | ABCA1 | NM_005502 |
| hsa-miR-130b-3p | ABCC5 | NM_005688 |
| hsa-miR-130b-3p | ABCE1 | NM_001040876 |
| hsa-miR-130b-3p | ABCE1 | NM_002940 |
| hsa-miR-130b-3p | ACVR1 | NM_001105 |
| hsa-miR-130b-3p | ACVR1 | NM_001111067 |
| hsa-miR-130b-3p | ADAMTS18 | NM_199355 |
| hsa-miR-130b-3p | ADAMTS19 | NM_133638 |
| hsa-miR-130b-3p | ARHGAP1 | NM_004308 |
| hsa-miR-130b-3p | ARHGAP12 | NM_018287 |
| hsa-miR-130b-3p | ARHGEF12 | NM_001198665 |
| hsa-miR-130b-3p | ARHGEF12 | NM_015313 |
| hsa-miR-130b-3p | ARL6IP1 | NM_015161 |
| hsa-miR-130b-3p | ARRDC3 | NM_020801 |
| hsa-miR-130b-3p | ATG14 | NM_014924 |
| hsa-miR-130b-3p | ATG2B | NM_018036 |
| hsa-miR-130b-3p | ATP11A | NM_015205 |
| hsa-miR-130b-3p | ATP11A | NM_032189 |
| hsa-miR-130b-3p | ATP13A3 | NM_024524 |
| hsa-miR-130b-3p | ATP1A2 | NM_000702 |
| hsa-miR-130b-3p | B4GALT5 | NM_004776 |
| hsa-miR-130b-3p | BAHD1 | NM_014952 |
| hsa-miR-130b-3p | BAI3 | NM_001704 |
| hsa-miR-130b-3p | BBX | NM_001142568 |
| hsa-miR-130b-3p | BBX | NM_020235 |
| hsa-miR-130b-3p | BCL2L11 | NM_001204106 |
| hsa-miR-130b-3p | BCL2L11 | NM_001204107 |
| hsa-miR-130b-3p | BCL2L11 | NM_001204108 |
| hsa-miR-130b-3p | BCL2L11 | NM_001204109 |
| hsa-miR-130b-3p | BCL2L11 | NM_001204110 |
| hsa-miR-130b-3p | BCL2L11 | NM_001204111 |
| hsa-miR-130b-3p | BCL2L11 | NM_001204112 |
| hsa-miR-130b-3p | BCL2L11 | NM_006538 |
| hsa-miR-130b-3p | BCL2L11 | NM_138621 |
| hsa-miR-130b-3p | BCL2L11 | NM_138622 |
| hsa-miR-130b-3p | BCL2L11 | NM_138623 |
| hsa-miR-130b-3p | BCL2L11 | NM_138624 |
| hsa-miR-130b-3p | BCL2L11 | NM_138625 |
| hsa-miR-130b-3p | BCL2L11 | NM_138626 |
| hsa-miR-130b-3p | BCL2L11 | NM_138627 |
| hsa-miR-130b-3p | BCL2L11 | NM_207003 |
| hsa-miR-130b-3p | BMP3 | NM_001201 |
| hsa-miR-130b-3p | BTBD3 | NM_014962 |
| hsa-miR-130b-3p | BTBD3 | NM_181443 |
| hsa-miR-130b-3p | C5orf30 | NM_033211 |
| hsa-miR-130b-3p | CAMTA1 | NM_015215 |
| hsa-miR-130b-3p | CCT6A | NM_001009186 |
| hsa-miR-130b-3p | CCT6A | NM_001762 |
| hsa-miR-130b-3p | CD69 | NM_001781 |
| hsa-miR-130b-3p | CDADC1 | NM_030911 |
| hsa-miR-130b-3p | CDK19 | NM_015076 |
| hsa-miR-130b-3p | CDKN1A | NM_000389 |
| hsa-miR-130b-3p | CDKN1A | NM_001220777 |
| hsa-miR-130b-3p | CDKN1A | NM_001220778 |
| hsa-miR-130b-3p | CDKN1A | NM_078467 |
| hsa-miR-130b-3p | CDS1 | NM_001263 |
| hsa-miR-130b-3p | CEP55 | NM_001127182 |
| hsa-miR-130b-3p | CEP55 | NM_018131 |
| hsa-miR-130b-3p | CHRM2 | NM_000739 |
| hsa-miR-130b-3p | CHRM2 | NM_001006626 |
| hsa-miR-130b-3p | CHRM2 | NM_001006627 |
| hsa-miR-130b-3p | CHRM2 | NM_001006628 |
| hsa-miR-130b-3p | CHRM2 | NM_001006629 |
| hsa-miR-130b-3p | CHRM2 | NM_001006630 |
| hsa-miR-130b-3p | CHRM2 | NM_001006631 |
| hsa-miR-130b-3p | CHRM2 | NM_001006632 |
| hsa-miR-130b-3p | CLCN6 | NM_001286 |
| hsa-miR-130b-3p | CLIP1 | NM_002956 |
| hsa-miR-130b-3p | CLIP1 | NM_198240 |
| hsa-miR-130b-3p | CLTC | NM_004859 |
| hsa-miR-130b-3p | CMPK1 | NM_001136140 |
| hsa-miR-130b-3p | CMPK1 | NM_016308 |
| hsa-miR-130b-3p | CNOT6 | NM_015455 |
| hsa-miR-130b-3p | COX7A2L | NM_004718 |
| hsa-miR-130b-3p | CPEB1 | NM_001079533 |
| hsa-miR-130b-3p | CPEB1 | NM_001079534 |
| hsa-miR-130b-3p | CPEB1 | NM_001079535 |
| hsa-miR-130b-3p | CPEB1 | NM_030594 |
| hsa-miR-130b-3p | CSF1 | NM_000757 |
| hsa-miR-130b-3p | CSF1 | NM_172211 |
| hsa-miR-130b-3p | CYP2U1 | NM_183075 |
| hsa-miR-130b-3p | DCUN1D4 | NM_001040402 |
| hsa-miR-130b-3p | DCUN1D4 | NM_015115 |
| hsa-miR-130b-3p | DDX6 | NM_004397 |
| hsa-miR-130b-3p | DEPDC1 | NM_001114120 |
| hsa-miR-130b-3p | DEPDC1 | NM_017779 |
| hsa-miR-130b-3p | DICER1 | NM_001195573 |
| hsa-miR-130b-3p | DICER1 | NM_030621 |
| hsa-miR-130b-3p | DICER1 | NM_177438 |
| hsa-miR-130b-3p | DLG5 | NM_004747 |
| hsa-miR-130b-3p | DLL1 | NM_005618 |
| hsa-miR-130b-3p | DNAJC16 | NM_015291 |
| hsa-miR-130b-3p | E2F7 | NM_203394 |
| hsa-miR-130b-3p | EBF3 | NM_001005463 |
| hsa-miR-130b-3p | EFNB2 | NM_004093 |
| hsa-miR-130b-3p | EGR3 | NM_001199880 |
| hsa-miR-130b-3p | EGR3 | NM_001199881 |
| hsa-miR-130b-3p | EGR3 | NM_004430 |
| hsa-miR-130b-3p | EIF5A2 | NM_020390 |
| hsa-miR-130b-3p | ENPP5 | NM_021572 |
| hsa-miR-130b-3p | EPS15 | NM_001159969 |
| hsa-miR-130b-3p | EPS15 | NM_001981 |
| hsa-miR-130b-3p | ERBB2IP | NM_001006600 |
| hsa-miR-130b-3p | ERBB2IP | NM_018695 |
| hsa-miR-130b-3p | ERBB3 | NM_001982 |
| hsa-miR-130b-3p | EREG | NM_001432 |
| hsa-miR-130b-3p | ESR1 | NM_000125 |
| hsa-miR-130b-3p | ESR1 | NM_001122740 |
| hsa-miR-130b-3p | ESR1 | NM_001122741 |
| hsa-miR-130b-3p | ESR1 | NM_001122742 |
| hsa-miR-130b-3p | FAM104A | NM_001098832 |
| hsa-miR-130b-3p | FAM104A | NM_032837 |
| hsa-miR-130b-3p | FAM107B | NM_031453 |
| hsa-miR-130b-3p | FAM179B | NM_015091 |
| hsa-miR-130b-3p | FAM73A | NM_198549 |
| hsa-miR-130b-3p | FBXO28 | NM_001136115 |
| hsa-miR-130b-3p | FBXO28 | NM_015176 |
| hsa-miR-130b-3p | FMR1 | NM_001185075 |
| hsa-miR-130b-3p | FMR1 | NM_001185076 |
| hsa-miR-130b-3p | FMR1 | NM_001185081 |
| hsa-miR-130b-3p | FMR1 | NM_001185082 |
| hsa-miR-130b-3p | FMR1 | NM_002024 |
| hsa-miR-130b-3p | FOXP1 | NM_032682 |
| hsa-miR-130b-3p | FRMD6 | NM_001042481 |
| hsa-miR-130b-3p | FRMD6 | NM_152330 |
| hsa-miR-130b-3p | FSTL5 | NM_001128427 |
| hsa-miR-130b-3p | FSTL5 | NM_001128428 |
| hsa-miR-130b-3p | FSTL5 | NM_020116 |
| hsa-miR-130b-3p | GOLT1B | NM_016072 |
| hsa-miR-130b-3p | GPR116 | NM_001098518 |
| hsa-miR-130b-3p | GPR116 | NM_015234 |
| hsa-miR-130b-3p | GPR158 | NM_020752 |
| hsa-miR-130b-3p | HCFC2 | NM_013320 |
| hsa-miR-130b-3p | HEG1 | NM_020733 |
| hsa-miR-130b-3p | HOXD1 | NM_024501 |
| hsa-miR-130b-3p | IGFBP5 | NM_000599 |
| hsa-miR-130b-3p | IL6ST | NM_001190981 |
| hsa-miR-130b-3p | IL6ST | NM_002184 |
| hsa-miR-130b-3p | INHBB | NM_002193 |
| hsa-miR-130b-3p | INO80 | NM_017553 |
| hsa-miR-130b-3p | IRF1 | NM_002198 |
| hsa-miR-130b-3p | ITFG3 | NM_032039 |
| hsa-miR-130b-3p | JARID2 | NM_004973 |
| hsa-miR-130b-3p | KCNJ10 | NM_002241 |
| hsa-miR-130b-3p | KDM2A | NM_012308 |
| hsa-miR-130b-3p | KLF3 | NM_016531 |
| hsa-miR-130b-3p | LCOR | NM_001170765 |
| hsa-miR-130b-3p | LCOR | NM_001170766 |
| hsa-miR-130b-3p | LCOR | NM_032440 |
| hsa-miR-130b-3p | LCORL | NM_001166139 |
| hsa-miR-130b-3p | LDLR | NM_000527 |
| hsa-miR-130b-3p | LDLR | NM_001195798 |
| hsa-miR-130b-3p | LDLR | NM_001195799 |
| hsa-miR-130b-3p | LDLR | NM_001195800 |
| hsa-miR-130b-3p | LDLR | NM_001195803 |
| hsa-miR-130b-3p | LDLRAD3 | NM_174902 |
| hsa-miR-130b-3p | LMTK2 | NM_014916 |
| hsa-miR-130b-3p | LRIG1 | NM_015541 |
| hsa-miR-130b-3p | LRP2 | NM_004525 |
| hsa-miR-130b-3p | LRRTM2 | NM_015564 |
| hsa-miR-130b-3p | LYSMD3 | NM_198273 |
| hsa-miR-130b-3p | MAP3K9 | NM_033141 |
| hsa-miR-130b-3p | MAPK1 | NM_002745 |
| hsa-miR-130b-3p | MDM4 | NM_001204171 |
| hsa-miR-130b-3p | MDM4 | NM_001204172 |
| hsa-miR-130b-3p | MDM4 | NM_002393 |
| hsa-miR-130b-3p | MED12L | NM_053002 |
| hsa-miR-130b-3p | MET | NM_000245 |
| hsa-miR-130b-3p | MET | NM_001127500 |
| hsa-miR-130b-3p | MIER1 | NM_001077700 |
| hsa-miR-130b-3p | MIER1 | NM_001077701 |
| hsa-miR-130b-3p | MIER1 | NM_001077702 |
| hsa-miR-130b-3p | MIER1 | NM_001077703 |
| hsa-miR-130b-3p | MIER1 | NM_001077704 |
| hsa-miR-130b-3p | MIER1 | NM_001146110 |
| hsa-miR-130b-3p | MIER1 | NM_001146111 |
| hsa-miR-130b-3p | MIER1 | NM_001146112 |
| hsa-miR-130b-3p | MIER1 | NM_001146113 |
| hsa-miR-130b-3p | MIER1 | NM_020948 |
| hsa-miR-130b-3p | MLLT10 | NM_001195626 |
| hsa-miR-130b-3p | MLLT10 | NM_004641 |
| hsa-miR-130b-3p | MLLT6 | NM_005937 |
| hsa-miR-130b-3p | MMGT1 | NM_173470 |
| hsa-miR-130b-3p | MPPED2 | NM_001584 |
| hsa-miR-130b-3p | MTMR9 | NM_015458 |
| hsa-miR-130b-3p | MUM1L1 | NM_001171020 |
| hsa-miR-130b-3p | MUM1L1 | NM_152423 |
| hsa-miR-130b-3p | MYB | NM_001130172 |
| hsa-miR-130b-3p | MYB | NM_001130173 |
| hsa-miR-130b-3p | MYB | NM_001161656 |
| hsa-miR-130b-3p | MYB | NM_001161657 |
| hsa-miR-130b-3p | MYB | NM_001161658 |
| hsa-miR-130b-3p | MYB | NM_001161659 |
| hsa-miR-130b-3p | MYB | NM_001161660 |
| hsa-miR-130b-3p | MYB | NM_005375 |
| hsa-miR-130b-3p | MYBL1 | NM_001080416 |
| hsa-miR-130b-3p | MYBL1 | NM_001144755 |
| hsa-miR-130b-3p | NBEA | NM_001204197 |
| hsa-miR-130b-3p | NBEA | NM_015678 |
| hsa-miR-130b-3p | NCOA1 | NM_003743 |
| hsa-miR-130b-3p | NCOA1 | NM_147223 |
| hsa-miR-130b-3p | NCOA1 | NM_147233 |
| hsa-miR-130b-3p | NDEL1 | NM_001025579 |
| hsa-miR-130b-3p | NDEL1 | NM_030808 |
| hsa-miR-130b-3p | NEUROG1 | NM_006161 |
| hsa-miR-130b-3p | NHLH2 | NM_001111061 |
| hsa-miR-130b-3p | NHLH2 | NM_005599 |
| hsa-miR-130b-3p | NHS | NM_001136024 |
| hsa-miR-130b-3p | NHS | NM_198270 |
| hsa-miR-130b-3p | NIPA1 | NM_001142275 |
| hsa-miR-130b-3p | NIPA1 | NM_144599 |
| hsa-miR-130b-3p | NPEPL1 | NM_001204872 |
| hsa-miR-130b-3p | NPEPL1 | NM_001204873 |
| hsa-miR-130b-3p | NPEPL1 | NM_024663 |
| hsa-miR-130b-3p | NPNT | NM_001033047 |
| hsa-miR-130b-3p | NPNT | NM_001184690 |
| hsa-miR-130b-3p | NPNT | NM_001184691 |
| hsa-miR-130b-3p | NPNT | NM_001184692 |
| hsa-miR-130b-3p | NPNT | NM_001184693 |
| hsa-miR-130b-3p | NRSN1 | NM_080723 |
| hsa-miR-130b-3p | OCRL | NM_000276 |
| hsa-miR-130b-3p | OCRL | NM_001587 |
| hsa-miR-130b-3p | OTUD3 | NM_015207 |
| hsa-miR-130b-3p | PCYOX1 | NM_016297 |
| hsa-miR-130b-3p | PHF12 | NM_001033561 |
| hsa-miR-130b-3p | PHF20 | NM_016436 |
| hsa-miR-130b-3p | PHF3 | NM_015153 |
| hsa-miR-130b-3p | PIGA | NM_002641 |
| hsa-miR-130b-3p | PIGA | NM_020473 |
| hsa-miR-130b-3p | PPARG | NM_005037 |
| hsa-miR-130b-3p | PPARG | NM_015869 |
| hsa-miR-130b-3p | PPARG | NM_138711 |
| hsa-miR-130b-3p | PPARG | NM_138712 |
| hsa-miR-130b-3p | PRKD3 | NM_005813 |
| hsa-miR-130b-3p | PRUNE2 | NM_015225 |
| hsa-miR-130b-3p | PTGES3 | NM_006601 |
| hsa-miR-130b-3p | RAB5A | NM_004162 |
| hsa-miR-130b-3p | RAB9B | NM_016370 |
| hsa-miR-130b-3p | RALGPS1 | NM_014636 |
| hsa-miR-130b-3p | RBM20 | NM_001134363 |
| hsa-miR-130b-3p | RFX7 | NM_022841 |
| hsa-miR-130b-3p | RNF180 | NM_001113561 |
| hsa-miR-130b-3p | RNF38 | NM_022781 |
| hsa-miR-130b-3p | RNF38 | NM_194328 |
| hsa-miR-130b-3p | RNF38 | NM_194329 |
| hsa-miR-130b-3p | RNF38 | NM_194330 |
| hsa-miR-130b-3p | RNF38 | NM_194332 |
| hsa-miR-130b-3p | ROBO2 | NM_002942 |
| hsa-miR-130b-3p | RPA2 | NM_002946 |
| hsa-miR-130b-3p | RPRD1A | NM_018170 |
| hsa-miR-130b-3p | RXFP2 | NM_001166058 |
| hsa-miR-130b-3p | RXFP2 | NM_130806 |
| hsa-miR-130b-3p | S1PR1 | NM_001400 |
| hsa-miR-130b-3p | SASH1 | NM_015278 |
| hsa-miR-130b-3p | SBF2 | NM_030962 |
| hsa-miR-130b-3p | SDR42E1 | NM_145168 |
| hsa-miR-130b-3p | SECISBP2L | NM_001193489 |
| hsa-miR-130b-3p | SECISBP2L | NM_014701 |
| hsa-miR-130b-3p | SESTD1 | NM_178123 |
| hsa-miR-130b-3p | SFMBT1 | NM_016329 |
| hsa-miR-130b-3p | SGCB | NM_000232 |
| hsa-miR-130b-3p | SIK1 | NM_173354 |
| hsa-miR-130b-3p | SLC24A2 | NM_001193288 |
| hsa-miR-130b-3p | SLC24A2 | NM_020344 |
| hsa-miR-130b-3p | SLC25A44 | NM_014655 |
| hsa-miR-130b-3p | SLC4A5 | NM_133478 |
| hsa-miR-130b-3p | SMAD5 | NM_001001419 |
| hsa-miR-130b-3p | SMAD5 | NM_001001420 |
| hsa-miR-130b-3p | SMAD5 | NM_005903 |
| hsa-miR-130b-3p | SMARCD2 | NM_001098426 |
| hsa-miR-130b-3p | SMOC1 | NM_001034852 |
| hsa-miR-130b-3p | SMOC1 | NM_022137 |
| hsa-miR-130b-3p | SNAPIN | NM_012437 |
| hsa-miR-130b-3p | SNX27 | NM_030918 |
| hsa-miR-130b-3p | SNX5 | NM_014426 |
| hsa-miR-130b-3p | SNX5 | NM_152227 |
| hsa-miR-130b-3p | SOCS6 | NM_004232 |
| hsa-miR-130b-3p | SOS2 | NM_006939 |
| hsa-miR-130b-3p | SPG20 | NM_001142294 |
| hsa-miR-130b-3p | SPG20 | NM_001142295 |
| hsa-miR-130b-3p | SPG20 | NM_001142296 |
| hsa-miR-130b-3p | SPG20 | NM_015087 |
| hsa-miR-130b-3p | SPIRE1 | NM_001128626 |
| hsa-miR-130b-3p | SPIRE1 | NM_001128627 |
| hsa-miR-130b-3p | SPIRE1 | NM_020148 |
| hsa-miR-130b-3p | SPOCK1 | NM_004598 |
| hsa-miR-130b-3p | SPOPL | NM_001001664 |
| hsa-miR-130b-3p | SPTY2D1 | NM_194285 |
| hsa-miR-130b-3p | SRSF2 | NM_003016 |
| hsa-miR-130b-3p | SRSF7 | NM_001031684 |
| hsa-miR-130b-3p | SRSF7 | NM_001195446 |
| hsa-miR-130b-3p | ST18 | NM_014682 |
| hsa-miR-130b-3p | ST8SIA3 | NM_015879 |
| hsa-miR-130b-3p | STARD13 | NM_052851 |
| hsa-miR-130b-3p | STARD13 | NM_178006 |
| hsa-miR-130b-3p | STARD13 | NM_178007 |
| hsa-miR-130b-3p | STAT3 | NM_003150 |
| hsa-miR-130b-3p | STAT3 | NM_139276 |
| hsa-miR-130b-3p | STAT3 | NM_213662 |
| hsa-miR-130b-3p | STK4 | NM_006282 |
| hsa-miR-130b-3p | STX12 | NM_177424 |
| hsa-miR-130b-3p | SYT10 | NM_198992 |
| hsa-miR-130b-3p | SYT6 | NM_205848 |
| hsa-miR-130b-3p | TAF4 | NM_003185 |
| hsa-miR-130b-3p | TAF4B | NM_005640 |
| hsa-miR-130b-3p | TANC1 | NM_001145909 |
| hsa-miR-130b-3p | TANC1 | NM_033394 |
| hsa-miR-130b-3p | TCF4 | NM_001083962 |
| hsa-miR-130b-3p | TCF4 | NM_003199 |
| hsa-miR-130b-3p | TES | NM_015641 |
| hsa-miR-130b-3p | TES | NM_152829 |
| hsa-miR-130b-3p | TFDP2 | NM_001178138 |
| hsa-miR-130b-3p | TFDP2 | NM_001178139 |
| hsa-miR-130b-3p | TFDP2 | NM_001178140 |
| hsa-miR-130b-3p | TFDP2 | NM_001178141 |
| hsa-miR-130b-3p | TFDP2 | NM_001178142 |
| hsa-miR-130b-3p | TFDP2 | NM_006286 |
| hsa-miR-130b-3p | TGFA | NM_001099691 |
| hsa-miR-130b-3p | TGFA | NM_003236 |
| hsa-miR-130b-3p | TGFBR1 | NM_001130916 |
| hsa-miR-130b-3p | TGFBR1 | NM_004612 |
| hsa-miR-130b-3p | TGFBR2 | NM_001024847 |
| hsa-miR-130b-3p | TGFBR2 | NM_003242 |
| hsa-miR-130b-3p | THOP1 | NM_003249 |
| hsa-miR-130b-3p | TMEM110 | NM_198563 |
| hsa-miR-130b-3p | TMEM159 | NM_020422 |
| hsa-miR-130b-3p | TMEM170B | NM_001100829 |
| hsa-miR-130b-3p | TMEM55A | NM_018710 |
| hsa-miR-130b-3p | TMEM9B | NM_020644 |
| hsa-miR-130b-3p | TNF | NM_000594 |
| hsa-miR-130b-3p | TNRC6A | NM_014494 |
| hsa-miR-130b-3p | TNRC6B | NM_001024843 |
| hsa-miR-130b-3p | TNRC6B | NM_001162501 |
| hsa-miR-130b-3p | TNRC6B | NM_015088 |
| hsa-miR-130b-3p | TPP1 | NM_000391 |
| hsa-miR-130b-3p | TRIM2 | NM_001130067 |
| hsa-miR-130b-3p | TRIM2 | NM_015271 |
| hsa-miR-130b-3p | TRPC3 | NM_001130698 |
| hsa-miR-130b-3p | TRPC3 | NM_003305 |
| hsa-miR-130b-3p | TSC1 | NM_000368 |
| hsa-miR-130b-3p | TSC1 | NM_001162426 |
| hsa-miR-130b-3p | TSC1 | NM_001162427 |
| hsa-miR-130b-3p | TSHZ1 | NM_005786 |
| hsa-miR-130b-3p | TSHZ2 | NM_001193421 |
| hsa-miR-130b-3p | TSHZ2 | NM_173485 |
| hsa-miR-130b-3p | TTYH3 | NM_025250 |
| hsa-miR-130b-3p | UCP3 | NM_003356 |
| hsa-miR-130b-3p | ULK2 | NM_014683 |
| hsa-miR-130b-3p | WASL | NM_003941 |
| hsa-miR-130b-3p | WDR47 | NM_001142550 |
| hsa-miR-130b-3p | WDR47 | NM_001142551 |
| hsa-miR-130b-3p | WDR47 | NM_014969 |
| hsa-miR-130b-3p | YTHDF2 | NM_001172828 |
| hsa-miR-130b-3p | YTHDF2 | NM_001173128 |
| hsa-miR-130b-3p | YTHDF2 | NM_016258 |
| hsa-miR-130b-3p | ZFYVE9 | NM_004799 |
| hsa-miR-130b-3p | ZFYVE9 | NM_007324 |
| hsa-miR-130b-3p | ZNF148 | NM_021964 |
| hsa-miR-130b-3p | ZNF217 | NM_006526 |
| hsa-miR-139-5p | AEBP2 | NM_001114176 |
| hsa-miR-139-5p | AEBP2 | NM_153207 |
| hsa-miR-139-5p | AJAP1 | NM_018836 |
| hsa-miR-139-5p | ANK2 | NM_001127493 |
| hsa-miR-139-5p | ANK2 | NM_001148 |
| hsa-miR-139-5p | ANK2 | NM_020977 |
| hsa-miR-139-5p | APLP2 | NM_001142276 |
| hsa-miR-139-5p | APLP2 | NM_001142277 |
| hsa-miR-139-5p | APLP2 | NM_001142278 |
| hsa-miR-139-5p | APLP2 | NM_001642 |
| hsa-miR-139-5p | ASH1L | NM_018489 |
| hsa-miR-139-5p | ATXN1 | NM_000332 |
| hsa-miR-139-5p | ATXN1 | NM_001128164 |
| hsa-miR-139-5p | BAZ2B | NM_013450 |
| hsa-miR-139-5p | CACNA2D2 | NM_001174051 |
| hsa-miR-139-5p | CACNA2D2 | NM_006030 |
| hsa-miR-139-5p | CNOT1 | NM_016284 |
| hsa-miR-139-5p | CUL3 | NM_003590 |
| hsa-miR-139-5p | CUX1 | NM_001202543 |
| hsa-miR-139-5p | CUX1 | NM_181552 |
| hsa-miR-139-5p | DCBLD2 | NM_080927 |
| hsa-miR-139-5p | DCC | NM_005215 |
| hsa-miR-139-5p | DDIT4 | NM_019058 |
| hsa-miR-139-5p | DDX3X | NM_001193416 |
| hsa-miR-139-5p | DDX3X | NM_001193417 |
| hsa-miR-139-5p | DDX3X | NM_001356 |
| hsa-miR-139-5p | DIP2C | NM_014974 |
| hsa-miR-139-5p | DMD | NM_000109 |
| hsa-miR-139-5p | DMD | NM_004006 |
| hsa-miR-139-5p | DMD | NM_004009 |
| hsa-miR-139-5p | DMD | NM_004010 |
| hsa-miR-139-5p | DMD | NM_004011 |
| hsa-miR-139-5p | DMD | NM_004012 |
| hsa-miR-139-5p | DMD | NM_004013 |
| hsa-miR-139-5p | DMD | NM_004014 |
| hsa-miR-139-5p | DMD | NM_004015 |
| hsa-miR-139-5p | DMD | NM_004016 |
| hsa-miR-139-5p | DMD | NM_004017 |
| hsa-miR-139-5p | DMD | NM_004018 |
| hsa-miR-139-5p | DMD | NM_004020 |
| hsa-miR-139-5p | DMD | NM_004021 |
| hsa-miR-139-5p | DMD | NM_004022 |
| hsa-miR-139-5p | DMD | NM_004023 |
| hsa-miR-139-5p | DNAJC5 | NM_025219 |
| hsa-miR-139-5p | DPYSL5 | NM_020134 |
| hsa-miR-139-5p | DTX3 | NM_178502 |
| hsa-miR-139-5p | DUSP19 | NM_001142314 |
| hsa-miR-139-5p | DUSP19 | NM_080876 |
| hsa-miR-139-5p | EBF1 | NM_024007 |
| hsa-miR-139-5p | EIF4G2 | NM_001042559 |
| hsa-miR-139-5p | EIF4G2 | NM_001172705 |
| hsa-miR-139-5p | EIF4G2 | NM_001418 |
| hsa-miR-139-5p | ESRRG | NM_001134285 |
| hsa-miR-139-5p | ESRRG | NM_001438 |
| hsa-miR-139-5p | ESRRG | NM_206594 |
| hsa-miR-139-5p | ESRRG | NM_206595 |
| hsa-miR-139-5p | FBN2 | NM_001999 |
| hsa-miR-139-5p | FOS | NM_005252 |
| hsa-miR-139-5p | FOXO1 | NM_002015 |
| hsa-miR-139-5p | GALNT3 | NM_004482 |
| hsa-miR-139-5p | GDE1 | NM_016641 |
| hsa-miR-139-5p | GNAI3 | NM_006496 |
| hsa-miR-139-5p | GPR56 | NM_001145770 |
| hsa-miR-139-5p | GPR56 | NM_001145771 |
| hsa-miR-139-5p | GPR56 | NM_001145772 |
| hsa-miR-139-5p | GPR56 | NM_001145773 |
| hsa-miR-139-5p | GPR56 | NM_001145774 |
| hsa-miR-139-5p | GPR56 | NM_005682 |
| hsa-miR-139-5p | GPR56 | NM_201524 |
| hsa-miR-139-5p | GPR56 | NM_201525 |
| hsa-miR-139-5p | H2AFV | NM_012412 |
| hsa-miR-139-5p | H2AFV | NM_201436 |
| hsa-miR-139-5p | H2AFV | NM_201516 |
| hsa-miR-139-5p | H2AFV | NM_201517 |
| hsa-miR-139-5p | HNRNPF | NM_001098204 |
| hsa-miR-139-5p | HNRNPF | NM_001098205 |
| hsa-miR-139-5p | HNRNPF | NM_001098206 |
| hsa-miR-139-5p | HNRNPF | NM_001098207 |
| hsa-miR-139-5p | HNRNPF | NM_001098208 |
| hsa-miR-139-5p | HNRNPF | NM_004966 |
| hsa-miR-139-5p | ITGA3 | NM_002204 |
| hsa-miR-139-5p | ITGA3 | NM_005501 |
| hsa-miR-139-5p | KDM3B | NM_016604 |
| hsa-miR-139-5p | KIAA2018 | NM_001009899 |
| hsa-miR-139-5p | LCOR | NM_001170765 |
| hsa-miR-139-5p | LCOR | NM_001170766 |
| hsa-miR-139-5p | LCOR | NM_032440 |
| hsa-miR-139-5p | LRCH2 | NM_020871 |
| hsa-miR-139-5p | 43531 | NM_022826 |
| hsa-miR-139-5p | MBD1 | NM_001204139 |
| hsa-miR-139-5p | MEIS2 | NM_001220482 |
| hsa-miR-139-5p | MEIS2 | NM_002399 |
| hsa-miR-139-5p | MEIS2 | NM_170674 |
| hsa-miR-139-5p | MEIS2 | NM_170675 |
| hsa-miR-139-5p | MEIS2 | NM_170676 |
| hsa-miR-139-5p | MEIS2 | NM_170677 |
| hsa-miR-139-5p | MEIS2 | NM_172315 |
| hsa-miR-139-5p | MEIS2 | NM_172316 |
| hsa-miR-139-5p | MEX3A | NM_001093725 |
| hsa-miR-139-5p | MFSD6 | NM_017694 |
| hsa-miR-139-5p | MLLT10 | NM_001195626 |
| hsa-miR-139-5p | MLLT10 | NM_004641 |
| hsa-miR-139-5p | MLLT4 | NM_001040000 |
| hsa-miR-139-5p | MORN4 | NM_001098831 |
| hsa-miR-139-5p | MORN4 | NM_178832 |
| hsa-miR-139-5p | MOSPD1 | NM_019556 |
| hsa-miR-139-5p | MRVI1 | NM_001098579 |
| hsa-miR-139-5p | MRVI1 | NM_001100163 |
| hsa-miR-139-5p | MRVI1 | NM_001100167 |
| hsa-miR-139-5p | MRVI1 | NM_001206880 |
| hsa-miR-139-5p | MRVI1 | NM_001206881 |
| hsa-miR-139-5p | MRVI1 | NM_130385 |
| hsa-miR-139-5p | NFIB | NM_001190737 |
| hsa-miR-139-5p | NFIB | NM_001190738 |
| hsa-miR-139-5p | NFIB | NM_005596 |
| hsa-miR-139-5p | NRK | NM_198465 |
| hsa-miR-139-5p | PCDH10 | NM_032961 |
| hsa-miR-139-5p | PCDH11X | NM_001168360 |
| hsa-miR-139-5p | PCDH11X | NM_001168362 |
| hsa-miR-139-5p | PCDH11X | NM_001168363 |
| hsa-miR-139-5p | PCDH11X | NM_032968 |
| hsa-miR-139-5p | PCDH11X | NM_032969 |
| hsa-miR-139-5p | PDE3A | NM_000921 |
| hsa-miR-139-5p | PDE4A | NM_001111307 |
| hsa-miR-139-5p | PDE4A | NM_001111308 |
| hsa-miR-139-5p | PDE4A | NM_001111309 |
| hsa-miR-139-5p | PDE4A | NM_006202 |
| hsa-miR-139-5p | PPARGC1B | NM_001172698 |
| hsa-miR-139-5p | PPARGC1B | NM_001172699 |
| hsa-miR-139-5p | PPARGC1B | NM_133263 |
| hsa-miR-139-5p | PRDM10 | NM_020228 |
| hsa-miR-139-5p | PRDM10 | NM_199437 |
| hsa-miR-139-5p | PRDM10 | NM_199438 |
| hsa-miR-139-5p | PRDM10 | NM_199439 |
| hsa-miR-139-5p | PRKD3 | NM_005813 |
| hsa-miR-139-5p | PRPF4B | NM_003913 |
| hsa-miR-139-5p | QKI | NM_206853 |
| hsa-miR-139-5p | QKI | NM_206854 |
| hsa-miR-139-5p | QKI | NM_206855 |
| hsa-miR-139-5p | RSBN1L | NM_198467 |
| hsa-miR-139-5p | 43719 | NM_018243 |
| hsa-miR-139-5p | SETD8 | NM_020382 |
| hsa-miR-139-5p | SOCS2 | NM_003877 |
| hsa-miR-139-5p | SSX2IP | NM_001166293 |
| hsa-miR-139-5p | SSX2IP | NM_001166294 |
| hsa-miR-139-5p | SSX2IP | NM_001166295 |
| hsa-miR-139-5p | SSX2IP | NM_001166417 |
| hsa-miR-139-5p | SSX2IP | NM_014021 |
| hsa-miR-139-5p | ST8SIA3 | NM_015879 |
| hsa-miR-139-5p | STAMBP | NM_006463 |
| hsa-miR-139-5p | STAMBP | NM_201647 |
| hsa-miR-139-5p | STAMBP | NM_213622 |
| hsa-miR-139-5p | SYT14 | NM_001146261 |
| hsa-miR-139-5p | SYT14 | NM_001146262 |
| hsa-miR-139-5p | SYT14 | NM_001146264 |
| hsa-miR-139-5p | SYT14 | NM_153262 |
| hsa-miR-139-5p | TBX1 | NM_080647 |
| hsa-miR-139-5p | TCF4 | NM_001083962 |
| hsa-miR-139-5p | TCF4 | NM_003199 |
| hsa-miR-139-5p | TET2 | NM_001127208 |
| hsa-miR-139-5p | TMCC1 | NM_001017395 |
| hsa-miR-139-5p | TMCC1 | NM_001128224 |
| hsa-miR-139-5p | TMED10 | NM_006827 |
| hsa-miR-139-5p | TMEM132D | NM_133448 |
| hsa-miR-139-5p | TNPO1 | NM_002270 |
| hsa-miR-139-5p | TNPO1 | NM_153188 |
| hsa-miR-139-5p | TPM3 | NM_001043352 |
| hsa-miR-139-5p | TPM3 | NM_001043353 |
| hsa-miR-139-5p | TRIM9 | NM_052978 |
| hsa-miR-139-5p | U2SURP | NM_001080415 |
| hsa-miR-139-5p | UBE2F | NM_080678 |
| hsa-miR-139-5p | UHMK1 | NM_001184763 |
| hsa-miR-139-5p | UHMK1 | NM_144624 |
| hsa-miR-139-5p | UHMK1 | NM_175866 |
| hsa-miR-139-5p | USP24 | NM_015306 |
| hsa-miR-139-5p | YWHAG | NM_012479 |
| hsa-miR-139-5p | ZFAND3 | NM_021943 |
| hsa-miR-139-5p | ZNF532 | NM_018181 |
| hsa-miR-139-5p | ZNF770 | NM_014106 |
| hsa-miR-141-3p | ABL2 | NM_001136000 |
| hsa-miR-141-3p | ABL2 | NM_001168236 |
| hsa-miR-141-3p | ABL2 | NM_001168237 |
| hsa-miR-141-3p | ABL2 | NM_001168238 |
| hsa-miR-141-3p | ABL2 | NM_001168239 |
| hsa-miR-141-3p | ABL2 | NM_005158 |
| hsa-miR-141-3p | ABL2 | NM_007314 |
| hsa-miR-141-3p | ACOT7 | NM_007274 |
| hsa-miR-141-3p | ACOT7 | NM_181864 |
| hsa-miR-141-3p | ACOT7 | NM_181865 |
| hsa-miR-141-3p | ACOT7 | NM_181866 |
| hsa-miR-141-3p | ADD3 | NM_001121 |
| hsa-miR-141-3p | ADD3 | NM_016824 |
| hsa-miR-141-3p | ADD3 | NM_019903 |
| hsa-miR-141-3p | ADRB1 | NM_000684 |
| hsa-miR-141-3p | AKAP11 | NM_016248 |
| hsa-miR-141-3p | AKAP2 | NM_001004065 |
| hsa-miR-141-3p | AKAP2 | NM_001136562 |
| hsa-miR-141-3p | AKAP2 | NM_001198656 |
| hsa-miR-141-3p | ANKFY1 | NM_016376 |
| hsa-miR-141-3p | ANP32E | NM_001136478 |
| hsa-miR-141-3p | ANP32E | NM_001136479 |
| hsa-miR-141-3p | ANP32E | NM_030920 |
| hsa-miR-141-3p | APBB2 | NM_001166050 |
| hsa-miR-141-3p | APBB2 | NM_001166052 |
| hsa-miR-141-3p | APBB2 | NM_001166054 |
| hsa-miR-141-3p | APBB2 | NM_004307 |
| hsa-miR-141-3p | APBB2 | NM_173075 |
| hsa-miR-141-3p | ARHGEF18 | NM_001130955 |
| hsa-miR-141-3p | ARHGEF18 | NM_015318 |
| hsa-miR-141-3p | ARL4A | NM_001037164 |
| hsa-miR-141-3p | ARL4A | NM_001195396 |
| hsa-miR-141-3p | ARL4A | NM_005738 |
| hsa-miR-141-3p | ARL4A | NM_212460 |
| hsa-miR-141-3p | ASXL1 | NM_015338 |
| hsa-miR-141-3p | ATP8A1 | NM_001105529 |
| hsa-miR-141-3p | ATP8A1 | NM_006095 |
| hsa-miR-141-3p | ATRN | NM_139321 |
| hsa-miR-141-3p | ATXN7L1 | NM_020725 |
| hsa-miR-141-3p | ATXN7L1 | NM_138495 |
| hsa-miR-141-3p | BICD2 | NM_001003800 |
| hsa-miR-141-3p | C1orf21 | NM_030806 |
| hsa-miR-141-3p | C3orf17 | NM_015412 |
| hsa-miR-141-3p | CACNA1B | NM_000718 |
| hsa-miR-141-3p | CALCR | NM_001164737 |
| hsa-miR-141-3p | CALCR | NM_001164738 |
| hsa-miR-141-3p | CALCR | NM_001742 |
| hsa-miR-141-3p | CBL | NM_005188 |
| hsa-miR-141-3p | CCDC6 | NM_005436 |
| hsa-miR-141-3p | CCDC80 | NM_199511 |
| hsa-miR-141-3p | CCDC80 | NM_199512 |
| hsa-miR-141-3p | CCND2 | NM_001759 |
| hsa-miR-141-3p | CCPG1 | NM_004748 |
| hsa-miR-141-3p | CCPG1 | NM_020739 |
| hsa-miR-141-3p | CDC14A | NM_003672 |
| hsa-miR-141-3p | CDC25B | NM_004358 |
| hsa-miR-141-3p | CDC25B | NM_021872 |
| hsa-miR-141-3p | CDC25B | NM_021873 |
| hsa-miR-141-3p | CHD2 | NM_001271 |
| hsa-miR-141-3p | CHD9 | NM_025134 |
| hsa-miR-141-3p | CIAO1 | NM_004804 |
| hsa-miR-141-3p | CLOCK | NM_004898 |
| hsa-miR-141-3p | CRMP1 | NM_001014809 |
| hsa-miR-141-3p | CRMP1 | NM_001313 |
| hsa-miR-141-3p | CSNK2A1 | NM_001895 |
| hsa-miR-141-3p | CSNK2A1 | NM_177559 |
| hsa-miR-141-3p | CSNK2A1 | NM_177560 |
| hsa-miR-141-3p | CTBP2 | NM_001083914 |
| hsa-miR-141-3p | CTBP2 | NM_001329 |
| hsa-miR-141-3p | CTBP2 | NM_022802 |
| hsa-miR-141-3p | CTNND2 | NM_001332 |
| hsa-miR-141-3p | CUL3 | NM_003590 |
| hsa-miR-141-3p | CUL4B | NM_001079872 |
| hsa-miR-141-3p | CUL4B | NM_003588 |
| hsa-miR-141-3p | CYP26B1 | NM_019885 |
| hsa-miR-141-3p | DCP2 | NM_001242377 |
| hsa-miR-141-3p | DCP2 | NM_152624 |
| hsa-miR-141-3p | DCUN1D3 | NM_173475 |
| hsa-miR-141-3p | DDIT4L | NM_145244 |
| hsa-miR-141-3p | DDX5 | NM_004396 |
| hsa-miR-141-3p | DIP2B | NM_173602 |
| hsa-miR-141-3p | DIS3 | NM_001128226 |
| hsa-miR-141-3p | DIS3 | NM_014953 |
| hsa-miR-141-3p | DLC1 | NM_001164271 |
| hsa-miR-141-3p | DLC1 | NM_006094 |
| hsa-miR-141-3p | DLC1 | NM_182643 |
| hsa-miR-141-3p | DNAJC13 | NM_015268 |
| hsa-miR-141-3p | DSTYK | NM_015375 |
| hsa-miR-141-3p | DSTYK | NM_199462 |
| hsa-miR-141-3p | DTL | NM_016448 |
| hsa-miR-141-3p | DUSP3 | NM_004090 |
| hsa-miR-141-3p | E2F3 | NM_001949 |
| hsa-miR-141-3p | EDEM1 | NM_014674 |
| hsa-miR-141-3p | ELAVL2 | NM_001171195 |
| hsa-miR-141-3p | ELAVL2 | NM_001171197 |
| hsa-miR-141-3p | ELAVL2 | NM_004432 |
| hsa-miR-141-3p | ELAVL4 | NM_001144774 |
| hsa-miR-141-3p | ELAVL4 | NM_001144775 |
| hsa-miR-141-3p | ELAVL4 | NM_001144776 |
| hsa-miR-141-3p | ELAVL4 | NM_001144777 |
| hsa-miR-141-3p | ELAVL4 | NM_021952 |
| hsa-miR-141-3p | ELMOD2 | NM_153702 |
| hsa-miR-141-3p | EPN1 | NM_001130071 |
| hsa-miR-141-3p | EPN1 | NM_001130072 |
| hsa-miR-141-3p | EPN1 | NM_013333 |
| hsa-miR-141-3p | EVI5L | NM_001159944 |
| hsa-miR-141-3p | EVI5L | NM_145245 |
| hsa-miR-141-3p | EXOC5 | NM_006544 |
| hsa-miR-141-3p | FAM118B | NM_024556 |
| hsa-miR-141-3p | FAM160B1 | NM_020940 |
| hsa-miR-141-3p | FAM168B | NM_001009993 |
| hsa-miR-141-3p | FBXW2 | NM_012164 |
| hsa-miR-141-3p | FKBP5 | NM_001145775 |
| hsa-miR-141-3p | FKBP5 | NM_001145776 |
| hsa-miR-141-3p | FKBP5 | NM_004117 |
| hsa-miR-141-3p | FOXA1 | NM_004496 |
| hsa-miR-141-3p | FOXC1 | NM_001453 |
| hsa-miR-141-3p | FOXJ1 | NM_001454 |
| hsa-miR-141-3p | FOXJ3 | NM_001198850 |
| hsa-miR-141-3p | FOXJ3 | NM_001198851 |
| hsa-miR-141-3p | FOXJ3 | NM_001198852 |
| hsa-miR-141-3p | FOXJ3 | NM_014947 |
| hsa-miR-141-3p | FRMD4A | NM_018027 |
| hsa-miR-141-3p | GJC1 | NM_001080383 |
| hsa-miR-141-3p | GJC1 | NM_005497 |
| hsa-miR-141-3p | GLCCI1 | NM_138426 |
| hsa-miR-141-3p | GLRX | NM_002064 |
| hsa-miR-141-3p | GLS | NM_014905 |
| hsa-miR-141-3p | GNG7 | NM_052847 |
| hsa-miR-141-3p | GNL1 | NM_005275 |
| hsa-miR-141-3p | GPR137C | NM_001099652 |
| hsa-miR-141-3p | GPR6 | NM_005284 |
| hsa-miR-141-3p | GRIN2D | NM_000836 |
| hsa-miR-141-3p | HCAR2 | NM_177551 |
| hsa-miR-141-3p | HCAR3 | NM_006018 |
| hsa-miR-141-3p | HCN1 | NM_021072 |
| hsa-miR-141-3p | HGF | NM_000601 |
| hsa-miR-141-3p | HGF | NM_001010932 |
| hsa-miR-141-3p | HMG20A | NM_018200 |
| hsa-miR-141-3p | HS2ST1 | NM_012262 |
| hsa-miR-141-3p | HSPA13 | NM_006948 |
| hsa-miR-141-3p | IFNAR1 | NM_000629 |
| hsa-miR-141-3p | IGF2BP2 | NM_001007225 |
| hsa-miR-141-3p | IGF2BP2 | NM_006548 |
| hsa-miR-141-3p | IRS2 | NM_003749 |
| hsa-miR-141-3p | KATNAL1 | NM_001014380 |
| hsa-miR-141-3p | KATNAL1 | NM_032116 |
| hsa-miR-141-3p | KCNJ2 | NM_000891 |
| hsa-miR-141-3p | KCTD20 | NM_173562 |
| hsa-miR-141-3p | KIF17 | NM_001122819 |
| hsa-miR-141-3p | KIF17 | NM_020816 |
| hsa-miR-141-3p | KIF3A | NM_007054 |
| hsa-miR-141-3p | LAMTOR3 | NM_021970 |
| hsa-miR-141-3p | LMO3 | NM_001001395 |
| hsa-miR-141-3p | LMO3 | NM_018640 |
| hsa-miR-141-3p | LRRTM2 | NM_015564 |
| hsa-miR-141-3p | LSAMP | NM_002338 |
| hsa-miR-141-3p | MACC1 | NM_182762 |
| hsa-miR-141-3p | MAP2K4 | NM_003010 |
| hsa-miR-141-3p | MAP3K2 | NM_006609 |
| hsa-miR-141-3p | MAP7D3 | NM_001173516 |
| hsa-miR-141-3p | MAP7D3 | NM_024597 |
| hsa-miR-141-3p | 43531 | NM_022826 |
| hsa-miR-141-3p | MBTPS2 | NM_015884 |
| hsa-miR-141-3p | MIER1 | NM_001077702 |
| hsa-miR-141-3p | MIER1 | NM_001077703 |
| hsa-miR-141-3p | MIER1 | NM_001077704 |
| hsa-miR-141-3p | MIER1 | NM_001146111 |
| hsa-miR-141-3p | MIER1 | NM_001146113 |
| hsa-miR-141-3p | MMP24 | NM_006690 |
| hsa-miR-141-3p | MPPED2 | NM_001584 |
| hsa-miR-141-3p | MTF2 | NM_001164391 |
| hsa-miR-141-3p | MTF2 | NM_001164392 |
| hsa-miR-141-3p | MTF2 | NM_001164393 |
| hsa-miR-141-3p | MTF2 | NM_007358 |
| hsa-miR-141-3p | MYH10 | NM_005964 |
| hsa-miR-141-3p | NDFIP2 | NM_001161407 |
| hsa-miR-141-3p | NDFIP2 | NM_019080 |
| hsa-miR-141-3p | NEK6 | NM_001145001 |
| hsa-miR-141-3p | NEK6 | NM_001166167 |
| hsa-miR-141-3p | NEK6 | NM_001166168 |
| hsa-miR-141-3p | NEK6 | NM_001166169 |
| hsa-miR-141-3p | NEK6 | NM_001166170 |
| hsa-miR-141-3p | NEK6 | NM_001166171 |
| hsa-miR-141-3p | NEK6 | NM_014397 |
| hsa-miR-141-3p | NME1 | NM_000269 |
| hsa-miR-141-3p | NME1 | NM_198175 |
| hsa-miR-141-3p | NR2C2 | NM_003298 |
| hsa-miR-141-3p | NRCAM | NM_001037132 |
| hsa-miR-141-3p | NRCAM | NM_001193582 |
| hsa-miR-141-3p | NRCAM | NM_001193583 |
| hsa-miR-141-3p | NRCAM | NM_001193584 |
| hsa-miR-141-3p | NRCAM | NM_005010 |
| hsa-miR-141-3p | NRIP3 | NM_020645 |
| hsa-miR-141-3p | NRP1 | NM_003873 |
| hsa-miR-141-3p | NUFIP2 | NM_020772 |
| hsa-miR-141-3p | OXSR1 | NM_005109 |
| hsa-miR-141-3p | PALM2-AKAP2 | NM_007203 |
| hsa-miR-141-3p | PALM2-AKAP2 | NM_147150 |
| hsa-miR-141-3p | PANK3 | NM_024594 |
| hsa-miR-141-3p | PAQR9 | NM_198504 |
| hsa-miR-141-3p | PAX3 | NM_001127366 |
| hsa-miR-141-3p | PAX3 | NM_181458 |
| hsa-miR-141-3p | PAX3 | NM_181459 |
| hsa-miR-141-3p | PAX3 | NM_181460 |
| hsa-miR-141-3p | PAX3 | NM_181461 |
| hsa-miR-141-3p | PCGF3 | NM_006315 |
| hsa-miR-141-3p | PDCD4 | NM_001199492 |
| hsa-miR-141-3p | PDCD4 | NM_014456 |
| hsa-miR-141-3p | PDCD4 | NM_145341 |
| hsa-miR-141-3p | PEG3 | NM_001146184 |
| hsa-miR-141-3p | PEG3 | NM_001146185 |
| hsa-miR-141-3p | PEG3 | NM_001146186 |
| hsa-miR-141-3p | PEG3 | NM_001146187 |
| hsa-miR-141-3p | PEG3 | NM_006210 |
| hsa-miR-141-3p | PHYHIPL | NM_001143774 |
| hsa-miR-141-3p | PHYHIPL | NM_032439 |
| hsa-miR-141-3p | PIGW | NM_178517 |
| hsa-miR-141-3p | PIKFYVE | NM_015040 |
| hsa-miR-141-3p | PITPNB | NM_012399 |
| hsa-miR-141-3p | PLAG1 | NM_001114634 |
| hsa-miR-141-3p | PLAG1 | NM_001114635 |
| hsa-miR-141-3p | PLAG1 | NM_002655 |
| hsa-miR-141-3p | PLCB4 | NM_000933 |
| hsa-miR-141-3p | PLCB4 | NM_001172646 |
| hsa-miR-141-3p | PLCB4 | NM_182797 |
| hsa-miR-141-3p | PLEK | NM_002664 |
| hsa-miR-141-3p | PLEKHA8 | NM_001197026 |
| hsa-miR-141-3p | PLXNA4 | NM_020911 |
| hsa-miR-141-3p | POT1 | NM_001042594 |
| hsa-miR-141-3p | POT1 | NM_015450 |
| hsa-miR-141-3p | PPARA | NM_001001928 |
| hsa-miR-141-3p | PPARA | NM_005036 |
| hsa-miR-141-3p | PPM1A | NM_177951 |
| hsa-miR-141-3p | PPM1E | NM_014906 |
| hsa-miR-141-3p | PPT2 | NM_001204103 |
| hsa-miR-141-3p | PPT2 | NM_005155 |
| hsa-miR-141-3p | PPT2 | NM_138717 |
| hsa-miR-141-3p | PRKACB | NM_001242857 |
| hsa-miR-141-3p | PRKACB | NM_001242858 |
| hsa-miR-141-3p | PRKACB | NM_001242859 |
| hsa-miR-141-3p | PRKACB | NM_001242860 |
| hsa-miR-141-3p | PRKACB | NM_001242861 |
| hsa-miR-141-3p | PRKACB | NM_001242862 |
| hsa-miR-141-3p | PRKACB | NM_002731 |
| hsa-miR-141-3p | PRKACB | NM_182948 |
| hsa-miR-141-3p | PRKAR1A | NM_002734 |
| hsa-miR-141-3p | PRKAR1A | NM_212471 |
| hsa-miR-141-3p | PRKAR1A | NM_212472 |
| hsa-miR-141-3p | PRKCE | NM_005400 |
| hsa-miR-141-3p | QSER1 | NM_001076786 |
| hsa-miR-141-3p | RAB8B | NM_016530 |
| hsa-miR-141-3p | RABGAP1 | NM_012197 |
| hsa-miR-141-3p | RALGPS2 | NM_152663 |
| hsa-miR-141-3p | RANBP6 | NM_012416 |
| hsa-miR-141-3p | RAPGEF5 | NM_012294 |
| hsa-miR-141-3p | RASSF8 | NM_001164746 |
| hsa-miR-141-3p | RASSF8 | NM_001164747 |
| hsa-miR-141-3p | RASSF8 | NM_001164748 |
| hsa-miR-141-3p | RBM24 | NM_001143941 |
| hsa-miR-141-3p | RBM24 | NM_001143942 |
| hsa-miR-141-3p | RBM24 | NM_153020 |
| hsa-miR-141-3p | RBM33 | NM_053043 |
| hsa-miR-141-3p | RCOR3 | NM_001136225 |
| hsa-miR-141-3p | RFTN1 | NM_015150 |
| hsa-miR-141-3p | RFX1 | NM_002918 |
| hsa-miR-141-3p | RNF145 | NM_001199380 |
| hsa-miR-141-3p | RNF145 | NM_001199381 |
| hsa-miR-141-3p | RNF145 | NM_001199382 |
| hsa-miR-141-3p | RNF145 | NM_001199383 |
| hsa-miR-141-3p | RNF145 | NM_144726 |
| hsa-miR-141-3p | RNF185 | NM_001135825 |
| hsa-miR-141-3p | RNF185 | NM_152267 |
| hsa-miR-141-3p | RUNX1 | NM_001001890 |
| hsa-miR-141-3p | RUNX1 | NM_001754 |
| hsa-miR-141-3p | S100PBP | NM_022753 |
| hsa-miR-141-3p | SAMD8 | NM_001174156 |
| hsa-miR-141-3p | SAMD8 | NM_144660 |
| hsa-miR-141-3p | 43716 | NM_001098812 |
| hsa-miR-141-3p | 43716 | NM_001098813 |
| hsa-miR-141-3p | 43716 | NM_015146 |
| hsa-miR-141-3p | SIAH1 | NM_001006610 |
| hsa-miR-141-3p | SIAH1 | NM_003031 |
| hsa-miR-141-3p | SIK1 | NM_173354 |
| hsa-miR-141-3p | SLC16A7 | NM_004731 |
| hsa-miR-141-3p | SLC25A3 | NM_002635 |
| hsa-miR-141-3p | SLC25A3 | NM_005888 |
| hsa-miR-141-3p | SLC25A3 | NM_213611 |
| hsa-miR-141-3p | SLC35D1 | NM_015139 |
| hsa-miR-141-3p | SLC5A3 | NM_006933 |
| hsa-miR-141-3p | SNX18 | NM_001102575 |
| hsa-miR-141-3p | SOX11 | NM_003108 |
| hsa-miR-141-3p | SOX17 | NM_022454 |
| hsa-miR-141-3p | SOX5 | NM_006940 |
| hsa-miR-141-3p | SOX5 | NM_152989 |
| hsa-miR-141-3p | SOX5 | NM_178010 |
| hsa-miR-141-3p | SPAG9 | NM_001130528 |
| hsa-miR-141-3p | SPAG9 | NM_003971 |
| hsa-miR-141-3p | SRCAP | NM_006662 |
| hsa-miR-141-3p | ST3GAL3 | NM_006279 |
| hsa-miR-141-3p | ST3GAL3 | NM_174963 |
| hsa-miR-141-3p | ST3GAL3 | NM_174964 |
| hsa-miR-141-3p | ST3GAL3 | NM_174965 |
| hsa-miR-141-3p | ST3GAL3 | NM_174966 |
| hsa-miR-141-3p | ST3GAL3 | NM_174967 |
| hsa-miR-141-3p | ST3GAL3 | NM_174968 |
| hsa-miR-141-3p | ST3GAL3 | NM_174969 |
| hsa-miR-141-3p | ST3GAL3 | NM_174970 |
| hsa-miR-141-3p | ST3GAL3 | NM_174971 |
| hsa-miR-141-3p | ST3GAL5 | NM_001042437 |
| hsa-miR-141-3p | ST3GAL5 | NM_003896 |
| hsa-miR-141-3p | STAT4 | NM_003151 |
| hsa-miR-141-3p | STRN | NM_003162 |
| hsa-miR-141-3p | STX16 | NM_001001433 |
| hsa-miR-141-3p | STX16 | NM_001134772 |
| hsa-miR-141-3p | STX16 | NM_001134773 |
| hsa-miR-141-3p | STX16 | NM_001204868 |
| hsa-miR-141-3p | STX16 | NM_003763 |
| hsa-miR-141-3p | STXBP5 | NM_001127715 |
| hsa-miR-141-3p | STXBP5 | NM_139244 |
| hsa-miR-141-3p | SUPT6H | NM_003170 |
| hsa-miR-141-3p | SYN3 | NM_001135774 |
| hsa-miR-141-3p | SYN3 | NM_003490 |
| hsa-miR-141-3p | SYN3 | NM_133633 |
| hsa-miR-141-3p | TADA1 | NM_053053 |
| hsa-miR-141-3p | TCERG1 | NM_001040006 |
| hsa-miR-141-3p | TCERG1 | NM_006706 |
| hsa-miR-141-3p | TET1 | NM_030625 |
| hsa-miR-141-3p | TM4SF1 | NM_014220 |
| hsa-miR-141-3p | TMEM110 | NM_198563 |
| hsa-miR-141-3p | TMEM170B | NM_001100829 |
| hsa-miR-141-3p | TMEM56 | NM_001199679 |
| hsa-miR-141-3p | TMEM56 | NM_152487 |
| hsa-miR-141-3p | TNKS2 | NM_025235 |
| hsa-miR-141-3p | TNS1 | NM_022648 |
| hsa-miR-141-3p | TP53INP1 | NM_001135733 |
| hsa-miR-141-3p | TP53INP1 | NM_033285 |
| hsa-miR-141-3p | TPH2 | NM_173353 |
| hsa-miR-141-3p | TSHZ3 | NM_020856 |
| hsa-miR-141-3p | TTR | NM_000371 |
| hsa-miR-141-3p | U2SURP | NM_001080415 |
| hsa-miR-141-3p | UACA | NM_001008224 |
| hsa-miR-141-3p | UACA | NM_018003 |
| hsa-miR-141-3p | VSIG8 | NM_001013661 |
| hsa-miR-141-3p | WAPAL | NM_015045 |
| hsa-miR-141-3p | WDR31 | NM_001012361 |
| hsa-miR-141-3p | WDR31 | NM_145241 |
| hsa-miR-141-3p | WDR43 | NM_015131 |
| hsa-miR-141-3p | WDR81 | NM_001163673 |
| hsa-miR-141-3p | WDR81 | NM_001163809 |
| hsa-miR-141-3p | WDR81 | NM_001163811 |
| hsa-miR-141-3p | WDR81 | NM_152348 |
| hsa-miR-141-3p | WWTR1 | NM_001168278 |
| hsa-miR-141-3p | WWTR1 | NM_001168280 |
| hsa-miR-141-3p | WWTR1 | NM_015472 |
| hsa-miR-141-3p | YAP1 | NM_001130145 |
| hsa-miR-141-3p | YAP1 | NM_001195044 |
| hsa-miR-141-3p | YAP1 | NM_001195045 |
| hsa-miR-141-3p | YAP1 | NM_006106 |
| hsa-miR-141-3p | YWHAG | NM_012479 |
| hsa-miR-141-3p | ZDHHC21 | NM_178566 |
| hsa-miR-141-3p | ZEB1 | NM_001128128 |
| hsa-miR-141-3p | ZEB1 | NM_001174093 |
| hsa-miR-141-3p | ZEB1 | NM_001174094 |
| hsa-miR-141-3p | ZEB1 | NM_001174095 |
| hsa-miR-141-3p | ZEB1 | NM_001174096 |
| hsa-miR-141-3p | ZEB1 | NM_030751 |
| hsa-miR-141-3p | ZEB2 | NM_001171653 |
| hsa-miR-141-3p | ZEB2 | NM_014795 |
| hsa-miR-141-3p | ZNF629 | NM_001080417 |
| hsa-miR-141-3p | ZNF660 | NM_173658 |
| hsa-miR-146a-5p | ALX4 | NM_021926 |
| hsa-miR-146a-5p | ATG12 | NM_004707 |
| hsa-miR-146a-5p | BCORL1 | NM_021946 |
| hsa-miR-146a-5p | C4orf3 | NM_001001701 |
| hsa-miR-146a-5p | C4orf3 | NM_001170330 |
| hsa-miR-146a-5p | CDS1 | NM_001263 |
| hsa-miR-146a-5p | DCAF12 | NM_015397 |
| hsa-miR-146a-5p | DCDC5 | NM_020869 |
| hsa-miR-146a-5p | EARS2 | NM_001083614 |
| hsa-miR-146a-5p | ERBB4 | NM_001042599 |
| hsa-miR-146a-5p | ERBB4 | NM_005235 |
| hsa-miR-146a-5p | FAM26E | NM_153711 |
| hsa-miR-146a-5p | FBXO28 | NM_015176 |
| hsa-miR-146a-5p | GALNT10 | NM_198321 |
| hsa-miR-146a-5p | IGSF1 | NM_001170963 |
| hsa-miR-146a-5p | IGSF1 | NM_205833 |
| hsa-miR-146a-5p | IRAK1 | NM_001025242 |
| hsa-miR-146a-5p | IRAK1 | NM_001025243 |
| hsa-miR-146a-5p | IRAK1 | NM_001569 |
| hsa-miR-146a-5p | KCTD15 | NM_001129994 |
| hsa-miR-146a-5p | KCTD15 | NM_001129995 |
| hsa-miR-146a-5p | KLF7 | NM_003709 |
| hsa-miR-146a-5p | LRRC15 | NM_001135057 |
| hsa-miR-146a-5p | LRRC15 | NM_130830 |
| hsa-miR-146a-5p | LRTOMT | NM_001145308 |
| hsa-miR-146a-5p | MICU1 | NM_001195519 |
| hsa-miR-146a-5p | MICU1 | NM_006077 |
| hsa-miR-146a-5p | NAIF1 | NM_197956 |
| hsa-miR-146a-5p | NF2 | NM_000268 |
| hsa-miR-146a-5p | NF2 | NM_016418 |
| hsa-miR-146a-5p | NF2 | NM_181828 |
| hsa-miR-146a-5p | NF2 | NM_181829 |
| hsa-miR-146a-5p | NF2 | NM_181830 |
| hsa-miR-146a-5p | NF2 | NM_181832 |
| hsa-miR-146a-5p | NF2 | NM_181833 |
| hsa-miR-146a-5p | PCDH1 | NM_002587 |
| hsa-miR-146a-5p | PPP1R11 | NM_021959 |
| hsa-miR-146a-5p | PRKCE | NM_005400 |
| hsa-miR-146a-5p | RABGAP1 | NM_012197 |
| hsa-miR-146a-5p | RNASEL | NM_021133 |
| hsa-miR-146a-5p | SIAH2 | NM_005067 |
| hsa-miR-146a-5p | SLC2A3 | NM_006931 |
| hsa-miR-146a-5p | SLC38A1 | NM_001077484 |
| hsa-miR-146a-5p | SLC38A1 | NM_030674 |
| hsa-miR-146a-5p | SNX21 | NM_001042632 |
| hsa-miR-146a-5p | SNX21 | NM_001042633 |
| hsa-miR-146a-5p | SNX21 | NM_033421 |
| hsa-miR-146a-5p | SNX21 | NM_152897 |
| hsa-miR-146a-5p | SORT1 | NM_001205228 |
| hsa-miR-146a-5p | SORT1 | NM_002959 |
| hsa-miR-146a-5p | STRBP | NM_001171137 |
| hsa-miR-146a-5p | STRBP | NM_018387 |
| hsa-miR-146a-5p | TANC2 | NM_025185 |
| hsa-miR-146a-5p | TDRKH | NM_001083963 |
| hsa-miR-146a-5p | TDRKH | NM_001083964 |
| hsa-miR-146a-5p | TDRKH | NM_001083965 |
| hsa-miR-146a-5p | TMEM194A | NM_001130963 |
| hsa-miR-146a-5p | TMEM194A | NM_015257 |
| hsa-miR-146a-5p | TRAF6 | NM_004620 |
| hsa-miR-146a-5p | TRAF6 | NM_145803 |
| hsa-miR-146a-5p | UHRF1 | NM_001048201 |
| hsa-miR-146a-5p | UHRF1 | NM_013282 |
| hsa-miR-146a-5p | USP3 | NM_006537 |
| hsa-miR-146a-5p | WWC2 | NM_024949 |
| hsa-miR-146a-5p | ZBTB2 | NM_020861 |
| hsa-miR-146a-5p | ZDHHC7 | NM_001145548 |
| hsa-miR-146a-5p | ZDHHC7 | NM_017740 |
| hsa-miR-146a-5p | ZNF367 | NM_153695 |
| hsa-miR-146a-5p | ZNF512B | NM_020713 |
| hsa-miR-146a-5p | ZNF532 | NM_018181 |
| hsa-miR-146a-5p | ZNF652 | NM_001145365 |
| hsa-miR-146a-5p | ZNF652 | NM_014897 |
| hsa-miR-146a-5p | ZNRF3 | NM_001206998 |
| hsa-miR-146a-5p | ZNRF3 | NM_032173 |
| hsa-miR-155-5p | AAK1 | NM_014911 |
| hsa-miR-155-5p | ASTN1 | NM_004319 |
| hsa-miR-155-5p | ATP6V1G1 | NM_004888 |
| hsa-miR-155-5p | BNC2 | NM_017637 |
| hsa-miR-155-5p | C8orf44-SGK3 | NM_001204173 |
| hsa-miR-155-5p | CARHSP1 | NM_001042476 |
| hsa-miR-155-5p | CARHSP1 | NM_014316 |
| hsa-miR-155-5p | CEBPB | NM_005194 |
| hsa-miR-155-5p | CLCN5 | NM_000084 |
| hsa-miR-155-5p | CLCN5 | NM_001127898 |
| hsa-miR-155-5p | CLCN5 | NM_001127899 |
| hsa-miR-155-5p | COL21A1 | NM_030820 |
| hsa-miR-155-5p | CSRNP2 | NM_030809 |
| hsa-miR-155-5p | DET1 | NM_001144074 |
| hsa-miR-155-5p | DET1 | NM_017996 |
| hsa-miR-155-5p | DHX40 | NM_001166301 |
| hsa-miR-155-5p | DHX40 | NM_024612 |
| hsa-miR-155-5p | FAM104A | NM_001098832 |
| hsa-miR-155-5p | FAM104A | NM_032837 |
| hsa-miR-155-5p | FBXO22 | NM_147188 |
| hsa-miR-155-5p | FGF7 | NM_002009 |
| hsa-miR-155-5p | FOS | NM_005252 |
| hsa-miR-155-5p | FXR1 | NM_001013438 |
| hsa-miR-155-5p | FXR1 | NM_001013439 |
| hsa-miR-155-5p | FXR1 | NM_005087 |
| hsa-miR-155-5p | GCNT1 | NM_001097633 |
| hsa-miR-155-5p | GCNT1 | NM_001097634 |
| hsa-miR-155-5p | GCNT1 | NM_001097635 |
| hsa-miR-155-5p | GCNT1 | NM_001097636 |
| hsa-miR-155-5p | GCNT1 | NM_001490 |
| hsa-miR-155-5p | IRF2BP2 | NM_001077397 |
| hsa-miR-155-5p | IRF2BP2 | NM_182972 |
| hsa-miR-155-5p | JARID2 | NM_004973 |
| hsa-miR-155-5p | KDM5B | NM_006618 |
| hsa-miR-155-5p | KIAA1715 | NM_030650 |
| hsa-miR-155-5p | LCA5 | NM_001122769 |
| hsa-miR-155-5p | LCA5 | NM_181714 |
| hsa-miR-155-5p | MAP3K14 | NM_003954 |
| hsa-miR-155-5p | MYLK | NM_053025 |
| hsa-miR-155-5p | MYLK | NM_053026 |
| hsa-miR-155-5p | MYLK | NM_053027 |
| hsa-miR-155-5p | MYLK | NM_053028 |
| hsa-miR-155-5p | MYLK | NM_053031 |
| hsa-miR-155-5p | MYLK | NM_053032 |
| hsa-miR-155-5p | MYO10 | NM_012334 |
| hsa-miR-155-5p | NFIA | NM_001134673 |
| hsa-miR-155-5p | NFIA | NM_001145511 |
| hsa-miR-155-5p | NFIA | NM_001145512 |
| hsa-miR-155-5p | NFIA | NM_005595 |
| hsa-miR-155-5p | NOVA1 | NM_002515 |
| hsa-miR-155-5p | NOVA1 | NM_006489 |
| hsa-miR-155-5p | PEG10 | NM_015068 |
| hsa-miR-155-5p | PHC2 | NM_004427 |
| hsa-miR-155-5p | PHC2 | NM_198040 |
| hsa-miR-155-5p | PHC3 | NM_024947 |
| hsa-miR-155-5p | PLD5 | NM_001195811 |
| hsa-miR-155-5p | PLD5 | NM_001195812 |
| hsa-miR-155-5p | PLD5 | NM_152666 |
| hsa-miR-155-5p | RAPH1 | NM_213589 |
| hsa-miR-155-5p | RNF149 | NM_173647 |
| hsa-miR-155-5p | S100PBP | NM_022753 |
| hsa-miR-155-5p | SEMA5A | NM_003966 |
| hsa-miR-155-5p | SGK3 | NM_001033578 |
| hsa-miR-155-5p | SGK3 | NM_013257 |
| hsa-miR-155-5p | SGK3 | NM_170709 |
| hsa-miR-155-5p | SLC11A2 | NM_001174126 |
| hsa-miR-155-5p | SLC11A2 | NM_001174127 |
| hsa-miR-155-5p | SLC12A6 | NM_001042494 |
| hsa-miR-155-5p | SLC12A6 | NM_001042495 |
| hsa-miR-155-5p | SLC12A6 | NM_001042496 |
| hsa-miR-155-5p | SLC12A6 | NM_001042497 |
| hsa-miR-155-5p | SLC12A6 | NM_005135 |
| hsa-miR-155-5p | SLC12A6 | NM_133647 |
| hsa-miR-155-5p | SLC33A1 | NM_004733 |
| hsa-miR-155-5p | SPI1 | NM_001080547 |
| hsa-miR-155-5p | SPI1 | NM_003120 |
| hsa-miR-155-5p | TBRG1 | NM_032811 |
| hsa-miR-155-5p | TCEB1 | NM_001204857 |
| hsa-miR-155-5p | TCEB1 | NM_001204858 |
| hsa-miR-155-5p | TCEB1 | NM_001204859 |
| hsa-miR-155-5p | TCEB1 | NM_001204860 |
| hsa-miR-155-5p | TCEB1 | NM_001204861 |
| hsa-miR-155-5p | TCEB1 | NM_001204862 |
| hsa-miR-155-5p | TCEB1 | NM_001204863 |
| hsa-miR-155-5p | TCEB1 | NM_001204864 |
| hsa-miR-155-5p | TCEB1 | NM_005648 |
| hsa-miR-155-5p | TRIM32 | NM_001099679 |
| hsa-miR-155-5p | TRIM32 | NM_012210 |
| hsa-miR-155-5p | USP43 | NM_153210 |
| hsa-miR-155-5p | ZBTB41 | NM_194314 |
| hsa-miR-155-5p | ZMYM2 | NM_001190964 |
| hsa-miR-155-5p | ZMYM2 | NM_001190965 |
| hsa-miR-155-5p | ZMYM2 | NM_003453 |
| hsa-miR-155-5p | ZMYM2 | NM_197968 |
| hsa-miR-155-5p | ZNF236 | NM_007345 |
| hsa-miR-155-5p | ZNF618 | NM_133374 |
| hsa-miR-155-5p | ZPLD1 | NM_175056 |
| hsa-miR-17-5p | ABCA1 | NM_005502 |
| hsa-miR-17-5p | ABCG4 | NM_001142505 |
| hsa-miR-17-5p | ABCG4 | NM_022169 |
| hsa-miR-17-5p | ABHD5 | NM_016006 |
| hsa-miR-17-5p | ACPL2 | NM_001037172 |
| hsa-miR-17-5p | AGFG2 | NM_006076 |
| hsa-miR-17-5p | AKTIP | NM_001012398 |
| hsa-miR-17-5p | AKTIP | NM_022476 |
| hsa-miR-17-5p | ANKFY1 | NM_016376 |
| hsa-miR-17-5p | ANKIB1 | NM_019004 |
| hsa-miR-17-5p | ANKRD29 | NM_173505 |
| hsa-miR-17-5p | ANKRD50 | NM_001167882 |
| hsa-miR-17-5p | ANKRD50 | NM_020337 |
| hsa-miR-17-5p | ANKRD52 | NM_173595 |
| hsa-miR-17-5p | ANKRD9 | NM_152326 |
| hsa-miR-17-5p | ANO6 | NM_001025356 |
| hsa-miR-17-5p | ANO6 | NM_001142678 |
| hsa-miR-17-5p | ANO6 | NM_001204803 |
| hsa-miR-17-5p | APBB2 | NM_001166050 |
| hsa-miR-17-5p | APBB2 | NM_001166052 |
| hsa-miR-17-5p | APBB2 | NM_001166054 |
| hsa-miR-17-5p | APBB2 | NM_004307 |
| hsa-miR-17-5p | APBB2 | NM_173075 |
| hsa-miR-17-5p | APP | NM_000484 |
| hsa-miR-17-5p | APP | NM_001136016 |
| hsa-miR-17-5p | APP | NM_001136129 |
| hsa-miR-17-5p | APP | NM_001136130 |
| hsa-miR-17-5p | APP | NM_001136131 |
| hsa-miR-17-5p | APP | NM_001204301 |
| hsa-miR-17-5p | APP | NM_001204302 |
| hsa-miR-17-5p | APP | NM_001204303 |
| hsa-miR-17-5p | APP | NM_201413 |
| hsa-miR-17-5p | APP | NM_201414 |
| hsa-miR-17-5p | ARHGAP1 | NM_004308 |
| hsa-miR-17-5p | ARHGAP12 | NM_018287 |
| hsa-miR-17-5p | ARHGAP26 | NM_001135608 |
| hsa-miR-17-5p | ARHGAP26 | NM_015071 |
| hsa-miR-17-5p | ARHGEF11 | NM_014784 |
| hsa-miR-17-5p | ARHGEF11 | NM_198236 |
| hsa-miR-17-5p | ARHGEF18 | NM_001130955 |
| hsa-miR-17-5p | ARHGEF18 | NM_015318 |
| hsa-miR-17-5p | ARHGEF7 | NM_001113513 |
| hsa-miR-17-5p | ARHGEF7 | NM_003899 |
| hsa-miR-17-5p | ARID4B | NM_001206794 |
| hsa-miR-17-5p | ARID4B | NM_016374 |
| hsa-miR-17-5p | ARID4B | NM_031371 |
| hsa-miR-17-5p | ARL1 | NM_001177 |
| hsa-miR-17-5p | ARL4C | NM_005737 |
| hsa-miR-17-5p | ASF1A | NM_014034 |
| hsa-miR-17-5p | ATAD2 | NM_014109 |
| hsa-miR-17-5p | ATG14 | NM_014924 |
| hsa-miR-17-5p | ATG2B | NM_018036 |
| hsa-miR-17-5p | ATL3 | NM_015459 |
| hsa-miR-17-5p | ATP1A2 | NM_000702 |
| hsa-miR-17-5p | BAMBI | NM_012342 |
| hsa-miR-17-5p | BMPR2 | NM_001204 |
| hsa-miR-17-5p | BNIP2 | NM_004330 |
| hsa-miR-17-5p | BNIP3L | NM_004331 |
| hsa-miR-17-5p | BRWD1 | NM_001007246 |
| hsa-miR-17-5p | BTBD7 | NM_001002860 |
| hsa-miR-17-5p | BVES | NM_001199563 |
| hsa-miR-17-5p | BVES | NM_007073 |
| hsa-miR-17-5p | BVES | NM_147147 |
| hsa-miR-17-5p | C16orf52 | NM_001164579 |
| hsa-miR-17-5p | C7orf43 | NM_018275 |
| hsa-miR-17-5p | C9orf40 | NM_017998 |
| hsa-miR-17-5p | CAMK2N2 | NM_033259 |
| hsa-miR-17-5p | CAPRIN2 | NM_001002259 |
| hsa-miR-17-5p | CAPRIN2 | NM_001206856 |
| hsa-miR-17-5p | CAPRIN2 | NM_023925 |
| hsa-miR-17-5p | CAPRIN2 | NM_032156 |
| hsa-miR-17-5p | CC2D1A | NM_017721 |
| hsa-miR-17-5p | CCNG2 | NM_004354 |
| hsa-miR-17-5p | CD274 | NM_014143 |
| hsa-miR-17-5p | CDC40 | NM_015891 |
| hsa-miR-17-5p | CENPQ | NM_018132 |
| hsa-miR-17-5p | CEP128 | NM_152446 |
| hsa-miR-17-5p | CEP57 | NM_014679 |
| hsa-miR-17-5p | CFL2 | NM_021914 |
| hsa-miR-17-5p | CFL2 | NM_138638 |
| hsa-miR-17-5p | CHAF1A | NM_005483 |
| hsa-miR-17-5p | CHD5 | NM_015557 |
| hsa-miR-17-5p | CHD9 | NM_025134 |
| hsa-miR-17-5p | CIT | NM_001206999 |
| hsa-miR-17-5p | CIT | NM_007174 |
| hsa-miR-17-5p | CNGB3 | NM_019098 |
| hsa-miR-17-5p | CNN1 | NM_001299 |
| hsa-miR-17-5p | CNOT4 | NM_001008225 |
| hsa-miR-17-5p | CNOT4 | NM_001190848 |
| hsa-miR-17-5p | CNRIP1 | NM_001111101 |
| hsa-miR-17-5p | COQ2 | NM_015697 |
| hsa-miR-17-5p | CORO2B | NM_001190456 |
| hsa-miR-17-5p | CORO2B | NM_001190457 |
| hsa-miR-17-5p | CORO2B | NM_006091 |
| hsa-miR-17-5p | COX7A2L | NM_004718 |
| hsa-miR-17-5p | CPEB3 | NM_001178137 |
| hsa-miR-17-5p | CPEB3 | NM_014912 |
| hsa-miR-17-5p | CRIPT | NM_014171 |
| hsa-miR-17-5p | CRK | NM_005206 |
| hsa-miR-17-5p | CRK | NM_016823 |
| hsa-miR-17-5p | CROT | NM_001143935 |
| hsa-miR-17-5p | CROT | NM_021151 |
| hsa-miR-17-5p | CSNK1G1 | NM_022048 |
| hsa-miR-17-5p | CTSA | NM_000308 |
| hsa-miR-17-5p | CTSA | NM_001127695 |
| hsa-miR-17-5p | CTSA | NM_001167594 |
| hsa-miR-17-5p | CYBB | NM_000397 |
| hsa-miR-17-5p | CYBRD1 | NM_001127383 |
| hsa-miR-17-5p | CYBRD1 | NM_024843 |
| hsa-miR-17-5p | DCAF8 | NM_015726 |
| hsa-miR-17-5p | DCBLD2 | NM_080927 |
| hsa-miR-17-5p | DCUN1D3 | NM_173475 |
| hsa-miR-17-5p | DDHD1 | NM_001160147 |
| hsa-miR-17-5p | DDHD1 | NM_001160148 |
| hsa-miR-17-5p | DDHD1 | NM_030637 |
| hsa-miR-17-5p | DEDD | NM_001039711 |
| hsa-miR-17-5p | DEDD | NM_001039712 |
| hsa-miR-17-5p | DEDD | NM_032998 |
| hsa-miR-17-5p | DENND5B | NM_144973 |
| hsa-miR-17-5p | DIP2A | NM_001146116 |
| hsa-miR-17-5p | DIP2A | NM_015151 |
| hsa-miR-17-5p | DMRTA2 | NM_032110 |
| hsa-miR-17-5p | DMTF1 | NM_001142326 |
| hsa-miR-17-5p | DMTF1 | NM_001142327 |
| hsa-miR-17-5p | DMTF1 | NM_021145 |
| hsa-miR-17-5p | DNAJB6 | NM_005494 |
| hsa-miR-17-5p | DOCK4 | NM_014705 |
| hsa-miR-17-5p | DPF3 | NM_012074 |
| hsa-miR-17-5p | DPYSL5 | NM_020134 |
| hsa-miR-17-5p | DYNC1LI2 | NM_006141 |
| hsa-miR-17-5p | E2F1 | NM_005225 |
| hsa-miR-17-5p | E2F5 | NM_001083588 |
| hsa-miR-17-5p | E2F5 | NM_001083589 |
| hsa-miR-17-5p | E2F5 | NM_001951 |
| hsa-miR-17-5p | EIF2S1 | NM_004094 |
| hsa-miR-17-5p | EIF4G2 | NM_001042559 |
| hsa-miR-17-5p | EIF4G2 | NM_001172705 |
| hsa-miR-17-5p | EIF4G2 | NM_001418 |
| hsa-miR-17-5p | EIF5A2 | NM_020390 |
| hsa-miR-17-5p | ELK4 | NM_001973 |
| hsa-miR-17-5p | ENPP5 | NM_021572 |
| hsa-miR-17-5p | ENTPD4 | NM_001128930 |
| hsa-miR-17-5p | ENTPD4 | NM_004901 |
| hsa-miR-17-5p | EPB41L5 | NM_001184937 |
| hsa-miR-17-5p | EPB41L5 | NM_020909 |
| hsa-miR-17-5p | EPHA4 | NM_004438 |
| hsa-miR-17-5p | ERBB3 | NM_001982 |
| hsa-miR-17-5p | EREG | NM_001432 |
| hsa-miR-17-5p | ETV1 | NM_001163147 |
| hsa-miR-17-5p | ETV1 | NM_001163148 |
| hsa-miR-17-5p | ETV1 | NM_001163149 |
| hsa-miR-17-5p | ETV1 | NM_001163150 |
| hsa-miR-17-5p | ETV1 | NM_001163151 |
| hsa-miR-17-5p | ETV1 | NM_001163152 |
| hsa-miR-17-5p | ETV1 | NM_004956 |
| hsa-miR-17-5p | EXPH5 | NM_015065 |
| hsa-miR-17-5p | FAM117A | NM_030802 |
| hsa-miR-17-5p | FAM129A | NM_052966 |
| hsa-miR-17-5p | FAM13A | NM_001015045 |
| hsa-miR-17-5p | FAM13A | NM_014883 |
| hsa-miR-17-5p | FAM45A | NM_207009 |
| hsa-miR-17-5p | FAM57A | NM_024792 |
| hsa-miR-17-5p | FAM60A | NM_001135811 |
| hsa-miR-17-5p | FAM60A | NM_001135812 |
| hsa-miR-17-5p | FAM60A | NM_021238 |
| hsa-miR-17-5p | FBXO39 | NM_153230 |
| hsa-miR-17-5p | FBXO48 | NM_001024680 |
| hsa-miR-17-5p | FBXW11 | NM_012300 |
| hsa-miR-17-5p | FBXW11 | NM_033644 |
| hsa-miR-17-5p | FBXW11 | NM_033645 |
| hsa-miR-17-5p | FGD4 | NM_139241 |
| hsa-miR-17-5p | FGD5 | NM_152536 |
| hsa-miR-17-5p | FNDC3A | NM_001079673 |
| hsa-miR-17-5p | FNDC3A | NM_014923 |
| hsa-miR-17-5p | FNDC3B | NM_001135095 |
| hsa-miR-17-5p | FNDC3B | NM_022763 |
| hsa-miR-17-5p | FRMD4A | NM_018027 |
| hsa-miR-17-5p | FRZB | NM_001463 |
| hsa-miR-17-5p | FURIN | NM_002569 |
| hsa-miR-17-5p | FYCO1 | NM_024513 |
| hsa-miR-17-5p | FZD3 | NM_017412 |
| hsa-miR-17-5p | FZD3 | NM_145866 |
| hsa-miR-17-5p | GAB1 | NM_002039 |
| hsa-miR-17-5p | GAB1 | NM_207123 |
| hsa-miR-17-5p | GABBR1 | NM_001470 |
| hsa-miR-17-5p | GABBR1 | NM_021903 |
| hsa-miR-17-5p | GABBR1 | NM_021904 |
| hsa-miR-17-5p | GABPB1 | NM_005254 |
| hsa-miR-17-5p | GABPB1 | NM_016654 |
| hsa-miR-17-5p | GGCX | NM_000821 |
| hsa-miR-17-5p | GGCX | NM_001142269 |
| hsa-miR-17-5p | GIGYF1 | NM_022574 |
| hsa-miR-17-5p | GLIS3 | NM_001042413 |
| hsa-miR-17-5p | GLIS3 | NM_152629 |
| hsa-miR-17-5p | GMCL1 | NM_178439 |
| hsa-miR-17-5p | GNB5 | NM_006578 |
| hsa-miR-17-5p | GNB5 | NM_016194 |
| hsa-miR-17-5p | GNPDA2 | NM_138335 |
| hsa-miR-17-5p | GNS | NM_002076 |
| hsa-miR-17-5p | GOLGA1 | NM_002077 |
| hsa-miR-17-5p | GOSR1 | NM_001007024 |
| hsa-miR-17-5p | GOSR1 | NM_001007025 |
| hsa-miR-17-5p | GOSR1 | NM_004871 |
| hsa-miR-17-5p | GPR137C | NM_001099652 |
| hsa-miR-17-5p | GPR6 | NM_005284 |
| hsa-miR-17-5p | GTDC1 | NM_001006636 |
| hsa-miR-17-5p | GTDC1 | NM_001164629 |
| hsa-miR-17-5p | GTDC1 | NM_024659 |
| hsa-miR-17-5p | GUCY1A3 | NM_000856 |
| hsa-miR-17-5p | GUCY1A3 | NM_001130682 |
| hsa-miR-17-5p | GUCY1A3 | NM_001130683 |
| hsa-miR-17-5p | GUCY1A3 | NM_001130684 |
| hsa-miR-17-5p | GUCY1A3 | NM_001130685 |
| hsa-miR-17-5p | HAUS8 | NM_001011699 |
| hsa-miR-17-5p | HAUS8 | NM_033417 |
| hsa-miR-17-5p | HBP1 | NM_012257 |
| hsa-miR-17-5p | HEG1 | NM_020733 |
| hsa-miR-17-5p | HIF1A | NM_001530 |
| hsa-miR-17-5p | HIF1A | NM_181054 |
| hsa-miR-17-5p | HLF | NM_002126 |
| hsa-miR-17-5p | HMBOX1 | NM_001135726 |
| hsa-miR-17-5p | HMBOX1 | NM_024567 |
| hsa-miR-17-5p | HMGB3 | NM_005342 |
| hsa-miR-17-5p | HOXB13 | NM_006361 |
| hsa-miR-17-5p | HS3ST5 | NM_153612 |
| hsa-miR-17-5p | HSPA6 | NM_002155 |
| hsa-miR-17-5p | IL1RAP | NM_001167928 |
| hsa-miR-17-5p | IL1RAP | NM_001167929 |
| hsa-miR-17-5p | IL1RAP | NM_002182 |
| hsa-miR-17-5p | INO80 | NM_017553 |
| hsa-miR-17-5p | IPO7 | NM_006391 |
| hsa-miR-17-5p | IQSEC1 | NM_014869 |
| hsa-miR-17-5p | IQSEC2 | NM_001111125 |
| hsa-miR-17-5p | IQSEC2 | NM_015075 |
| hsa-miR-17-5p | IRAK4 | NM_001114182 |
| hsa-miR-17-5p | IRAK4 | NM_001145256 |
| hsa-miR-17-5p | IRAK4 | NM_001145257 |
| hsa-miR-17-5p | IRAK4 | NM_001145258 |
| hsa-miR-17-5p | IRAK4 | NM_016123 |
| hsa-miR-17-5p | ISM2 | NM_182509 |
| hsa-miR-17-5p | ISM2 | NM_199296 |
| hsa-miR-17-5p | ITGB8 | NM_002214 |
| hsa-miR-17-5p | ITPRIPL2 | NM_001034841 |
| hsa-miR-17-5p | JAK1 | NM_002227 |
| hsa-miR-17-5p | JRKL | NM_003772 |
| hsa-miR-17-5p | KAT2B | NM_003884 |
| hsa-miR-17-5p | KIAA1191 | NM_001079684 |
| hsa-miR-17-5p | KIAA1191 | NM_001079685 |
| hsa-miR-17-5p | KIAA1191 | NM_020444 |
| hsa-miR-17-5p | KIF23 | NM_004856 |
| hsa-miR-17-5p | KIF23 | NM_138555 |
| hsa-miR-17-5p | KIF26B | NM_018012 |
| hsa-miR-17-5p | KPNA2 | NM_002266 |
| hsa-miR-17-5p | LAPTM4A | NM_014713 |
| hsa-miR-17-5p | LCOR | NM_001170766 |
| hsa-miR-17-5p | LDLR | NM_000527 |
| hsa-miR-17-5p | LDLR | NM_001195798 |
| hsa-miR-17-5p | LDLR | NM_001195799 |
| hsa-miR-17-5p | LDLR | NM_001195800 |
| hsa-miR-17-5p | LDLR | NM_001195803 |
| hsa-miR-17-5p | LDLRAD3 | NM_174902 |
| hsa-miR-17-5p | LHX6 | NM_001242333 |
| hsa-miR-17-5p | LHX6 | NM_001242334 |
| hsa-miR-17-5p | LHX6 | NM_001242335 |
| hsa-miR-17-5p | LHX6 | NM_014368 |
| hsa-miR-17-5p | LHX6 | NM_199160 |
| hsa-miR-17-5p | LRCH1 | NM_001164213 |
| hsa-miR-17-5p | LRP8 | NM_001018054 |
| hsa-miR-17-5p | LRP8 | NM_004631 |
| hsa-miR-17-5p | LRP8 | NM_017522 |
| hsa-miR-17-5p | LRP8 | NM_033300 |
| hsa-miR-17-5p | LRRC20 | NM_018205 |
| hsa-miR-17-5p | LRRC20 | NM_018239 |
| hsa-miR-17-5p | LRRC20 | NM_207119 |
| hsa-miR-17-5p | LRRC55 | NM_001005210 |
| hsa-miR-17-5p | LYSMD3 | NM_198273 |
| hsa-miR-17-5p | MAP3K2 | NM_006609 |
| hsa-miR-17-5p | MAP3K8 | NM_005204 |
| hsa-miR-17-5p | MAP3K9 | NM_033141 |
| hsa-miR-17-5p | MAPK9 | NM_002752 |
| hsa-miR-17-5p | MAPK9 | NM_139070 |
| hsa-miR-17-5p | MAPRE1 | NM_012325 |
| hsa-miR-17-5p | MBD2 | NM_003927 |
| hsa-miR-17-5p | MED12L | NM_053002 |
| hsa-miR-17-5p | METAP1 | NM_015143 |
| hsa-miR-17-5p | MICAL3 | NM_015241 |
| hsa-miR-17-5p | MKRN1 | NM_013446 |
| hsa-miR-17-5p | MMP2 | NM_001127891 |
| hsa-miR-17-5p | MMP2 | NM_004530 |
| hsa-miR-17-5p | MMP24 | NM_006690 |
| hsa-miR-17-5p | MYCN | NM_005378 |
| hsa-miR-17-5p | MYNN | NM_001185118 |
| hsa-miR-17-5p | MYNN | NM_001185119 |
| hsa-miR-17-5p | MYNN | NM_018657 |
| hsa-miR-17-5p | NANOS1 | NM_199461 |
| hsa-miR-17-5p | NAPEPLD | NM_001122838 |
| hsa-miR-17-5p | NBEA | NM_001204197 |
| hsa-miR-17-5p | NBEA | NM_015678 |
| hsa-miR-17-5p | NCEH1 | NM_001146276 |
| hsa-miR-17-5p | NCEH1 | NM_001146277 |
| hsa-miR-17-5p | NCEH1 | NM_001146278 |
| hsa-miR-17-5p | NCEH1 | NM_020792 |
| hsa-miR-17-5p | NCOA3 | NM_001174087 |
| hsa-miR-17-5p | NCOA3 | NM_001174088 |
| hsa-miR-17-5p | NCOA3 | NM_006534 |
| hsa-miR-17-5p | NCOA3 | NM_181659 |
| hsa-miR-17-5p | NEDD4L | NM_001144964 |
| hsa-miR-17-5p | NEDD4L | NM_001144965 |
| hsa-miR-17-5p | NEDD4L | NM_001144966 |
| hsa-miR-17-5p | NEDD4L | NM_001144967 |
| hsa-miR-17-5p | NEDD4L | NM_001144968 |
| hsa-miR-17-5p | NEDD4L | NM_001144969 |
| hsa-miR-17-5p | NEDD4L | NM_001144970 |
| hsa-miR-17-5p | NEDD4L | NM_001144971 |
| hsa-miR-17-5p | NEDD4L | NM_015277 |
| hsa-miR-17-5p | NEK9 | NM_033116 |
| hsa-miR-17-5p | NHLRC3 | NM_001012754 |
| hsa-miR-17-5p | NHLRC3 | NM_001017370 |
| hsa-miR-17-5p | NPAS3 | NM_001164749 |
| hsa-miR-17-5p | NPAS3 | NM_001165893 |
| hsa-miR-17-5p | NPAS3 | NM_022123 |
| hsa-miR-17-5p | NPAS3 | NM_173159 |
| hsa-miR-17-5p | NPLOC4 | NM_017921 |
| hsa-miR-17-5p | NR2C2 | NM_003298 |
| hsa-miR-17-5p | NR4A2 | NM_006186 |
| hsa-miR-17-5p | NR4A3 | NM_006981 |
| hsa-miR-17-5p | NR4A3 | NM_173200 |
| hsa-miR-17-5p | NRIP3 | NM_020645 |
| hsa-miR-17-5p | NTN4 | NM_021229 |
| hsa-miR-17-5p | NUP35 | NM_138285 |
| hsa-miR-17-5p | OCRL | NM_000276 |
| hsa-miR-17-5p | OCRL | NM_001587 |
| hsa-miR-17-5p | ORMDL3 | NM_139280 |
| hsa-miR-17-5p | OSM | NM_020530 |
| hsa-miR-17-5p | P2RX4 | NM_002560 |
| hsa-miR-17-5p | PAFAH1B2 | NM_002572 |
| hsa-miR-17-5p | PAPOLA | NM_032632 |
| hsa-miR-17-5p | PAPOLB | NM_020144 |
| hsa-miR-17-5p | PBK | NM_018492 |
| hsa-miR-17-5p | PCDH15 | NM_001142771 |
| hsa-miR-17-5p | PCDH15 | NM_001142772 |
| hsa-miR-17-5p | PCDHA1 | NM_018900 |
| hsa-miR-17-5p | PCDHA1 | NM_031411 |
| hsa-miR-17-5p | PCDHA10 | NM_018901 |
| hsa-miR-17-5p | PCDHA10 | NM_031860 |
| hsa-miR-17-5p | PCDHA11 | NM_018902 |
| hsa-miR-17-5p | PCDHA12 | NM_018903 |
| hsa-miR-17-5p | PCDHA13 | NM_018904 |
| hsa-miR-17-5p | PCDHA2 | NM_018905 |
| hsa-miR-17-5p | PCDHA3 | NM_018906 |
| hsa-miR-17-5p | PCDHA4 | NM_018907 |
| hsa-miR-17-5p | PCDHA5 | NM_018908 |
| hsa-miR-17-5p | PCDHA6 | NM_018909 |
| hsa-miR-17-5p | PCDHA6 | NM_031849 |
| hsa-miR-17-5p | PCDHA7 | NM_018910 |
| hsa-miR-17-5p | PCDHA8 | NM_018911 |
| hsa-miR-17-5p | PCDHA9 | NM_031857 |
| hsa-miR-17-5p | PCDHAC1 | NM_018898 |
| hsa-miR-17-5p | PCDHAC2 | NM_018899 |
| hsa-miR-17-5p | PDGFRA | NM_006206 |
| hsa-miR-17-5p | PEX5L | NM_016559 |
| hsa-miR-17-5p | PFN2 | NM_002628 |
| hsa-miR-17-5p | PFN2 | NM_053024 |
| hsa-miR-17-5p | PGM2L1 | NM_173582 |
| hsa-miR-17-5p | PGP | NM_001042371 |
| hsa-miR-17-5p | PHC3 | NM_024947 |
| hsa-miR-17-5p | PIP4K2C | NM_001146258 |
| hsa-miR-17-5p | PKD2 | NM_000297 |
| hsa-miR-17-5p | PKN2 | NM_006256 |
| hsa-miR-17-5p | PKNOX1 | NM_004571 |
| hsa-miR-17-5p | PLSCR4 | NM_001128306 |
| hsa-miR-17-5p | PLSCR4 | NM_001177304 |
| hsa-miR-17-5p | PLSCR4 | NM_020353 |
| hsa-miR-17-5p | POLQ | NM_199420 |
| hsa-miR-17-5p | POLR3G | NM_006467 |
| hsa-miR-17-5p | POU6F1 | NM_002702 |
| hsa-miR-17-5p | PPARA | NM_001001928 |
| hsa-miR-17-5p | PPARA | NM_005036 |
| hsa-miR-17-5p | PPP1R12B | NM_001197131 |
| hsa-miR-17-5p | PPP1R12B | NM_002481 |
| hsa-miR-17-5p | PPP1R12B | NM_032103 |
| hsa-miR-17-5p | PPP1R12B | NM_032104 |
| hsa-miR-17-5p | PPP1R3B | NM_001201329 |
| hsa-miR-17-5p | PPP1R3B | NM_024607 |
| hsa-miR-17-5p | PPP3R1 | NM_000945 |
| hsa-miR-17-5p | PPP6C | NM_001123355 |
| hsa-miR-17-5p | PPP6C | NM_001123369 |
| hsa-miR-17-5p | PPP6C | NM_002721 |
| hsa-miR-17-5p | PRRG4 | NM_024081 |
| hsa-miR-17-5p | PTGDR | NM_000953 |
| hsa-miR-17-5p | PTPDC1 | NM_152422 |
| hsa-miR-17-5p | PTPDC1 | NM_177995 |
| hsa-miR-17-5p | PTPN4 | NM_002830 |
| hsa-miR-17-5p | PTPRD | NM_001040712 |
| hsa-miR-17-5p | PTPRD | NM_001171025 |
| hsa-miR-17-5p | PTPRD | NM_002839 |
| hsa-miR-17-5p | PTPRD | NM_130391 |
| hsa-miR-17-5p | PTPRD | NM_130392 |
| hsa-miR-17-5p | PTPRD | NM_130393 |
| hsa-miR-17-5p | RAB11FIP5 | NM_015470 |
| hsa-miR-17-5p | RAB22A | NM_020673 |
| hsa-miR-17-5p | RAB5B | NM_002868 |
| hsa-miR-17-5p | RAPGEF5 | NM_012294 |
| hsa-miR-17-5p | RAPGEFL1 | NM_016339 |
| hsa-miR-17-5p | RBL2 | NM_005611 |
| hsa-miR-17-5p | RBMS2 | NM_002898 |
| hsa-miR-17-5p | REEP3 | NM_001001330 |
| hsa-miR-17-5p | REST | NM_001193508 |
| hsa-miR-17-5p | REST | NM_005612 |
| hsa-miR-17-5p | RGL1 | NM_015149 |
| hsa-miR-17-5p | RGMB | NM_001012761 |
| hsa-miR-17-5p | RIMKLA | NM_173642 |
| hsa-miR-17-5p | RND3 | NM_005168 |
| hsa-miR-17-5p | RNF38 | NM_022781 |
| hsa-miR-17-5p | RNF38 | NM_194328 |
| hsa-miR-17-5p | RNF38 | NM_194329 |
| hsa-miR-17-5p | RNF38 | NM_194330 |
| hsa-miR-17-5p | RNF38 | NM_194332 |
| hsa-miR-17-5p | RNF6 | NM_005977 |
| hsa-miR-17-5p | RNF6 | NM_183043 |
| hsa-miR-17-5p | RNF6 | NM_183044 |
| hsa-miR-17-5p | RPS6KA2 | NM_001006932 |
| hsa-miR-17-5p | RPS6KA2 | NM_021135 |
| hsa-miR-17-5p | RPS6KA6 | NM_014496 |
| hsa-miR-17-5p | RRAGD | NM_021244 |
| hsa-miR-17-5p | RSRC2 | NM_023012 |
| hsa-miR-17-5p | RUFY2 | NM_017987 |
| hsa-miR-17-5p | RUNDC1 | NM_173079 |
| hsa-miR-17-5p | S1PR1 | NM_001400 |
| hsa-miR-17-5p | SACS | NM_014363 |
| hsa-miR-17-5p | SALL1 | NM_001127892 |
| hsa-miR-17-5p | SALL1 | NM_002968 |
| hsa-miR-17-5p | SAMD12 | NM_001101676 |
| hsa-miR-17-5p | SAR1B | NM_001033503 |
| hsa-miR-17-5p | SAR1B | NM_016103 |
| hsa-miR-17-5p | SASH1 | NM_015278 |
| hsa-miR-17-5p | SCD5 | NM_001037582 |
| hsa-miR-17-5p | SCN1A | NM_001165964 |
| hsa-miR-17-5p | SCN1A | NM_001202435 |
| hsa-miR-17-5p | SCN1A | NM_006920 |
| hsa-miR-17-5p | SGTB | NM_019072 |
| hsa-miR-17-5p | SH2D5 | NM_001103160 |
| hsa-miR-17-5p | SH2D5 | NM_001103161 |
| hsa-miR-17-5p | SIKE1 | NM_001102396 |
| hsa-miR-17-5p | SIKE1 | NM_025073 |
| hsa-miR-17-5p | SLC11A1 | NM_000578 |
| hsa-miR-17-5p | SLC16A9 | NM_194298 |
| hsa-miR-17-5p | SLC22A23 | NM_015482 |
| hsa-miR-17-5p | SLC22A23 | NM_021945 |
| hsa-miR-17-5p | SLC29A2 | NM_001532 |
| hsa-miR-17-5p | SLC2A4 | NM_001042 |
| hsa-miR-17-5p | SLC39A6 | NM_012319 |
| hsa-miR-17-5p | SLC40A1 | NM_014585 |
| hsa-miR-17-5p | SLC45A4 | NM_001080431 |
| hsa-miR-17-5p | SLC46A3 | NM_181785 |
| hsa-miR-17-5p | SLITRK3 | NM_014926 |
| hsa-miR-17-5p | SMAD5 | NM_001001419 |
| hsa-miR-17-5p | SMAD5 | NM_001001420 |
| hsa-miR-17-5p | SMAD5 | NM_005903 |
| hsa-miR-17-5p | SMOC1 | NM_001034852 |
| hsa-miR-17-5p | SMOC1 | NM_022137 |
| hsa-miR-17-5p | SOBP | NM_018013 |
| hsa-miR-17-5p | SOCS6 | NM_004232 |
| hsa-miR-17-5p | SOCS7 | NM_014598 |
| hsa-miR-17-5p | SRCIN1 | NM_025248 |
| hsa-miR-17-5p | SRGAP1 | NM_020762 |
| hsa-miR-17-5p | SRSF12 | NM_080743 |
| hsa-miR-17-5p | SSH1 | NM_018984 |
| hsa-miR-17-5p | ST3GAL1 | NM_003033 |
| hsa-miR-17-5p | ST3GAL1 | NM_173344 |
| hsa-miR-17-5p | ST6GALNAC6 | NM_013443 |
| hsa-miR-17-5p | ST8SIA2 | NM_006011 |
| hsa-miR-17-5p | STAT3 | NM_003150 |
| hsa-miR-17-5p | STAT3 | NM_139276 |
| hsa-miR-17-5p | STAT3 | NM_213662 |
| hsa-miR-17-5p | STC1 | NM_003155 |
| hsa-miR-17-5p | STK17B | NM_004226 |
| hsa-miR-17-5p | STK33 | NM_030906 |
| hsa-miR-17-5p | STK38 | NM_007271 |
| hsa-miR-17-5p | SYAP1 | NM_032796 |
| hsa-miR-17-5p | SYTL4 | NM_001129896 |
| hsa-miR-17-5p | SYTL4 | NM_001174068 |
| hsa-miR-17-5p | SYTL4 | NM_080737 |
| hsa-miR-17-5p | TAGAP | NM_054114 |
| hsa-miR-17-5p | TAGAP | NM_152133 |
| hsa-miR-17-5p | TAOK2 | NM_016151 |
| hsa-miR-17-5p | TBC1D15 | NM_001146213 |
| hsa-miR-17-5p | TBC1D15 | NM_001146214 |
| hsa-miR-17-5p | TBC1D15 | NM_022771 |
| hsa-miR-17-5p | TBC1D17 | NM_001168222 |
| hsa-miR-17-5p | TBC1D17 | NM_024682 |
| hsa-miR-17-5p | TBC1D8B | NM_017752 |
| hsa-miR-17-5p | TGFB1I1 | NM_001042454 |
| hsa-miR-17-5p | TGFB1I1 | NM_001164719 |
| hsa-miR-17-5p | TGFB1I1 | NM_015927 |
| hsa-miR-17-5p | TLE4 | NM_007005 |
| hsa-miR-17-5p | TMEM123 | NM_052932 |
| hsa-miR-17-5p | TMEM133 | NM_032021 |
| hsa-miR-17-5p | TMEM168 | NM_022484 |
| hsa-miR-17-5p | TMEM25 | NM_001144034 |
| hsa-miR-17-5p | TMEM25 | NM_001144035 |
| hsa-miR-17-5p | TMEM25 | NM_001144036 |
| hsa-miR-17-5p | TMEM25 | NM_032780 |
| hsa-miR-17-5p | TMEM64 | NM_001008495 |
| hsa-miR-17-5p | TMEM64 | NM_001146273 |
| hsa-miR-17-5p | TMUB2 | NM_001076674 |
| hsa-miR-17-5p | TMUB2 | NM_024107 |
| hsa-miR-17-5p | TMUB2 | NM_177441 |
| hsa-miR-17-5p | TNFAIP1 | NM_021137 |
| hsa-miR-17-5p | TNKS1BP1 | NM_033396 |
| hsa-miR-17-5p | TNKS2 | NM_025235 |
| hsa-miR-17-5p | TNRC6B | NM_001024843 |
| hsa-miR-17-5p | TNRC6B | NM_001162501 |
| hsa-miR-17-5p | TNRC6B | NM_015088 |
| hsa-miR-17-5p | TP53INP1 | NM_001135733 |
| hsa-miR-17-5p | TP53INP1 | NM_033285 |
| hsa-miR-17-5p | TP73 | NM_001126240 |
| hsa-miR-17-5p | TP73 | NM_001126241 |
| hsa-miR-17-5p | TP73 | NM_001126242 |
| hsa-miR-17-5p | TP73 | NM_001204184 |
| hsa-miR-17-5p | TP73 | NM_001204185 |
| hsa-miR-17-5p | TP73 | NM_001204186 |
| hsa-miR-17-5p | TP73 | NM_001204187 |
| hsa-miR-17-5p | TP73 | NM_001204188 |
| hsa-miR-17-5p | TP73 | NM_001204189 |
| hsa-miR-17-5p | TP73 | NM_001204190 |
| hsa-miR-17-5p | TP73 | NM_001204191 |
| hsa-miR-17-5p | TP73 | NM_001204192 |
| hsa-miR-17-5p | TP73 | NM_005427 |
| hsa-miR-17-5p | TPRG1L | NM_182752 |
| hsa-miR-17-5p | TRIM3 | NM_006458 |
| hsa-miR-17-5p | TRIM3 | NM_033278 |
| hsa-miR-17-5p | TRIM66 | NM_014818 |
| hsa-miR-17-5p | TRIM8 | NM_030912 |
| hsa-miR-17-5p | TRPS1 | NM_014112 |
| hsa-miR-17-5p | TRPV6 | NM_018646 |
| hsa-miR-17-5p | TSHZ3 | NM_020856 |
| hsa-miR-17-5p | TUSC2 | NM_007275 |
| hsa-miR-17-5p | TXLNA | NM_175852 |
| hsa-miR-17-5p | U2SURP | NM_001080415 |
| hsa-miR-17-5p | UBE2W | NM_001001481 |
| hsa-miR-17-5p | UBE2W | NM_018299 |
| hsa-miR-17-5p | UBE3C | NM_014671 |
| hsa-miR-17-5p | UBXN2A | NM_181713 |
| hsa-miR-17-5p | UCP3 | NM_003356 |
| hsa-miR-17-5p | UCP3 | NM_022803 |
| hsa-miR-17-5p | UNKL | NM_001193388 |
| hsa-miR-17-5p | UNKL | NM_001193389 |
| hsa-miR-17-5p | UPF3A | NM_023011 |
| hsa-miR-17-5p | UPF3A | NM_080687 |
| hsa-miR-17-5p | USP31 | NM_020718 |
| hsa-miR-17-5p | USP53 | NM_019050 |
| hsa-miR-17-5p | VLDLR | NM_001018056 |
| hsa-miR-17-5p | VLDLR | NM_003383 |
| hsa-miR-17-5p | VPS26A | NM_004896 |
| hsa-miR-17-5p | VSX1 | NM_014588 |
| hsa-miR-17-5p | WDFY2 | NM_052950 |
| hsa-miR-17-5p | WDR37 | NM_014023 |
| hsa-miR-17-5p | WEE1 | NM_001143976 |
| hsa-miR-17-5p | WEE1 | NM_003390 |
| hsa-miR-17-5p | WFS1 | NM_001145853 |
| hsa-miR-17-5p | WFS1 | NM_006005 |
| hsa-miR-17-5p | YPEL4 | NM_145008 |
| hsa-miR-17-5p | YTHDF3 | NM_152758 |
| hsa-miR-17-5p | ZBTB33 | NM_001184742 |
| hsa-miR-17-5p | ZBTB33 | NM_006777 |
| hsa-miR-17-5p | ZBTB4 | NM_001128833 |
| hsa-miR-17-5p | ZBTB4 | NM_020899 |
| hsa-miR-17-5p | ZBTB44 | NM_014155 |
| hsa-miR-17-5p | ZBTB6 | NM_006626 |
| hsa-miR-17-5p | ZBTB9 | NM_152735 |
| hsa-miR-17-5p | ZDHHC1 | NM_013304 |
| hsa-miR-17-5p | ZDHHC8 | NM_001185024 |
| hsa-miR-17-5p | ZDHHC8 | NM_013373 |
| hsa-miR-17-5p | ZFP3 | NM_153018 |
| hsa-miR-17-5p | ZFPM2 | NM_012082 |
| hsa-miR-17-5p | ZFYVE16 | NM_001105251 |
| hsa-miR-17-5p | ZFYVE16 | NM_014733 |
| hsa-miR-17-5p | ZFYVE26 | NM_015346 |
| hsa-miR-17-5p | ZNF2 | NM_001017396 |
| hsa-miR-17-5p | ZNF2 | NM_021088 |
| hsa-miR-17-5p | ZNF367 | NM_153695 |
| hsa-miR-17-5p | ZNF512B | NM_020713 |
| hsa-miR-17-5p | ZNF597 | NM_152457 |
| hsa-miR-17-5p | ZNF800 | NM_176814 |
| hsa-miR-17-5p | ZNFX1 | NM_021035 |
| hsa-miR-17-5p | ZSCAN20 | NM_145238 |
| hsa-miR-17-5p | ZXDA | NM_007156 |
| hsa-miR-182-5p | A1CF | NM_001198818 |
| hsa-miR-182-5p | A1CF | NM_001198819 |
| hsa-miR-182-5p | A1CF | NM_001198820 |
| hsa-miR-182-5p | A1CF | NM_014576 |
| hsa-miR-182-5p | A1CF | NM_138932 |
| hsa-miR-182-5p | A1CF | NM_138933 |
| hsa-miR-182-5p | AAK1 | NM_014911 |
| hsa-miR-182-5p | AATK | NM_001080395 |
| hsa-miR-182-5p | AATK | NM_004920 |
| hsa-miR-182-5p | ACTR2 | NM_001005386 |
| hsa-miR-182-5p | ACTR2 | NM_005722 |
| hsa-miR-182-5p | ADAM22 | NM_016351 |
| hsa-miR-182-5p | ADAM22 | NM_021722 |
| hsa-miR-182-5p | ADAM22 | NM_021723 |
| hsa-miR-182-5p | ADAMTS9 | NM_182920 |
| hsa-miR-182-5p | ANGPTL1 | NM_004673 |
| hsa-miR-182-5p | ANK3 | NM_001149 |
| hsa-miR-182-5p | ANK3 | NM_001204403 |
| hsa-miR-182-5p | ANK3 | NM_001204404 |
| hsa-miR-182-5p | ANK3 | NM_020987 |
| hsa-miR-182-5p | APLN | NM_017413 |
| hsa-miR-182-5p | ARCN1 | NM_001142281 |
| hsa-miR-182-5p | ARCN1 | NM_001655 |
| hsa-miR-182-5p | ARF4 | NM_001660 |
| hsa-miR-182-5p | ARHGAP29 | NM_004815 |
| hsa-miR-182-5p | ARHGEF3 | NM_001128615 |
| hsa-miR-182-5p | ARHGEF3 | NM_001128616 |
| hsa-miR-182-5p | ARHGEF3 | NM_019555 |
| hsa-miR-182-5p | ARHGEF7 | NM_001113511 |
| hsa-miR-182-5p | ARHGEF7 | NM_001113512 |
| hsa-miR-182-5p | ARHGEF7 | NM_145735 |
| hsa-miR-182-5p | ARMC1 | NM_018120 |
| hsa-miR-182-5p | ATAD2B | NM_001242338 |
| hsa-miR-182-5p | ATAD2B | NM_017552 |
| hsa-miR-182-5p | ATAT1 | NM_024909 |
| hsa-miR-182-5p | ATP7A | NM_000052 |
| hsa-miR-182-5p | ATXN7 | NM_000333 |
| hsa-miR-182-5p | ATXN7 | NM_001128149 |
| hsa-miR-182-5p | ATXN7 | NM_001177387 |
| hsa-miR-182-5p | BAG4 | NM_001204878 |
| hsa-miR-182-5p | BAG4 | NM_004874 |
| hsa-miR-182-5p | BCL11A | NM_022893 |
| hsa-miR-182-5p | BCL2 | NM_000633 |
| hsa-miR-182-5p | BCL2L13 | NM_015367 |
| hsa-miR-182-5p | BDNF | NM_001143805 |
| hsa-miR-182-5p | BDNF | NM_001143806 |
| hsa-miR-182-5p | BDNF | NM_001143807 |
| hsa-miR-182-5p | BDNF | NM_001143808 |
| hsa-miR-182-5p | BDNF | NM_001143809 |
| hsa-miR-182-5p | BDNF | NM_001143810 |
| hsa-miR-182-5p | BDNF | NM_001143811 |
| hsa-miR-182-5p | BDNF | NM_001143812 |
| hsa-miR-182-5p | BDNF | NM_001143813 |
| hsa-miR-182-5p | BDNF | NM_001143814 |
| hsa-miR-182-5p | BDNF | NM_001143816 |
| hsa-miR-182-5p | BDNF | NM_001709 |
| hsa-miR-182-5p | BDNF | NM_170731 |
| hsa-miR-182-5p | BDNF | NM_170732 |
| hsa-miR-182-5p | BDNF | NM_170733 |
| hsa-miR-182-5p | BDNF | NM_170734 |
| hsa-miR-182-5p | BDNF | NM_170735 |
| hsa-miR-182-5p | BRD4 | NM_014299 |
| hsa-miR-182-5p | BRMS1L | NM_032352 |
| hsa-miR-182-5p | BTBD11 | NM_001017523 |
| hsa-miR-182-5p | BTBD11 | NM_001018072 |
| hsa-miR-182-5p | C7orf60 | NM_152556 |
| hsa-miR-182-5p | C8orf37 | NM_177965 |
| hsa-miR-182-5p | CACNB4 | NM_000726 |
| hsa-miR-182-5p | CACNB4 | NM_001005746 |
| hsa-miR-182-5p | CACNB4 | NM_001005747 |
| hsa-miR-182-5p | CACNB4 | NM_001145798 |
| hsa-miR-182-5p | CCDC117 | NM_173510 |
| hsa-miR-182-5p | CD2AP | NM_012120 |
| hsa-miR-182-5p | CD47 | NM_001777 |
| hsa-miR-182-5p | CD47 | NM_198793 |
| hsa-miR-182-5p | CELF2 | NM_001025077 |
| hsa-miR-182-5p | CELF6 | NM_001172684 |
| hsa-miR-182-5p | CELF6 | NM_001172685 |
| hsa-miR-182-5p | CELF6 | NM_052840 |
| hsa-miR-182-5p | CEP128 | NM_152446 |
| hsa-miR-182-5p | CHMP1A | NM_001083314 |
| hsa-miR-182-5p | CHMP1A | NM_002768 |
| hsa-miR-182-5p | CHMP1B | NM_020412 |
| hsa-miR-182-5p | CHST11 | NM_001173982 |
| hsa-miR-182-5p | CHST11 | NM_018413 |
| hsa-miR-182-5p | CLPTM1L | NM_030782 |
| hsa-miR-182-5p | COL4A4 | NM_000092 |
| hsa-miR-182-5p | COL5A1 | NM_000093 |
| hsa-miR-182-5p | CREB1 | NM_004379 |
| hsa-miR-182-5p | CREB1 | NM_134442 |
| hsa-miR-182-5p | CREB3L1 | NM_052854 |
| hsa-miR-182-5p | CTDSP2 | NM_005730 |
| hsa-miR-182-5p | CTTN | NM_005231 |
| hsa-miR-182-5p | CTTN | NM_138565 |
| hsa-miR-182-5p | DCP2 | NM_001242377 |
| hsa-miR-182-5p | DCP2 | NM_152624 |
| hsa-miR-182-5p | DENR | NM_003677 |
| hsa-miR-182-5p | DIO1 | NM_000792 |
| hsa-miR-182-5p | DIO1 | NM_001039715 |
| hsa-miR-182-5p | DKK2 | NM_014421 |
| hsa-miR-182-5p | DNAJC15 | NM_013238 |
| hsa-miR-182-5p | DOK4 | NM_018110 |
| hsa-miR-182-5p | DSG2 | NM_001943 |
| hsa-miR-182-5p | DTNBP1 | NM_032122 |
| hsa-miR-182-5p | EFNA5 | NM_001962 |
| hsa-miR-182-5p | EGLN1 | NM_022051 |
| hsa-miR-182-5p | EIF2S1 | NM_004094 |
| hsa-miR-182-5p | EIF5 | NM_001969 |
| hsa-miR-182-5p | EIF5 | NM_183004 |
| hsa-miR-182-5p | ELAVL2 | NM_001171195 |
| hsa-miR-182-5p | ELAVL2 | NM_001171197 |
| hsa-miR-182-5p | ELAVL2 | NM_004432 |
| hsa-miR-182-5p | ELMO1 | NM_001039459 |
| hsa-miR-182-5p | ELMO1 | NM_001206480 |
| hsa-miR-182-5p | ELMO1 | NM_001206482 |
| hsa-miR-182-5p | ELMO1 | NM_014800 |
| hsa-miR-182-5p | ENY2 | NM_001193557 |
| hsa-miR-182-5p | ENY2 | NM_020189 |
| hsa-miR-182-5p | EPAS1 | NM_001430 |
| hsa-miR-182-5p | EPHB1 | NM_004441 |
| hsa-miR-182-5p | EPM2A | NM_005670 |
| hsa-miR-182-5p | EVI5 | NM_005665 |
| hsa-miR-182-5p | EXOC4 | NM_021807 |
| hsa-miR-182-5p | FAM122A | NM_138333 |
| hsa-miR-182-5p | FAM171A1 | NM_001010924 |
| hsa-miR-182-5p | FAM78A | NM_033387 |
| hsa-miR-182-5p | FAM81A | NM_152450 |
| hsa-miR-182-5p | FAT3 | NM_001008781 |
| hsa-miR-182-5p | FBXW7 | NM_001013415 |
| hsa-miR-182-5p | FBXW7 | NM_018315 |
| hsa-miR-182-5p | FBXW7 | NM_033632 |
| hsa-miR-182-5p | FGF9 | NM_002010 |
| hsa-miR-182-5p | FLNB | NM_001164317 |
| hsa-miR-182-5p | FLNB | NM_001164318 |
| hsa-miR-182-5p | FLNB | NM_001164319 |
| hsa-miR-182-5p | FLNB | NM_001457 |
| hsa-miR-182-5p | FLOT1 | NM_005803 |
| hsa-miR-182-5p | FOXF2 | NM_001452 |
| hsa-miR-182-5p | FOXO3 | NM_001455 |
| hsa-miR-182-5p | FOXO3 | NM_201559 |
| hsa-miR-182-5p | FRS2 | NM_001042555 |
| hsa-miR-182-5p | FRS2 | NM_006654 |
| hsa-miR-182-5p | FXR1 | NM_001013438 |
| hsa-miR-182-5p | FXR1 | NM_001013439 |
| hsa-miR-182-5p | FXR1 | NM_005087 |
| hsa-miR-182-5p | GIT2 | NM_001135213 |
| hsa-miR-182-5p | GIT2 | NM_001135214 |
| hsa-miR-182-5p | GIT2 | NM_014776 |
| hsa-miR-182-5p | GIT2 | NM_057169 |
| hsa-miR-182-5p | GIT2 | NM_057170 |
| hsa-miR-182-5p | GMFB | NM_004124 |
| hsa-miR-182-5p | GNAQ | NM_002072 |
| hsa-miR-182-5p | GPC1 | NM_002081 |
| hsa-miR-182-5p | GXYLT1 | NM_001099650 |
| hsa-miR-182-5p | GXYLT1 | NM_173601 |
| hsa-miR-182-5p | H6PD | NM_004285 |
| hsa-miR-182-5p | HDAC9 | NM_001204144 |
| hsa-miR-182-5p | HDAC9 | NM_001204145 |
| hsa-miR-182-5p | HDAC9 | NM_001204146 |
| hsa-miR-182-5p | HDAC9 | NM_001204147 |
| hsa-miR-182-5p | HDAC9 | NM_001204148 |
| hsa-miR-182-5p | HDAC9 | NM_014707 |
| hsa-miR-182-5p | HMCN1 | NM_031935 |
| hsa-miR-182-5p | HMGCLL1 | NM_001042406 |
| hsa-miR-182-5p | HMGCLL1 | NM_019036 |
| hsa-miR-182-5p | HOOK3 | NM_032410 |
| hsa-miR-182-5p | HSBP1 | NM_001537 |
| hsa-miR-182-5p | IGF1R | NM_000875 |
| hsa-miR-182-5p | IGSF3 | NM_001007237 |
| hsa-miR-182-5p | IGSF3 | NM_001542 |
| hsa-miR-182-5p | INSL5 | NM_005478 |
| hsa-miR-182-5p | ISL1 | NM_002202 |
| hsa-miR-182-5p | ITGB8 | NM_002214 |
| hsa-miR-182-5p | JAZF1 | NM_175061 |
| hsa-miR-182-5p | KCNMB2 | NM_005832 |
| hsa-miR-182-5p | KCNMB2 | NM_181361 |
| hsa-miR-182-5p | KCNN3 | NM_001204087 |
| hsa-miR-182-5p | KCNN3 | NM_002249 |
| hsa-miR-182-5p | KCNN3 | NM_170782 |
| hsa-miR-182-5p | KDM2B | NM_001005366 |
| hsa-miR-182-5p | KDM2B | NM_032590 |
| hsa-miR-182-5p | KIAA1217 | NM_001098500 |
| hsa-miR-182-5p | KIAA1217 | NM_019590 |
| hsa-miR-182-5p | KIAA1324L | NM_001142749 |
| hsa-miR-182-5p | KIAA1324L | NM_152748 |
| hsa-miR-182-5p | KIF19 | NM_153209 |
| hsa-miR-182-5p | KIF1B | NM_015074 |
| hsa-miR-182-5p | KLHL31 | NM_001003760 |
| hsa-miR-182-5p | KPNA3 | NM_002267 |
| hsa-miR-182-5p | LARP1 | NM_015315 |
| hsa-miR-182-5p | LDB3 | NM_001080114 |
| hsa-miR-182-5p | LDB3 | NM_001171610 |
| hsa-miR-182-5p | LDB3 | NM_007078 |
| hsa-miR-182-5p | LHX1 | NM_005568 |
| hsa-miR-182-5p | LPHN1 | NM_001008701 |
| hsa-miR-182-5p | LPHN1 | NM_014921 |
| hsa-miR-182-5p | LPPR4 | NM_001166252 |
| hsa-miR-182-5p | LPPR4 | NM_014839 |
| hsa-miR-182-5p | LSM14A | NM_001114093 |
| hsa-miR-182-5p | LSM14A | NM_015578 |
| hsa-miR-182-5p | MAF | NM_001031804 |
| hsa-miR-182-5p | MAP1B | NM_005909 |
| hsa-miR-182-5p | MEF2D | NM_005920 |
| hsa-miR-182-5p | MET | NM_000245 |
| hsa-miR-182-5p | MET | NM_001127500 |
| hsa-miR-182-5p | MITF | NM_000248 |
| hsa-miR-182-5p | MITF | NM_001184967 |
| hsa-miR-182-5p | MITF | NM_006722 |
| hsa-miR-182-5p | MITF | NM_198158 |
| hsa-miR-182-5p | MITF | NM_198159 |
| hsa-miR-182-5p | MITF | NM_198177 |
| hsa-miR-182-5p | MITF | NM_198178 |
| hsa-miR-182-5p | MTSS1 | NM_014751 |
| hsa-miR-182-5p | MYO19 | NM_001163735 |
| hsa-miR-182-5p | MYO19 | NM_025109 |
| hsa-miR-182-5p | MYRIP | NM_015460 |
| hsa-miR-182-5p | MYT1L | NM_015025 |
| hsa-miR-182-5p | N4BP1 | NM_153029 |
| hsa-miR-182-5p | NCALD | NM_001040624 |
| hsa-miR-182-5p | NCALD | NM_001040625 |
| hsa-miR-182-5p | NCALD | NM_001040626 |
| hsa-miR-182-5p | NCALD | NM_001040627 |
| hsa-miR-182-5p | NCALD | NM_001040628 |
| hsa-miR-182-5p | NCALD | NM_001040629 |
| hsa-miR-182-5p | NCALD | NM_001040630 |
| hsa-miR-182-5p | NCALD | NM_032041 |
| hsa-miR-182-5p | NCOA4 | NM_001145260 |
| hsa-miR-182-5p | NCOA4 | NM_001145261 |
| hsa-miR-182-5p | NCOA4 | NM_001145262 |
| hsa-miR-182-5p | NCOA4 | NM_001145263 |
| hsa-miR-182-5p | NCOA4 | NM_005437 |
| hsa-miR-182-5p | NHS | NM_001136024 |
| hsa-miR-182-5p | NHS | NM_198270 |
| hsa-miR-182-5p | NIPBL | NM_015384 |
| hsa-miR-182-5p | NKX2-2 | NM_002509 |
| hsa-miR-182-5p | NPM1 | NM_002520 |
| hsa-miR-182-5p | NPM1 | NM_199185 |
| hsa-miR-182-5p | NRF1 | NM_001040110 |
| hsa-miR-182-5p | NRF1 | NM_005011 |
| hsa-miR-182-5p | NRN1 | NM_016588 |
| hsa-miR-182-5p | NT5DC3 | NM_001031701 |
| hsa-miR-182-5p | NUAK1 | NM_014840 |
| hsa-miR-182-5p | NUDT21 | NM_007006 |
| hsa-miR-182-5p | NUMB | NM_001005743 |
| hsa-miR-182-5p | NUMB | NM_001005744 |
| hsa-miR-182-5p | NUMB | NM_001005745 |
| hsa-miR-182-5p | NUMB | NM_003744 |
| hsa-miR-182-5p | NUP50 | NM_007172 |
| hsa-miR-182-5p | NUP50 | NM_153645 |
| hsa-miR-182-5p | OAS2 | NM_016817 |
| hsa-miR-182-5p | ONECUT2 | NM_004852 |
| hsa-miR-182-5p | PABPC1L2A | NM_001012977 |
| hsa-miR-182-5p | PABPC1L2B | NM_001042506 |
| hsa-miR-182-5p | PAFAH1B2 | NM_002572 |
| hsa-miR-182-5p | PAIP2 | NM_001033112 |
| hsa-miR-182-5p | PAIP2 | NM_016480 |
| hsa-miR-182-5p | PAN2 | NM_001127460 |
| hsa-miR-182-5p | PAN2 | NM_001166279 |
| hsa-miR-182-5p | PAN2 | NM_014871 |
| hsa-miR-182-5p | PBX3 | NM_001134778 |
| hsa-miR-182-5p | PBX3 | NM_006195 |
| hsa-miR-182-5p | PC | NM_000920 |
| hsa-miR-182-5p | PC | NM_001040716 |
| hsa-miR-182-5p | PC | NM_022172 |
| hsa-miR-182-5p | PCDH18 | NM_019035 |
| hsa-miR-182-5p | PCDH8 | NM_002590 |
| hsa-miR-182-5p | PCDH8 | NM_032949 |
| hsa-miR-182-5p | PCMT1 | NM_005389 |
| hsa-miR-182-5p | PDPN | NM_001006624 |
| hsa-miR-182-5p | PDPN | NM_001006625 |
| hsa-miR-182-5p | PDPN | NM_006474 |
| hsa-miR-182-5p | PDPN | NM_198389 |
| hsa-miR-182-5p | PEG10 | NM_015068 |
| hsa-miR-182-5p | PHF13 | NM_153812 |
| hsa-miR-182-5p | PHF8 | NM_001184896 |
| hsa-miR-182-5p | PHF8 | NM_015107 |
| hsa-miR-182-5p | PLD1 | NM_001130081 |
| hsa-miR-182-5p | PLD1 | NM_002662 |
| hsa-miR-182-5p | PPFIBP2 | NM_003621 |
| hsa-miR-182-5p | PPM1A | NM_021003 |
| hsa-miR-182-5p | PPM1A | NM_177952 |
| hsa-miR-182-5p | PPM1E | NM_014906 |
| hsa-miR-182-5p | PPP1R2 | NM_006241 |
| hsa-miR-182-5p | PPP1R3D | NM_006242 |
| hsa-miR-182-5p | PRAF2 | NM_007213 |
| hsa-miR-182-5p | PRDM1 | NM_001198 |
| hsa-miR-182-5p | PRDM1 | NM_182907 |
| hsa-miR-182-5p | PRKAR1A | NM_002734 |
| hsa-miR-182-5p | PRKAR1A | NM_212471 |
| hsa-miR-182-5p | PRKAR1A | NM_212472 |
| hsa-miR-182-5p | PRKD1 | NM_002742 |
| hsa-miR-182-5p | PRUNE2 | NM_015225 |
| hsa-miR-182-5p | PTPRG | NM_002841 |
| hsa-miR-182-5p | RAB10 | NM_016131 |
| hsa-miR-182-5p | RAB3GAP2 | NM_012414 |
| hsa-miR-182-5p | RAPGEF5 | NM_012294 |
| hsa-miR-182-5p | RARG | NM_000966 |
| hsa-miR-182-5p | RARG | NM_001042728 |
| hsa-miR-182-5p | RASA1 | NM_002890 |
| hsa-miR-182-5p | RASA1 | NM_022650 |
| hsa-miR-182-5p | RD3 | NM_001164688 |
| hsa-miR-182-5p | RD3 | NM_183059 |
| hsa-miR-182-5p | RDX | NM_002906 |
| hsa-miR-182-5p | RECK | NM_021111 |
| hsa-miR-182-5p | RMND5A | NM_022780 |
| hsa-miR-182-5p | RNF222 | NM_001146684 |
| hsa-miR-182-5p | S100PBP | NM_022753 |
| hsa-miR-182-5p | SAMD12 | NM_001101676 |
| hsa-miR-182-5p | SAMD4A | NM_001161576 |
| hsa-miR-182-5p | SAMD4A | NM_001161577 |
| hsa-miR-182-5p | SAMD4A | NM_015589 |
| hsa-miR-182-5p | SAMD5 | NM_001030060 |
| hsa-miR-182-5p | SCARB2 | NM_001204255 |
| hsa-miR-182-5p | SCARB2 | NM_005506 |
| hsa-miR-182-5p | SCML4 | NM_198081 |
| hsa-miR-182-5p | 43717 | NM_001113491 |
| hsa-miR-182-5p | 43717 | NM_001113492 |
| hsa-miR-182-5p | 43717 | NM_001113493 |
| hsa-miR-182-5p | 43717 | NM_001113494 |
| hsa-miR-182-5p | 43717 | NM_001113495 |
| hsa-miR-182-5p | 43717 | NM_001113496 |
| hsa-miR-182-5p | 43717 | NM_006640 |
| hsa-miR-182-5p | SESN2 | NM_031459 |
| hsa-miR-182-5p | SETD7 | NM_030648 |
| hsa-miR-182-5p | SGMS2 | NM_001136257 |
| hsa-miR-182-5p | SGMS2 | NM_001136258 |
| hsa-miR-182-5p | SGMS2 | NM_152621 |
| hsa-miR-182-5p | SH2D1A | NM_001114937 |
| hsa-miR-182-5p | SH2D1A | NM_002351 |
| hsa-miR-182-5p | SH3BGRL | NM_003022 |
| hsa-miR-182-5p | SH3BP4 | NM_014521 |
| hsa-miR-182-5p | SHC4 | NM_203349 |
| hsa-miR-182-5p | SHOX | NM_000451 |
| hsa-miR-182-5p | SLAIN2 | NM_020846 |
| hsa-miR-182-5p | SLC1A2 | NM_001195728 |
| hsa-miR-182-5p | SLC1A2 | NM_004171 |
| hsa-miR-182-5p | SLC30A1 | NM_021194 |
| hsa-miR-182-5p | SLC30A7 | NM_001144884 |
| hsa-miR-182-5p | SLC30A7 | NM_133496 |
| hsa-miR-182-5p | SLC39A9 | NM_018375 |
| hsa-miR-182-5p | SLC43A2 | NM_152346 |
| hsa-miR-182-5p | SLITRK4 | NM_001184749 |
| hsa-miR-182-5p | SLITRK4 | NM_001184750 |
| hsa-miR-182-5p | SLITRK4 | NM_173078 |
| hsa-miR-182-5p | SNAP23 | NM_003825 |
| hsa-miR-182-5p | SNAP23 | NM_130798 |
| hsa-miR-182-5p | SNX17 | NM_014748 |
| hsa-miR-182-5p | SNX30 | NM_001012994 |
| hsa-miR-182-5p | SOX11 | NM_003108 |
| hsa-miR-182-5p | SPATA13 | NM_001166271 |
| hsa-miR-182-5p | SPATA13 | NM_153023 |
| hsa-miR-182-5p | SPIN1 | NM_006717 |
| hsa-miR-182-5p | SRSF6 | NM_006275 |
| hsa-miR-182-5p | STK19 | NM_004197 |
| hsa-miR-182-5p | STK19 | NM_032454 |
| hsa-miR-182-5p | SYNCRIP | NM_001159673 |
| hsa-miR-182-5p | SYNCRIP | NM_001159674 |
| hsa-miR-182-5p | SYNCRIP | NM_001159676 |
| hsa-miR-182-5p | SYNCRIP | NM_001159677 |
| hsa-miR-182-5p | SYNE2 | NM_015180 |
| hsa-miR-182-5p | SYNE2 | NM_182910 |
| hsa-miR-182-5p | SYNE2 | NM_182913 |
| hsa-miR-182-5p | SYNE2 | NM_182914 |
| hsa-miR-182-5p | TAF15 | NM_003487 |
| hsa-miR-182-5p | TAF15 | NM_139215 |
| hsa-miR-182-5p | TAF4B | NM_005640 |
| hsa-miR-182-5p | TAOK1 | NM_020791 |
| hsa-miR-182-5p | TAOK1 | NM_025142 |
| hsa-miR-182-5p | TMEM189 | NM_001162505 |
| hsa-miR-182-5p | TMEM189 | NM_199129 |
| hsa-miR-182-5p | TMEM67 | NM_001142301 |
| hsa-miR-182-5p | TMEM67 | NM_153704 |
| hsa-miR-182-5p | TMOD2 | NM_001142885 |
| hsa-miR-182-5p | TMOD2 | NM_014548 |
| hsa-miR-182-5p | TNFSF11 | NM_003701 |
| hsa-miR-182-5p | TNFSF11 | NM_033012 |
| hsa-miR-182-5p | TP53INP2 | NM_021202 |
| hsa-miR-182-5p | TSN | NM_004622 |
| hsa-miR-182-5p | TSNAX | NM_005999 |
| hsa-miR-182-5p | TXNL1 | NM_004786 |
| hsa-miR-182-5p | UBASH3B | NM_032873 |
| hsa-miR-182-5p | UBE2W | NM_001001481 |
| hsa-miR-182-5p | UBE2W | NM_018299 |
| hsa-miR-182-5p | UBE3C | NM_014671 |
| hsa-miR-182-5p | USP13 | NM_003940 |
| hsa-miR-182-5p | USP6NL | NM_001080491 |
| hsa-miR-182-5p | USP6NL | NM_014688 |
| hsa-miR-182-5p | VAMP3 | NM_004781 |
| hsa-miR-182-5p | VEZT | NM_017599 |
| hsa-miR-182-5p | VGLL3 | NM_016206 |
| hsa-miR-182-5p | WDR47 | NM_001142550 |
| hsa-miR-182-5p | WDR47 | NM_001142551 |
| hsa-miR-182-5p | WDR47 | NM_014969 |
| hsa-miR-182-5p | WDR76 | NM_001167941 |
| hsa-miR-182-5p | WDR76 | NM_024908 |
| hsa-miR-182-5p | WDR82 | NM_025222 |
| hsa-miR-182-5p | WHSC1 | NM_001042424 |
| hsa-miR-182-5p | WHSC1 | NM_133330 |
| hsa-miR-182-5p | WHSC1 | NM_133331 |
| hsa-miR-182-5p | WHSC1 | NM_133335 |
| hsa-miR-182-5p | WIPI2 | NM_001033518 |
| hsa-miR-182-5p | WIPI2 | NM_001033519 |
| hsa-miR-182-5p | WIPI2 | NM_001033520 |
| hsa-miR-182-5p | WIPI2 | NM_015610 |
| hsa-miR-182-5p | WIPI2 | NM_016003 |
| hsa-miR-182-5p | WWC2 | NM_024949 |
| hsa-miR-182-5p | ZBTB8B | NM_001145720 |
| hsa-miR-182-5p | ZDHHC15 | NM_001146256 |
| hsa-miR-182-5p | ZDHHC15 | NM_144969 |
| hsa-miR-182-5p | ZFC3H1 | NM_144982 |
| hsa-miR-182-5p | ZFP30 | NM_014898 |
| hsa-miR-182-5p | ZFP36 | NM_003407 |
| hsa-miR-182-5p | ZMPSTE24 | NM_005857 |
| hsa-miR-182-5p | ZNF24 | NM_006965 |
| hsa-miR-182-5p | ZNF706 | NM_001042510 |
| hsa-miR-182-5p | ZNF706 | NM_016096 |
| hsa-miR-182-5p | ZNRF1 | NM_032268 |
| hsa-miR-188-5p | AGFG1 | NM_001135187 |
| hsa-miR-188-5p | AGFG1 | NM_001135188 |
| hsa-miR-188-5p | AGFG1 | NM_001135189 |
| hsa-miR-188-5p | AGFG1 | NM_004504 |
| hsa-miR-188-5p | ATXN7 | NM_000333 |
| hsa-miR-188-5p | ATXN7 | NM_001128149 |
| hsa-miR-188-5p | ATXN7 | NM_001177387 |
| hsa-miR-188-5p | BEND4 | NM_001159547 |
| hsa-miR-188-5p | CATSPER2 | NM_172097 |
| hsa-miR-188-5p | CBFB | NM_001755 |
| hsa-miR-188-5p | CBFB | NM_022845 |
| hsa-miR-188-5p | CD2AP | NM_012120 |
| hsa-miR-188-5p | CDC25B | NM_004358 |
| hsa-miR-188-5p | CDC25B | NM_021872 |
| hsa-miR-188-5p | CDC25B | NM_021873 |
| hsa-miR-188-5p | CPT1A | NM_001876 |
| hsa-miR-188-5p | CX3CR1 | NM_001171171 |
| hsa-miR-188-5p | CX3CR1 | NM_001171172 |
| hsa-miR-188-5p | CX3CR1 | NM_001171174 |
| hsa-miR-188-5p | CX3CR1 | NM_001337 |
| hsa-miR-188-5p | FBXO45 | NM_001105573 |
| hsa-miR-188-5p | H3F3B | NM_005324 |
| hsa-miR-188-5p | IGLON5 | NM_001101372 |
| hsa-miR-188-5p | KCNG3 | NM_133329 |
| hsa-miR-188-5p | KCNG3 | NM_172344 |
| hsa-miR-188-5p | KPNA3 | NM_002267 |
| hsa-miR-188-5p | MAGI1 | NM_015520 |
| hsa-miR-188-5p | MAP3K9 | NM_033141 |
| hsa-miR-188-5p | MGAT3 | NM_001098270 |
| hsa-miR-188-5p | MGAT3 | NM_002409 |
| hsa-miR-188-5p | MSX1 | NM_002448 |
| hsa-miR-188-5p | NEK6 | NM_001145001 |
| hsa-miR-188-5p | NEK6 | NM_001166167 |
| hsa-miR-188-5p | NEK6 | NM_001166168 |
| hsa-miR-188-5p | NEK6 | NM_001166169 |
| hsa-miR-188-5p | NEK6 | NM_001166170 |
| hsa-miR-188-5p | NEK6 | NM_001166171 |
| hsa-miR-188-5p | NEK6 | NM_014397 |
| hsa-miR-188-5p | NIPBL | NM_015384 |
| hsa-miR-188-5p | NTM | NM_001048209 |
| hsa-miR-188-5p | NTM | NM_001144058 |
| hsa-miR-188-5p | NTM | NM_016522 |
| hsa-miR-188-5p | PCMT1 | NM_005389 |
| hsa-miR-188-5p | PPP6R3 | NM_001164160 |
| hsa-miR-188-5p | PPP6R3 | NM_001164161 |
| hsa-miR-188-5p | PPP6R3 | NM_001164162 |
| hsa-miR-188-5p | PPP6R3 | NM_001164163 |
| hsa-miR-188-5p | PPP6R3 | NM_018312 |
| hsa-miR-188-5p | PTEN | NM_000314 |
| hsa-miR-188-5p | RIMKLA | NM_173642 |
| hsa-miR-188-5p | SEMA4B | NM_020210 |
| hsa-miR-188-5p | SEMA4B | NM_198925 |
| hsa-miR-188-5p | SLC30A7 | NM_133496 |
| hsa-miR-188-5p | SLITRK4 | NM_001184749 |
| hsa-miR-188-5p | SLITRK4 | NM_001184750 |
| hsa-miR-188-5p | SLITRK4 | NM_173078 |
| hsa-miR-188-5p | SOS1 | NM_005633 |
| hsa-miR-188-5p | SPRED1 | NM_152594 |
| hsa-miR-188-5p | SRSF7 | NM_001031684 |
| hsa-miR-188-5p | SRSF7 | NM_001195446 |
| hsa-miR-188-5p | TFEC | NM_001018058 |
| hsa-miR-188-5p | TFEC | NM_012252 |
| hsa-miR-188-5p | ZBTB44 | NM_014155 |
| hsa-miR-188-5p | ZNF281 | NM_012482 |
| hsa-miR-188-5p | ZNF660 | NM_173658 |
| hsa-miR-18a-5p | ABTB2 | NM_145804 |
| hsa-miR-18a-5p | ADAT2 | NM_182503 |
| hsa-miR-18a-5p | ADD3 | NM_001121 |
| hsa-miR-18a-5p | ADD3 | NM_016824 |
| hsa-miR-18a-5p | ADD3 | NM_019903 |
| hsa-miR-18a-5p | AKR1D1 | NM_001190906 |
| hsa-miR-18a-5p | AKR1D1 | NM_001190907 |
| hsa-miR-18a-5p | AKR1D1 | NM_005989 |
| hsa-miR-18a-5p | ALCAM | NM_001627 |
| hsa-miR-18a-5p | ANKRD50 | NM_001167882 |
| hsa-miR-18a-5p | ANKRD50 | NM_020337 |
| hsa-miR-18a-5p | ATP8A1 | NM_001105529 |
| hsa-miR-18a-5p | ATP8A1 | NM_006095 |
| hsa-miR-18a-5p | BBX | NM_001142568 |
| hsa-miR-18a-5p | BBX | NM_020235 |
| hsa-miR-18a-5p | BEND3 | NM_001080450 |
| hsa-miR-18a-5p | BHLHE22 | NM_152414 |
| hsa-miR-18a-5p | BRWD1 | NM_018963 |
| hsa-miR-18a-5p | BRWD1 | NM_033656 |
| hsa-miR-18a-5p | BTG3 | NM_001130914 |
| hsa-miR-18a-5p | BTG3 | NM_006806 |
| hsa-miR-18a-5p | C1orf21 | NM_030806 |
| hsa-miR-18a-5p | CACNB3 | NM_000725 |
| hsa-miR-18a-5p | CACNB3 | NM_001206915 |
| hsa-miR-18a-5p | CACNB3 | NM_001206916 |
| hsa-miR-18a-5p | CACNB3 | NM_001206917 |
| hsa-miR-18a-5p | CADM2 | NM_001167674 |
| hsa-miR-18a-5p | CADM2 | NM_001167675 |
| hsa-miR-18a-5p | CADM2 | NM_153184 |
| hsa-miR-18a-5p | CCDC88A | NM_001135597 |
| hsa-miR-18a-5p | CCDC88A | NM_018084 |
| hsa-miR-18a-5p | CCND2 | NM_001759 |
| hsa-miR-18a-5p | CDH6 | NM_004932 |
| hsa-miR-18a-5p | CDK19 | NM_015076 |
| hsa-miR-18a-5p | CDK2 | NM_001798 |
| hsa-miR-18a-5p | CDK2 | NM_052827 |
| hsa-miR-18a-5p | CREBL2 | NM_001310 |
| hsa-miR-18a-5p | CSRNP3 | NM_001172173 |
| hsa-miR-18a-5p | CSRNP3 | NM_024969 |
| hsa-miR-18a-5p | CTDSPL | NM_001008392 |
| hsa-miR-18a-5p | CTDSPL | NM_005808 |
| hsa-miR-18a-5p | CTGF | NM_001901 |
| hsa-miR-18a-5p | DAAM2 | NM_001201427 |
| hsa-miR-18a-5p | DAAM2 | NM_015345 |
| hsa-miR-18a-5p | DDX18 | NM_006773 |
| hsa-miR-18a-5p | DICER1 | NM_001195573 |
| hsa-miR-18a-5p | DICER1 | NM_030621 |
| hsa-miR-18a-5p | DICER1 | NM_177438 |
| hsa-miR-18a-5p | DIRAS2 | NM_017594 |
| hsa-miR-18a-5p | ENDOD1 | NM_015036 |
| hsa-miR-18a-5p | ERI1 | NM_153332 |
| hsa-miR-18a-5p | ESR1 | NM_000125 |
| hsa-miR-18a-5p | ESR1 | NM_001122740 |
| hsa-miR-18a-5p | ESR1 | NM_001122741 |
| hsa-miR-18a-5p | ESR1 | NM_001122742 |
| hsa-miR-18a-5p | ETV6 | NM_001987 |
| hsa-miR-18a-5p | F3 | NM_001178096 |
| hsa-miR-18a-5p | F3 | NM_001993 |
| hsa-miR-18a-5p | FAM136A | NM_032822 |
| hsa-miR-18a-5p | FAM3C | NM_001040020 |
| hsa-miR-18a-5p | FAM3C | NM_014888 |
| hsa-miR-18a-5p | FBXL3 | NM_012158 |
| hsa-miR-18a-5p | FCHSD2 | NM_014824 |
| hsa-miR-18a-5p | FGF1 | NM_000800 |
| hsa-miR-18a-5p | FGF1 | NM_001144892 |
| hsa-miR-18a-5p | FGF1 | NM_001144934 |
| hsa-miR-18a-5p | FGF1 | NM_001144935 |
| hsa-miR-18a-5p | FGF1 | NM_033136 |
| hsa-miR-18a-5p | FGF1 | NM_033137 |
| hsa-miR-18a-5p | FNBP1 | NM_015033 |
| hsa-miR-18a-5p | FNDC3B | NM_001135095 |
| hsa-miR-18a-5p | FNDC3B | NM_022763 |
| hsa-miR-18a-5p | FOXH1 | NM_003923 |
| hsa-miR-18a-5p | FRS2 | NM_001042555 |
| hsa-miR-18a-5p | FRS2 | NM_006654 |
| hsa-miR-18a-5p | GIGYF1 | NM_022574 |
| hsa-miR-18a-5p | GNPDA2 | NM_138335 |
| hsa-miR-18a-5p | HEATR5A | NM_015473 |
| hsa-miR-18a-5p | HSF5 | NM_001080439 |
| hsa-miR-18a-5p | HTR1D | NM_000864 |
| hsa-miR-18a-5p | INADL | NM_176877 |
| hsa-miR-18a-5p | INO80D | NM_017759 |
| hsa-miR-18a-5p | IRF2 | NM_002199 |
| hsa-miR-18a-5p | ISM1 | NM_080826 |
| hsa-miR-18a-5p | JAKMIP3 | NM_001105521 |
| hsa-miR-18a-5p | KCMF1 | NM_020122 |
| hsa-miR-18a-5p | KCNA1 | NM_000217 |
| hsa-miR-18a-5p | KDM2A | NM_012308 |
| hsa-miR-18a-5p | KDM5B | NM_006618 |
| hsa-miR-18a-5p | KIAA0513 | NM_014732 |
| hsa-miR-18a-5p | LIF | NM_002309 |
| hsa-miR-18a-5p | LIN28A | NM_024674 |
| hsa-miR-18a-5p | LIN54 | NM_001115007 |
| hsa-miR-18a-5p | LIN54 | NM_001115008 |
| hsa-miR-18a-5p | LIN54 | NM_194282 |
| hsa-miR-18a-5p | LMO4 | NM_006769 |
| hsa-miR-18a-5p | LRRFIP1 | NM_001137552 |
| hsa-miR-18a-5p | LRRFIP1 | NM_001137553 |
| hsa-miR-18a-5p | LRRFIP1 | NM_004735 |
| hsa-miR-18a-5p | MBNL1 | NM_021038 |
| hsa-miR-18a-5p | MBNL1 | NM_207292 |
| hsa-miR-18a-5p | MBNL1 | NM_207293 |
| hsa-miR-18a-5p | MBNL1 | NM_207294 |
| hsa-miR-18a-5p | MBNL1 | NM_207295 |
| hsa-miR-18a-5p | MBNL1 | NM_207296 |
| hsa-miR-18a-5p | MBNL1 | NM_207297 |
| hsa-miR-18a-5p | MDGA1 | NM_153487 |
| hsa-miR-18a-5p | MYLK | NM_053025 |
| hsa-miR-18a-5p | MYLK | NM_053026 |
| hsa-miR-18a-5p | MYLK | NM_053027 |
| hsa-miR-18a-5p | MYLK | NM_053028 |
| hsa-miR-18a-5p | MYLK | NM_053031 |
| hsa-miR-18a-5p | MYLK | NM_053032 |
| hsa-miR-18a-5p | NDFIP1 | NM_030571 |
| hsa-miR-18a-5p | NEDD4 | NM_006154 |
| hsa-miR-18a-5p | NEDD4 | NM_198400 |
| hsa-miR-18a-5p | NEDD9 | NM_001142393 |
| hsa-miR-18a-5p | NEDD9 | NM_006403 |
| hsa-miR-18a-5p | NEO1 | NM_001172623 |
| hsa-miR-18a-5p | NEO1 | NM_001172624 |
| hsa-miR-18a-5p | NEO1 | NM_002499 |
| hsa-miR-18a-5p | NOS1 | NM_000620 |
| hsa-miR-18a-5p | NOS1 | NM_001204213 |
| hsa-miR-18a-5p | NOS1 | NM_001204214 |
| hsa-miR-18a-5p | NOS1 | NM_001204218 |
| hsa-miR-18a-5p | NOTCH2 | NM_024408 |
| hsa-miR-18a-5p | NR1H2 | NM_007121 |
| hsa-miR-18a-5p | ORAI3 | NM_152288 |
| hsa-miR-18a-5p | OTX1 | NM_001199770 |
| hsa-miR-18a-5p | OTX1 | NM_014562 |
| hsa-miR-18a-5p | PARD6B | NM_032521 |
| hsa-miR-18a-5p | PDE4D | NM_001104631 |
| hsa-miR-18a-5p | PDE4D | NM_001165899 |
| hsa-miR-18a-5p | PDE4D | NM_001197218 |
| hsa-miR-18a-5p | PDE4D | NM_001197219 |
| hsa-miR-18a-5p | PDE4D | NM_001197220 |
| hsa-miR-18a-5p | PDE4D | NM_001197221 |
| hsa-miR-18a-5p | PDE4D | NM_001197222 |
| hsa-miR-18a-5p | PDE4D | NM_001197223 |
| hsa-miR-18a-5p | PDE4D | NM_006203 |
| hsa-miR-18a-5p | PHC3 | NM_024947 |
| hsa-miR-18a-5p | PHF2 | NM_005392 |
| hsa-miR-18a-5p | PKNOX1 | NM_004571 |
| hsa-miR-18a-5p | PSD3 | NM_015310 |
| hsa-miR-18a-5p | PSD3 | NM_206909 |
| hsa-miR-18a-5p | PTGFRN | NM_020440 |
| hsa-miR-18a-5p | PTP4A3 | NM_007079 |
| hsa-miR-18a-5p | PTP4A3 | NM_032611 |
| hsa-miR-18a-5p | RAB11FIP2 | NM_014904 |
| hsa-miR-18a-5p | RABGAP1 | NM_012197 |
| hsa-miR-18a-5p | RIMKLA | NM_173642 |
| hsa-miR-18a-5p | SAR1A | NM_001142648 |
| hsa-miR-18a-5p | SAR1A | NM_020150 |
| hsa-miR-18a-5p | SEC14L3 | NM_174975 |
| hsa-miR-18a-5p | SH3BP4 | NM_014521 |
| hsa-miR-18a-5p | SH3KBP1 | NM_001024666 |
| hsa-miR-18a-5p | SH3KBP1 | NM_001184960 |
| hsa-miR-18a-5p | SH3KBP1 | NM_031892 |
| hsa-miR-18a-5p | SIK3 | NM_025164 |
| hsa-miR-18a-5p | SIM2 | NM_005069 |
| hsa-miR-18a-5p | SLC12A6 | NM_001042494 |
| hsa-miR-18a-5p | SLC12A6 | NM_001042495 |
| hsa-miR-18a-5p | SLC12A6 | NM_001042496 |
| hsa-miR-18a-5p | SLC12A6 | NM_001042497 |
| hsa-miR-18a-5p | SLC12A6 | NM_005135 |
| hsa-miR-18a-5p | SLC12A6 | NM_133647 |
| hsa-miR-18a-5p | SLC50A1 | NM_001122837 |
| hsa-miR-18a-5p | SLC50A1 | NM_001122839 |
| hsa-miR-18a-5p | SLC50A1 | NM_018845 |
| hsa-miR-18a-5p | SMAD2 | NM_001003652 |
| hsa-miR-18a-5p | SMAD2 | NM_001135937 |
| hsa-miR-18a-5p | SMAD2 | NM_005901 |
| hsa-miR-18a-5p | SMARCC2 | NM_001130420 |
| hsa-miR-18a-5p | SMARCC2 | NM_003075 |
| hsa-miR-18a-5p | SMARCC2 | NM_139067 |
| hsa-miR-18a-5p | SNRK | NM_001100594 |
| hsa-miR-18a-5p | SNRK | NM_017719 |
| hsa-miR-18a-5p | SORBS2 | NM_001145670 |
| hsa-miR-18a-5p | SORBS2 | NM_001145671 |
| hsa-miR-18a-5p | SORBS2 | NM_001145672 |
| hsa-miR-18a-5p | SORBS2 | NM_001145673 |
| hsa-miR-18a-5p | SORBS2 | NM_001145674 |
| hsa-miR-18a-5p | SORBS2 | NM_001145675 |
| hsa-miR-18a-5p | SORBS2 | NM_003603 |
| hsa-miR-18a-5p | SORBS2 | NM_021069 |
| hsa-miR-18a-5p | SOX6 | NM_001145811 |
| hsa-miR-18a-5p | SOX6 | NM_001145819 |
| hsa-miR-18a-5p | SOX6 | NM_017508 |
| hsa-miR-18a-5p | SOX6 | NM_033326 |
| hsa-miR-18a-5p | TAOK1 | NM_020791 |
| hsa-miR-18a-5p | TAOK1 | NM_025142 |
| hsa-miR-18a-5p | TBPL1 | NM_004865 |
| hsa-miR-18a-5p | THBD | NM_000361 |
| hsa-miR-18a-5p | THBS1 | NM_003246 |
| hsa-miR-18a-5p | TMEM170B | NM_001100829 |
| hsa-miR-18a-5p | TMEM2 | NM_001135820 |
| hsa-miR-18a-5p | TMEM2 | NM_013390 |
| hsa-miR-18a-5p | TNFAIP3 | NM_006290 |
| hsa-miR-18a-5p | TNRC6B | NM_001024843 |
| hsa-miR-18a-5p | TNRC6B | NM_001162501 |
| hsa-miR-18a-5p | TNRC6B | NM_015088 |
| hsa-miR-18a-5p | TPM3 | NM_001043351 |
| hsa-miR-18a-5p | TPM3 | NM_153649 |
| hsa-miR-18a-5p | TRAPPC8 | NM_014939 |
| hsa-miR-18a-5p | TRIB2 | NM_021643 |
| hsa-miR-18a-5p | TRIM2 | NM_001130067 |
| hsa-miR-18a-5p | TRIM2 | NM_015271 |
| hsa-miR-18a-5p | TTPAL | NM_001039199 |
| hsa-miR-18a-5p | TTPAL | NM_024331 |
| hsa-miR-18a-5p | TWF1 | NM_001242397 |
| hsa-miR-18a-5p | TWF1 | NM_002822 |
| hsa-miR-18a-5p | UBE2Z | NM_023079 |
| hsa-miR-18a-5p | UBTD2 | NM_152277 |
| hsa-miR-18a-5p | UQCRB | NM_001199975 |
| hsa-miR-18a-5p | UQCRB | NM_006294 |
| hsa-miR-18a-5p | VMA21 | NM_001017980 |
| hsa-miR-18a-5p | XYLT1 | NM_022166 |
| hsa-miR-18a-5p | ZBTB47 | NM_145166 |
| hsa-miR-18a-5p | ZCCHC3 | NM_033089 |
| hsa-miR-18a-5p | ZCCHC4 | NM_024936 |
| hsa-miR-18a-5p | ZNF367 | NM_153695 |
| hsa-miR-18a-5p | ZSCAN30 | NM_001112734 |
| hsa-miR-18a-5p | ZSCAN30 | NM_001166012 |
| hsa-miR-18b-5p | ABTB2 | NM_145804 |
| hsa-miR-18b-5p | ADAT2 | NM_182503 |
| hsa-miR-18b-5p | ADD3 | NM_001121 |
| hsa-miR-18b-5p | ADD3 | NM_016824 |
| hsa-miR-18b-5p | ADD3 | NM_019903 |
| hsa-miR-18b-5p | AKR1D1 | NM_001190906 |
| hsa-miR-18b-5p | AKR1D1 | NM_001190907 |
| hsa-miR-18b-5p | AKR1D1 | NM_005989 |
| hsa-miR-18b-5p | ALCAM | NM_001627 |
| hsa-miR-18b-5p | ANKRD50 | NM_001167882 |
| hsa-miR-18b-5p | ANKRD50 | NM_020337 |
| hsa-miR-18b-5p | ATM | NM_000051 |
| hsa-miR-18b-5p | BBX | NM_001142568 |
| hsa-miR-18b-5p | BBX | NM_020235 |
| hsa-miR-18b-5p | BHLHE22 | NM_152414 |
| hsa-miR-18b-5p | C1orf21 | NM_030806 |
| hsa-miR-18b-5p | CACNB3 | NM_000725 |
| hsa-miR-18b-5p | CACNB3 | NM_001206915 |
| hsa-miR-18b-5p | CACNB3 | NM_001206916 |
| hsa-miR-18b-5p | CACNB3 | NM_001206917 |
| hsa-miR-18b-5p | CADM2 | NM_001167674 |
| hsa-miR-18b-5p | CADM2 | NM_001167675 |
| hsa-miR-18b-5p | CADM2 | NM_153184 |
| hsa-miR-18b-5p | CCND2 | NM_001759 |
| hsa-miR-18b-5p | CDH6 | NM_004932 |
| hsa-miR-18b-5p | CDK19 | NM_015076 |
| hsa-miR-18b-5p | CDK2 | NM_001798 |
| hsa-miR-18b-5p | CDK2 | NM_052827 |
| hsa-miR-18b-5p | CLCC1 | NM_001048210 |
| hsa-miR-18b-5p | CLCC1 | NM_015127 |
| hsa-miR-18b-5p | CREBL2 | NM_001310 |
| hsa-miR-18b-5p | CRIM1 | NM_016441 |
| hsa-miR-18b-5p | CSRNP3 | NM_001172173 |
| hsa-miR-18b-5p | CSRNP3 | NM_024969 |
| hsa-miR-18b-5p | CTDSPL | NM_001008392 |
| hsa-miR-18b-5p | CTDSPL | NM_005808 |
| hsa-miR-18b-5p | CTGF | NM_001901 |
| hsa-miR-18b-5p | DAAM2 | NM_001201427 |
| hsa-miR-18b-5p | DAAM2 | NM_015345 |
| hsa-miR-18b-5p | DDX18 | NM_006773 |
| hsa-miR-18b-5p | DICER1 | NM_001195573 |
| hsa-miR-18b-5p | DICER1 | NM_030621 |
| hsa-miR-18b-5p | DICER1 | NM_177438 |
| hsa-miR-18b-5p | ENDOD1 | NM_015036 |
| hsa-miR-18b-5p | ERI1 | NM_153332 |
| hsa-miR-18b-5p | ESR1 | NM_000125 |
| hsa-miR-18b-5p | ESR1 | NM_001122740 |
| hsa-miR-18b-5p | ESR1 | NM_001122741 |
| hsa-miR-18b-5p | ESR1 | NM_001122742 |
| hsa-miR-18b-5p | ETV6 | NM_001987 |
| hsa-miR-18b-5p | F3 | NM_001178096 |
| hsa-miR-18b-5p | F3 | NM_001993 |
| hsa-miR-18b-5p | FAM136A | NM_032822 |
| hsa-miR-18b-5p | FAM3C | NM_001040020 |
| hsa-miR-18b-5p | FAM3C | NM_014888 |
| hsa-miR-18b-5p | FBXL3 | NM_012158 |
| hsa-miR-18b-5p | FGF1 | NM_000800 |
| hsa-miR-18b-5p | FGF1 | NM_001144892 |
| hsa-miR-18b-5p | FGF1 | NM_001144934 |
| hsa-miR-18b-5p | FGF1 | NM_001144935 |
| hsa-miR-18b-5p | FGF1 | NM_033136 |
| hsa-miR-18b-5p | FGF1 | NM_033137 |
| hsa-miR-18b-5p | FNBP1 | NM_015033 |
| hsa-miR-18b-5p | FNDC3B | NM_001135095 |
| hsa-miR-18b-5p | FNDC3B | NM_022763 |
| hsa-miR-18b-5p | FRS2 | NM_001042555 |
| hsa-miR-18b-5p | FRS2 | NM_006654 |
| hsa-miR-18b-5p | GIGYF1 | NM_022574 |
| hsa-miR-18b-5p | GNPDA2 | NM_138335 |
| hsa-miR-18b-5p | HEATR5A | NM_015473 |
| hsa-miR-18b-5p | INADL | NM_176877 |
| hsa-miR-18b-5p | INO80D | NM_017759 |
| hsa-miR-18b-5p | ISM1 | NM_080826 |
| hsa-miR-18b-5p | KCNA1 | NM_000217 |
| hsa-miR-18b-5p | KDM2A | NM_012308 |
| hsa-miR-18b-5p | KDM5B | NM_006618 |
| hsa-miR-18b-5p | KIAA0513 | NM_014732 |
| hsa-miR-18b-5p | LIF | NM_002309 |
| hsa-miR-18b-5p | LIN28A | NM_024674 |
| hsa-miR-18b-5p | LIN54 | NM_001115007 |
| hsa-miR-18b-5p | LIN54 | NM_001115008 |
| hsa-miR-18b-5p | LIN54 | NM_194282 |
| hsa-miR-18b-5p | LMO4 | NM_006769 |
| hsa-miR-18b-5p | LRRFIP1 | NM_001137552 |
| hsa-miR-18b-5p | LRRFIP1 | NM_001137553 |
| hsa-miR-18b-5p | LRRFIP1 | NM_004735 |
| hsa-miR-18b-5p | MAP3K1 | NM_005921 |
| hsa-miR-18b-5p | MBNL1 | NM_021038 |
| hsa-miR-18b-5p | MBNL1 | NM_207292 |
| hsa-miR-18b-5p | MBNL1 | NM_207293 |
| hsa-miR-18b-5p | MBNL1 | NM_207294 |
| hsa-miR-18b-5p | MBNL1 | NM_207295 |
| hsa-miR-18b-5p | MBNL1 | NM_207296 |
| hsa-miR-18b-5p | MBNL1 | NM_207297 |
| hsa-miR-18b-5p | MDGA1 | NM_153487 |
| hsa-miR-18b-5p | MYLK | NM_053025 |
| hsa-miR-18b-5p | MYLK | NM_053026 |
| hsa-miR-18b-5p | MYLK | NM_053027 |
| hsa-miR-18b-5p | MYLK | NM_053028 |
| hsa-miR-18b-5p | MYLK | NM_053031 |
| hsa-miR-18b-5p | MYLK | NM_053032 |
| hsa-miR-18b-5p | NACC1 | NM_052876 |
| hsa-miR-18b-5p | NEDD4 | NM_006154 |
| hsa-miR-18b-5p | NEDD4 | NM_198400 |
| hsa-miR-18b-5p | NEDD9 | NM_001142393 |
| hsa-miR-18b-5p | NEDD9 | NM_006403 |
| hsa-miR-18b-5p | NEO1 | NM_001172623 |
| hsa-miR-18b-5p | NEO1 | NM_001172624 |
| hsa-miR-18b-5p | NEO1 | NM_002499 |
| hsa-miR-18b-5p | NOS1 | NM_000620 |
| hsa-miR-18b-5p | NOS1 | NM_001204213 |
| hsa-miR-18b-5p | NOS1 | NM_001204214 |
| hsa-miR-18b-5p | NOS1 | NM_001204218 |
| hsa-miR-18b-5p | NOTCH2 | NM_024408 |
| hsa-miR-18b-5p | NR1H2 | NM_007121 |
| hsa-miR-18b-5p | OCRL | NM_000276 |
| hsa-miR-18b-5p | OCRL | NM_001587 |
| hsa-miR-18b-5p | ORAI3 | NM_152288 |
| hsa-miR-18b-5p | OSBP2 | NM_030758 |
| hsa-miR-18b-5p | OTX1 | NM_001199770 |
| hsa-miR-18b-5p | OTX1 | NM_014562 |
| hsa-miR-18b-5p | PARD6B | NM_032521 |
| hsa-miR-18b-5p | PDE4D | NM_001104631 |
| hsa-miR-18b-5p | PDE4D | NM_001165899 |
| hsa-miR-18b-5p | PDE4D | NM_001197218 |
| hsa-miR-18b-5p | PDE4D | NM_001197219 |
| hsa-miR-18b-5p | PDE4D | NM_001197220 |
| hsa-miR-18b-5p | PDE4D | NM_001197221 |
| hsa-miR-18b-5p | PDE4D | NM_001197222 |
| hsa-miR-18b-5p | PDE4D | NM_001197223 |
| hsa-miR-18b-5p | PDE4D | NM_006203 |
| hsa-miR-18b-5p | PHC3 | NM_024947 |
| hsa-miR-18b-5p | PHF19 | NM_015651 |
| hsa-miR-18b-5p | PHF2 | NM_005392 |
| hsa-miR-18b-5p | PSD3 | NM_015310 |
| hsa-miR-18b-5p | PSD3 | NM_206909 |
| hsa-miR-18b-5p | PTGFRN | NM_020440 |
| hsa-miR-18b-5p | PTP4A3 | NM_007079 |
| hsa-miR-18b-5p | PTP4A3 | NM_032611 |
| hsa-miR-18b-5p | RAB11FIP2 | NM_014904 |
| hsa-miR-18b-5p | RAB22A | NM_020673 |
| hsa-miR-18b-5p | RGS4 | NM_001102445 |
| hsa-miR-18b-5p | RGS4 | NM_001113380 |
| hsa-miR-18b-5p | RGS4 | NM_001113381 |
| hsa-miR-18b-5p | RGS4 | NM_005613 |
| hsa-miR-18b-5p | RIMKLA | NM_173642 |
| hsa-miR-18b-5p | SAR1A | NM_001142648 |
| hsa-miR-18b-5p | SAR1A | NM_020150 |
| hsa-miR-18b-5p | SH3BP4 | NM_014521 |
| hsa-miR-18b-5p | SH3KBP1 | NM_001024666 |
| hsa-miR-18b-5p | SH3KBP1 | NM_001184960 |
| hsa-miR-18b-5p | SH3KBP1 | NM_031892 |
| hsa-miR-18b-5p | SIK3 | NM_025164 |
| hsa-miR-18b-5p | SIM2 | NM_005069 |
| hsa-miR-18b-5p | SLC12A6 | NM_001042494 |
| hsa-miR-18b-5p | SLC12A6 | NM_001042495 |
| hsa-miR-18b-5p | SLC12A6 | NM_001042496 |
| hsa-miR-18b-5p | SLC12A6 | NM_001042497 |
| hsa-miR-18b-5p | SLC12A6 | NM_005135 |
| hsa-miR-18b-5p | SLC12A6 | NM_133647 |
| hsa-miR-18b-5p | SLC50A1 | NM_001122837 |
| hsa-miR-18b-5p | SLC50A1 | NM_001122839 |
| hsa-miR-18b-5p | SLC50A1 | NM_018845 |
| hsa-miR-18b-5p | SNRK | NM_001100594 |
| hsa-miR-18b-5p | SNRK | NM_017719 |
| hsa-miR-18b-5p | SORBS2 | NM_001145670 |
| hsa-miR-18b-5p | SORBS2 | NM_001145671 |
| hsa-miR-18b-5p | SORBS2 | NM_001145672 |
| hsa-miR-18b-5p | SORBS2 | NM_001145673 |
| hsa-miR-18b-5p | SORBS2 | NM_001145674 |
| hsa-miR-18b-5p | SORBS2 | NM_001145675 |
| hsa-miR-18b-5p | SORBS2 | NM_003603 |
| hsa-miR-18b-5p | SORBS2 | NM_021069 |
| hsa-miR-18b-5p | SOX6 | NM_001145811 |
| hsa-miR-18b-5p | SOX6 | NM_001145819 |
| hsa-miR-18b-5p | SOX6 | NM_017508 |
| hsa-miR-18b-5p | SOX6 | NM_033326 |
| hsa-miR-18b-5p | SYNCRIP | NM_001159673 |
| hsa-miR-18b-5p | SYNCRIP | NM_001159674 |
| hsa-miR-18b-5p | SYNCRIP | NM_001159676 |
| hsa-miR-18b-5p | SYNCRIP | NM_001159677 |
| hsa-miR-18b-5p | TAOK1 | NM_020791 |
| hsa-miR-18b-5p | TAOK1 | NM_025142 |
| hsa-miR-18b-5p | THBD | NM_000361 |
| hsa-miR-18b-5p | THBS1 | NM_003246 |
| hsa-miR-18b-5p | TMEM170B | NM_001100829 |
| hsa-miR-18b-5p | TMEM2 | NM_001135820 |
| hsa-miR-18b-5p | TMEM2 | NM_013390 |
| hsa-miR-18b-5p | TNFAIP3 | NM_006290 |
| hsa-miR-18b-5p | TNRC6B | NM_001024843 |
| hsa-miR-18b-5p | TNRC6B | NM_001162501 |
| hsa-miR-18b-5p | TNRC6B | NM_015088 |
| hsa-miR-18b-5p | TPM3 | NM_001043351 |
| hsa-miR-18b-5p | TPM3 | NM_153649 |
| hsa-miR-18b-5p | TRAPPC8 | NM_014939 |
| hsa-miR-18b-5p | TRIM2 | NM_001130067 |
| hsa-miR-18b-5p | TRIM2 | NM_015271 |
| hsa-miR-18b-5p | TTPAL | NM_001039199 |
| hsa-miR-18b-5p | TTPAL | NM_024331 |
| hsa-miR-18b-5p | TWF1 | NM_001242397 |
| hsa-miR-18b-5p | TWF1 | NM_002822 |
| hsa-miR-18b-5p | UBE2Z | NM_023079 |
| hsa-miR-18b-5p | UBTD2 | NM_152277 |
| hsa-miR-18b-5p | UQCRB | NM_001199975 |
| hsa-miR-18b-5p | UQCRB | NM_006294 |
| hsa-miR-18b-5p | USP24 | NM_015306 |
| hsa-miR-18b-5p | VMA21 | NM_001017980 |
| hsa-miR-18b-5p | XYLT1 | NM_022166 |
| hsa-miR-18b-5p | YWHAB | NM_003404 |
| hsa-miR-18b-5p | YWHAB | NM_139323 |
| hsa-miR-18b-5p | ZBTB47 | NM_145166 |
| hsa-miR-18b-5p | ZCCHC3 | NM_033089 |
| hsa-miR-18b-5p | ZCCHC4 | NM_024936 |
| hsa-miR-18b-5p | ZNF367 | NM_153695 |
| hsa-miR-18b-5p | ZSCAN30 | NM_001112734 |
| hsa-miR-18b-5p | ZSCAN30 | NM_001166012 |
| hsa-miR-193b-3p | ABI2 | NM_005759 |
| hsa-miR-193b-3p | ADAMTSL3 | NM_207517 |
| hsa-miR-193b-3p | ARHGEF15 | NM_025014 |
| hsa-miR-193b-3p | ARHGEF15 | NM_173728 |
| hsa-miR-193b-3p | ATOH8 | NM_032827 |
| hsa-miR-193b-3p | ATXN1 | NM_000332 |
| hsa-miR-193b-3p | ATXN1 | NM_001128164 |
| hsa-miR-193b-3p | BAZ2A | NM_013449 |
| hsa-miR-193b-3p | BRPF1 | NM_001003694 |
| hsa-miR-193b-3p | BRPF1 | NM_004634 |
| hsa-miR-193b-3p | CALM1 | NM_006888 |
| hsa-miR-193b-3p | CALU | NM_001130674 |
| hsa-miR-193b-3p | CALU | NM_001199671 |
| hsa-miR-193b-3p | CALU | NM_001199672 |
| hsa-miR-193b-3p | CALU | NM_001199673 |
| hsa-miR-193b-3p | CALU | NM_001219 |
| hsa-miR-193b-3p | CBX7 | NM_175709 |
| hsa-miR-193b-3p | CNOT6 | NM_015455 |
| hsa-miR-193b-3p | DENND5B | NM_144973 |
| hsa-miR-193b-3p | DMXL2 | NM_001174116 |
| hsa-miR-193b-3p | DMXL2 | NM_001174117 |
| hsa-miR-193b-3p | DMXL2 | NM_015263 |
| hsa-miR-193b-3p | EN2 | NM_001427 |
| hsa-miR-193b-3p | ETV6 | NM_001987 |
| hsa-miR-193b-3p | GSG1L | NM_001109763 |
| hsa-miR-193b-3p | GSG1L | NM_144675 |
| hsa-miR-193b-3p | HEG1 | NM_020733 |
| hsa-miR-193b-3p | HYOU1 | NM_001130991 |
| hsa-miR-193b-3p | HYOU1 | NM_006389 |
| hsa-miR-193b-3p | IGSF3 | NM_001007237 |
| hsa-miR-193b-3p | IGSF3 | NM_001542 |
| hsa-miR-193b-3p | IL17RD | NM_017563 |
| hsa-miR-193b-3p | INO80D | NM_017759 |
| hsa-miR-193b-3p | KCNJ2 | NM_000891 |
| hsa-miR-193b-3p | KCTD7 | NM_001167961 |
| hsa-miR-193b-3p | KCTD7 | NM_153033 |
| hsa-miR-193b-3p | KIAA0825 | NM_001145678 |
| hsa-miR-193b-3p | KLHL15 | NM_030624 |
| hsa-miR-193b-3p | KRAS | NM_004985 |
| hsa-miR-193b-3p | KRAS | NM_033360 |
| hsa-miR-193b-3p | LAMC1 | NM_002293 |
| hsa-miR-193b-3p | LAMP2 | NM_013995 |
| hsa-miR-193b-3p | MAP3K3 | NM_002401 |
| hsa-miR-193b-3p | MAP3K3 | NM_203351 |
| hsa-miR-193b-3p | MBNL3 | NM_001170701 |
| hsa-miR-193b-3p | MBNL3 | NM_001170702 |
| hsa-miR-193b-3p | MBNL3 | NM_001170703 |
| hsa-miR-193b-3p | MBNL3 | NM_001170704 |
| hsa-miR-193b-3p | MBNL3 | NM_018388 |
| hsa-miR-193b-3p | MBNL3 | NM_133486 |
| hsa-miR-193b-3p | MGAT3 | NM_001098270 |
| hsa-miR-193b-3p | MGAT3 | NM_002409 |
| hsa-miR-193b-3p | MMP14 | NM_004995 |
| hsa-miR-193b-3p | MMP16 | NM_005941 |
| hsa-miR-193b-3p | N4BP3 | NM_015111 |
| hsa-miR-193b-3p | NPAS4 | NM_178864 |
| hsa-miR-193b-3p | NSF | NM_006178 |
| hsa-miR-193b-3p | OSMR | NM_003999 |
| hsa-miR-193b-3p | PAIP2B | NM_020459 |
| hsa-miR-193b-3p | PIGA | NM_002641 |
| hsa-miR-193b-3p | PIGA | NM_020473 |
| hsa-miR-193b-3p | PLAG1 | NM_001114634 |
| hsa-miR-193b-3p | PLAG1 | NM_001114635 |
| hsa-miR-193b-3p | PLAG1 | NM_002655 |
| hsa-miR-193b-3p | PLAU | NM_001145031 |
| hsa-miR-193b-3p | PLAU | NM_002658 |
| hsa-miR-193b-3p | PRR14L | NM_173566 |
| hsa-miR-193b-3p | RFT1 | NM_052859 |
| hsa-miR-193b-3p | RNF103 | NM_001198952 |
| hsa-miR-193b-3p | RUNX1T1 | NM_001198625 |
| hsa-miR-193b-3p | RUNX1T1 | NM_001198626 |
| hsa-miR-193b-3p | RUNX1T1 | NM_001198627 |
| hsa-miR-193b-3p | RUNX1T1 | NM_001198628 |
| hsa-miR-193b-3p | RUNX1T1 | NM_001198629 |
| hsa-miR-193b-3p | RUNX1T1 | NM_001198630 |
| hsa-miR-193b-3p | RUNX1T1 | NM_001198631 |
| hsa-miR-193b-3p | RUNX1T1 | NM_001198632 |
| hsa-miR-193b-3p | RUNX1T1 | NM_001198633 |
| hsa-miR-193b-3p | RUNX1T1 | NM_001198634 |
| hsa-miR-193b-3p | RUNX1T1 | NM_001198679 |
| hsa-miR-193b-3p | RUNX1T1 | NM_004349 |
| hsa-miR-193b-3p | RUNX1T1 | NM_175634 |
| hsa-miR-193b-3p | RUNX1T1 | NM_175635 |
| hsa-miR-193b-3p | RUNX1T1 | NM_175636 |
| hsa-miR-193b-3p | SEPN1 | NM_020451 |
| hsa-miR-193b-3p | SEPN1 | NM_206926 |
| hsa-miR-193b-3p | SIX4 | NM_017420 |
| hsa-miR-193b-3p | SLC10A6 | NM_197965 |
| hsa-miR-193b-3p | SLC15A1 | NM_005073 |
| hsa-miR-193b-3p | SLC23A2 | NM_005116 |
| hsa-miR-193b-3p | SLC23A2 | NM_203327 |
| hsa-miR-193b-3p | SLC39A5 | NM_001135195 |
| hsa-miR-193b-3p | SLC39A5 | NM_173596 |
| hsa-miR-193b-3p | SPOPL | NM_001001664 |
| hsa-miR-193b-3p | SRSF6 | NM_006275 |
| hsa-miR-193b-3p | ST6GALNAC3 | NM_152996 |
| hsa-miR-193b-3p | STMN1 | NM_005563 |
| hsa-miR-193b-3p | STMN1 | NM_203399 |
| hsa-miR-193b-3p | STMN1 | NM_203401 |
| hsa-miR-193b-3p | STX16 | NM_001001433 |
| hsa-miR-193b-3p | STX16 | NM_001134772 |
| hsa-miR-193b-3p | STX16 | NM_001134773 |
| hsa-miR-193b-3p | STX16 | NM_001204868 |
| hsa-miR-193b-3p | STX16 | NM_003763 |
| hsa-miR-193b-3p | TGFB2 | NM_001135599 |
| hsa-miR-193b-3p | TGFB2 | NM_003238 |
| hsa-miR-193b-3p | TSPYL5 | NM_033512 |
| hsa-miR-193b-3p | WDR82 | NM_025222 |
| hsa-miR-193b-3p | YWHAZ | NM_001135699 |
| hsa-miR-193b-3p | YWHAZ | NM_001135700 |
| hsa-miR-193b-3p | YWHAZ | NM_001135701 |
| hsa-miR-193b-3p | YWHAZ | NM_001135702 |
| hsa-miR-193b-3p | YWHAZ | NM_003406 |
| hsa-miR-193b-3p | YWHAZ | NM_145690 |
| hsa-miR-193b-3p | ZNF365 | NM_014951 |
| hsa-miR-19a-3p | ABHD5 | NM_016006 |
| hsa-miR-19a-3p | ACBD5 | NM_001042473 |
| hsa-miR-19a-3p | ACBD5 | NM_145698 |
| hsa-miR-19a-3p | ADAM12 | NM_003474 |
| hsa-miR-19a-3p | AFF1 | NM_001166693 |
| hsa-miR-19a-3p | AFF1 | NM_005935 |
| hsa-miR-19a-3p | ALG2 | NM_033087 |
| hsa-miR-19a-3p | ANKRD34A | NM_001039888 |
| hsa-miR-19a-3p | ANXA7 | NM_001156 |
| hsa-miR-19a-3p | ANXA7 | NM_004034 |
| hsa-miR-19a-3p | ARHGEF26 | NM_015595 |
| hsa-miR-19a-3p | ASNA1 | NM_004317 |
| hsa-miR-19a-3p | ASXL2 | NM_018263 |
| hsa-miR-19a-3p | ATP2C1 | NM_001199179 |
| hsa-miR-19a-3p | ATP2C1 | NM_001199181 |
| hsa-miR-19a-3p | ATP2C1 | NM_001199184 |
| hsa-miR-19a-3p | ATP2C1 | NM_014382 |
| hsa-miR-19a-3p | ATXN1 | NM_000332 |
| hsa-miR-19a-3p | ATXN1 | NM_001128164 |
| hsa-miR-19a-3p | ATXN1L | NM_001137675 |
| hsa-miR-19a-3p | B3GALNT2 | NM_152490 |
| hsa-miR-19a-3p | BCL3 | NM_005178 |
| hsa-miR-19a-3p | BEND4 | NM_001159547 |
| hsa-miR-19a-3p | BEND4 | NM_207406 |
| hsa-miR-19a-3p | BRWD3 | NM_153252 |
| hsa-miR-19a-3p | BTF3L4 | NM_001136497 |
| hsa-miR-19a-3p | BTF3L4 | NM_152265 |
| hsa-miR-19a-3p | C11orf96 | NM_001145033 |
| hsa-miR-19a-3p | C2orf42 | NM_017880 |
| hsa-miR-19a-3p | CACNA1C | NM_000719 |
| hsa-miR-19a-3p | CACNA1C | NM_001129827 |
| hsa-miR-19a-3p | CACNA1C | NM_001129829 |
| hsa-miR-19a-3p | CACNA1C | NM_001129830 |
| hsa-miR-19a-3p | CACNA1C | NM_001129831 |
| hsa-miR-19a-3p | CACNA1C | NM_001129832 |
| hsa-miR-19a-3p | CACNA1C | NM_001129833 |
| hsa-miR-19a-3p | CACNA1C | NM_001129834 |
| hsa-miR-19a-3p | CACNA1C | NM_001129835 |
| hsa-miR-19a-3p | CACNA1C | NM_001129836 |
| hsa-miR-19a-3p | CACNA1C | NM_001129837 |
| hsa-miR-19a-3p | CACNA1C | NM_001129838 |
| hsa-miR-19a-3p | CACNA1C | NM_001129839 |
| hsa-miR-19a-3p | CACNA1C | NM_001129840 |
| hsa-miR-19a-3p | CACNA1C | NM_001129841 |
| hsa-miR-19a-3p | CACNA1C | NM_001129842 |
| hsa-miR-19a-3p | CACNA1C | NM_001129843 |
| hsa-miR-19a-3p | CACNA1C | NM_001129844 |
| hsa-miR-19a-3p | CACNA1C | NM_001129846 |
| hsa-miR-19a-3p | CACNA1C | NM_001167623 |
| hsa-miR-19a-3p | CACNA1C | NM_001167624 |
| hsa-miR-19a-3p | CACNA1C | NM_001167625 |
| hsa-miR-19a-3p | CACNA1C | NM_199460 |
| hsa-miR-19a-3p | CAMTA1 | NM_015215 |
| hsa-miR-19a-3p | CARD10 | NM_014550 |
| hsa-miR-19a-3p | CBFB | NM_001755 |
| hsa-miR-19a-3p | CBFB | NM_022845 |
| hsa-miR-19a-3p | CBX1 | NM_001127228 |
| hsa-miR-19a-3p | CBX1 | NM_006807 |
| hsa-miR-19a-3p | CCDC126 | NM_138771 |
| hsa-miR-19a-3p | CDC42BPA | NM_003607 |
| hsa-miR-19a-3p | CDC42BPA | NM_014826 |
| hsa-miR-19a-3p | CEP350 | NM_014810 |
| hsa-miR-19a-3p | CLOCK | NM_004898 |
| hsa-miR-19a-3p | CNGA3 | NM_001079878 |
| hsa-miR-19a-3p | CNGA3 | NM_001298 |
| hsa-miR-19a-3p | CNKSR2 | NM_001168647 |
| hsa-miR-19a-3p | CNKSR2 | NM_014927 |
| hsa-miR-19a-3p | CNOT4 | NM_001008225 |
| hsa-miR-19a-3p | CNOT4 | NM_001190848 |
| hsa-miR-19a-3p | CPEB3 | NM_001178137 |
| hsa-miR-19a-3p | CPEB3 | NM_014912 |
| hsa-miR-19a-3p | CR2 | NM_001006658 |
| hsa-miR-19a-3p | CR2 | NM_001877 |
| hsa-miR-19a-3p | CSPP1 | NM_024790 |
| hsa-miR-19a-3p | CUX1 | NM_001202544 |
| hsa-miR-19a-3p | CUX1 | NM_001202545 |
| hsa-miR-19a-3p | CUX1 | NM_001202546 |
| hsa-miR-19a-3p | CUX1 | NM_001913 |
| hsa-miR-19a-3p | CUX1 | NM_181500 |
| hsa-miR-19a-3p | DBT | NM_001918 |
| hsa-miR-19a-3p | DDHD1 | NM_001160147 |
| hsa-miR-19a-3p | DDHD1 | NM_001160148 |
| hsa-miR-19a-3p | DDHD1 | NM_030637 |
| hsa-miR-19a-3p | DDX6 | NM_004397 |
| hsa-miR-19a-3p | DGKG | NM_001080744 |
| hsa-miR-19a-3p | DGKG | NM_001080745 |
| hsa-miR-19a-3p | DGKG | NM_001346 |
| hsa-miR-19a-3p | DHX40 | NM_001166301 |
| hsa-miR-19a-3p | DHX40 | NM_024612 |
| hsa-miR-19a-3p | DIP2A | NM_001146116 |
| hsa-miR-19a-3p | DIP2A | NM_015151 |
| hsa-miR-19a-3p | DLC1 | NM_001164271 |
| hsa-miR-19a-3p | DLC1 | NM_006094 |
| hsa-miR-19a-3p | DLC1 | NM_182643 |
| hsa-miR-19a-3p | DSEL | NM_032160 |
| hsa-miR-19a-3p | EHBP1 | NM_001142614 |
| hsa-miR-19a-3p | EHBP1 | NM_001142615 |
| hsa-miR-19a-3p | EHBP1 | NM_001142616 |
| hsa-miR-19a-3p | EHBP1 | NM_015252 |
| hsa-miR-19a-3p | ELOVL5 | NM_001242828 |
| hsa-miR-19a-3p | ELOVL5 | NM_001242830 |
| hsa-miR-19a-3p | ELOVL5 | NM_021814 |
| hsa-miR-19a-3p | ENPP5 | NM_021572 |
| hsa-miR-19a-3p | EPG5 | NM_020964 |
| hsa-miR-19a-3p | ETV5 | NM_004454 |
| hsa-miR-19a-3p | FAM160A1 | NM_001109977 |
| hsa-miR-19a-3p | FAM69A | NM_001006605 |
| hsa-miR-19a-3p | FAM83F | NM_138435 |
| hsa-miR-19a-3p | FASTK | NM_006712 |
| hsa-miR-19a-3p | FASTK | NM_033015 |
| hsa-miR-19a-3p | FBXO36 | NM_174899 |
| hsa-miR-19a-3p | FIGNL2 | NM_001013690 |
| hsa-miR-19a-3p | FKBP15 | NM_015258 |
| hsa-miR-19a-3p | FLNC | NM_001127487 |
| hsa-miR-19a-3p | FLNC | NM_001458 |
| hsa-miR-19a-3p | FNDC3B | NM_001135095 |
| hsa-miR-19a-3p | FNDC3B | NM_022763 |
| hsa-miR-19a-3p | FOXD4L1 | NM_012184 |
| hsa-miR-19a-3p | FRAS1 | NM_025074 |
| hsa-miR-19a-3p | FRMD6 | NM_001042481 |
| hsa-miR-19a-3p | FRMD6 | NM_152330 |
| hsa-miR-19a-3p | FUT4 | NM_002033 |
| hsa-miR-19a-3p | GLTP | NM_016433 |
| hsa-miR-19a-3p | GPCPD1 | NM_019593 |
| hsa-miR-19a-3p | GPRC5B | NM_016235 |
| hsa-miR-19a-3p | GULP1 | NM_016315 |
| hsa-miR-19a-3p | HCFC2 | NM_013320 |
| hsa-miR-19a-3p | HIP1 | NM_005338 |
| hsa-miR-19a-3p | HIPK3 | NM_001048200 |
| hsa-miR-19a-3p | HIPK3 | NM_005734 |
| hsa-miR-19a-3p | HNRNPUL1 | NM_007040 |
| hsa-miR-19a-3p | HNRNPUL1 | NM_144732 |
| hsa-miR-19a-3p | IL6ST | NM_001190981 |
| hsa-miR-19a-3p | IL6ST | NM_002184 |
| hsa-miR-19a-3p | IMPDH1 | NM_000883 |
| hsa-miR-19a-3p | IMPDH1 | NM_001102605 |
| hsa-miR-19a-3p | IMPDH1 | NM_001142573 |
| hsa-miR-19a-3p | IMPDH1 | NM_001142574 |
| hsa-miR-19a-3p | IMPDH1 | NM_001142575 |
| hsa-miR-19a-3p | IMPDH1 | NM_001142576 |
| hsa-miR-19a-3p | IMPDH1 | NM_183243 |
| hsa-miR-19a-3p | ITPRIPL2 | NM_001034841 |
| hsa-miR-19a-3p | IVNS1ABP | NM_006469 |
| hsa-miR-19a-3p | JAZF1 | NM_175061 |
| hsa-miR-19a-3p | KCNA4 | NM_002233 |
| hsa-miR-19a-3p | KCNJ2 | NM_000891 |
| hsa-miR-19a-3p | KIAA1211 | NM_020722 |
| hsa-miR-19a-3p | KIAA1598 | NM_001127211 |
| hsa-miR-19a-3p | KIAA1598 | NM_018330 |
| hsa-miR-19a-3p | KIAA2013 | NM_138346 |
| hsa-miR-19a-3p | KLF13 | NM_015995 |
| hsa-miR-19a-3p | KPNA3 | NM_002267 |
| hsa-miR-19a-3p | L3MBTL3 | NM_001007102 |
| hsa-miR-19a-3p | L3MBTL3 | NM_032438 |
| hsa-miR-19a-3p | LIMCH1 | NM_001112717 |
| hsa-miR-19a-3p | LIMCH1 | NM_001112718 |
| hsa-miR-19a-3p | LIMCH1 | NM_001112719 |
| hsa-miR-19a-3p | LIMCH1 | NM_001112720 |
| hsa-miR-19a-3p | LIMCH1 | NM_014988 |
| hsa-miR-19a-3p | MAP2K3 | NM_002756 |
| hsa-miR-19a-3p | MAP2K3 | NM_145109 |
| hsa-miR-19a-3p | MAP4K3 | NM_003618 |
| hsa-miR-19a-3p | MAPK14 | NM_001315 |
| hsa-miR-19a-3p | MAPK14 | NM_139012 |
| hsa-miR-19a-3p | MAPK14 | NM_139014 |
| hsa-miR-19a-3p | MAPK6 | NM_002748 |
| hsa-miR-19a-3p | MBD4 | NM_003925 |
| hsa-miR-19a-3p | MDFIC | NM_199072 |
| hsa-miR-19a-3p | MFAP3L | NM_001009554 |
| hsa-miR-19a-3p | MFAP3L | NM_021647 |
| hsa-miR-19a-3p | MID1IP1 | NM_001098790 |
| hsa-miR-19a-3p | MID1IP1 | NM_001098791 |
| hsa-miR-19a-3p | MID1IP1 | NM_021242 |
| hsa-miR-19a-3p | MIER1 | NM_001077700 |
| hsa-miR-19a-3p | MIER1 | NM_001077701 |
| hsa-miR-19a-3p | MIER1 | NM_001077702 |
| hsa-miR-19a-3p | MIER1 | NM_001077703 |
| hsa-miR-19a-3p | MIER1 | NM_001077704 |
| hsa-miR-19a-3p | MIER1 | NM_001146110 |
| hsa-miR-19a-3p | MIER1 | NM_001146111 |
| hsa-miR-19a-3p | MIER1 | NM_001146112 |
| hsa-miR-19a-3p | MIER1 | NM_001146113 |
| hsa-miR-19a-3p | MIER1 | NM_020948 |
| hsa-miR-19a-3p | MPPED2 | NM_001584 |
| hsa-miR-19a-3p | NF1 | NM_000267 |
| hsa-miR-19a-3p | NF1 | NM_001042492 |
| hsa-miR-19a-3p | NME7 | NM_013330 |
| hsa-miR-19a-3p | NME7 | NM_197972 |
| hsa-miR-19a-3p | NUDT13 | NM_015901 |
| hsa-miR-19a-3p | PARM1 | NM_015393 |
| hsa-miR-19a-3p | PCDH10 | NM_032961 |
| hsa-miR-19a-3p | PGM2L1 | NM_173582 |
| hsa-miR-19a-3p | PHF13 | NM_153812 |
| hsa-miR-19a-3p | PMEPA1 | NM_020182 |
| hsa-miR-19a-3p | PMEPA1 | NM_199169 |
| hsa-miR-19a-3p | PMEPA1 | NM_199170 |
| hsa-miR-19a-3p | PMEPA1 | NM_199171 |
| hsa-miR-19a-3p | PPFIA2 | NM_001220473 |
| hsa-miR-19a-3p | PPFIA2 | NM_001220474 |
| hsa-miR-19a-3p | PPFIA2 | NM_001220475 |
| hsa-miR-19a-3p | PPFIA2 | NM_001220476 |
| hsa-miR-19a-3p | PPFIA2 | NM_001220477 |
| hsa-miR-19a-3p | PPFIA2 | NM_001220478 |
| hsa-miR-19a-3p | PPFIA2 | NM_001220479 |
| hsa-miR-19a-3p | PPFIA2 | NM_001220480 |
| hsa-miR-19a-3p | PPFIA2 | NM_003625 |
| hsa-miR-19a-3p | PRICKLE2 | NM_198859 |
| hsa-miR-19a-3p | PRKAA1 | NM_006251 |
| hsa-miR-19a-3p | PRKAA1 | NM_206907 |
| hsa-miR-19a-3p | PRR14L | NM_173566 |
| hsa-miR-19a-3p | PRR5L | NM_001160167 |
| hsa-miR-19a-3p | PRR5L | NM_001160168 |
| hsa-miR-19a-3p | PRR5L | NM_001160169 |
| hsa-miR-19a-3p | PRR5L | NM_024841 |
| hsa-miR-19a-3p | PTEN | NM_000314 |
| hsa-miR-19a-3p | PTGES3 | NM_006601 |
| hsa-miR-19a-3p | PTPRD | NM_001040712 |
| hsa-miR-19a-3p | PTPRD | NM_001171025 |
| hsa-miR-19a-3p | PTPRD | NM_002839 |
| hsa-miR-19a-3p | PTPRD | NM_130391 |
| hsa-miR-19a-3p | PTPRD | NM_130392 |
| hsa-miR-19a-3p | PTPRD | NM_130393 |
| hsa-miR-19a-3p | QKI | NM_206854 |
| hsa-miR-19a-3p | RAB2B | NM_001163380 |
| hsa-miR-19a-3p | RAB2B | NM_032846 |
| hsa-miR-19a-3p | RAP1A | NM_001010935 |
| hsa-miR-19a-3p | RAP1A | NM_002884 |
| hsa-miR-19a-3p | RDH12 | NM_152443 |
| hsa-miR-19a-3p | RHEBL1 | NM_144593 |
| hsa-miR-19a-3p | RICTOR | NM_152756 |
| hsa-miR-19a-3p | RNF11 | NM_014372 |
| hsa-miR-19a-3p | RNF111 | NM_017610 |
| hsa-miR-19a-3p | RORA | NM_002943 |
| hsa-miR-19a-3p | RORA | NM_134260 |
| hsa-miR-19a-3p | RORA | NM_134261 |
| hsa-miR-19a-3p | RORA | NM_134262 |
| hsa-miR-19a-3p | RPAP3 | NM_001146075 |
| hsa-miR-19a-3p | RPAP3 | NM_001146076 |
| hsa-miR-19a-3p | RPAP3 | NM_024604 |
| hsa-miR-19a-3p | RPS6KA2 | NM_001006932 |
| hsa-miR-19a-3p | RPS6KA2 | NM_021135 |
| hsa-miR-19a-3p | SAMD4A | NM_001161576 |
| hsa-miR-19a-3p | SAMD4A | NM_001161577 |
| hsa-miR-19a-3p | SAMD4A | NM_015589 |
| hsa-miR-19a-3p | SCN1B | NM_001037 |
| hsa-miR-19a-3p | SEC63 | NM_007214 |
| hsa-miR-19a-3p | SEMA4C | NM_017789 |
| hsa-miR-19a-3p | SGCD | NM_000337 |
| hsa-miR-19a-3p | SGCD | NM_001128209 |
| hsa-miR-19a-3p | SH3KBP1 | NM_001024666 |
| hsa-miR-19a-3p | SH3KBP1 | NM_001184960 |
| hsa-miR-19a-3p | SH3KBP1 | NM_031892 |
| hsa-miR-19a-3p | SHC3 | NM_016848 |
| hsa-miR-19a-3p | SIK3 | NM_025164 |
| hsa-miR-19a-3p | SLC25A34 | NM_207348 |
| hsa-miR-19a-3p | SLC30A7 | NM_001144884 |
| hsa-miR-19a-3p | SLC30A7 | NM_133496 |
| hsa-miR-19a-3p | SLC6A8 | NM_001142805 |
| hsa-miR-19a-3p | SLC6A8 | NM_001142806 |
| hsa-miR-19a-3p | SLC6A8 | NM_005629 |
| hsa-miR-19a-3p | SLC9A6 | NM_001042537 |
| hsa-miR-19a-3p | SLC9A6 | NM_001177651 |
| hsa-miR-19a-3p | SLC9A6 | NM_006359 |
| hsa-miR-19a-3p | SMOC2 | NM_001166412 |
| hsa-miR-19a-3p | SMOC2 | NM_022138 |
| hsa-miR-19a-3p | SNX18 | NM_001102575 |
| hsa-miR-19a-3p | SNX18 | NM_001145427 |
| hsa-miR-19a-3p | SOX4 | NM_003107 |
| hsa-miR-19a-3p | SPHK2 | NM_001204158 |
| hsa-miR-19a-3p | SPHK2 | NM_001204159 |
| hsa-miR-19a-3p | SPHK2 | NM_001204160 |
| hsa-miR-19a-3p | SPHK2 | NM_020126 |
| hsa-miR-19a-3p | SPOCK1 | NM_004598 |
| hsa-miR-19a-3p | SPRYD3 | NM_032840 |
| hsa-miR-19a-3p | SUZ12 | NM_015355 |
| hsa-miR-19a-3p | SYBU | NM_001099743 |
| hsa-miR-19a-3p | SYBU | NM_001099744 |
| hsa-miR-19a-3p | SYBU | NM_001099745 |
| hsa-miR-19a-3p | SYBU | NM_001099746 |
| hsa-miR-19a-3p | SYBU | NM_001099747 |
| hsa-miR-19a-3p | SYBU | NM_001099748 |
| hsa-miR-19a-3p | SYBU | NM_001099749 |
| hsa-miR-19a-3p | SYBU | NM_001099750 |
| hsa-miR-19a-3p | SYBU | NM_001099751 |
| hsa-miR-19a-3p | SYBU | NM_001099752 |
| hsa-miR-19a-3p | SYBU | NM_001099753 |
| hsa-miR-19a-3p | SYBU | NM_001099754 |
| hsa-miR-19a-3p | SYBU | NM_001099755 |
| hsa-miR-19a-3p | SYBU | NM_001099756 |
| hsa-miR-19a-3p | SYBU | NM_017786 |
| hsa-miR-19a-3p | SYT1 | NM_001135805 |
| hsa-miR-19a-3p | SYT1 | NM_001135806 |
| hsa-miR-19a-3p | SYT1 | NM_005639 |
| hsa-miR-19a-3p | TFDP2 | NM_001178138 |
| hsa-miR-19a-3p | TFDP2 | NM_001178139 |
| hsa-miR-19a-3p | TFDP2 | NM_001178140 |
| hsa-miR-19a-3p | TFDP2 | NM_001178141 |
| hsa-miR-19a-3p | TFDP2 | NM_001178142 |
| hsa-miR-19a-3p | TFDP2 | NM_006286 |
| hsa-miR-19a-3p | TFPI | NM_006287 |
| hsa-miR-19a-3p | TMEM167B | NM_020141 |
| hsa-miR-19a-3p | TMEM196 | NM_152774 |
| hsa-miR-19a-3p | TNFAIP3 | NM_006290 |
| hsa-miR-19a-3p | TNFRSF12A | NM_016639 |
| hsa-miR-19a-3p | TNIP1 | NM_006058 |
| hsa-miR-19a-3p | TNRC6B | NM_001024843 |
| hsa-miR-19a-3p | TNRC6B | NM_001162501 |
| hsa-miR-19a-3p | TNRC6B | NM_015088 |
| hsa-miR-19a-3p | TOR1B | NM_014506 |
| hsa-miR-19a-3p | TP53INP1 | NM_001135733 |
| hsa-miR-19a-3p | TP53INP1 | NM_033285 |
| hsa-miR-19a-3p | TRAK2 | NM_015049 |
| hsa-miR-19a-3p | TUB | NM_003320 |
| hsa-miR-19a-3p | TUB | NM_177972 |
| hsa-miR-19a-3p | TXK | NM_003328 |
| hsa-miR-19a-3p | UBAP2L | NM_001127320 |
| hsa-miR-19a-3p | UBFD1 | NM_019116 |
| hsa-miR-19a-3p | USP6 | NM_004505 |
| hsa-miR-19a-3p | VAMP1 | NM_014231 |
| hsa-miR-19a-3p | VAMP1 | NM_199245 |
| hsa-miR-19a-3p | VAPA | NM_003574 |
| hsa-miR-19a-3p | VAPA | NM_194434 |
| hsa-miR-19a-3p | VGLL4 | NM_001128219 |
| hsa-miR-19a-3p | VGLL4 | NM_001128220 |
| hsa-miR-19a-3p | VGLL4 | NM_001128221 |
| hsa-miR-19a-3p | VGLL4 | NM_014667 |
| hsa-miR-19a-3p | VPS53 | NM_001128159 |
| hsa-miR-19a-3p | VSIG10L | NM_001163922 |
| hsa-miR-19a-3p | WIPF3 | NM_001080529 |
| hsa-miR-19a-3p | WNT1 | NM_005430 |
| hsa-miR-19a-3p | ZBTB11 | NM_014415 |
| hsa-miR-19a-3p | ZDHHC7 | NM_001145548 |
| hsa-miR-19a-3p | ZDHHC7 | NM_017740 |
| hsa-miR-19a-3p | ZFPM2 | NM_012082 |
| hsa-miR-19a-3p | ZHX3 | NM_015035 |
| hsa-miR-19a-3p | ZMYND11 | NM_001202464 |
| hsa-miR-19a-3p | ZMYND11 | NM_001202465 |
| hsa-miR-19a-3p | ZMYND11 | NM_001202466 |
| hsa-miR-19a-3p | ZMYND11 | NM_006624 |
| hsa-miR-19a-3p | ZNF217 | NM_006526 |
| hsa-miR-19a-3p | ZNF469 | NM_001127464 |
| hsa-miR-19a-3p | ZNF831 | NM_178457 |
| hsa-miR-19a-3p | ZNF862 | NM_001099220 |
| hsa-miR-19a-3p | ZPLD1 | NM_175056 |
| hsa-miR-19a-3p | ZYG11B | NM_024646 |
| hsa-miR-200a-3p | ABL2 | NM_001136000 |
| hsa-miR-200a-3p | ABL2 | NM_001168236 |
| hsa-miR-200a-3p | ABL2 | NM_001168237 |
| hsa-miR-200a-3p | ABL2 | NM_001168238 |
| hsa-miR-200a-3p | ABL2 | NM_001168239 |
| hsa-miR-200a-3p | ABL2 | NM_005158 |
| hsa-miR-200a-3p | ABL2 | NM_007314 |
| hsa-miR-200a-3p | ACOT7 | NM_007274 |
| hsa-miR-200a-3p | ACOT7 | NM_181864 |
| hsa-miR-200a-3p | ACOT7 | NM_181865 |
| hsa-miR-200a-3p | ACOT7 | NM_181866 |
| hsa-miR-200a-3p | ADD3 | NM_001121 |
| hsa-miR-200a-3p | ADD3 | NM_016824 |
| hsa-miR-200a-3p | ADD3 | NM_019903 |
| hsa-miR-200a-3p | ADRB1 | NM_000684 |
| hsa-miR-200a-3p | AKAP11 | NM_016248 |
| hsa-miR-200a-3p | ANP32E | NM_001136478 |
| hsa-miR-200a-3p | ANP32E | NM_001136479 |
| hsa-miR-200a-3p | ANP32E | NM_030920 |
| hsa-miR-200a-3p | APBB2 | NM_001166050 |
| hsa-miR-200a-3p | APBB2 | NM_001166052 |
| hsa-miR-200a-3p | APBB2 | NM_001166054 |
| hsa-miR-200a-3p | APBB2 | NM_004307 |
| hsa-miR-200a-3p | APBB2 | NM_173075 |
| hsa-miR-200a-3p | ARHGAP23 | NM_001199417 |
| hsa-miR-200a-3p | ARHGEF18 | NM_001130955 |
| hsa-miR-200a-3p | ARHGEF18 | NM_015318 |
| hsa-miR-200a-3p | ASXL1 | NM_015338 |
| hsa-miR-200a-3p | ATP8A1 | NM_001105529 |
| hsa-miR-200a-3p | ATP8A1 | NM_006095 |
| hsa-miR-200a-3p | ATP8A2 | NM_016529 |
| hsa-miR-200a-3p | ATRN | NM_139321 |
| hsa-miR-200a-3p | ATXN7L1 | NM_020725 |
| hsa-miR-200a-3p | ATXN7L1 | NM_138495 |
| hsa-miR-200a-3p | B3GNT5 | NM_032047 |
| hsa-miR-200a-3p | C11orf95 | NM_001144936 |
| hsa-miR-200a-3p | C1GALT1 | NM_020156 |
| hsa-miR-200a-3p | C1orf21 | NM_030806 |
| hsa-miR-200a-3p | CACNA1B | NM_000718 |
| hsa-miR-200a-3p | CALCR | NM_001164737 |
| hsa-miR-200a-3p | CALCR | NM_001164738 |
| hsa-miR-200a-3p | CALCR | NM_001742 |
| hsa-miR-200a-3p | CBL | NM_005188 |
| hsa-miR-200a-3p | CCDC6 | NM_005436 |
| hsa-miR-200a-3p | CCND2 | NM_001759 |
| hsa-miR-200a-3p | CCPG1 | NM_004748 |
| hsa-miR-200a-3p | CCPG1 | NM_020739 |
| hsa-miR-200a-3p | CDC14A | NM_003672 |
| hsa-miR-200a-3p | CDC42EP3 | NM_006449 |
| hsa-miR-200a-3p | CHD2 | NM_001271 |
| hsa-miR-200a-3p | CHD9 | NM_025134 |
| hsa-miR-200a-3p | CLOCK | NM_004898 |
| hsa-miR-200a-3p | CRLF3 | NM_015986 |
| hsa-miR-200a-3p | CTBP2 | NM_001083914 |
| hsa-miR-200a-3p | CTBP2 | NM_001329 |
| hsa-miR-200a-3p | CTBP2 | NM_022802 |
| hsa-miR-200a-3p | CUL3 | NM_003590 |
| hsa-miR-200a-3p | CYP26B1 | NM_019885 |
| hsa-miR-200a-3p | DDIT4L | NM_145244 |
| hsa-miR-200a-3p | DDX5 | NM_004396 |
| hsa-miR-200a-3p | DIP2B | NM_173602 |
| hsa-miR-200a-3p | DLC1 | NM_001164271 |
| hsa-miR-200a-3p | DLC1 | NM_006094 |
| hsa-miR-200a-3p | DLC1 | NM_182643 |
| hsa-miR-200a-3p | DNAJC13 | NM_015268 |
| hsa-miR-200a-3p | DR1 | NM_001938 |
| hsa-miR-200a-3p | DTL | NM_016448 |
| hsa-miR-200a-3p | DUSP3 | NM_004090 |
| hsa-miR-200a-3p | E2F3 | NM_001949 |
| hsa-miR-200a-3p | EDEM1 | NM_014674 |
| hsa-miR-200a-3p | ELAVL2 | NM_001171195 |
| hsa-miR-200a-3p | ELAVL2 | NM_001171197 |
| hsa-miR-200a-3p | ELAVL2 | NM_004432 |
| hsa-miR-200a-3p | ELMOD1 | NM_001130037 |
| hsa-miR-200a-3p | ELMOD1 | NM_018712 |
| hsa-miR-200a-3p | EPN1 | NM_001130071 |
| hsa-miR-200a-3p | EPN1 | NM_001130072 |
| hsa-miR-200a-3p | EPN1 | NM_013333 |
| hsa-miR-200a-3p | ERG | NM_001136154 |
| hsa-miR-200a-3p | ERG | NM_001136155 |
| hsa-miR-200a-3p | ERG | NM_004449 |
| hsa-miR-200a-3p | ERG | NM_182918 |
| hsa-miR-200a-3p | EVI5L | NM_001159944 |
| hsa-miR-200a-3p | EVI5L | NM_145245 |
| hsa-miR-200a-3p | EXOC5 | NM_006544 |
| hsa-miR-200a-3p | FAM118B | NM_024556 |
| hsa-miR-200a-3p | FAM160B1 | NM_020940 |
| hsa-miR-200a-3p | FAM168B | NM_001009993 |
| hsa-miR-200a-3p | FAM49A | NM_030797 |
| hsa-miR-200a-3p | FBXW2 | NM_012164 |
| hsa-miR-200a-3p | FKBP5 | NM_001145775 |
| hsa-miR-200a-3p | FKBP5 | NM_001145776 |
| hsa-miR-200a-3p | FKBP5 | NM_004117 |
| hsa-miR-200a-3p | FOXA1 | NM_004496 |
| hsa-miR-200a-3p | FOXC1 | NM_001453 |
| hsa-miR-200a-3p | FOXJ1 | NM_001454 |
| hsa-miR-200a-3p | FOXJ3 | NM_001198850 |
| hsa-miR-200a-3p | FOXJ3 | NM_001198851 |
| hsa-miR-200a-3p | FOXJ3 | NM_001198852 |
| hsa-miR-200a-3p | FOXJ3 | NM_014947 |
| hsa-miR-200a-3p | FOXN3 | NM_001085471 |
| hsa-miR-200a-3p | FOXN3 | NM_005197 |
| hsa-miR-200a-3p | FOXP1 | NM_032682 |
| hsa-miR-200a-3p | FRMD4A | NM_018027 |
| hsa-miR-200a-3p | FSD1L | NM_001145313 |
| hsa-miR-200a-3p | GAB1 | NM_002039 |
| hsa-miR-200a-3p | GAB1 | NM_207123 |
| hsa-miR-200a-3p | GATA6 | NM_005257 |
| hsa-miR-200a-3p | GJC1 | NM_001080383 |
| hsa-miR-200a-3p | GJC1 | NM_005497 |
| hsa-miR-200a-3p | GLCCI1 | NM_138426 |
| hsa-miR-200a-3p | GLRX | NM_002064 |
| hsa-miR-200a-3p | GPR137C | NM_001099652 |
| hsa-miR-200a-3p | GPR6 | NM_005284 |
| hsa-miR-200a-3p | HCAR2 | NM_177551 |
| hsa-miR-200a-3p | HCAR3 | NM_006018 |
| hsa-miR-200a-3p | HCN1 | NM_021072 |
| hsa-miR-200a-3p | HMG20A | NM_018200 |
| hsa-miR-200a-3p | HSPA13 | NM_006948 |
| hsa-miR-200a-3p | IRS2 | NM_003749 |
| hsa-miR-200a-3p | KATNAL1 | NM_001014380 |
| hsa-miR-200a-3p | KATNAL1 | NM_032116 |
| hsa-miR-200a-3p | KCTD20 | NM_173562 |
| hsa-miR-200a-3p | KIF17 | NM_001122819 |
| hsa-miR-200a-3p | KIF17 | NM_020816 |
| hsa-miR-200a-3p | KIF3A | NM_007054 |
| hsa-miR-200a-3p | LAMTOR3 | NM_021970 |
| hsa-miR-200a-3p | LSAMP | NM_002338 |
| hsa-miR-200a-3p | MACC1 | NM_182762 |
| hsa-miR-200a-3p | MAP2K4 | NM_003010 |
| hsa-miR-200a-3p | MAP3K2 | NM_006609 |
| hsa-miR-200a-3p | MIB1 | NM_020774 |
| hsa-miR-200a-3p | MIER1 | NM_001077702 |
| hsa-miR-200a-3p | MIER1 | NM_001077703 |
| hsa-miR-200a-3p | MIER1 | NM_001077704 |
| hsa-miR-200a-3p | MIER1 | NM_001146111 |
| hsa-miR-200a-3p | MIER1 | NM_001146113 |
| hsa-miR-200a-3p | MN1 | NM_002430 |
| hsa-miR-200a-3p | MPPED2 | NM_001584 |
| hsa-miR-200a-3p | MYH10 | NM_005964 |
| hsa-miR-200a-3p | MYRIP | NM_015460 |
| hsa-miR-200a-3p | NDFIP2 | NM_001161407 |
| hsa-miR-200a-3p | NDFIP2 | NM_019080 |
| hsa-miR-200a-3p | NEK6 | NM_001145001 |
| hsa-miR-200a-3p | NEK6 | NM_001166167 |
| hsa-miR-200a-3p | NEK6 | NM_001166168 |
| hsa-miR-200a-3p | NEK6 | NM_001166169 |
| hsa-miR-200a-3p | NEK6 | NM_001166170 |
| hsa-miR-200a-3p | NEK6 | NM_001166171 |
| hsa-miR-200a-3p | NEK6 | NM_014397 |
| hsa-miR-200a-3p | NME1 | NM_000269 |
| hsa-miR-200a-3p | NME1 | NM_198175 |
| hsa-miR-200a-3p | NRCAM | NM_001037132 |
| hsa-miR-200a-3p | NRCAM | NM_001193582 |
| hsa-miR-200a-3p | NRCAM | NM_001193583 |
| hsa-miR-200a-3p | NRCAM | NM_001193584 |
| hsa-miR-200a-3p | NRCAM | NM_005010 |
| hsa-miR-200a-3p | NRP1 | NM_003873 |
| hsa-miR-200a-3p | OSBPL11 | NM_022776 |
| hsa-miR-200a-3p | OXSR1 | NM_005109 |
| hsa-miR-200a-3p | PANK3 | NM_024594 |
| hsa-miR-200a-3p | PAX3 | NM_001127366 |
| hsa-miR-200a-3p | PAX3 | NM_181458 |
| hsa-miR-200a-3p | PAX3 | NM_181459 |
| hsa-miR-200a-3p | PAX3 | NM_181460 |
| hsa-miR-200a-3p | PAX3 | NM_181461 |
| hsa-miR-200a-3p | PCDH9 | NM_020403 |
| hsa-miR-200a-3p | PCDH9 | NM_203487 |
| hsa-miR-200a-3p | PCGF3 | NM_006315 |
| hsa-miR-200a-3p | PEG3 | NM_001146184 |
| hsa-miR-200a-3p | PEG3 | NM_001146185 |
| hsa-miR-200a-3p | PEG3 | NM_001146186 |
| hsa-miR-200a-3p | PEG3 | NM_001146187 |
| hsa-miR-200a-3p | PEG3 | NM_006210 |
| hsa-miR-200a-3p | PHYHIPL | NM_001143774 |
| hsa-miR-200a-3p | PHYHIPL | NM_032439 |
| hsa-miR-200a-3p | PIKFYVE | NM_015040 |
| hsa-miR-200a-3p | PITPNB | NM_012399 |
| hsa-miR-200a-3p | PLAG1 | NM_001114634 |
| hsa-miR-200a-3p | PLAG1 | NM_001114635 |
| hsa-miR-200a-3p | PLAG1 | NM_002655 |
| hsa-miR-200a-3p | PLEKHA8 | NM_001197026 |
| hsa-miR-200a-3p | PLXNA4 | NM_020911 |
| hsa-miR-200a-3p | POT1 | NM_001042594 |
| hsa-miR-200a-3p | POT1 | NM_015450 |
| hsa-miR-200a-3p | PPARA | NM_001001928 |
| hsa-miR-200a-3p | PPARA | NM_005036 |
| hsa-miR-200a-3p | PPM1E | NM_014906 |
| hsa-miR-200a-3p | PRKACB | NM_001242857 |
| hsa-miR-200a-3p | PRKACB | NM_001242858 |
| hsa-miR-200a-3p | PRKACB | NM_001242859 |
| hsa-miR-200a-3p | PRKACB | NM_001242860 |
| hsa-miR-200a-3p | PRKACB | NM_001242861 |
| hsa-miR-200a-3p | PRKACB | NM_001242862 |
| hsa-miR-200a-3p | PRKACB | NM_002731 |
| hsa-miR-200a-3p | PRKACB | NM_182948 |
| hsa-miR-200a-3p | PRKCE | NM_005400 |
| hsa-miR-200a-3p | QSER1 | NM_001076786 |
| hsa-miR-200a-3p | RANBP6 | NM_012416 |
| hsa-miR-200a-3p | RAPGEF5 | NM_012294 |
| hsa-miR-200a-3p | RASSF8 | NM_001164746 |
| hsa-miR-200a-3p | RASSF8 | NM_001164747 |
| hsa-miR-200a-3p | RASSF8 | NM_001164748 |
| hsa-miR-200a-3p | RBM24 | NM_001143941 |
| hsa-miR-200a-3p | RBM24 | NM_001143942 |
| hsa-miR-200a-3p | RBM24 | NM_153020 |
| hsa-miR-200a-3p | RFTN1 | NM_015150 |
| hsa-miR-200a-3p | RNF185 | NM_001135825 |
| hsa-miR-200a-3p | RNF185 | NM_152267 |
| hsa-miR-200a-3p | RUNX1 | NM_001001890 |
| hsa-miR-200a-3p | RUNX1 | NM_001754 |
| hsa-miR-200a-3p | S100PBP | NM_022753 |
| hsa-miR-200a-3p | SAMD8 | NM_001174156 |
| hsa-miR-200a-3p | SAMD8 | NM_144660 |
| hsa-miR-200a-3p | 43716 | NM_001098813 |
| hsa-miR-200a-3p | 43716 | NM_015146 |
| hsa-miR-200a-3p | SIAH1 | NM_001006610 |
| hsa-miR-200a-3p | SIAH1 | NM_003031 |
| hsa-miR-200a-3p | SLC5A3 | NM_006933 |
| hsa-miR-200a-3p | SOX17 | NM_022454 |
| hsa-miR-200a-3p | SOX5 | NM_006940 |
| hsa-miR-200a-3p | SOX5 | NM_152989 |
| hsa-miR-200a-3p | SOX5 | NM_178010 |
| hsa-miR-200a-3p | SPAG9 | NM_001130528 |
| hsa-miR-200a-3p | SPAG9 | NM_003971 |
| hsa-miR-200a-3p | SRCAP | NM_006662 |
| hsa-miR-200a-3p | ST3GAL3 | NM_006279 |
| hsa-miR-200a-3p | ST3GAL3 | NM_174963 |
| hsa-miR-200a-3p | ST3GAL3 | NM_174964 |
| hsa-miR-200a-3p | ST3GAL3 | NM_174965 |
| hsa-miR-200a-3p | ST3GAL3 | NM_174966 |
| hsa-miR-200a-3p | ST3GAL3 | NM_174967 |
| hsa-miR-200a-3p | ST3GAL3 | NM_174968 |
| hsa-miR-200a-3p | ST3GAL3 | NM_174969 |
| hsa-miR-200a-3p | ST3GAL3 | NM_174970 |
| hsa-miR-200a-3p | ST3GAL3 | NM_174971 |
| hsa-miR-200a-3p | ST3GAL5 | NM_001042437 |
| hsa-miR-200a-3p | ST3GAL5 | NM_003896 |
| hsa-miR-200a-3p | STRN | NM_003162 |
| hsa-miR-200a-3p | STX16 | NM_001001433 |
| hsa-miR-200a-3p | STX16 | NM_001134772 |
| hsa-miR-200a-3p | STX16 | NM_001134773 |
| hsa-miR-200a-3p | STX16 | NM_001204868 |
| hsa-miR-200a-3p | STX16 | NM_003763 |
| hsa-miR-200a-3p | STXBP5 | NM_001127715 |
| hsa-miR-200a-3p | STXBP5 | NM_139244 |
| hsa-miR-200a-3p | TADA1 | NM_053053 |
| hsa-miR-200a-3p | TCERG1 | NM_001040006 |
| hsa-miR-200a-3p | TCERG1 | NM_006706 |
| hsa-miR-200a-3p | TET1 | NM_030625 |
| hsa-miR-200a-3p | TM4SF1 | NM_014220 |
| hsa-miR-200a-3p | TMEM110 | NM_198563 |
| hsa-miR-200a-3p | TMEM170B | NM_001100829 |
| hsa-miR-200a-3p | TMEM56 | NM_001199679 |
| hsa-miR-200a-3p | TMEM56 | NM_152487 |
| hsa-miR-200a-3p | TNS1 | NM_022648 |
| hsa-miR-200a-3p | TP53INP1 | NM_001135733 |
| hsa-miR-200a-3p | TP53INP1 | NM_033285 |
| hsa-miR-200a-3p | TRAM1 | NM_014294 |
| hsa-miR-200a-3p | TSC1 | NM_000368 |
| hsa-miR-200a-3p | TSC1 | NM_001162426 |
| hsa-miR-200a-3p | TSC1 | NM_001162427 |
| hsa-miR-200a-3p | TSHZ3 | NM_020856 |
| hsa-miR-200a-3p | TTC28 | NM_001145418 |
| hsa-miR-200a-3p | TTR | NM_000371 |
| hsa-miR-200a-3p | UBA6 | NM_018227 |
| hsa-miR-200a-3p | VCAN | NM_001126336 |
| hsa-miR-200a-3p | VCAN | NM_001164097 |
| hsa-miR-200a-3p | VCAN | NM_001164098 |
| hsa-miR-200a-3p | VCAN | NM_004385 |
| hsa-miR-200a-3p | WAPAL | NM_015045 |
| hsa-miR-200a-3p | WDR81 | NM_001163673 |
| hsa-miR-200a-3p | WDR81 | NM_001163809 |
| hsa-miR-200a-3p | WDR81 | NM_001163811 |
| hsa-miR-200a-3p | WDR81 | NM_152348 |
| hsa-miR-200a-3p | WWTR1 | NM_001168278 |
| hsa-miR-200a-3p | WWTR1 | NM_001168280 |
| hsa-miR-200a-3p | WWTR1 | NM_015472 |
| hsa-miR-200a-3p | YAP1 | NM_001130145 |
| hsa-miR-200a-3p | YAP1 | NM_001195044 |
| hsa-miR-200a-3p | YAP1 | NM_001195045 |
| hsa-miR-200a-3p | YAP1 | NM_006106 |
| hsa-miR-200a-3p | YPEL5 | NM_001127399 |
| hsa-miR-200a-3p | YPEL5 | NM_001127400 |
| hsa-miR-200a-3p | YPEL5 | NM_001127401 |
| hsa-miR-200a-3p | YPEL5 | NM_016061 |
| hsa-miR-200a-3p | YY1 | NM_003403 |
| hsa-miR-200a-3p | ZCCHC24 | NM_153367 |
| hsa-miR-200a-3p | ZDHHC21 | NM_178566 |
| hsa-miR-200a-3p | ZEB1 | NM_001128128 |
| hsa-miR-200a-3p | ZEB1 | NM_001174093 |
| hsa-miR-200a-3p | ZEB1 | NM_001174094 |
| hsa-miR-200a-3p | ZEB1 | NM_001174095 |
| hsa-miR-200a-3p | ZEB1 | NM_001174096 |
| hsa-miR-200a-3p | ZEB1 | NM_030751 |
| hsa-miR-200a-3p | ZEB2 | NM_001171653 |
| hsa-miR-200a-3p | ZEB2 | NM_014795 |
| hsa-miR-200a-3p | ZNF629 | NM_001080417 |
| hsa-miR-200a-3p | ZNF660 | NM_173658 |
| hsa-miR-200c-3p | A1CF | NM_001198818 |
| hsa-miR-200c-3p | A1CF | NM_001198819 |
| hsa-miR-200c-3p | A1CF | NM_001198820 |
| hsa-miR-200c-3p | A1CF | NM_014576 |
| hsa-miR-200c-3p | A1CF | NM_138932 |
| hsa-miR-200c-3p | A1CF | NM_138933 |
| hsa-miR-200c-3p | ABI2 | NM_005759 |
| hsa-miR-200c-3p | ACE2 | NM_021804 |
| hsa-miR-200c-3p | ACVR1C | NM_001111031 |
| hsa-miR-200c-3p | ACVR1C | NM_001111032 |
| hsa-miR-200c-3p | ACVR1C | NM_001111033 |
| hsa-miR-200c-3p | ACVR1C | NM_145259 |
| hsa-miR-200c-3p | ANLN | NM_018685 |
| hsa-miR-200c-3p | ARHGAP19 | NM_001204300 |
| hsa-miR-200c-3p | ARHGAP19 | NM_032900 |
| hsa-miR-200c-3p | ARHGEF3 | NM_001128615 |
| hsa-miR-200c-3p | ARHGEF3 | NM_001128616 |
| hsa-miR-200c-3p | ARHGEF3 | NM_019555 |
| hsa-miR-200c-3p | ASF1A | NM_014034 |
| hsa-miR-200c-3p | ATP2A2 | NM_170665 |
| hsa-miR-200c-3p | ATXN1 | NM_000332 |
| hsa-miR-200c-3p | ATXN1 | NM_001128164 |
| hsa-miR-200c-3p | B3GNT1 | NM_006876 |
| hsa-miR-200c-3p | C22orf39 | NM_173793 |
| hsa-miR-200c-3p | CASR | NM_000388 |
| hsa-miR-200c-3p | CASR | NM_001178065 |
| hsa-miR-200c-3p | CDR2 | NM_001802 |
| hsa-miR-200c-3p | CHD1 | NM_001270 |
| hsa-miR-200c-3p | CHMP5 | NM_016410 |
| hsa-miR-200c-3p | CLIC4 | NM_013943 |
| hsa-miR-200c-3p | COPS8 | NM_006710 |
| hsa-miR-200c-3p | COPS8 | NM_198189 |
| hsa-miR-200c-3p | CUX1 | NM_001202543 |
| hsa-miR-200c-3p | CUX1 | NM_181552 |
| hsa-miR-200c-3p | CYTH1 | NM_017456 |
| hsa-miR-200c-3p | DGKA | NM_001345 |
| hsa-miR-200c-3p | DGKA | NM_201444 |
| hsa-miR-200c-3p | DGKA | NM_201445 |
| hsa-miR-200c-3p | DGKA | NM_201554 |
| hsa-miR-200c-3p | DIXDC1 | NM_001037954 |
| hsa-miR-200c-3p | DIXDC1 | NM_033425 |
| hsa-miR-200c-3p | DLGAP2 | NM_004745 |
| hsa-miR-200c-3p | DNAJC18 | NM_152686 |
| hsa-miR-200c-3p | DOCK4 | NM_014705 |
| hsa-miR-200c-3p | DTNA | NM_001198939 |
| hsa-miR-200c-3p | DTNA | NM_001198940 |
| hsa-miR-200c-3p | DTNA | NM_001198942 |
| hsa-miR-200c-3p | DTNA | NM_001198943 |
| hsa-miR-200c-3p | DTNA | NM_001198944 |
| hsa-miR-200c-3p | DTNA | NM_001390 |
| hsa-miR-200c-3p | DTNA | NM_032975 |
| hsa-miR-200c-3p | DTNA | NM_032980 |
| hsa-miR-200c-3p | DUSP1 | NM_004417 |
| hsa-miR-200c-3p | DZIP1 | NM_014934 |
| hsa-miR-200c-3p | DZIP1 | NM_198968 |
| hsa-miR-200c-3p | EFNA1 | NM_004428 |
| hsa-miR-200c-3p | EFNA1 | NM_182685 |
| hsa-miR-200c-3p | ELAVL2 | NM_001171195 |
| hsa-miR-200c-3p | ELAVL2 | NM_001171197 |
| hsa-miR-200c-3p | ELAVL2 | NM_004432 |
| hsa-miR-200c-3p | ELF2 | NM_006874 |
| hsa-miR-200c-3p | ELF2 | NM_201999 |
| hsa-miR-200c-3p | ERRFI1 | NM_018948 |
| hsa-miR-200c-3p | FAM179B | NM_015091 |
| hsa-miR-200c-3p | FAT3 | NM_001008781 |
| hsa-miR-200c-3p | FEZ2 | NM_001042548 |
| hsa-miR-200c-3p | FEZ2 | NM_005102 |
| hsa-miR-200c-3p | FHOD1 | NM_013241 |
| hsa-miR-200c-3p | FREM1 | NM_001177704 |
| hsa-miR-200c-3p | FREM1 | NM_144966 |
| hsa-miR-200c-3p | GABPA | NM_001197297 |
| hsa-miR-200c-3p | GABPA | NM_002040 |
| hsa-miR-200c-3p | GLCCI1 | NM_138426 |
| hsa-miR-200c-3p | GPATCH8 | NM_001002909 |
| hsa-miR-200c-3p | GPR107 | NM_001136557 |
| hsa-miR-200c-3p | GPR107 | NM_001136558 |
| hsa-miR-200c-3p | GPR107 | NM_020960 |
| hsa-miR-200c-3p | GXYLT1 | NM_001099650 |
| hsa-miR-200c-3p | GXYLT1 | NM_173601 |
| hsa-miR-200c-3p | HCCS | NM_001122608 |
| hsa-miR-200c-3p | HCCS | NM_001171991 |
| hsa-miR-200c-3p | HCCS | NM_005333 |
| hsa-miR-200c-3p | HMBOX1 | NM_001135726 |
| hsa-miR-200c-3p | HMBOX1 | NM_024567 |
| hsa-miR-200c-3p | HOXA5 | NM_019102 |
| hsa-miR-200c-3p | HPS5 | NM_007216 |
| hsa-miR-200c-3p | HPS5 | NM_181507 |
| hsa-miR-200c-3p | HPS5 | NM_181508 |
| hsa-miR-200c-3p | HS3ST3A1 | NM_006042 |
| hsa-miR-200c-3p | IPO8 | NM_001190995 |
| hsa-miR-200c-3p | IPO8 | NM_006390 |
| hsa-miR-200c-3p | KDR | NM_002253 |
| hsa-miR-200c-3p | KIAA1432 | NM_001206557 |
| hsa-miR-200c-3p | KIAA1432 | NM_020829 |
| hsa-miR-200c-3p | LCA5 | NM_001122769 |
| hsa-miR-200c-3p | LCA5 | NM_181714 |
| hsa-miR-200c-3p | LIN7B | NM_022165 |
| hsa-miR-200c-3p | LMAN1 | NM_005570 |
| hsa-miR-200c-3p | LPPR4 | NM_001166252 |
| hsa-miR-200c-3p | LPPR4 | NM_014839 |
| hsa-miR-200c-3p | MAP4K3 | NM_003618 |
| hsa-miR-200c-3p | MAPRE1 | NM_012325 |
| hsa-miR-200c-3p | MPRIP | NM_015134 |
| hsa-miR-200c-3p | MTF2 | NM_001164391 |
| hsa-miR-200c-3p | MTF2 | NM_001164392 |
| hsa-miR-200c-3p | MTF2 | NM_001164393 |
| hsa-miR-200c-3p | MTF2 | NM_007358 |
| hsa-miR-200c-3p | NAB1 | NM_005966 |
| hsa-miR-200c-3p | NBR1 | NM_005899 |
| hsa-miR-200c-3p | NBR1 | NM_031858 |
| hsa-miR-200c-3p | NBR1 | NM_031862 |
| hsa-miR-200c-3p | NCOA3 | NM_001174087 |
| hsa-miR-200c-3p | NCOA3 | NM_001174088 |
| hsa-miR-200c-3p | NCOA3 | NM_006534 |
| hsa-miR-200c-3p | NCOA3 | NM_181659 |
| hsa-miR-200c-3p | NIN | NM_182944 |
| hsa-miR-200c-3p | NLGN4X | NM_020742 |
| hsa-miR-200c-3p | NLGN4X | NM_181332 |
| hsa-miR-200c-3p | NOG | NM_005450 |
| hsa-miR-200c-3p | NRBF2 | NM_030759 |
| hsa-miR-200c-3p | NRIP1 | NM_003489 |
| hsa-miR-200c-3p | NTRK2 | NM_001018064 |
| hsa-miR-200c-3p | NTRK2 | NM_006180 |
| hsa-miR-200c-3p | OSTM1 | NM_014028 |
| hsa-miR-200c-3p | OXR1 | NM_001198532 |
| hsa-miR-200c-3p | OXR1 | NM_001198533 |
| hsa-miR-200c-3p | OXR1 | NM_001198534 |
| hsa-miR-200c-3p | OXR1 | NM_001198535 |
| hsa-miR-200c-3p | OXR1 | NM_018002 |
| hsa-miR-200c-3p | OXR1 | NM_181354 |
| hsa-miR-200c-3p | PAN3 | NM_175854 |
| hsa-miR-200c-3p | PAPOLG | NM_022894 |
| hsa-miR-200c-3p | PARD6B | NM_032521 |
| hsa-miR-200c-3p | PCSK2 | NM_001201528 |
| hsa-miR-200c-3p | PCSK2 | NM_001201529 |
| hsa-miR-200c-3p | PDIK1L | NM_152835 |
| hsa-miR-200c-3p | PHTF2 | NM_001127357 |
| hsa-miR-200c-3p | PHTF2 | NM_001127358 |
| hsa-miR-200c-3p | PHTF2 | NM_020432 |
| hsa-miR-200c-3p | PIM2 | NM_006875 |
| hsa-miR-200c-3p | PLK2 | NM_006622 |
| hsa-miR-200c-3p | PMAIP1 | NM_021127 |
| hsa-miR-200c-3p | PMPCB | NM_004279 |
| hsa-miR-200c-3p | POLK | NM_016218 |
| hsa-miR-200c-3p | PPFIA1 | NM_003626 |
| hsa-miR-200c-3p | PPM1B | NM_177968 |
| hsa-miR-200c-3p | PPP1R12B | NM_001197131 |
| hsa-miR-200c-3p | PPP1R12B | NM_002481 |
| hsa-miR-200c-3p | PPP1R12B | NM_032103 |
| hsa-miR-200c-3p | PPP1R12B | NM_032104 |
| hsa-miR-200c-3p | PRKAR2B | NM_002736 |
| hsa-miR-200c-3p | PSIP1 | NM_001128217 |
| hsa-miR-200c-3p | PSIP1 | NM_033222 |
| hsa-miR-200c-3p | PTGER2 | NM_000956 |
| hsa-miR-200c-3p | PTPN21 | NM_007039 |
| hsa-miR-200c-3p | RAB37 | NM_001006638 |
| hsa-miR-200c-3p | RAB37 | NM_001163989 |
| hsa-miR-200c-3p | RAB37 | NM_001163990 |
| hsa-miR-200c-3p | RAB37 | NM_175738 |
| hsa-miR-200c-3p | RBFOX3 | NM_001082575 |
| hsa-miR-200c-3p | RBM12B | NM_203390 |
| hsa-miR-200c-3p | RECK | NM_021111 |
| hsa-miR-200c-3p | REEP1 | NM_001164730 |
| hsa-miR-200c-3p | REEP1 | NM_001164731 |
| hsa-miR-200c-3p | REEP1 | NM_001164732 |
| hsa-miR-200c-3p | REEP1 | NM_022912 |
| hsa-miR-200c-3p | RELN | NM_005045 |
| hsa-miR-200c-3p | RELN | NM_173054 |
| hsa-miR-200c-3p | RHOA | NM_001664 |
| hsa-miR-200c-3p | RIMS2 | NM_001100117 |
| hsa-miR-200c-3p | RIMS2 | NM_014677 |
| hsa-miR-200c-3p | RIPK2 | NM_003821 |
| hsa-miR-200c-3p | RND3 | NM_005168 |
| hsa-miR-200c-3p | S100PBP | NM_022753 |
| hsa-miR-200c-3p | SCAF11 | NM_004719 |
| hsa-miR-200c-3p | SCOC | NM_001153446 |
| hsa-miR-200c-3p | SCOC | NM_001153484 |
| hsa-miR-200c-3p | SCOC | NM_001153552 |
| hsa-miR-200c-3p | SCOC | NM_001153585 |
| hsa-miR-200c-3p | SCOC | NM_001153635 |
| hsa-miR-200c-3p | SCOC | NM_001153663 |
| hsa-miR-200c-3p | SCOC | NM_001153690 |
| hsa-miR-200c-3p | SCOC | NM_032547 |
| hsa-miR-200c-3p | SCRT2 | NM_033129 |
| hsa-miR-200c-3p | SDK2 | NM_001144952 |
| hsa-miR-200c-3p | SEC23A | NM_006364 |
| hsa-miR-200c-3p | SECISBP2L | NM_001193489 |
| hsa-miR-200c-3p | SECISBP2L | NM_014701 |
| hsa-miR-200c-3p | SGCE | NM_001099400 |
| hsa-miR-200c-3p | SGCE | NM_001099401 |
| hsa-miR-200c-3p | SGCE | NM_003919 |
| hsa-miR-200c-3p | SGIP1 | NM_032291 |
| hsa-miR-200c-3p | SIN3A | NM_001145357 |
| hsa-miR-200c-3p | SIN3A | NM_001145358 |
| hsa-miR-200c-3p | SIN3A | NM_015477 |
| hsa-miR-200c-3p | SLC35E2 | NM_182838 |
| hsa-miR-200c-3p | SNX16 | NM_022133 |
| hsa-miR-200c-3p | SNX16 | NM_152836 |
| hsa-miR-200c-3p | SNX16 | NM_152837 |
| hsa-miR-200c-3p | SORT1 | NM_001205228 |
| hsa-miR-200c-3p | SORT1 | NM_002959 |
| hsa-miR-200c-3p | ST3GAL2 | NM_006927 |
| hsa-miR-200c-3p | SYNJ1 | NM_001160302 |
| hsa-miR-200c-3p | SYNJ1 | NM_001160306 |
| hsa-miR-200c-3p | SYNJ1 | NM_003895 |
| hsa-miR-200c-3p | SYNJ1 | NM_203446 |
| hsa-miR-200c-3p | TAOK3 | NM_016281 |
| hsa-miR-200c-3p | TFAP2A | NM_001032280 |
| hsa-miR-200c-3p | TFAP2A | NM_001042425 |
| hsa-miR-200c-3p | TFAP2A | NM_003220 |
| hsa-miR-200c-3p | TMEM170B | NM_001100829 |
| hsa-miR-200c-3p | TP73 | NM_001126240 |
| hsa-miR-200c-3p | TP73 | NM_001126241 |
| hsa-miR-200c-3p | TP73 | NM_001126242 |
| hsa-miR-200c-3p | TP73 | NM_001204184 |
| hsa-miR-200c-3p | TP73 | NM_001204185 |
| hsa-miR-200c-3p | TP73 | NM_001204186 |
| hsa-miR-200c-3p | TP73 | NM_001204187 |
| hsa-miR-200c-3p | TP73 | NM_001204188 |
| hsa-miR-200c-3p | TP73 | NM_001204189 |
| hsa-miR-200c-3p | TP73 | NM_001204190 |
| hsa-miR-200c-3p | TP73 | NM_001204191 |
| hsa-miR-200c-3p | TP73 | NM_001204192 |
| hsa-miR-200c-3p | TP73 | NM_005427 |
| hsa-miR-200c-3p | TRIM33 | NM_015906 |
| hsa-miR-200c-3p | TRIM33 | NM_033020 |
| hsa-miR-200c-3p | TRIM62 | NM_018207 |
| hsa-miR-200c-3p | TSC22D1 | NM_006022 |
| hsa-miR-200c-3p | TSC22D1 | NM_183422 |
| hsa-miR-200c-3p | TWISTNB | NM_001002926 |
| hsa-miR-200c-3p | UBE2B | NM_003337 |
| hsa-miR-200c-3p | UBE2W | NM_001001481 |
| hsa-miR-200c-3p | UBE2W | NM_018299 |
| hsa-miR-200c-3p | UHRF1BP1 | NM_017754 |
| hsa-miR-200c-3p | USP25 | NM_013396 |
| hsa-miR-200c-3p | USP6NL | NM_001080491 |
| hsa-miR-200c-3p | USP6NL | NM_014688 |
| hsa-miR-200c-3p | VAT1L | NM_020927 |
| hsa-miR-200c-3p | WAPAL | NM_015045 |
| hsa-miR-200c-3p | WASF3 | NM_006646 |
| hsa-miR-200c-3p | YWHAG | NM_012479 |
| hsa-miR-200c-3p | ZBTB8A | NM_001040441 |
| hsa-miR-200c-3p | ZEB1 | NM_001128128 |
| hsa-miR-200c-3p | ZEB1 | NM_001174093 |
| hsa-miR-200c-3p | ZEB1 | NM_001174094 |
| hsa-miR-200c-3p | ZEB1 | NM_001174095 |
| hsa-miR-200c-3p | ZEB1 | NM_001174096 |
| hsa-miR-200c-3p | ZEB1 | NM_030751 |
| hsa-miR-200c-3p | ZEB2 | NM_001171653 |
| hsa-miR-200c-3p | ZEB2 | NM_014795 |
| hsa-miR-200c-3p | ZFPM2 | NM_012082 |
| hsa-miR-200c-3p | ZFYVE20 | NM_022340 |
| hsa-miR-203a | ACO2 | NM_001098 |
| hsa-miR-203a | AFAP1L2 | NM_001001936 |
| hsa-miR-203a | AFAP1L2 | NM_032550 |
| hsa-miR-203a | AFF2 | NM_001169122 |
| hsa-miR-203a | AFF2 | NM_001169123 |
| hsa-miR-203a | AFF2 | NM_001169124 |
| hsa-miR-203a | AFF2 | NM_001169125 |
| hsa-miR-203a | AFF2 | NM_001170628 |
| hsa-miR-203a | AFF2 | NM_002025 |
| hsa-miR-203a | AHR | NM_001621 |
| hsa-miR-203a | ALG10B | NM_001013620 |
| hsa-miR-203a | ARHGAP42 | NM_152432 |
| hsa-miR-203a | C11orf68 | NM_001135635 |
| hsa-miR-203a | C11orf68 | NM_031450 |
| hsa-miR-203a | C8orf4 | NM_020130 |
| hsa-miR-203a | CASK | NM_001126054 |
| hsa-miR-203a | CASK | NM_001126055 |
| hsa-miR-203a | CASK | NM_003688 |
| hsa-miR-203a | CITED2 | NM_001168388 |
| hsa-miR-203a | CITED2 | NM_001168389 |
| hsa-miR-203a | CITED2 | NM_006079 |
| hsa-miR-203a | CLSTN3 | NM_014718 |
| hsa-miR-203a | DLG5 | NM_004747 |
| hsa-miR-203a | DPY19L4 | NM_181787 |
| hsa-miR-203a | DUSP5 | NM_004419 |
| hsa-miR-203a | EID2 | NM_153232 |
| hsa-miR-203a | EIF5A2 | NM_020390 |
| hsa-miR-203a | ELL2 | NM_012081 |
| hsa-miR-203a | FAM126B | NM_173822 |
| hsa-miR-203a | FOXK1 | NM_001037165 |
| hsa-miR-203a | G6PC2 | NM_001081686 |
| hsa-miR-203a | G6PC2 | NM_021176 |
| hsa-miR-203a | GLCCI1 | NM_138426 |
| hsa-miR-203a | GPATCH1 | NM_018025 |
| hsa-miR-203a | GPR155 | NM_001033045 |
| hsa-miR-203a | GPR155 | NM_152529 |
| hsa-miR-203a | GXYLT1 | NM_001099650 |
| hsa-miR-203a | GXYLT1 | NM_173601 |
| hsa-miR-203a | HERPUD2 | NM_022373 |
| hsa-miR-203a | IDS | NM_000202 |
| hsa-miR-203a | IDS | NM_001166550 |
| hsa-miR-203a | IL24 | NM_001185156 |
| hsa-miR-203a | IL24 | NM_001185157 |
| hsa-miR-203a | IL24 | NM_001185158 |
| hsa-miR-203a | IL24 | NM_006850 |
| hsa-miR-203a | IRS2 | NM_003749 |
| hsa-miR-203a | KAT6A | NM_001099412 |
| hsa-miR-203a | KAT6A | NM_001099413 |
| hsa-miR-203a | KAT6A | NM_006766 |
| hsa-miR-203a | KAZN | NM_001017999 |
| hsa-miR-203a | KAZN | NM_001018000 |
| hsa-miR-203a | KAZN | NM_001018001 |
| hsa-miR-203a | KAZN | NM_015209 |
| hsa-miR-203a | KIAA1429 | NM_015496 |
| hsa-miR-203a | KRT85 | NM_002283 |
| hsa-miR-203a | LASP1 | NM_006148 |
| hsa-miR-203a | LOX | NM_001178102 |
| hsa-miR-203a | LOX | NM_002317 |
| hsa-miR-203a | LPO | NM_001160102 |
| hsa-miR-203a | LPO | NM_006151 |
| hsa-miR-203a | MAP3K13 | NM_001242314 |
| hsa-miR-203a | MAP3K13 | NM_001242317 |
| hsa-miR-203a | MAP3K13 | NM_004721 |
| hsa-miR-203a | MKL2 | NM_014048 |
| hsa-miR-203a | MLANA | NM_005511 |
| hsa-miR-203a | MSR1 | NM_002445 |
| hsa-miR-203a | NBEA | NM_001204197 |
| hsa-miR-203a | NBEA | NM_015678 |
| hsa-miR-203a | NEDD9 | NM_001142393 |
| hsa-miR-203a | NEDD9 | NM_006403 |
| hsa-miR-203a | NUAK1 | NM_014840 |
| hsa-miR-203a | NUDT21 | NM_007006 |
| hsa-miR-203a | OSBPL1A | NM_001242508 |
| hsa-miR-203a | OSBPL1A | NM_018030 |
| hsa-miR-203a | OSBPL1A | NM_080597 |
| hsa-miR-203a | PAQR3 | NM_001040202 |
| hsa-miR-203a | PARP16 | NM_017851 |
| hsa-miR-203a | PCBP2 | NM_001098620 |
| hsa-miR-203a | PCBP2 | NM_001128911 |
| hsa-miR-203a | PCBP2 | NM_001128912 |
| hsa-miR-203a | PCBP2 | NM_001128913 |
| hsa-miR-203a | PCBP2 | NM_001128914 |
| hsa-miR-203a | PCBP2 | NM_005016 |
| hsa-miR-203a | PCBP2 | NM_031989 |
| hsa-miR-203a | PDPN | NM_001006624 |
| hsa-miR-203a | PDPN | NM_001006625 |
| hsa-miR-203a | PDPN | NM_006474 |
| hsa-miR-203a | PDPN | NM_198389 |
| hsa-miR-203a | PRUNE2 | NM_015225 |
| hsa-miR-203a | RAP2A | NM_021033 |
| hsa-miR-203a | RNASE4 | NM_002937 |
| hsa-miR-203a | RNASE4 | NM_194431 |
| hsa-miR-203a | SAMD5 | NM_001030060 |
| hsa-miR-203a | SEMA5A | NM_003966 |
| hsa-miR-203a | SLC30A6 | NM_001193513 |
| hsa-miR-203a | SLC30A6 | NM_001193514 |
| hsa-miR-203a | SLC30A6 | NM_001193515 |
| hsa-miR-203a | SLC30A6 | NM_017964 |
| hsa-miR-203a | SMAD9 | NM_001127217 |
| hsa-miR-203a | SMAD9 | NM_005905 |
| hsa-miR-203a | SP4 | NM_003112 |
| hsa-miR-203a | STXBP5L | NM_014980 |
| hsa-miR-203a | TADA1 | NM_053053 |
| hsa-miR-203a | TAF9B | NM_015975 |
| hsa-miR-203a | TTC39A | NM_001080494 |
| hsa-miR-203a | TTC39A | NM_001144832 |
| hsa-miR-203a | UBP1 | NM_001128160 |
| hsa-miR-203a | UBP1 | NM_001128161 |
| hsa-miR-203a | UBP1 | NM_014517 |
| hsa-miR-203a | UBR1 | NM_174916 |
| hsa-miR-203a | VTI1A | NM_145206 |
| hsa-miR-203a | ZAK | NM_133646 |
| hsa-miR-203a | ZDHHC15 | NM_001146257 |
| hsa-miR-203a | ZNF281 | NM_012482 |
| hsa-miR-21-5p | ARMCX1 | NM_016608 |
| hsa-miR-21-5p | BEST3 | NM_032735 |
| hsa-miR-21-5p | BEST3 | NM_152439 |
| hsa-miR-21-5p | CCL1 | NM_002981 |
| hsa-miR-21-5p | CCR7 | NM_001838 |
| hsa-miR-21-5p | CHD7 | NM_017780 |
| hsa-miR-21-5p | CNKSR2 | NM_001168648 |
| hsa-miR-21-5p | CNKSR2 | NM_001168649 |
| hsa-miR-21-5p | CNTFR | NM_001207011 |
| hsa-miR-21-5p | CNTFR | NM_001842 |
| hsa-miR-21-5p | CNTFR | NM_147164 |
| hsa-miR-21-5p | DLGAP1 | NM_001003809 |
| hsa-miR-21-5p | DLGAP1 | NM_001242762 |
| hsa-miR-21-5p | DLGAP1 | NM_001242763 |
| hsa-miR-21-5p | DLGAP1 | NM_001242764 |
| hsa-miR-21-5p | DLGAP1 | NM_001242766 |
| hsa-miR-21-5p | DLGAP1 | NM_004746 |
| hsa-miR-21-5p | ENAH | NM_001008493 |
| hsa-miR-21-5p | ENAH | NM_018212 |
| hsa-miR-21-5p | FAM63B | NM_001040450 |
| hsa-miR-21-5p | FAM63B | NM_001040453 |
| hsa-miR-21-5p | GLYR1 | NM_032569 |
| hsa-miR-21-5p | GPR64 | NM_001079858 |
| hsa-miR-21-5p | GPR64 | NM_001079859 |
| hsa-miR-21-5p | GPR64 | NM_001079860 |
| hsa-miR-21-5p | GPR64 | NM_001184833 |
| hsa-miR-21-5p | GPR64 | NM_001184834 |
| hsa-miR-21-5p | GPR64 | NM_001184835 |
| hsa-miR-21-5p | GPR64 | NM_001184836 |
| hsa-miR-21-5p | GPR64 | NM_001184837 |
| hsa-miR-21-5p | GPR64 | NM_005756 |
| hsa-miR-21-5p | KLHL15 | NM_030624 |
| hsa-miR-21-5p | KRIT1 | NM_001013406 |
| hsa-miR-21-5p | KRIT1 | NM_004912 |
| hsa-miR-21-5p | KRIT1 | NM_194454 |
| hsa-miR-21-5p | KRIT1 | NM_194455 |
| hsa-miR-21-5p | KRIT1 | NM_194456 |
| hsa-miR-21-5p | LANCL1 | NM_001136574 |
| hsa-miR-21-5p | LANCL1 | NM_001136575 |
| hsa-miR-21-5p | LANCL1 | NM_006055 |
| hsa-miR-21-5p | LMBR1 | NM_022458 |
| hsa-miR-21-5p | MEIS1 | NM_002398 |
| hsa-miR-21-5p | MPRIP | NM_015134 |
| hsa-miR-21-5p | MPRIP | NM_201274 |
| hsa-miR-21-5p | MSH2 | NM_000251 |
| hsa-miR-21-5p | NFIB | NM_001190737 |
| hsa-miR-21-5p | NFIB | NM_001190738 |
| hsa-miR-21-5p | NFIB | NM_005596 |
| hsa-miR-21-5p | PAIP2B | NM_020459 |
| hsa-miR-21-5p | PCBP1 | NM_006196 |
| hsa-miR-21-5p | PCBP2 | NM_001098620 |
| hsa-miR-21-5p | PCBP2 | NM_001128911 |
| hsa-miR-21-5p | PCBP2 | NM_001128912 |
| hsa-miR-21-5p | PCBP2 | NM_001128913 |
| hsa-miR-21-5p | PCBP2 | NM_001128914 |
| hsa-miR-21-5p | PCBP2 | NM_005016 |
| hsa-miR-21-5p | PCBP2 | NM_031989 |
| hsa-miR-21-5p | PDZD2 | NM_178140 |
| hsa-miR-21-5p | PPARA | NM_001001928 |
| hsa-miR-21-5p | PPARA | NM_005036 |
| hsa-miR-21-5p | PTPN14 | NM_005401 |
| hsa-miR-21-5p | RECK | NM_021111 |
| hsa-miR-21-5p | RHOB | NM_004040 |
| hsa-miR-21-5p | RP2 | NM_006915 |
| hsa-miR-21-5p | SLC8A3 | NM_001130417 |
| hsa-miR-21-5p | SLC8A3 | NM_033262 |
| hsa-miR-21-5p | SLC8A3 | NM_058240 |
| hsa-miR-21-5p | SLC8A3 | NM_182932 |
| hsa-miR-21-5p | SLC8A3 | NM_182936 |
| hsa-miR-21-5p | SLC8A3 | NM_183002 |
| hsa-miR-21-5p | SOX5 | NM_006940 |
| hsa-miR-21-5p | SOX5 | NM_152989 |
| hsa-miR-21-5p | SOX5 | NM_178010 |
| hsa-miR-21-5p | SRL | NM_001098814 |
| hsa-miR-21-5p | STAG2 | NM_001042749 |
| hsa-miR-21-5p | STAG2 | NM_001042750 |
| hsa-miR-21-5p | STAG2 | NM_001042751 |
| hsa-miR-21-5p | STAG2 | NM_006603 |
| hsa-miR-21-5p | STK40 | NM_032017 |
| hsa-miR-21-5p | TGFBR2 | NM_001024847 |
| hsa-miR-21-5p | TGFBR2 | NM_003242 |
| hsa-miR-21-5p | TNFRSF11B | NM_002546 |
| hsa-miR-21-5p | YOD1 | NM_018566 |
| hsa-miR-21-5p | ZNF704 | NM_001033723 |
| hsa-miR-210-3p | B4GALT5 | NM_004776 |
| hsa-miR-210-3p | DTX1 | NM_004416 |
| hsa-miR-210-3p | ELFN2 | NM_052906 |
| hsa-miR-210-3p | GPD1L | NM_015141 |
| hsa-miR-210-3p | HIF3A | NM_022462 |
| hsa-miR-210-3p | HIF3A | NM_152794 |
| hsa-miR-210-3p | HIF3A | NM_152795 |
| hsa-miR-210-3p | ISCU | NM_014301 |
| hsa-miR-210-3p | ISCU | NM_213595 |
| hsa-miR-210-3p | RGMA | NM_001166283 |
| hsa-miR-210-3p | RGMA | NM_001166286 |
| hsa-miR-210-3p | RGMA | NM_001166287 |
| hsa-miR-210-3p | RGMA | NM_001166288 |
| hsa-miR-210-3p | RGMA | NM_001166289 |
| hsa-miR-210-3p | RGMA | NM_020211 |
| hsa-miR-210-3p | SCRT1 | NM_031309 |
| hsa-miR-210-3p | SMG5 | NM_015327 |
| hsa-miR-210-3p | SYNGAP1 | NM_006772 |
| hsa-miR-210-3p | ZFAND3 | NM_021943 |
| hsa-miR-210-3p | ZNF148 | NM_021964 |
| hsa-miR-210-3p | ZNF462 | NM_021224 |
| hsa-miR-224-5p | ACSL4 | NM_004458 |
| hsa-miR-224-5p | ACSL4 | NM_022977 |
| hsa-miR-224-5p | ADAMTS5 | NM_007038 |
| hsa-miR-224-5p | AFF3 | NM_001025108 |
| hsa-miR-224-5p | AFF3 | NM_002285 |
| hsa-miR-224-5p | ANKRD40 | NM_052855 |
| hsa-miR-224-5p | APBA2 | NM_001130414 |
| hsa-miR-224-5p | APBA2 | NM_005503 |
| hsa-miR-224-5p | ARHGEF12 | NM_001198665 |
| hsa-miR-224-5p | ARHGEF12 | NM_015313 |
| hsa-miR-224-5p | ASCL4 | NM_203436 |
| hsa-miR-224-5p | AZIN1 | NM_015878 |
| hsa-miR-224-5p | AZIN1 | NM_148174 |
| hsa-miR-224-5p | BAZ2A | NM_013449 |
| hsa-miR-224-5p | C1GALT1 | NM_020156 |
| hsa-miR-224-5p | C1orf52 | NM_198077 |
| hsa-miR-224-5p | CBL | NM_005188 |
| hsa-miR-224-5p | CBX6 | NM_014292 |
| hsa-miR-224-5p | CCDC71 | NM_022903 |
| hsa-miR-224-5p | CDK9 | NM_001261 |
| hsa-miR-224-5p | CDS2 | NM_003818 |
| hsa-miR-224-5p | CORO1C | NM_014325 |
| hsa-miR-224-5p | DDX3X | NM_001193416 |
| hsa-miR-224-5p | DDX3X | NM_001193417 |
| hsa-miR-224-5p | DDX3X | NM_001356 |
| hsa-miR-224-5p | DDX52 | NM_007010 |
| hsa-miR-224-5p | DOT1L | NM_032482 |
| hsa-miR-224-5p | DPYSL2 | NM_001197293 |
| hsa-miR-224-5p | DPYSL2 | NM_001386 |
| hsa-miR-224-5p | DUSP11 | NM_003584 |
| hsa-miR-224-5p | DUSP16 | NM_030640 |
| hsa-miR-224-5p | ENAH | NM_001008493 |
| hsa-miR-224-5p | ENAH | NM_018212 |
| hsa-miR-224-5p | FNDC5 | NM_001171941 |
| hsa-miR-224-5p | FUT9 | NM_006581 |
| hsa-miR-224-5p | GALNT2 | NM_004481 |
| hsa-miR-224-5p | GNAI3 | NM_006496 |
| hsa-miR-224-5p | GPC4 | NM_001448 |
| hsa-miR-224-5p | GPR180 | NM_180989 |
| hsa-miR-224-5p | HMOX2 | NM_001127204 |
| hsa-miR-224-5p | HMOX2 | NM_001127205 |
| hsa-miR-224-5p | HMOX2 | NM_001127206 |
| hsa-miR-224-5p | HMOX2 | NM_002134 |
| hsa-miR-224-5p | HOXA11 | NM_005523 |
| hsa-miR-224-5p | IPO7 | NM_006391 |
| hsa-miR-224-5p | KAL1 | NM_000216 |
| hsa-miR-224-5p | KAT6A | NM_001099412 |
| hsa-miR-224-5p | KAT6A | NM_001099413 |
| hsa-miR-224-5p | KAT6A | NM_006766 |
| hsa-miR-224-5p | KCNMA1 | NM_001014797 |
| hsa-miR-224-5p | KDELR1 | NM_006801 |
| hsa-miR-224-5p | KLK15 | NM_017509 |
| hsa-miR-224-5p | KLLN | NM_001126049 |
| hsa-miR-224-5p | LPAR5 | NM_001142961 |
| hsa-miR-224-5p | LPAR5 | NM_020400 |
| hsa-miR-224-5p | MAP3K7 | NM_003188 |
| hsa-miR-224-5p | MAP3K7 | NM_145331 |
| hsa-miR-224-5p | MAPK14 | NM_001315 |
| hsa-miR-224-5p | MAPK14 | NM_139012 |
| hsa-miR-224-5p | MAPK14 | NM_139014 |
| hsa-miR-224-5p | MRPL42 | NM_014050 |
| hsa-miR-224-5p | MRPL42 | NM_172177 |
| hsa-miR-224-5p | NSMAF | NM_001144772 |
| hsa-miR-224-5p | NSMAF | NM_003580 |
| hsa-miR-224-5p | NUAK1 | NM_014840 |
| hsa-miR-224-5p | NUP153 | NM_005124 |
| hsa-miR-224-5p | OCLN | NM_001205254 |
| hsa-miR-224-5p | OCLN | NM_001205255 |
| hsa-miR-224-5p | OCLN | NM_002538 |
| hsa-miR-224-5p | OPHN1 | NM_002547 |
| hsa-miR-224-5p | OTUB2 | NM_023112 |
| hsa-miR-224-5p | PDCD7 | NM_005707 |
| hsa-miR-224-5p | PDE8A | NM_002605 |
| hsa-miR-224-5p | PDE8A | NM_173454 |
| hsa-miR-224-5p | PFKFB3 | NM_001145443 |
| hsa-miR-224-5p | PFKFB3 | NM_004566 |
| hsa-miR-224-5p | PHF6 | NM_001015877 |
| hsa-miR-224-5p | PHF6 | NM_032458 |
| hsa-miR-224-5p | PHLPP2 | NM_015020 |
| hsa-miR-224-5p | PJA2 | NM_014819 |
| hsa-miR-224-5p | PRPS2 | NM_001039091 |
| hsa-miR-224-5p | PRPS2 | NM_002765 |
| hsa-miR-224-5p | PUS10 | NM_144709 |
| hsa-miR-224-5p | RALA | NM_005402 |
| hsa-miR-224-5p | RASD1 | NM_016084 |
| hsa-miR-224-5p | RBFOX1 | NM_001142333 |
| hsa-miR-224-5p | RBFOX1 | NM_001142334 |
| hsa-miR-224-5p | RBFOX1 | NM_018723 |
| hsa-miR-224-5p | RBFOX1 | NM_145891 |
| hsa-miR-224-5p | RBFOX1 | NM_145892 |
| hsa-miR-224-5p | RBFOX1 | NM_145893 |
| hsa-miR-224-5p | RBM33 | NM_053043 |
| hsa-miR-224-5p | RNF38 | NM_022781 |
| hsa-miR-224-5p | RNF38 | NM_194328 |
| hsa-miR-224-5p | RNF38 | NM_194329 |
| hsa-miR-224-5p | RNF38 | NM_194330 |
| hsa-miR-224-5p | RNF38 | NM_194332 |
| hsa-miR-224-5p | SFRP2 | NM_003013 |
| hsa-miR-224-5p | SH3KBP1 | NM_001024666 |
| hsa-miR-224-5p | SH3KBP1 | NM_001184960 |
| hsa-miR-224-5p | SH3KBP1 | NM_031892 |
| hsa-miR-224-5p | SLC12A2 | NM_001046 |
| hsa-miR-224-5p | SLC12A5 | NM_001134771 |
| hsa-miR-224-5p | SLC12A5 | NM_020708 |
| hsa-miR-224-5p | SMAD5 | NM_001001419 |
| hsa-miR-224-5p | SMAD5 | NM_001001420 |
| hsa-miR-224-5p | SMAD5 | NM_005903 |
| hsa-miR-224-5p | SP7 | NM_001173467 |
| hsa-miR-224-5p | SP7 | NM_152860 |
| hsa-miR-224-5p | TMEM110 | NM_198563 |
| hsa-miR-224-5p | TMEM132B | NM_052907 |
| hsa-miR-224-5p | TMOD2 | NM_001142885 |
| hsa-miR-224-5p | TMOD2 | NM_014548 |
| hsa-miR-224-5p | TRAPPC10 | NM_003274 |
| hsa-miR-224-5p | TRIM9 | NM_015163 |
| hsa-miR-224-5p | WTAP | NM_152857 |
| hsa-miR-224-5p | WTAP | NM_152858 |
| hsa-miR-224-5p | YIPF6 | NM_001195214 |
| hsa-miR-224-5p | YIPF6 | NM_173834 |
| hsa-miR-224-5p | ZDHHC20 | NM_153251 |
| hsa-miR-224-5p | ZFHX4 | NM_024721 |
| hsa-miR-224-5p | ZNF207 | NM_001032293 |
| hsa-miR-224-5p | ZNF207 | NM_001098507 |
| hsa-miR-224-5p | ZNF207 | NM_003457 |
| hsa-miR-25-5p | DPYSL5 | NM_020134 |
| hsa-miR-25-5p | MARK2 | NM_001039469 |
| hsa-miR-25-5p | MARK2 | NM_001163296 |
| hsa-miR-25-5p | MARK2 | NM_001163297 |
| hsa-miR-25-5p | MARK2 | NM_004954 |
| hsa-miR-25-5p | MARK2 | NM_017490 |
| hsa-miR-25-5p | 43709 | NM_052838 |
| hsa-miR-30a-5p | A1CF | NM_001198818 |
| hsa-miR-30a-5p | A1CF | NM_001198819 |
| hsa-miR-30a-5p | A1CF | NM_001198820 |
| hsa-miR-30a-5p | A1CF | NM_014576 |
| hsa-miR-30a-5p | A1CF | NM_138932 |
| hsa-miR-30a-5p | A1CF | NM_138933 |
| hsa-miR-30a-5p | ACVR1 | NM_001105 |
| hsa-miR-30a-5p | ACVR1 | NM_001111067 |
| hsa-miR-30a-5p | ADAM19 | NM_033274 |
| hsa-miR-30a-5p | ALG9 | NM_001077690 |
| hsa-miR-30a-5p | ALG9 | NM_001077691 |
| hsa-miR-30a-5p | ALG9 | NM_001077692 |
| hsa-miR-30a-5p | ALG9 | NM_024740 |
| hsa-miR-30a-5p | ANKRA2 | NM_023039 |
| hsa-miR-30a-5p | ARHGEF6 | NM_004840 |
| hsa-miR-30a-5p | ASB3 | NM_001201965 |
| hsa-miR-30a-5p | ASB3 | NM_016115 |
| hsa-miR-30a-5p | ASB3 | NM_145863 |
| hsa-miR-30a-5p | ATF1 | NM_005171 |
| hsa-miR-30a-5p | B3GNT5 | NM_032047 |
| hsa-miR-30a-5p | B4GALT6 | NM_004775 |
| hsa-miR-30a-5p | BEAN1 | NM_001136106 |
| hsa-miR-30a-5p | BEAN1 | NM_001178020 |
| hsa-miR-30a-5p | BRWD3 | NM_153252 |
| hsa-miR-30a-5p | C12orf76 | NM_207435 |
| hsa-miR-30a-5p | C14orf28 | NM_001017923 |
| hsa-miR-30a-5p | C3orf18 | NM_001171740 |
| hsa-miR-30a-5p | C3orf18 | NM_001171741 |
| hsa-miR-30a-5p | C3orf18 | NM_001171743 |
| hsa-miR-30a-5p | C3orf18 | NM_016210 |
| hsa-miR-30a-5p | CADPS | NM_003716 |
| hsa-miR-30a-5p | CADPS | NM_183393 |
| hsa-miR-30a-5p | CADPS | NM_183394 |
| hsa-miR-30a-5p | CALCR | NM_001164737 |
| hsa-miR-30a-5p | CALCR | NM_001164738 |
| hsa-miR-30a-5p | CALCR | NM_001742 |
| hsa-miR-30a-5p | CCDC117 | NM_173510 |
| hsa-miR-30a-5p | CELSR3 | NM_001407 |
| hsa-miR-30a-5p | CFL2 | NM_021914 |
| hsa-miR-30a-5p | CFL2 | NM_138638 |
| hsa-miR-30a-5p | CHMP2B | NM_014043 |
| hsa-miR-30a-5p | CHST1 | NM_003654 |
| hsa-miR-30a-5p | CLN8 | NM_018941 |
| hsa-miR-30a-5p | CLRN1 | NM_001195794 |
| hsa-miR-30a-5p | CLRN1 | NM_174878 |
| hsa-miR-30a-5p | COL25A1 | NM_198721 |
| hsa-miR-30a-5p | CPSF6 | NM_007007 |
| hsa-miR-30a-5p | CTTNBP2NL | NM_018704 |
| hsa-miR-30a-5p | DCX | NM_000555 |
| hsa-miR-30a-5p | DCX | NM_001195553 |
| hsa-miR-30a-5p | DCX | NM_178151 |
| hsa-miR-30a-5p | DCX | NM_178153 |
| hsa-miR-30a-5p | DDIT4 | NM_019058 |
| hsa-miR-30a-5p | DEXI | NM_014015 |
| hsa-miR-30a-5p | DGKZ | NM_001105540 |
| hsa-miR-30a-5p | DGKZ | NM_001199266 |
| hsa-miR-30a-5p | DGKZ | NM_001199267 |
| hsa-miR-30a-5p | DGKZ | NM_001199268 |
| hsa-miR-30a-5p | DGKZ | NM_003646 |
| hsa-miR-30a-5p | DGKZ | NM_201532 |
| hsa-miR-30a-5p | DGKZ | NM_201533 |
| hsa-miR-30a-5p | DLGAP1 | NM_001242761 |
| hsa-miR-30a-5p | DLGAP1 | NM_001242765 |
| hsa-miR-30a-5p | DNAJC25-GNG10 | NM_004125 |
| hsa-miR-30a-5p | EED | NM_003797 |
| hsa-miR-30a-5p | EED | NM_152991 |
| hsa-miR-30a-5p | ELL | NM_006532 |
| hsa-miR-30a-5p | ELMOD2 | NM_153702 |
| hsa-miR-30a-5p | ERLIN1 | NM_001100626 |
| hsa-miR-30a-5p | ERLIN1 | NM_006459 |
| hsa-miR-30a-5p | FAHD1 | NM_031208 |
| hsa-miR-30a-5p | FAM160B1 | NM_020940 |
| hsa-miR-30a-5p | FAM43A | NM_153690 |
| hsa-miR-30a-5p | FAM73B | NM_032809 |
| hsa-miR-30a-5p | FLVCR1 | NM_014053 |
| hsa-miR-30a-5p | GALNT2 | NM_004481 |
| hsa-miR-30a-5p | GALNT3 | NM_004482 |
| hsa-miR-30a-5p | GALNT7 | NM_017423 |
| hsa-miR-30a-5p | GCLC | NM_001197115 |
| hsa-miR-30a-5p | GCLC | NM_001498 |
| hsa-miR-30a-5p | GLCCI1 | NM_138426 |
| hsa-miR-30a-5p | GNAI2 | NM_001166425 |
| hsa-miR-30a-5p | GNAI2 | NM_002070 |
| hsa-miR-30a-5p | GNG10 | NM_001017998 |
| hsa-miR-30a-5p | GNG10 | NM_001198664 |
| hsa-miR-30a-5p | GPR108 | NM_001080452 |
| hsa-miR-30a-5p | GPR75-ASB3 | NM_001164165 |
| hsa-miR-30a-5p | HHIPL1 | NM_001127258 |
| hsa-miR-30a-5p | HNRNPA3 | NM_194247 |
| hsa-miR-30a-5p | IP6K3 | NM_001142883 |
| hsa-miR-30a-5p | IP6K3 | NM_054111 |
| hsa-miR-30a-5p | IRF4 | NM_001195286 |
| hsa-miR-30a-5p | IRF4 | NM_002460 |
| hsa-miR-30a-5p | JAKMIP3 | NM_001105521 |
| hsa-miR-30a-5p | JPH4 | NM_001146028 |
| hsa-miR-30a-5p | JPH4 | NM_032452 |
| hsa-miR-30a-5p | KCNJ12 | NM_021012 |
| hsa-miR-30a-5p | KCNJ18 | NM_001194958 |
| hsa-miR-30a-5p | KIAA1715 | NM_030650 |
| hsa-miR-30a-5p | KIF16B | NM_001199865 |
| hsa-miR-30a-5p | KIF16B | NM_024704 |
| hsa-miR-30a-5p | KLF9 | NM_001206 |
| hsa-miR-30a-5p | LHX1 | NM_005568 |
| hsa-miR-30a-5p | LIMCH1 | NM_001112717 |
| hsa-miR-30a-5p | LIMCH1 | NM_001112718 |
| hsa-miR-30a-5p | LIMCH1 | NM_001112719 |
| hsa-miR-30a-5p | LIMCH1 | NM_001112720 |
| hsa-miR-30a-5p | LIMCH1 | NM_014988 |
| hsa-miR-30a-5p | LPPR4 | NM_001166252 |
| hsa-miR-30a-5p | LPPR4 | NM_014839 |
| hsa-miR-30a-5p | LRFN2 | NM_020737 |
| hsa-miR-30a-5p | MAP3K5 | NM_005923 |
| hsa-miR-30a-5p | MAP4K4 | NM_001242559 |
| hsa-miR-30a-5p | MAP4K4 | NM_001242560 |
| hsa-miR-30a-5p | MAP4K4 | NM_004834 |
| hsa-miR-30a-5p | MAP4K4 | NM_145686 |
| hsa-miR-30a-5p | MAP4K4 | NM_145687 |
| hsa-miR-30a-5p | MAP6 | NM_207577 |
| hsa-miR-30a-5p | MBOAT1 | NM_001080480 |
| hsa-miR-30a-5p | METAP2 | NM_006838 |
| hsa-miR-30a-5p | MIER3 | NM_152622 |
| hsa-miR-30a-5p | MKRN3 | NM_005664 |
| hsa-miR-30a-5p | NAPG | NM_003826 |
| hsa-miR-30a-5p | NEFL | NM_006158 |
| hsa-miR-30a-5p | NHLH2 | NM_001111061 |
| hsa-miR-30a-5p | NHLH2 | NM_005599 |
| hsa-miR-30a-5p | NRG3 | NM_001010848 |
| hsa-miR-30a-5p | NRG3 | NM_001165972 |
| hsa-miR-30a-5p | NRG3 | NM_001165973 |
| hsa-miR-30a-5p | ORC2 | NM_006190 |
| hsa-miR-30a-5p | PAPOLB | NM_020144 |
| hsa-miR-30a-5p | PARP16 | NM_017851 |
| hsa-miR-30a-5p | PCDH10 | NM_032961 |
| hsa-miR-30a-5p | PDE7A | NM_001242318 |
| hsa-miR-30a-5p | PDE7A | NM_002603 |
| hsa-miR-30a-5p | PHIP | NM_017934 |
| hsa-miR-30a-5p | PHTF2 | NM_001127357 |
| hsa-miR-30a-5p | PHTF2 | NM_001127358 |
| hsa-miR-30a-5p | PHTF2 | NM_020432 |
| hsa-miR-30a-5p | PLEKHM3 | NM_001080475 |
| hsa-miR-30a-5p | POP1 | NM_001145860 |
| hsa-miR-30a-5p | POP1 | NM_001145861 |
| hsa-miR-30a-5p | POP1 | NM_015029 |
| hsa-miR-30a-5p | PPARGC1B | NM_001172698 |
| hsa-miR-30a-5p | PPARGC1B | NM_001172699 |
| hsa-miR-30a-5p | PPARGC1B | NM_133263 |
| hsa-miR-30a-5p | PRLR | NM_000949 |
| hsa-miR-30a-5p | PRLR | NM_001204314 |
| hsa-miR-30a-5p | PTGFRN | NM_020440 |
| hsa-miR-30a-5p | PTPDC1 | NM_152422 |
| hsa-miR-30a-5p | PTPDC1 | NM_177995 |
| hsa-miR-30a-5p | RALGPS2 | NM_152663 |
| hsa-miR-30a-5p | RBM12 | NM_001198838 |
| hsa-miR-30a-5p | RBM12 | NM_001198840 |
| hsa-miR-30a-5p | RBM12 | NM_006047 |
| hsa-miR-30a-5p | RBM12 | NM_152838 |
| hsa-miR-30a-5p | REV1 | NM_001037872 |
| hsa-miR-30a-5p | REV1 | NM_016316 |
| hsa-miR-30a-5p | RFX2 | NM_000635 |
| hsa-miR-30a-5p | RFX2 | NM_134433 |
| hsa-miR-30a-5p | RFX6 | NM_173560 |
| hsa-miR-30a-5p | RHEBL1 | NM_144593 |
| hsa-miR-30a-5p | RHOB | NM_004040 |
| hsa-miR-30a-5p | RNF220 | NM_018150 |
| hsa-miR-30a-5p | SCARA5 | NM_173833 |
| hsa-miR-30a-5p | SEC22A | NM_012430 |
| hsa-miR-30a-5p | SEMA3A | NM_006080 |
| hsa-miR-30a-5p | SETD5 | NM_001080517 |
| hsa-miR-30a-5p | SIK3 | NM_025164 |
| hsa-miR-30a-5p | SLC35A5 | NM_017945 |
| hsa-miR-30a-5p | SLC38A7 | NM_018231 |
| hsa-miR-30a-5p | SLC41A2 | NM_032148 |
| hsa-miR-30a-5p | SLC7A10 | NM_019849 |
| hsa-miR-30a-5p | SMAD2 | NM_001003652 |
| hsa-miR-30a-5p | SMAD2 | NM_001135937 |
| hsa-miR-30a-5p | SMAD2 | NM_005901 |
| hsa-miR-30a-5p | SNAI1 | NM_005985 |
| hsa-miR-30a-5p | SNTB2 | NM_006750 |
| hsa-miR-30a-5p | SNX1 | NM_003099 |
| hsa-miR-30a-5p | SNX1 | NM_148955 |
| hsa-miR-30a-5p | SNX16 | NM_022133 |
| hsa-miR-30a-5p | SNX16 | NM_152836 |
| hsa-miR-30a-5p | SNX16 | NM_152837 |
| hsa-miR-30a-5p | SP4 | NM_003112 |
| hsa-miR-30a-5p | SRGAP3 | NM_001033117 |
| hsa-miR-30a-5p | SRGAP3 | NM_014850 |
| hsa-miR-30a-5p | SSX2IP | NM_001166293 |
| hsa-miR-30a-5p | SSX2IP | NM_001166294 |
| hsa-miR-30a-5p | SSX2IP | NM_001166295 |
| hsa-miR-30a-5p | SSX2IP | NM_001166417 |
| hsa-miR-30a-5p | SSX2IP | NM_014021 |
| hsa-miR-30a-5p | STARD3 | NM_001165937 |
| hsa-miR-30a-5p | STARD3 | NM_001165938 |
| hsa-miR-30a-5p | STARD3 | NM_006804 |
| hsa-miR-30a-5p | STK39 | NM_013233 |
| hsa-miR-30a-5p | SUPT3H | NM_181356 |
| hsa-miR-30a-5p | TAOK1 | NM_020791 |
| hsa-miR-30a-5p | TAOK1 | NM_025142 |
| hsa-miR-30a-5p | TMCC1 | NM_001017395 |
| hsa-miR-30a-5p | TMCC1 | NM_001128224 |
| hsa-miR-30a-5p | TMEFF1 | NM_003692 |
| hsa-miR-30a-5p | TMEM26 | NM_178505 |
| hsa-miR-30a-5p | TNKS | NM_003747 |
| hsa-miR-30a-5p | TOX | NM_014729 |
| hsa-miR-30a-5p | TPRG1L | NM_182752 |
| hsa-miR-30a-5p | TRPA1 | NM_007332 |
| hsa-miR-30a-5p | TTBK1 | NM_032538 |
| hsa-miR-30a-5p | UBE2G1 | NM_003342 |
| hsa-miR-30a-5p | UBN1 | NM_001079514 |
| hsa-miR-30a-5p | UBN2 | NM_173569 |
| hsa-miR-30a-5p | WDR82 | NM_025222 |
| hsa-miR-30a-5p | ZBTB39 | NM_014830 |
| hsa-miR-30a-5p | ZBTB41 | NM_194314 |
| hsa-miR-30a-5p | ZDHHC20 | NM_153251 |
| hsa-miR-30a-5p | ZFC3H1 | NM_144982 |
| hsa-miR-30a-5p | ZNF644 | NM_016620 |
| hsa-miR-30a-5p | ZNF644 | NM_032186 |
| hsa-miR-30a-5p | ZNF644 | NM_201269 |
| hsa-miR-30a-5p | ZNF711 | NM_021998 |
| hsa-miR-30a-5p | ZNRF1 | NM_032268 |
| hsa-miR-3175 | ABAT | NM_000663 |
| hsa-miR-3175 | ABAT | NM_001127448 |
| hsa-miR-3175 | ABAT | NM_020686 |
| hsa-miR-3175 | ACTR1A | NM_005736 |
| hsa-miR-3175 | ADAMTS5 | NM_007038 |
| hsa-miR-3175 | AGPAT6 | NM_178819 |
| hsa-miR-3175 | ANKRD52 | NM_173595 |
| hsa-miR-3175 | APH1A | NM_001077628 |
| hsa-miR-3175 | APH1A | NM_016022 |
| hsa-miR-3175 | APLNR | NM_005161 |
| hsa-miR-3175 | APOA5 | NM_001166598 |
| hsa-miR-3175 | APOA5 | NM_052968 |
| hsa-miR-3175 | ARHGAP39 | NM_025251 |
| hsa-miR-3175 | ARPC4-TTLL3 | NM_001198793 |
| hsa-miR-3175 | ATP2B4 | NM_001001396 |
| hsa-miR-3175 | ATP2B4 | NM_001684 |
| hsa-miR-3175 | ATXN7L3 | NM_001098833 |
| hsa-miR-3175 | ATXN7L3 | NM_020218 |
| hsa-miR-3175 | B4GALT5 | NM_004776 |
| hsa-miR-3175 | BARHL2 | NM_020063 |
| hsa-miR-3175 | BRPF3 | NM_015695 |
| hsa-miR-3175 | BSDC1 | NM_001143888 |
| hsa-miR-3175 | BSDC1 | NM_001143889 |
| hsa-miR-3175 | BSDC1 | NM_001143890 |
| hsa-miR-3175 | BSDC1 | NM_018045 |
| hsa-miR-3175 | C16orf89 | NM_152459 |
| hsa-miR-3175 | C1orf87 | NM_152377 |
| hsa-miR-3175 | CACNG2 | NM_006078 |
| hsa-miR-3175 | CACNG4 | NM_014405 |
| hsa-miR-3175 | CBX5 | NM_001127321 |
| hsa-miR-3175 | CBX5 | NM_001127322 |
| hsa-miR-3175 | CBX5 | NM_012117 |
| hsa-miR-3175 | CCDC97 | NM_052848 |
| hsa-miR-3175 | CCL22 | NM_002990 |
| hsa-miR-3175 | CCNY | NM_145012 |
| hsa-miR-3175 | CCNY | NM_181698 |
| hsa-miR-3175 | CDK5R2 | NM_003936 |
| hsa-miR-3175 | CDR2L | NM_014603 |
| hsa-miR-3175 | CPSF7 | NM_001136040 |
| hsa-miR-3175 | CPSF7 | NM_001142565 |
| hsa-miR-3175 | CPSF7 | NM_024811 |
| hsa-miR-3175 | CRTAP | NM_006371 |
| hsa-miR-3175 | CSNK1A1 | NM_001025105 |
| hsa-miR-3175 | CSNK1A1 | NM_001892 |
| hsa-miR-3175 | CTDSP1 | NM_001206878 |
| hsa-miR-3175 | CTDSP1 | NM_021198 |
| hsa-miR-3175 | CTDSP1 | NM_182642 |
| hsa-miR-3175 | CUL2 | NM_001198777 |
| hsa-miR-3175 | CUL2 | NM_001198778 |
| hsa-miR-3175 | CUL2 | NM_001198779 |
| hsa-miR-3175 | CUL2 | NM_003591 |
| hsa-miR-3175 | CUX1 | NM_001202543 |
| hsa-miR-3175 | CUX1 | NM_181552 |
| hsa-miR-3175 | CYFIP2 | NM_001037332 |
| hsa-miR-3175 | CYFIP2 | NM_001037333 |
| hsa-miR-3175 | CYFIP2 | NM_014376 |
| hsa-miR-3175 | DLGAP3 | NM_001080418 |
| hsa-miR-3175 | DNAJB2 | NM_006736 |
| hsa-miR-3175 | DOCK2 | NM_004946 |
| hsa-miR-3175 | ELK1 | NM_001114123 |
| hsa-miR-3175 | ELK1 | NM_005229 |
| hsa-miR-3175 | FAM134C | NM_178126 |
| hsa-miR-3175 | FBXO41 | NM_001080410 |
| hsa-miR-3175 | FIGNL2 | NM_001013690 |
| hsa-miR-3175 | FOXK1 | NM_001037165 |
| hsa-miR-3175 | FOXRED2 | NM_001102371 |
| hsa-miR-3175 | FOXRED2 | NM_024955 |
| hsa-miR-3175 | FREM1 | NM_001177704 |
| hsa-miR-3175 | FREM1 | NM_144966 |
| hsa-miR-3175 | FUZ | NM_001171937 |
| hsa-miR-3175 | FUZ | NM_025129 |
| hsa-miR-3175 | GABBR1 | NM_001470 |
| hsa-miR-3175 | GABBR1 | NM_021903 |
| hsa-miR-3175 | GABBR1 | NM_021904 |
| hsa-miR-3175 | GATAD2B | NM_020699 |
| hsa-miR-3175 | GBP2 | NM_004120 |
| hsa-miR-3175 | GPD1 | NM_005276 |
| hsa-miR-3175 | HAP1 | NM_001079870 |
| hsa-miR-3175 | HAP1 | NM_001079871 |
| hsa-miR-3175 | HAP1 | NM_177977 |
| hsa-miR-3175 | HNRNPC | NM_001077442 |
| hsa-miR-3175 | HNRNPC | NM_001077443 |
| hsa-miR-3175 | HNRNPC | NM_004500 |
| hsa-miR-3175 | HNRNPC | NM_031314 |
| hsa-miR-3175 | IKZF4 | NM_022465 |
| hsa-miR-3175 | IPO9 | NM_018085 |
| hsa-miR-3175 | IQSEC2 | NM_001111125 |
| hsa-miR-3175 | IQSEC2 | NM_015075 |
| hsa-miR-3175 | ITGA10 | NM_003637 |
| hsa-miR-3175 | KDM5C | NM_001146702 |
| hsa-miR-3175 | KDM5C | NM_004187 |
| hsa-miR-3175 | KIF1B | NM_015074 |
| hsa-miR-3175 | KPTN | NM_007059 |
| hsa-miR-3175 | MAFF | NM_001161572 |
| hsa-miR-3175 | MAFF | NM_001161573 |
| hsa-miR-3175 | MAFF | NM_001161574 |
| hsa-miR-3175 | MAFF | NM_012323 |
| hsa-miR-3175 | MAP4 | NM_002375 |
| hsa-miR-3175 | MAPK8IP3 | NM_001040439 |
| hsa-miR-3175 | MAPK8IP3 | NM_015133 |
| hsa-miR-3175 | MARK2 | NM_001039469 |
| hsa-miR-3175 | MARK2 | NM_001163296 |
| hsa-miR-3175 | MARK2 | NM_001163297 |
| hsa-miR-3175 | MARK2 | NM_004954 |
| hsa-miR-3175 | MARK2 | NM_017490 |
| hsa-miR-3175 | MATN1 | NM_002379 |
| hsa-miR-3175 | MBD6 | NM_052897 |
| hsa-miR-3175 | MN1 | NM_002430 |
| hsa-miR-3175 | MRO | NM_001127174 |
| hsa-miR-3175 | MRO | NM_001127175 |
| hsa-miR-3175 | MRO | NM_001127176 |
| hsa-miR-3175 | MRO | NM_031939 |
| hsa-miR-3175 | MSN | NM_002444 |
| hsa-miR-3175 | MTPN | NM_145808 |
| hsa-miR-3175 | NACC1 | NM_052876 |
| hsa-miR-3175 | NCS1 | NM_001128826 |
| hsa-miR-3175 | NCS1 | NM_014286 |
| hsa-miR-3175 | NEUROD2 | NM_006160 |
| hsa-miR-3175 | NLGN2 | NM_020795 |
| hsa-miR-3175 | OBSL1 | NM_001173408 |
| hsa-miR-3175 | PADI2 | NM_007365 |
| hsa-miR-3175 | PCGF2 | NM_007144 |
| hsa-miR-3175 | PCP4L1 | NM_001102566 |
| hsa-miR-3175 | PDE11A | NM_001077196 |
| hsa-miR-3175 | PDE11A | NM_001077197 |
| hsa-miR-3175 | PDE11A | NM_016953 |
| hsa-miR-3175 | PDE1B | NM_000924 |
| hsa-miR-3175 | PDE1B | NM_001165975 |
| hsa-miR-3175 | PKNOX2 | NM_022062 |
| hsa-miR-3175 | PLEKHG6 | NM_001144856 |
| hsa-miR-3175 | PLEKHG6 | NM_001144857 |
| hsa-miR-3175 | PLEKHG6 | NM_018173 |
| hsa-miR-3175 | PLP1 | NM_000533 |
| hsa-miR-3175 | PLP1 | NM_001128834 |
| hsa-miR-3175 | PLP1 | NM_199478 |
| hsa-miR-3175 | PNKD | NM_015488 |
| hsa-miR-3175 | PNKD | NM_022572 |
| hsa-miR-3175 | PPP2R1A | NM_014225 |
| hsa-miR-3175 | PRKACA | NM_002730 |
| hsa-miR-3175 | PRKACA | NM_207518 |
| hsa-miR-3175 | PRX | NM_020956 |
| hsa-miR-3175 | PTMS | NM_002824 |
| hsa-miR-3175 | RAB11A | NM_001206836 |
| hsa-miR-3175 | RAB5B | NM_002868 |
| hsa-miR-3175 | RANBP10 | NM_020850 |
| hsa-miR-3175 | RARG | NM_000966 |
| hsa-miR-3175 | RARG | NM_001042728 |
| hsa-miR-3175 | RASAL2 | NM_004841 |
| hsa-miR-3175 | RASAL2 | NM_170692 |
| hsa-miR-3175 | RBM14 | NM_001198836 |
| hsa-miR-3175 | RBM14 | NM_001198837 |
| hsa-miR-3175 | RBM14 | NM_006328 |
| hsa-miR-3175 | RGS6 | NM_001204416 |
| hsa-miR-3175 | RGS6 | NM_001204419 |
| hsa-miR-3175 | RGS6 | NM_001204420 |
| hsa-miR-3175 | RGS6 | NM_001204423 |
| hsa-miR-3175 | RGS6 | NM_001204424 |
| hsa-miR-3175 | RGS6 | NM_004296 |
| hsa-miR-3175 | RNF144A | NM_014746 |
| hsa-miR-3175 | S1PR2 | NM_004230 |
| hsa-miR-3175 | SAMD8 | NM_001174156 |
| hsa-miR-3175 | SCN4A | NM_000334 |
| hsa-miR-3175 | SDK1 | NM_152744 |
| hsa-miR-3175 | SETD1A | NM_014712 |
| hsa-miR-3175 | SF1 | NM_001178031 |
| hsa-miR-3175 | SF1 | NM_004630 |
| hsa-miR-3175 | SIDT1 | NM_017699 |
| hsa-miR-3175 | SLAMF8 | NM_020125 |
| hsa-miR-3175 | SLC16A2 | NM_006517 |
| hsa-miR-3175 | SLC6A17 | NM_001010898 |
| hsa-miR-3175 | SLC8A2 | NM_015063 |
| hsa-miR-3175 | SMC1A | NM_006306 |
| hsa-miR-3175 | SORBS3 | NM_001018003 |
| hsa-miR-3175 | SORBS3 | NM_005775 |
| hsa-miR-3175 | SSBP2 | NM_012446 |
| hsa-miR-3175 | STAU1 | NM_001037328 |
| hsa-miR-3175 | STAU1 | NM_004602 |
| hsa-miR-3175 | STAU1 | NM_017452 |
| hsa-miR-3175 | STAU1 | NM_017453 |
| hsa-miR-3175 | STAU1 | NM_017454 |
| hsa-miR-3175 | SYNGAP1 | NM_006772 |
| hsa-miR-3175 | SYT6 | NM_205848 |
| hsa-miR-3175 | THTPA | NM_001126339 |
| hsa-miR-3175 | THTPA | NM_024328 |
| hsa-miR-3175 | TMEM176B | NM_001101312 |
| hsa-miR-3175 | TMEM176B | NM_001101314 |
| hsa-miR-3175 | TMEM176B | NM_014020 |
| hsa-miR-3175 | TMEM198 | NM_001005209 |
| hsa-miR-3175 | TMEM63B | NM_018426 |
| hsa-miR-3175 | TNRC6B | NM_001024843 |
| hsa-miR-3175 | TNRC6B | NM_001162501 |
| hsa-miR-3175 | TNRC6B | NM_015088 |
| hsa-miR-3175 | TOB2 | NM_016272 |
| hsa-miR-3175 | TRIM66 | NM_014818 |
| hsa-miR-3175 | TSPAN2 | NM_005725 |
| hsa-miR-3175 | TTBK1 | NM_032538 |
| hsa-miR-3175 | TTC9 | NM_015351 |
| hsa-miR-3175 | UBTF | NM_001076683 |
| hsa-miR-3175 | UBTF | NM_001076684 |
| hsa-miR-3175 | UBTF | NM_014233 |
| hsa-miR-3175 | VASH1 | NM_014909 |
| hsa-miR-3175 | WASF2 | NM_001201404 |
| hsa-miR-3175 | WASF2 | NM_006990 |
| hsa-miR-3175 | WDR48 | NM_020839 |
| hsa-miR-3175 | ZNF385A | NM_001130967 |
| hsa-miR-3175 | ZNF385A | NM_001130968 |
| hsa-miR-3175 | ZNF385A | NM_015481 |
| hsa-miR-3175 | ZNF618 | NM_133374 |
| hsa-miR-3175 | ZNF740 | NM_001004304 |
| hsa-miR-3175 | ZNRF1 | NM_032268 |
| hsa-miR-3175 | ZYG11B | NM_024646 |
| hsa-miR-3648 | AK2 | NM_001199199 |
| hsa-miR-3648 | AK2 | NM_013411 |
| hsa-miR-3648 | H2AFX | NM_002105 |
| hsa-miR-3651 | CACNA1B | NM_000718 |
| hsa-miR-3651 | CHURC1-FNTB | NM_001202558 |
| hsa-miR-3651 | CHURC1-FNTB | NM_001202559 |
| hsa-miR-3651 | DCX | NM_000555 |
| hsa-miR-3651 | DCX | NM_001195553 |
| hsa-miR-3651 | DCX | NM_178151 |
| hsa-miR-3651 | DCX | NM_178153 |
| hsa-miR-3651 | DOK4 | NM_018110 |
| hsa-miR-3651 | FAM69A | NM_001006605 |
| hsa-miR-3651 | FBXO34 | NM_017943 |
| hsa-miR-3651 | FNTB | NM_002028 |
| hsa-miR-3651 | FRMD3 | NM_174938 |
| hsa-miR-3651 | IKZF2 | NM_001079526 |
| hsa-miR-3651 | IKZF2 | NM_016260 |
| hsa-miR-3651 | ITSN2 | NM_006277 |
| hsa-miR-3651 | ITSN2 | NM_019595 |
| hsa-miR-3651 | LYN | NM_001111097 |
| hsa-miR-3651 | LYN | NM_002350 |
| hsa-miR-3651 | NBEA | NM_001204197 |
| hsa-miR-3651 | NBEA | NM_015678 |
| hsa-miR-3651 | PIM3 | NM_001001852 |
| hsa-miR-3651 | PRICKLE1 | NM_001144881 |
| hsa-miR-3651 | PRICKLE1 | NM_001144882 |
| hsa-miR-3651 | PRICKLE1 | NM_001144883 |
| hsa-miR-3651 | PRICKLE1 | NM_153026 |
| hsa-miR-3651 | PRKCA | NM_002737 |
| hsa-miR-3651 | SEC23IP | NM_007190 |
| hsa-miR-3651 | SEC63 | NM_007214 |
| hsa-miR-3651 | TSC22D2 | NM_014779 |
| hsa-miR-3651 | USP13 | NM_003940 |
| hsa-miR-3651 | VGLL3 | NM_016206 |
| hsa-miR-3687 | NCS1 | NM_001128826 |
| hsa-miR-3687 | NCS1 | NM_014286 |
| hsa-miR-3687 | PSD | NM_002779 |
| hsa-miR-378a-3p | AAK1 | NM_014911 |
| hsa-miR-378a-3p | ADD2 | NM_001185054 |
| hsa-miR-378a-3p | ADD2 | NM_001617 |
| hsa-miR-378a-3p | ADD2 | NM_017488 |
| hsa-miR-378a-3p | ALPK3 | NM_020778 |
| hsa-miR-378a-3p | ANGPT4 | NM_015985 |
| hsa-miR-378a-3p | ATPIF1 | NM_178191 |
| hsa-miR-378a-3p | BMP2 | NM_001200 |
| hsa-miR-378a-3p | C16orf72 | NM_014117 |
| hsa-miR-378a-3p | C17orf72 | NM_001191029 |
| hsa-miR-378a-3p | CACNA2D4 | NM_172364 |
| hsa-miR-378a-3p | CAMKK2 | NM_006549 |
| hsa-miR-378a-3p | CAMKK2 | NM_153499 |
| hsa-miR-378a-3p | CAMKK2 | NM_153500 |
| hsa-miR-378a-3p | CAMKK2 | NM_172216 |
| hsa-miR-378a-3p | CAMKK2 | NM_172226 |
| hsa-miR-378a-3p | CBL | NM_005188 |
| hsa-miR-378a-3p | CDC40 | NM_015891 |
| hsa-miR-378a-3p | CHIT1 | NM_003465 |
| hsa-miR-378a-3p | DCAF12 | NM_015397 |
| hsa-miR-378a-3p | DCX | NM_000555 |
| hsa-miR-378a-3p | DCX | NM_001195553 |
| hsa-miR-378a-3p | DCX | NM_178151 |
| hsa-miR-378a-3p | DCX | NM_178153 |
| hsa-miR-378a-3p | DUSP8 | NM_004420 |
| hsa-miR-378a-3p | EFNA5 | NM_001962 |
| hsa-miR-378a-3p | GOLT1A | NM_198447 |
| hsa-miR-378a-3p | GPM6B | NM_001001995 |
| hsa-miR-378a-3p | H3F3B | NM_005324 |
| hsa-miR-378a-3p | HDAC4 | NM_006037 |
| hsa-miR-378a-3p | HSPA12A | NM_025015 |
| hsa-miR-378a-3p | IGF1R | NM_000875 |
| hsa-miR-378a-3p | IPO9 | NM_018085 |
| hsa-miR-378a-3p | IQSEC2 | NM_001111125 |
| hsa-miR-378a-3p | IQSEC2 | NM_015075 |
| hsa-miR-378a-3p | KCNIP2 | NM_014591 |
| hsa-miR-378a-3p | KCNIP2 | NM_173191 |
| hsa-miR-378a-3p | KCNIP2 | NM_173192 |
| hsa-miR-378a-3p | KCNIP2 | NM_173193 |
| hsa-miR-378a-3p | KCNIP2 | NM_173194 |
| hsa-miR-378a-3p | KCNIP2 | NM_173195 |
| hsa-miR-378a-3p | KIAA1467 | NM_020853 |
| hsa-miR-378a-3p | KIAA1522 | NM_001198972 |
| hsa-miR-378a-3p | KIAA1522 | NM_001198973 |
| hsa-miR-378a-3p | KIAA1522 | NM_020888 |
| hsa-miR-378a-3p | KSR1 | NM_014238 |
| hsa-miR-378a-3p | LBX2 | NM_001009812 |
| hsa-miR-378a-3p | MAFG | NM_002359 |
| hsa-miR-378a-3p | MAFG | NM_032711 |
| hsa-miR-378a-3p | MAPK1IP1L | NM_144578 |
| hsa-miR-378a-3p | MED12L | NM_053002 |
| hsa-miR-378a-3p | METTL4 | NM_022840 |
| hsa-miR-378a-3p | MREG | NM_018000 |
| hsa-miR-378a-3p | NEK4 | NM_001193533 |
| hsa-miR-378a-3p | NEK4 | NM_003157 |
| hsa-miR-378a-3p | NSFL1C | NM_016143 |
| hsa-miR-378a-3p | NSFL1C | NM_018839 |
| hsa-miR-378a-3p | NTRK3 | NM_001007156 |
| hsa-miR-378a-3p | OTUB2 | NM_023112 |
| hsa-miR-378a-3p | PAPD5 | NM_001040284 |
| hsa-miR-378a-3p | PAPD5 | NM_001040285 |
| hsa-miR-378a-3p | PAPOLA | NM_032632 |
| hsa-miR-378a-3p | PAPPA | NM_002581 |
| hsa-miR-378a-3p | PARVA | NM_018222 |
| hsa-miR-378a-3p | PAX8 | NM_003466 |
| hsa-miR-378a-3p | PAX8 | NM_013952 |
| hsa-miR-378a-3p | PAX8 | NM_013953 |
| hsa-miR-378a-3p | PAX8 | NM_013992 |
| hsa-miR-378a-3p | PHC3 | NM_024947 |
| hsa-miR-378a-3p | PLEKHG2 | NM_022835 |
| hsa-miR-378a-3p | PSMA5 | NM_001199772 |
| hsa-miR-378a-3p | PSMA5 | NM_001199773 |
| hsa-miR-378a-3p | PSMA5 | NM_001199774 |
| hsa-miR-378a-3p | PSMA5 | NM_002790 |
| hsa-miR-378a-3p | QSER1 | NM_001076786 |
| hsa-miR-378a-3p | RBMS1 | NM_002897 |
| hsa-miR-378a-3p | RBMS1 | NM_016836 |
| hsa-miR-378a-3p | RIMS4 | NM_001205317 |
| hsa-miR-378a-3p | RIMS4 | NM_182970 |
| hsa-miR-378a-3p | RRP1B | NM_015056 |
| hsa-miR-378a-3p | SBDS | NM_016038 |
| hsa-miR-378a-3p | SCN5A | NM_000335 |
| hsa-miR-378a-3p | SCN5A | NM_001099404 |
| hsa-miR-378a-3p | SCN5A | NM_001099405 |
| hsa-miR-378a-3p | SCN5A | NM_001160160 |
| hsa-miR-378a-3p | SCN5A | NM_001160161 |
| hsa-miR-378a-3p | SCN5A | NM_198056 |
| hsa-miR-378a-3p | SFT2D3 | NM_032740 |
| hsa-miR-378a-3p | SLC2A1 | NM_006516 |
| hsa-miR-378a-3p | SLC38A1 | NM_001077484 |
| hsa-miR-378a-3p | SLC38A1 | NM_030674 |
| hsa-miR-378a-3p | SLC39A9 | NM_018375 |
| hsa-miR-378a-3p | SOX7 | NM_031439 |
| hsa-miR-378a-3p | SPEG | NM_001173476 |
| hsa-miR-378a-3p | SRSF3 | NM_003017 |
| hsa-miR-378a-3p | SSH2 | NM_033389 |
| hsa-miR-378a-3p | SULF1 | NM_001128204 |
| hsa-miR-378a-3p | SULF1 | NM_001128205 |
| hsa-miR-378a-3p | SULF1 | NM_001128206 |
| hsa-miR-378a-3p | SULF1 | NM_015170 |
| hsa-miR-378a-3p | TMEM129 | NM_001127266 |
| hsa-miR-378a-3p | TMEM129 | NM_138385 |
| hsa-miR-378a-3p | TRAF3 | NM_001199427 |
| hsa-miR-378a-3p | TRAF3 | NM_003300 |
| hsa-miR-378a-3p | TRAF3 | NM_145725 |
| hsa-miR-378a-3p | TRAF3 | NM_145726 |
| hsa-miR-378a-3p | TSPAN17 | NM_001006616 |
| hsa-miR-378a-3p | UBE2W | NM_001001481 |
| hsa-miR-378a-3p | UBE2W | NM_018299 |
| hsa-miR-378a-3p | VANGL1 | NM_001172411 |
| hsa-miR-378a-3p | VANGL1 | NM_001172412 |
| hsa-miR-378a-3p | VANGL1 | NM_138959 |
| hsa-miR-378a-3p | VAT1 | NM_006373 |
| hsa-miR-378a-3p | XPO5 | NM_020750 |
| hsa-miR-378a-3p | ZDHHC9 | NM_001008222 |
| hsa-miR-378a-3p | ZDHHC9 | NM_016032 |
| hsa-miR-378a-3p | ZNF507 | NM_001136156 |
| hsa-miR-378a-3p | ZNF507 | NM_014910 |
| hsa-miR-378a-3p | ZNF652 | NM_001145365 |
| hsa-miR-378a-3p | ZNF652 | NM_014897 |
| hsa-miR-378a-3p | ZNF805 | NM_001023563 |
| hsa-miR-378a-3p | ZNF805 | NM_001145078 |
| hsa-miR-378c | AAK1 | NM_014911 |
| hsa-miR-378c | ADD2 | NM_001185054 |
| hsa-miR-378c | ADD2 | NM_001617 |
| hsa-miR-378c | ADD2 | NM_017488 |
| hsa-miR-378c | ALPK3 | NM_020778 |
| hsa-miR-378c | ANGPT4 | NM_015985 |
| hsa-miR-378c | ATPIF1 | NM_178191 |
| hsa-miR-378c | BMP2 | NM_001200 |
| hsa-miR-378c | C11orf49 | NM_001003678 |
| hsa-miR-378c | C16orf72 | NM_014117 |
| hsa-miR-378c | C17orf72 | NM_001191029 |
| hsa-miR-378c | CACNA2D4 | NM_172364 |
| hsa-miR-378c | CAMKK2 | NM_006549 |
| hsa-miR-378c | CAMKK2 | NM_153499 |
| hsa-miR-378c | CAMKK2 | NM_153500 |
| hsa-miR-378c | CAMKK2 | NM_172216 |
| hsa-miR-378c | CAMKK2 | NM_172226 |
| hsa-miR-378c | CBL | NM_005188 |
| hsa-miR-378c | CDC40 | NM_015891 |
| hsa-miR-378c | CEP44 | NM_001145314 |
| hsa-miR-378c | CHIT1 | NM_003465 |
| hsa-miR-378c | DACT1 | NM_001079520 |
| hsa-miR-378c | DACT1 | NM_016651 |
| hsa-miR-378c | DCAF12 | NM_015397 |
| hsa-miR-378c | DCX | NM_000555 |
| hsa-miR-378c | DCX | NM_001195553 |
| hsa-miR-378c | DCX | NM_178151 |
| hsa-miR-378c | DCX | NM_178153 |
| hsa-miR-378c | DUSP8 | NM_004420 |
| hsa-miR-378c | DYRK1A | NM_001396 |
| hsa-miR-378c | DYRK1A | NM_101395 |
| hsa-miR-378c | DYRK1A | NM_130436 |
| hsa-miR-378c | DYRK1A | NM_130438 |
| hsa-miR-378c | EFNA5 | NM_001962 |
| hsa-miR-378c | FBXL20 | NM_001184906 |
| hsa-miR-378c | FBXL20 | NM_032875 |
| hsa-miR-378c | FRMPD4 | NM_014728 |
| hsa-miR-378c | FZD5 | NM_003468 |
| hsa-miR-378c | GLS | NM_014905 |
| hsa-miR-378c | GOLT1A | NM_198447 |
| hsa-miR-378c | GPM6B | NM_001001995 |
| hsa-miR-378c | H3F3B | NM_005324 |
| hsa-miR-378c | HDAC4 | NM_006037 |
| hsa-miR-378c | HSPA12A | NM_025015 |
| hsa-miR-378c | IGF1R | NM_000875 |
| hsa-miR-378c | IPO9 | NM_018085 |
| hsa-miR-378c | IQSEC2 | NM_001111125 |
| hsa-miR-378c | IQSEC2 | NM_015075 |
| hsa-miR-378c | KCND1 | NM_004979 |
| hsa-miR-378c | KCNIP2 | NM_014591 |
| hsa-miR-378c | KCNIP2 | NM_173191 |
| hsa-miR-378c | KCNIP2 | NM_173192 |
| hsa-miR-378c | KCNIP2 | NM_173193 |
| hsa-miR-378c | KCNIP2 | NM_173194 |
| hsa-miR-378c | KCNIP2 | NM_173195 |
| hsa-miR-378c | KIAA1467 | NM_020853 |
| hsa-miR-378c | KIAA1522 | NM_001198972 |
| hsa-miR-378c | KIAA1522 | NM_001198973 |
| hsa-miR-378c | KIAA1522 | NM_020888 |
| hsa-miR-378c | KSR1 | NM_014238 |
| hsa-miR-378c | LBX2 | NM_001009812 |
| hsa-miR-378c | MAFG | NM_002359 |
| hsa-miR-378c | MAFG | NM_032711 |
| hsa-miR-378c | MAPK1IP1L | NM_144578 |
| hsa-miR-378c | MED12L | NM_053002 |
| hsa-miR-378c | METTL4 | NM_022840 |
| hsa-miR-378c | MREG | NM_018000 |
| hsa-miR-378c | NEK4 | NM_001193533 |
| hsa-miR-378c | NEK4 | NM_003157 |
| hsa-miR-378c | NSFL1C | NM_016143 |
| hsa-miR-378c | NSFL1C | NM_018839 |
| hsa-miR-378c | NTRK3 | NM_001007156 |
| hsa-miR-378c | OTUB2 | NM_023112 |
| hsa-miR-378c | PAG1 | NM_018440 |
| hsa-miR-378c | PAPD5 | NM_001040284 |
| hsa-miR-378c | PAPD5 | NM_001040285 |
| hsa-miR-378c | PAPOLA | NM_032632 |
| hsa-miR-378c | PAPPA | NM_002581 |
| hsa-miR-378c | PARVA | NM_018222 |
| hsa-miR-378c | PAX8 | NM_003466 |
| hsa-miR-378c | PAX8 | NM_013952 |
| hsa-miR-378c | PAX8 | NM_013953 |
| hsa-miR-378c | PAX8 | NM_013992 |
| hsa-miR-378c | PHC3 | NM_024947 |
| hsa-miR-378c | PLEKHG2 | NM_022835 |
| hsa-miR-378c | PSMA5 | NM_001199772 |
| hsa-miR-378c | PSMA5 | NM_001199773 |
| hsa-miR-378c | PSMA5 | NM_001199774 |
| hsa-miR-378c | PSMA5 | NM_002790 |
| hsa-miR-378c | QSER1 | NM_001076786 |
| hsa-miR-378c | RBMS1 | NM_002897 |
| hsa-miR-378c | RBMS1 | NM_016836 |
| hsa-miR-378c | RIMS4 | NM_001205317 |
| hsa-miR-378c | RIMS4 | NM_182970 |
| hsa-miR-378c | RRP1B | NM_015056 |
| hsa-miR-378c | SBDS | NM_016038 |
| hsa-miR-378c | SCN5A | NM_000335 |
| hsa-miR-378c | SCN5A | NM_001099404 |
| hsa-miR-378c | SCN5A | NM_001099405 |
| hsa-miR-378c | SCN5A | NM_001160160 |
| hsa-miR-378c | SCN5A | NM_001160161 |
| hsa-miR-378c | SCN5A | NM_198056 |
| hsa-miR-378c | SFT2D3 | NM_032740 |
| hsa-miR-378c | SLC2A1 | NM_006516 |
| hsa-miR-378c | SLC38A1 | NM_001077484 |
| hsa-miR-378c | SLC38A1 | NM_030674 |
| hsa-miR-378c | SLC39A9 | NM_018375 |
| hsa-miR-378c | SOX7 | NM_031439 |
| hsa-miR-378c | SPEG | NM_001173476 |
| hsa-miR-378c | SPOPL | NM_001001664 |
| hsa-miR-378c | SRSF3 | NM_003017 |
| hsa-miR-378c | SSH2 | NM_033389 |
| hsa-miR-378c | SULF1 | NM_001128204 |
| hsa-miR-378c | SULF1 | NM_001128205 |
| hsa-miR-378c | SULF1 | NM_001128206 |
| hsa-miR-378c | SULF1 | NM_015170 |
| hsa-miR-378c | TMCO1 | NM_019026 |
| hsa-miR-378c | TMEM129 | NM_001127266 |
| hsa-miR-378c | TMEM129 | NM_138385 |
| hsa-miR-378c | TOB2 | NM_016272 |
| hsa-miR-378c | TRAF3 | NM_001199427 |
| hsa-miR-378c | TRAF3 | NM_003300 |
| hsa-miR-378c | TRAF3 | NM_145725 |
| hsa-miR-378c | TRAF3 | NM_145726 |
| hsa-miR-378c | TSPAN17 | NM_001006616 |
| hsa-miR-378c | UBE2W | NM_001001481 |
| hsa-miR-378c | UBE2W | NM_018299 |
| hsa-miR-378c | VANGL1 | NM_001172411 |
| hsa-miR-378c | VANGL1 | NM_001172412 |
| hsa-miR-378c | VANGL1 | NM_138959 |
| hsa-miR-378c | VAT1 | NM_006373 |
| hsa-miR-378c | XPO5 | NM_020750 |
| hsa-miR-378c | ZDHHC9 | NM_001008222 |
| hsa-miR-378c | ZDHHC9 | NM_016032 |
| hsa-miR-378c | ZNF507 | NM_001136156 |
| hsa-miR-378c | ZNF507 | NM_014910 |
| hsa-miR-378c | ZNF652 | NM_001145365 |
| hsa-miR-378c | ZNF652 | NM_014897 |
| hsa-miR-378c | ZNF71 | NM_021216 |
| hsa-miR-378c | ZNF805 | NM_001023563 |
| hsa-miR-378c | ZNF805 | NM_001145078 |
| hsa-miR-378f | AAK1 | NM_014911 |
| hsa-miR-378f | ADD2 | NM_001185054 |
| hsa-miR-378f | ADD2 | NM_001617 |
| hsa-miR-378f | ALPK3 | NM_020778 |
| hsa-miR-378f | ATPIF1 | NM_178191 |
| hsa-miR-378f | BMP2 | NM_001200 |
| hsa-miR-378f | C17orf72 | NM_001164257 |
| hsa-miR-378f | C17orf72 | NM_001191029 |
| hsa-miR-378f | C17orf72 | NM_001191030 |
| hsa-miR-378f | C17orf72 | NM_001191031 |
| hsa-miR-378f | CACNA2D4 | NM_172364 |
| hsa-miR-378f | CAMKK2 | NM_006549 |
| hsa-miR-378f | CAMKK2 | NM_153499 |
| hsa-miR-378f | CAMKK2 | NM_153500 |
| hsa-miR-378f | CAMKK2 | NM_172216 |
| hsa-miR-378f | CAMKK2 | NM_172226 |
| hsa-miR-378f | CDC40 | NM_015891 |
| hsa-miR-378f | CHIT1 | NM_003465 |
| hsa-miR-378f | DCX | NM_000555 |
| hsa-miR-378f | DCX | NM_001195553 |
| hsa-miR-378f | DCX | NM_178151 |
| hsa-miR-378f | DCX | NM_178153 |
| hsa-miR-378f | EFNA5 | NM_001962 |
| hsa-miR-378f | FKBP5 | NM_001145775 |
| hsa-miR-378f | FKBP5 | NM_001145776 |
| hsa-miR-378f | FKBP5 | NM_004117 |
| hsa-miR-378f | GOLT1A | NM_198447 |
| hsa-miR-378f | GPM6B | NM_001001995 |
| hsa-miR-378f | HSPA12A | NM_025015 |
| hsa-miR-378f | IGF1R | NM_000875 |
| hsa-miR-378f | IPO9 | NM_018085 |
| hsa-miR-378f | IQSEC2 | NM_001111125 |
| hsa-miR-378f | IQSEC2 | NM_015075 |
| hsa-miR-378f | KCNIP2 | NM_014591 |
| hsa-miR-378f | KCNIP2 | NM_173191 |
| hsa-miR-378f | KCNIP2 | NM_173192 |
| hsa-miR-378f | KCNIP2 | NM_173193 |
| hsa-miR-378f | KCNIP2 | NM_173194 |
| hsa-miR-378f | KCNIP2 | NM_173195 |
| hsa-miR-378f | KIAA1467 | NM_020853 |
| hsa-miR-378f | KLF15 | NM_014079 |
| hsa-miR-378f | KSR1 | NM_014238 |
| hsa-miR-378f | LBX2 | NM_001009812 |
| hsa-miR-378f | MAFG | NM_002359 |
| hsa-miR-378f | MAFG | NM_032711 |
| hsa-miR-378f | MAPK1IP1L | NM_144578 |
| hsa-miR-378f | MED12L | NM_053002 |
| hsa-miR-378f | MPHOSPH8 | NM_017520 |
| hsa-miR-378f | OTUB2 | NM_023112 |
| hsa-miR-378f | PAPD5 | NM_001040284 |
| hsa-miR-378f | PAPD5 | NM_001040285 |
| hsa-miR-378f | PAPPA | NM_002581 |
| hsa-miR-378f | PARVA | NM_018222 |
| hsa-miR-378f | PDE1B | NM_000924 |
| hsa-miR-378f | PDE1B | NM_001165975 |
| hsa-miR-378f | RIMS4 | NM_001205317 |
| hsa-miR-378f | RIMS4 | NM_182970 |
| hsa-miR-378f | SCN5A | NM_000335 |
| hsa-miR-378f | SCN5A | NM_001099404 |
| hsa-miR-378f | SCN5A | NM_001099405 |
| hsa-miR-378f | SCN5A | NM_001160160 |
| hsa-miR-378f | SCN5A | NM_001160161 |
| hsa-miR-378f | SCN5A | NM_198056 |
| hsa-miR-378f | SLC2A1 | NM_006516 |
| hsa-miR-378f | SLC38A1 | NM_001077484 |
| hsa-miR-378f | SLC38A1 | NM_030674 |
| hsa-miR-378f | SRSF3 | NM_003017 |
| hsa-miR-378f | TMEM129 | NM_001127266 |
| hsa-miR-378f | TMEM129 | NM_138385 |
| hsa-miR-378f | TRAF3 | NM_001199427 |
| hsa-miR-378f | TRAF3 | NM_003300 |
| hsa-miR-378f | TRAF3 | NM_145725 |
| hsa-miR-378f | TRAF3 | NM_145726 |
| hsa-miR-378f | UBE2W | NM_001001481 |
| hsa-miR-378f | UBE2W | NM_018299 |
| hsa-miR-378f | VANGL1 | NM_001172411 |
| hsa-miR-378f | VANGL1 | NM_001172412 |
| hsa-miR-378f | VANGL1 | NM_138959 |
| hsa-miR-378f | VAT1 | NM_006373 |
| hsa-miR-378f | XPO5 | NM_020750 |
| hsa-miR-378f | ZNF507 | NM_001136156 |
| hsa-miR-378f | ZNF507 | NM_014910 |
| hsa-miR-378f | ZNF652 | NM_001145365 |
| hsa-miR-378f | ZNF652 | NM_014897 |
| hsa-miR-378f | ZNF805 | NM_001023563 |
| hsa-miR-378f | ZNF805 | NM_001145078 |
| hsa-miR-378i | AAK1 | NM_014911 |
| hsa-miR-378i | ADD2 | NM_001185054 |
| hsa-miR-378i | ADD2 | NM_001617 |
| hsa-miR-378i | ADD2 | NM_017488 |
| hsa-miR-378i | ALPK3 | NM_020778 |
| hsa-miR-378i | ATPIF1 | NM_178191 |
| hsa-miR-378i | C16orf72 | NM_014117 |
| hsa-miR-378i | C17orf72 | NM_001191029 |
| hsa-miR-378i | CACNA2D4 | NM_172364 |
| hsa-miR-378i | CAMKK2 | NM_006549 |
| hsa-miR-378i | CAMKK2 | NM_153499 |
| hsa-miR-378i | CAMKK2 | NM_153500 |
| hsa-miR-378i | CAMKK2 | NM_172216 |
| hsa-miR-378i | CAMKK2 | NM_172226 |
| hsa-miR-378i | CBX5 | NM_001127321 |
| hsa-miR-378i | CBX5 | NM_001127322 |
| hsa-miR-378i | CBX5 | NM_012117 |
| hsa-miR-378i | CDC40 | NM_015891 |
| hsa-miR-378i | CHIT1 | NM_003465 |
| hsa-miR-378i | DUSP8 | NM_004420 |
| hsa-miR-378i | EIF4G3 | NM_001198801 |
| hsa-miR-378i | EIF4G3 | NM_001198802 |
| hsa-miR-378i | EIF4G3 | NM_003760 |
| hsa-miR-378i | GLS | NM_014905 |
| hsa-miR-378i | GPM6B | NM_001001995 |
| hsa-miR-378i | HSPA12A | NM_025015 |
| hsa-miR-378i | IGF1R | NM_000875 |
| hsa-miR-378i | IPO9 | NM_018085 |
| hsa-miR-378i | IQSEC2 | NM_001111125 |
| hsa-miR-378i | IQSEC2 | NM_015075 |
| hsa-miR-378i | KCNIP2 | NM_014591 |
| hsa-miR-378i | KCNIP2 | NM_173191 |
| hsa-miR-378i | KCNIP2 | NM_173192 |
| hsa-miR-378i | KCNIP2 | NM_173193 |
| hsa-miR-378i | KCNIP2 | NM_173194 |
| hsa-miR-378i | KCNIP2 | NM_173195 |
| hsa-miR-378i | KIAA1467 | NM_020853 |
| hsa-miR-378i | KSR1 | NM_014238 |
| hsa-miR-378i | LBX2 | NM_001009812 |
| hsa-miR-378i | MAFG | NM_002359 |
| hsa-miR-378i | MAFG | NM_032711 |
| hsa-miR-378i | MAPK1IP1L | NM_144578 |
| hsa-miR-378i | MED12L | NM_053002 |
| hsa-miR-378i | OTUB2 | NM_023112 |
| hsa-miR-378i | PAG1 | NM_018440 |
| hsa-miR-378i | PAPD5 | NM_001040284 |
| hsa-miR-378i | PAPD5 | NM_001040285 |
| hsa-miR-378i | PAPPA | NM_002581 |
| hsa-miR-378i | PARVA | NM_018222 |
| hsa-miR-378i | PDIA4 | NM_004911 |
| hsa-miR-378i | PHC3 | NM_024947 |
| hsa-miR-378i | PSMA5 | NM_001199772 |
| hsa-miR-378i | PSMA5 | NM_001199773 |
| hsa-miR-378i | PSMA5 | NM_001199774 |
| hsa-miR-378i | PSMA5 | NM_002790 |
| hsa-miR-378i | QSER1 | NM_001076786 |
| hsa-miR-378i | REST | NM_001193508 |
| hsa-miR-378i | REST | NM_005612 |
| hsa-miR-378i | RIMS4 | NM_001205317 |
| hsa-miR-378i | RIMS4 | NM_182970 |
| hsa-miR-378i | SCN5A | NM_000335 |
| hsa-miR-378i | SCN5A | NM_001099404 |
| hsa-miR-378i | SCN5A | NM_001099405 |
| hsa-miR-378i | SCN5A | NM_001160160 |
| hsa-miR-378i | SCN5A | NM_001160161 |
| hsa-miR-378i | SCN5A | NM_198056 |
| hsa-miR-378i | SFT2D3 | NM_032740 |
| hsa-miR-378i | SH3TC2 | NM_024577 |
| hsa-miR-378i | SLC38A1 | NM_001077484 |
| hsa-miR-378i | SLC38A1 | NM_030674 |
| hsa-miR-378i | SLC39A9 | NM_018375 |
| hsa-miR-378i | SOX7 | NM_031439 |
| hsa-miR-378i | SRSF3 | NM_003017 |
| hsa-miR-378i | TLK2 | NM_006852 |
| hsa-miR-378i | TMCO1 | NM_019026 |
| hsa-miR-378i | TMEM129 | NM_001127266 |
| hsa-miR-378i | TMEM129 | NM_138385 |
| hsa-miR-378i | TOB2 | NM_016272 |
| hsa-miR-378i | TRAF3 | NM_001199427 |
| hsa-miR-378i | TRAF3 | NM_003300 |
| hsa-miR-378i | TRAF3 | NM_145725 |
| hsa-miR-378i | TRAF3 | NM_145726 |
| hsa-miR-378i | UBE2W | NM_001001481 |
| hsa-miR-378i | UBE2W | NM_018299 |
| hsa-miR-378i | VANGL1 | NM_001172411 |
| hsa-miR-378i | VANGL1 | NM_001172412 |
| hsa-miR-378i | VANGL1 | NM_138959 |
| hsa-miR-378i | ZDHHC9 | NM_001008222 |
| hsa-miR-378i | ZDHHC9 | NM_016032 |
| hsa-miR-378i | ZNF507 | NM_001136156 |
| hsa-miR-378i | ZNF507 | NM_014910 |
| hsa-miR-378i | ZNF652 | NM_001145365 |
| hsa-miR-378i | ZNF652 | NM_014897 |
| hsa-miR-378i | ZNF805 | NM_001023563 |
| hsa-miR-378i | ZNF805 | NM_001145078 |
| hsa-miR-422a | AAK1 | NM_014911 |
| hsa-miR-422a | ADD2 | NM_001185054 |
| hsa-miR-422a | ADD2 | NM_001617 |
| hsa-miR-422a | ADD2 | NM_017488 |
| hsa-miR-422a | ALPK3 | NM_020778 |
| hsa-miR-422a | ANGPT4 | NM_015985 |
| hsa-miR-422a | ATPIF1 | NM_178191 |
| hsa-miR-422a | C16orf72 | NM_014117 |
| hsa-miR-422a | C17orf72 | NM_001191029 |
| hsa-miR-422a | CACNA2D4 | NM_172364 |
| hsa-miR-422a | CAMKK2 | NM_006549 |
| hsa-miR-422a | CAMKK2 | NM_153499 |
| hsa-miR-422a | CAMKK2 | NM_153500 |
| hsa-miR-422a | CAMKK2 | NM_172216 |
| hsa-miR-422a | CAMKK2 | NM_172226 |
| hsa-miR-422a | CBL | NM_005188 |
| hsa-miR-422a | CDC40 | NM_015891 |
| hsa-miR-422a | CEP44 | NM_001145314 |
| hsa-miR-422a | CHIT1 | NM_003465 |
| hsa-miR-422a | CRLF3 | NM_015986 |
| hsa-miR-422a | DCAF12 | NM_015397 |
| hsa-miR-422a | DUSP8 | NM_004420 |
| hsa-miR-422a | EFNA5 | NM_001962 |
| hsa-miR-422a | FKBP5 | NM_001145775 |
| hsa-miR-422a | FKBP5 | NM_001145776 |
| hsa-miR-422a | FKBP5 | NM_004117 |
| hsa-miR-422a | FRMPD4 | NM_014728 |
| hsa-miR-422a | GLS | NM_014905 |
| hsa-miR-422a | GOLT1A | NM_198447 |
| hsa-miR-422a | GPM6B | NM_001001995 |
| hsa-miR-422a | HSPA12A | NM_025015 |
| hsa-miR-422a | IGF1R | NM_000875 |
| hsa-miR-422a | IPO9 | NM_018085 |
| hsa-miR-422a | IQSEC2 | NM_001111125 |
| hsa-miR-422a | IQSEC2 | NM_015075 |
| hsa-miR-422a | KCNIP2 | NM_014591 |
| hsa-miR-422a | KCNIP2 | NM_173191 |
| hsa-miR-422a | KCNIP2 | NM_173192 |
| hsa-miR-422a | KCNIP2 | NM_173193 |
| hsa-miR-422a | KCNIP2 | NM_173194 |
| hsa-miR-422a | KCNIP2 | NM_173195 |
| hsa-miR-422a | KIAA1467 | NM_020853 |
| hsa-miR-422a | KIAA1522 | NM_001198972 |
| hsa-miR-422a | KIAA1522 | NM_001198973 |
| hsa-miR-422a | KIAA1522 | NM_020888 |
| hsa-miR-422a | KPNA6 | NM_012316 |
| hsa-miR-422a | KSR1 | NM_014238 |
| hsa-miR-422a | LBX2 | NM_001009812 |
| hsa-miR-422a | MAFG | NM_002359 |
| hsa-miR-422a | MAFG | NM_032711 |
| hsa-miR-422a | MAPK1IP1L | NM_144578 |
| hsa-miR-422a | MED12L | NM_053002 |
| hsa-miR-422a | METTL4 | NM_022840 |
| hsa-miR-422a | MKL2 | NM_014048 |
| hsa-miR-422a | MPHOSPH8 | NM_017520 |
| hsa-miR-422a | MREG | NM_018000 |
| hsa-miR-422a | NEK4 | NM_001193533 |
| hsa-miR-422a | NEK4 | NM_003157 |
| hsa-miR-422a | NSFL1C | NM_016143 |
| hsa-miR-422a | NSFL1C | NM_018839 |
| hsa-miR-422a | NTRK3 | NM_001007156 |
| hsa-miR-422a | OTUB2 | NM_023112 |
| hsa-miR-422a | PAPD5 | NM_001040284 |
| hsa-miR-422a | PAPD5 | NM_001040285 |
| hsa-miR-422a | PAPOLA | NM_032632 |
| hsa-miR-422a | PAPPA | NM_002581 |
| hsa-miR-422a | PARVA | NM_018222 |
| hsa-miR-422a | PAX8 | NM_003466 |
| hsa-miR-422a | PAX8 | NM_013952 |
| hsa-miR-422a | PAX8 | NM_013953 |
| hsa-miR-422a | PAX8 | NM_013992 |
| hsa-miR-422a | PDE1B | NM_000924 |
| hsa-miR-422a | PDE1B | NM_001165975 |
| hsa-miR-422a | PLEKHG2 | NM_022835 |
| hsa-miR-422a | PSMA5 | NM_001199772 |
| hsa-miR-422a | PSMA5 | NM_001199773 |
| hsa-miR-422a | PSMA5 | NM_001199774 |
| hsa-miR-422a | PSMA5 | NM_002790 |
| hsa-miR-422a | RASGRF1 | NM_001145648 |
| hsa-miR-422a | RASGRF1 | NM_002891 |
| hsa-miR-422a | RASGRF1 | NM_153815 |
| hsa-miR-422a | RRP1B | NM_015056 |
| hsa-miR-422a | SBDS | NM_016038 |
| hsa-miR-422a | SCN5A | NM_000335 |
| hsa-miR-422a | SCN5A | NM_001099404 |
| hsa-miR-422a | SCN5A | NM_001099405 |
| hsa-miR-422a | SCN5A | NM_001160160 |
| hsa-miR-422a | SCN5A | NM_001160161 |
| hsa-miR-422a | SCN5A | NM_198056 |
| hsa-miR-422a | SFT2D3 | NM_032740 |
| hsa-miR-422a | SLC2A1 | NM_006516 |
| hsa-miR-422a | SLC38A1 | NM_001077484 |
| hsa-miR-422a | SLC38A1 | NM_030674 |
| hsa-miR-422a | SLC39A9 | NM_018375 |
| hsa-miR-422a | SOX7 | NM_031439 |
| hsa-miR-422a | SPEG | NM_001173476 |
| hsa-miR-422a | SRSF3 | NM_003017 |
| hsa-miR-422a | SULF1 | NM_001128204 |
| hsa-miR-422a | SULF1 | NM_001128205 |
| hsa-miR-422a | SULF1 | NM_001128206 |
| hsa-miR-422a | SULF1 | NM_015170 |
| hsa-miR-422a | TMCO1 | NM_019026 |
| hsa-miR-422a | TMEM129 | NM_001127266 |
| hsa-miR-422a | TMEM129 | NM_138385 |
| hsa-miR-422a | TRAF3 | NM_001199427 |
| hsa-miR-422a | TRAF3 | NM_003300 |
| hsa-miR-422a | TRAF3 | NM_145725 |
| hsa-miR-422a | TRAF3 | NM_145726 |
| hsa-miR-422a | VANGL1 | NM_001172411 |
| hsa-miR-422a | VANGL1 | NM_001172412 |
| hsa-miR-422a | VANGL1 | NM_138959 |
| hsa-miR-422a | VAT1 | NM_006373 |
| hsa-miR-422a | XPO5 | NM_020750 |
| hsa-miR-422a | ZDHHC9 | NM_001008222 |
| hsa-miR-422a | ZDHHC9 | NM_016032 |
| hsa-miR-422a | ZNF507 | NM_001136156 |
| hsa-miR-422a | ZNF507 | NM_014910 |
| hsa-miR-422a | ZNF652 | NM_001145365 |
| hsa-miR-422a | ZNF652 | NM_014897 |
| hsa-miR-422a | ZNF71 | NM_021216 |
| hsa-miR-422a | ZNF805 | NM_001023563 |
| hsa-miR-422a | ZNF805 | NM_001145078 |
| hsa-miR-424-3p | HNRNPA0 | NM_006805 |
| hsa-miR-424-3p | SRF | NM_003131 |
| hsa-miR-4286 | ABR | NM_001092 |
| hsa-miR-4286 | ABR | NM_001159746 |
| hsa-miR-4286 | ABR | NM_021962 |
| hsa-miR-4286 | ATG9A | NM_001077198 |
| hsa-miR-4286 | ATG9A | NM_024085 |
| hsa-miR-4286 | ATP2A2 | NM_170665 |
| hsa-miR-4286 | ATXN7L3 | NM_001098833 |
| hsa-miR-4286 | ATXN7L3 | NM_020218 |
| hsa-miR-4286 | C1orf95 | NM_001003665 |
| hsa-miR-4286 | C22orf46 | NM_001142964 |
| hsa-miR-4286 | CBX5 | NM_001127321 |
| hsa-miR-4286 | CBX5 | NM_001127322 |
| hsa-miR-4286 | CBX5 | NM_012117 |
| hsa-miR-4286 | CHTF8 | NM_001039690 |
| hsa-miR-4286 | CHTF8 | NM_001040146 |
| hsa-miR-4286 | DGCR14 | NM_022719 |
| hsa-miR-4286 | DYNLL2 | NM_080677 |
| hsa-miR-4286 | GAB2 | NM_012296 |
| hsa-miR-4286 | GAB2 | NM_080491 |
| hsa-miR-4286 | HNRNPUL2 | NM_001079559 |
| hsa-miR-4286 | HOXB9 | NM_024017 |
| hsa-miR-4286 | IL13RA1 | NM_001560 |
| hsa-miR-4286 | KCNK5 | NM_003740 |
| hsa-miR-4286 | KREMEN1 | NM_001039570 |
| hsa-miR-4286 | MPRIP | NM_015134 |
| hsa-miR-4286 | MPRIP | NM_201274 |
| hsa-miR-4286 | NEURL4 | NM_001005408 |
| hsa-miR-4286 | NEURL4 | NM_032442 |
| hsa-miR-4286 | ORMDL3 | NM_139280 |
| hsa-miR-4286 | PBX2 | NM_002586 |
| hsa-miR-4286 | PDCD6IP | NM_001162429 |
| hsa-miR-4286 | PDCD6IP | NM_013374 |
| hsa-miR-4286 | PRKAG1 | NM_001206709 |
| hsa-miR-4286 | PRKAG1 | NM_001206710 |
| hsa-miR-4286 | PRKAG1 | NM_002733 |
| hsa-miR-4286 | PSMD11 | NM_002815 |
| hsa-miR-4286 | RANBP10 | NM_020850 |
| hsa-miR-4286 | RCAN2 | NM_005822 |
| hsa-miR-4286 | RNF121 | NM_018320 |
| hsa-miR-4286 | RNF144B | NM_182757 |
| hsa-miR-4286 | TOMM40L | NM_032174 |
| hsa-miR-4286 | TRAF7 | NM_032271 |
| hsa-miR-4286 | UBE2R2 | NM_017811 |
| hsa-miR-4286 | VAMP1 | NM_199245 |
| hsa-miR-4286 | ZBTB47 | NM_145166 |
| hsa-miR-4286 | ZHX3 | NM_015035 |
| hsa-miR-4417 | ABLIM3 | NM_014945 |
| hsa-miR-4417 | ARHGEF4 | NM_015320 |
| hsa-miR-4417 | ARHGEF4 | NM_032995 |
| hsa-miR-4417 | C1orf95 | NM_001003665 |
| hsa-miR-4417 | CHST2 | NM_004267 |
| hsa-miR-4417 | DMBX1 | NM_147192 |
| hsa-miR-4417 | DMBX1 | NM_172225 |
| hsa-miR-4417 | DPYSL5 | NM_020134 |
| hsa-miR-4417 | ENOSF1 | NM_001126123 |
| hsa-miR-4417 | ENOSF1 | NM_017512 |
| hsa-miR-4417 | ENOSF1 | NM_202758 |
| hsa-miR-4417 | FAF2 | NM_014613 |
| hsa-miR-4417 | FKRP | NM_001039885 |
| hsa-miR-4417 | FKRP | NM_024301 |
| hsa-miR-4417 | GRIN1 | NM_000832 |
| hsa-miR-4417 | GRIN1 | NM_001185090 |
| hsa-miR-4417 | GRIN1 | NM_001185091 |
| hsa-miR-4417 | KPNA1 | NM_002264 |
| hsa-miR-4417 | MYO1C | NM_001080779 |
| hsa-miR-4417 | MYO1C | NM_001080950 |
| hsa-miR-4417 | MYO1C | NM_033375 |
| hsa-miR-4417 | NAT8L | NM_178557 |
| hsa-miR-4417 | PRM2 | NM_002762 |
| hsa-miR-4417 | PSKH1 | NM_006742 |
| hsa-miR-4417 | QRICH2 | NM_032134 |
| hsa-miR-4417 | STK38 | NM_007271 |
| hsa-miR-4417 | SYNGR1 | NM_145731 |
| hsa-miR-4417 | SYNGR1 | NM_145738 |
| hsa-miR-4417 | TET1 | NM_030625 |
| hsa-miR-4417 | TUFT1 | NM_001126337 |
| hsa-miR-4417 | TUFT1 | NM_020127 |
| hsa-miR-4417 | TXLNG | NM_001168683 |
| hsa-miR-4417 | TXLNG | NM_018360 |
| hsa-miR-4417 | WDR48 | NM_020839 |
| hsa-miR-4417 | ZFHX2 | NM_033400 |
| hsa-miR-4449 | ACAN | NM_001135 |
| hsa-miR-4449 | ACAN | NM_013227 |
| hsa-miR-4449 | CDC42BPG | NM_017525 |
| hsa-miR-4449 | CDK5R2 | NM_003936 |
| hsa-miR-4449 | HIC1 | NM_001098202 |
| hsa-miR-4449 | HIC1 | NM_006497 |
| hsa-miR-4449 | LRFN1 | NM_020862 |
| hsa-miR-4449 | RBM14-RBM4 | NM_001198845 |
| hsa-miR-4449 | RBM14-RBM4 | NM_001198846 |
| hsa-miR-4449 | RBM4 | NM_001198843 |
| hsa-miR-4449 | RBM4 | NM_002896 |
| hsa-miR-486-5p | ARID1A | NM_006015 |
| hsa-miR-486-5p | ARID1A | NM_139135 |
| hsa-miR-486-5p | ASB4 | NM_016116 |
| hsa-miR-486-5p | CCDC85C | NM_001144995 |
| hsa-miR-486-5p | CEP350 | NM_014810 |
| hsa-miR-486-5p | CXCR5 | NM_001716 |
| hsa-miR-486-5p | CXCR5 | NM_032966 |
| hsa-miR-486-5p | DCBLD2 | NM_080927 |
| hsa-miR-486-5p | EPHA3 | NM_005233 |
| hsa-miR-486-5p | FLRT2 | NM_013231 |
| hsa-miR-486-5p | FOXO1 | NM_002015 |
| hsa-miR-486-5p | GAB2 | NM_012296 |
| hsa-miR-486-5p | GAB2 | NM_080491 |
| hsa-miR-486-5p | GABRB3 | NM_000814 |
| hsa-miR-486-5p | GABRB3 | NM_001191320 |
| hsa-miR-486-5p | GABRB3 | NM_001191321 |
| hsa-miR-486-5p | GABRB3 | NM_021912 |
| hsa-miR-486-5p | HAT1 | NM_003642 |
| hsa-miR-486-5p | KCNQ3 | NM_001204824 |
| hsa-miR-486-5p | KCNQ3 | NM_004519 |
| hsa-miR-486-5p | NCOA6 | NM_001242539 |
| hsa-miR-486-5p | NCOA6 | NM_014071 |
| hsa-miR-486-5p | NFE2L1 | NM_003204 |
| hsa-miR-486-5p | OLFM4 | NM_006418 |
| hsa-miR-486-5p | PELI2 | NM_021255 |
| hsa-miR-486-5p | POU2F1 | NM_001198783 |
| hsa-miR-486-5p | POU2F1 | NM_001198786 |
| hsa-miR-486-5p | POU2F1 | NM_002697 |
| hsa-miR-486-5p | RBM12 | NM_001198838 |
| hsa-miR-486-5p | RBM12 | NM_001198840 |
| hsa-miR-486-5p | RBM12 | NM_006047 |
| hsa-miR-486-5p | RBM12 | NM_152838 |
| hsa-miR-486-5p | SHB | NM_003028 |
| hsa-miR-486-5p | SLC4A8 | NM_001039960 |
| hsa-miR-486-5p | SNRPD1 | NM_006938 |
| hsa-miR-486-5p | SORCS1 | NM_001013031 |
| hsa-miR-486-5p | SORCS1 | NM_001206570 |
| hsa-miR-486-5p | SORCS1 | NM_001206572 |
| hsa-miR-486-5p | SORCS1 | NM_052918 |
| hsa-miR-486-5p | SP5 | NM_001003845 |
| hsa-miR-486-5p | ST5 | NM_005418 |
| hsa-miR-486-5p | ST5 | NM_139157 |
| hsa-miR-486-5p | ST5 | NM_213618 |
| hsa-miR-486-5p | STK35 | NM_080836 |
| hsa-miR-486-5p | STK4 | NM_006282 |
| hsa-miR-486-5p | TMOD1 | NM_001166116 |
| hsa-miR-486-5p | TMOD1 | NM_003275 |
| hsa-miR-486-5p | TTC28 | NM_001145418 |
| hsa-miR-486-5p | TWF1 | NM_001242397 |
| hsa-miR-486-5p | TWF1 | NM_002822 |
| hsa-miR-486-5p | UNC5C | NM_003728 |
| hsa-miR-486-5p | ZNF740 | NM_001004304 |
| hsa-miR-501-5p | CDC27 | NM_001114091 |
| hsa-miR-501-5p | CDC27 | NM_001256 |
| hsa-miR-501-5p | CELF2 | NM_001025077 |
| hsa-miR-501-5p | DNAJB12 | NM_001002762 |
| hsa-miR-501-5p | ERRFI1 | NM_018948 |
| hsa-miR-501-5p | FBXO34 | NM_017943 |
| hsa-miR-501-5p | GATAD2B | NM_020699 |
| hsa-miR-501-5p | GRPEL2 | NM_152407 |
| hsa-miR-501-5p | HIATL1 | NM_032558 |
| hsa-miR-501-5p | HSPA4L | NM_014278 |
| hsa-miR-501-5p | IGF2BP1 | NM_001160423 |
| hsa-miR-501-5p | IGF2BP1 | NM_006546 |
| hsa-miR-501-5p | JMY | NM_152405 |
| hsa-miR-501-5p | KIF24 | NM_194313 |
| hsa-miR-501-5p | LPAR1 | NM_001401 |
| hsa-miR-501-5p | LPAR1 | NM_057159 |
| hsa-miR-501-5p | LPIN1 | NM_145693 |
| hsa-miR-501-5p | MTDH | NM_178812 |
| hsa-miR-501-5p | NAP1L1 | NM_139207 |
| hsa-miR-501-5p | NFASC | NM_001005388 |
| hsa-miR-501-5p | NFASC | NM_001160331 |
| hsa-miR-501-5p | NFASC | NM_001160332 |
| hsa-miR-501-5p | NFASC | NM_015090 |
| hsa-miR-501-5p | NFKB2 | NM_001077494 |
| hsa-miR-501-5p | NFKB2 | NM_002502 |
| hsa-miR-501-5p | PANK3 | NM_024594 |
| hsa-miR-501-5p | PDE11A | NM_001077196 |
| hsa-miR-501-5p | PDE11A | NM_001077197 |
| hsa-miR-501-5p | PDE11A | NM_016953 |
| hsa-miR-501-5p | PEX5L | NM_016559 |
| hsa-miR-501-5p | PI4KB | NM_001198773 |
| hsa-miR-501-5p | PI4KB | NM_001198774 |
| hsa-miR-501-5p | PI4KB | NM_001198775 |
| hsa-miR-501-5p | PI4KB | NM_002651 |
| hsa-miR-501-5p | PPP6R3 | NM_001164160 |
| hsa-miR-501-5p | PPP6R3 | NM_001164161 |
| hsa-miR-501-5p | PPP6R3 | NM_001164162 |
| hsa-miR-501-5p | PPP6R3 | NM_001164163 |
| hsa-miR-501-5p | PPP6R3 | NM_018312 |
| hsa-miR-501-5p | RGSL1 | NM_001137669 |
| hsa-miR-501-5p | RNF165 | NM_152470 |
| hsa-miR-501-5p | ROBO2 | NM_002942 |
| hsa-miR-501-5p | SATB1 | NM_001131010 |
| hsa-miR-501-5p | SATB1 | NM_001195470 |
| hsa-miR-501-5p | SATB1 | NM_002971 |
| hsa-miR-501-5p | SLC24A2 | NM_001193288 |
| hsa-miR-501-5p | SLC24A2 | NM_020344 |
| hsa-miR-501-5p | SMC1A | NM_006306 |
| hsa-miR-501-5p | SPRR2F | NM_001014450 |
| hsa-miR-501-5p | TAF5L | NM_014409 |
| hsa-miR-501-5p | TFRC | NM_001128148 |
| hsa-miR-501-5p | TFRC | NM_003234 |
| hsa-miR-501-5p | UBE2Q1 | NM_017582 |
| hsa-miR-501-5p | WIPF2 | NM_133264 |
| hsa-miR-501-5p | ZMAT3 | NM_022470 |
| hsa-miR-501-5p | ZMAT3 | NM_152240 |
| hsa-miR-503-5p | AATK | NM_001080395 |
| hsa-miR-503-5p | AATK | NM_004920 |
| hsa-miR-503-5p | ACTR2 | NM_001005386 |
| hsa-miR-503-5p | ACTR2 | NM_005722 |
| hsa-miR-503-5p | ACVR2B | NM_001106 |
| hsa-miR-503-5p | ANKS1A | NM_015245 |
| hsa-miR-503-5p | AP3D1 | NM_003938 |
| hsa-miR-503-5p | APLN | NM_017413 |
| hsa-miR-503-5p | ATXN2 | NM_002973 |
| hsa-miR-503-5p | CAPN6 | NM_014289 |
| hsa-miR-503-5p | CCND2 | NM_001759 |
| hsa-miR-503-5p | CDCA4 | NM_017955 |
| hsa-miR-503-5p | CNOT6L | NM_144571 |
| hsa-miR-503-5p | DCTN5 | NM_001199743 |
| hsa-miR-503-5p | DCTN5 | NM_032486 |
| hsa-miR-503-5p | EIF4G1 | NM_001194946 |
| hsa-miR-503-5p | EIF4G1 | NM_001194947 |
| hsa-miR-503-5p | EIF4G1 | NM_004953 |
| hsa-miR-503-5p | EIF4G1 | NM_182917 |
| hsa-miR-503-5p | EIF4G1 | NM_198241 |
| hsa-miR-503-5p | EIF4G1 | NM_198242 |
| hsa-miR-503-5p | EIF4G1 | NM_198244 |
| hsa-miR-503-5p | FAM122A | NM_138333 |
| hsa-miR-503-5p | FAM189A1 | NM_015307 |
| hsa-miR-503-5p | FSTL4 | NM_015082 |
| hsa-miR-503-5p | HELZ | NM_014877 |
| hsa-miR-503-5p | IGF1R | NM_000875 |
| hsa-miR-503-5p | IRAK2 | NM_001570 |
| hsa-miR-503-5p | IRF2BP2 | NM_001077397 |
| hsa-miR-503-5p | IRF2BP2 | NM_182972 |
| hsa-miR-503-5p | KDSR | NM_002035 |
| hsa-miR-503-5p | LRRC58 | NM_001099678 |
| hsa-miR-503-5p | MAFB | NM_005461 |
| hsa-miR-503-5p | MAPK8IP2 | NM_012324 |
| hsa-miR-503-5p | MAPK8IP2 | NM_016431 |
| hsa-miR-503-5p | MYBL1 | NM_001080416 |
| hsa-miR-503-5p | MYBL1 | NM_001144755 |
| hsa-miR-503-5p | NYNRIN | NM_025081 |
| hsa-miR-503-5p | PAPPA | NM_002581 |
| hsa-miR-503-5p | PHOX2B | NM_003924 |
| hsa-miR-503-5p | PTPN12 | NM_001131008 |
| hsa-miR-503-5p | PTPN12 | NM_001131009 |
| hsa-miR-503-5p | PTPN12 | NM_002835 |
| hsa-miR-503-5p | SCAI | NM_001144877 |
| hsa-miR-503-5p | SCAI | NM_173690 |
| hsa-miR-503-5p | TFCP2L1 | NM_014553 |
| hsa-miR-503-5p | TLN1 | NM_006289 |
| hsa-miR-503-5p | TMEM55A | NM_018710 |
| hsa-miR-503-5p | TMEM55B | NM_001100814 |
| hsa-miR-503-5p | TMEM55B | NM_144568 |
| hsa-miR-503-5p | TNPO1 | NM_002270 |
| hsa-miR-503-5p | TNPO1 | NM_153188 |
| hsa-miR-503-5p | USP2 | NM_004205 |
| hsa-miR-503-5p | USP2 | NM_171997 |
| hsa-miR-503-5p | WNT4 | NM_030761 |
| hsa-miR-552-3p | ACVR2B | NM_001106 |
| hsa-miR-552-3p | AMOTL1 | NM_130847 |
| hsa-miR-552-3p | API5 | NM_001142930 |
| hsa-miR-552-3p | API5 | NM_001142931 |
| hsa-miR-552-3p | API5 | NM_006595 |
| hsa-miR-552-3p | ATXN2L | NM_007245 |
| hsa-miR-552-3p | BCL11A | NM_022893 |
| hsa-miR-552-3p | BTBD9 | NM_001099272 |
| hsa-miR-552-3p | BTBD9 | NM_001172418 |
| hsa-miR-552-3p | BTBD9 | NM_052893 |
| hsa-miR-552-3p | BTBD9 | NM_152733 |
| hsa-miR-552-3p | C17orf59 | NM_017622 |
| hsa-miR-552-3p | C20orf112 | NM_080616 |
| hsa-miR-552-3p | CAMTA1 | NM_015215 |
| hsa-miR-552-3p | CAST | NM_001042440 |
| hsa-miR-552-3p | CAST | NM_001190442 |
| hsa-miR-552-3p | CAST | NM_173060 |
| hsa-miR-552-3p | CBL | NM_005188 |
| hsa-miR-552-3p | CELF6 | NM_001172684 |
| hsa-miR-552-3p | CELF6 | NM_001172685 |
| hsa-miR-552-3p | CELF6 | NM_052840 |
| hsa-miR-552-3p | EFR3A | NM_015137 |
| hsa-miR-552-3p | EIF2B3 | NM_020365 |
| hsa-miR-552-3p | FSCN3 | NM_020369 |
| hsa-miR-552-3p | GANAB | NM_198334 |
| hsa-miR-552-3p | GANAB | NM_198335 |
| hsa-miR-552-3p | GRSF1 | NM_001098477 |
| hsa-miR-552-3p | GRSF1 | NM_002092 |
| hsa-miR-552-3p | HMP19 | NM_015980 |
| hsa-miR-552-3p | HNRNPUL2 | NM_001079559 |
| hsa-miR-552-3p | INPP5B | NM_005540 |
| hsa-miR-552-3p | IRX5 | NM_005853 |
| hsa-miR-552-3p | KIAA0247 | NM_014734 |
| hsa-miR-552-3p | KLK15 | NM_017509 |
| hsa-miR-552-3p | LRRC8C | NM_032270 |
| hsa-miR-552-3p | LRRC8E | NM_025061 |
| hsa-miR-552-3p | MYO10 | NM_012334 |
| hsa-miR-552-3p | NDUFA6 | NM_002490 |
| hsa-miR-552-3p | NRG1 | NM_001159996 |
| hsa-miR-552-3p | NRG1 | NM_001160004 |
| hsa-miR-552-3p | NRG1 | NM_013960 |
| hsa-miR-552-3p | NUP153 | NM_005124 |
| hsa-miR-552-3p | PDE5A | NM_001083 |
| hsa-miR-552-3p | PDE5A | NM_033430 |
| hsa-miR-552-3p | PDE5A | NM_033437 |
| hsa-miR-552-3p | PHRF1 | NM_020901 |
| hsa-miR-552-3p | PLXNA2 | NM_025179 |
| hsa-miR-552-3p | PPEF2 | NM_006239 |
| hsa-miR-552-3p | PTGFR | NM_000959 |
| hsa-miR-552-3p | PTGFR | NM_001039585 |
| hsa-miR-552-3p | RAB4B | NM_016154 |
| hsa-miR-552-3p | RAP2C | NM_021183 |
| hsa-miR-552-3p | RIC3 | NM_001135109 |
| hsa-miR-552-3p | RIC3 | NM_001206671 |
| hsa-miR-552-3p | RIC3 | NM_001206672 |
| hsa-miR-552-3p | RIC3 | NM_024557 |
| hsa-miR-552-3p | SGK1 | NM_001143676 |
| hsa-miR-552-3p | SGK1 | NM_001143677 |
| hsa-miR-552-3p | SGK1 | NM_001143678 |
| hsa-miR-552-3p | SGK1 | NM_005627 |
| hsa-miR-552-3p | SLC30A8 | NM_001172811 |
| hsa-miR-552-3p | SLC30A8 | NM_001172813 |
| hsa-miR-552-3p | SLC30A8 | NM_001172814 |
| hsa-miR-552-3p | SLC30A8 | NM_001172815 |
| hsa-miR-552-3p | SLC30A8 | NM_173851 |
| hsa-miR-552-3p | SMAD3 | NM_001145102 |
| hsa-miR-552-3p | SMAD3 | NM_001145103 |
| hsa-miR-552-3p | SMAD3 | NM_001145104 |
| hsa-miR-552-3p | SMAD3 | NM_005902 |
| hsa-miR-552-3p | SMG5 | NM_015327 |
| hsa-miR-552-3p | SPRY3 | NM_005840 |
| hsa-miR-552-3p | TBX19 | NM_005149 |
| hsa-miR-552-3p | TEK | NM_000459 |
| hsa-miR-552-3p | TMCC3 | NM_020698 |
| hsa-miR-552-3p | TMEM150A | NM_001031738 |
| hsa-miR-552-3p | TOE1 | NM_025077 |
| hsa-miR-552-3p | TOP1 | NM_003286 |
| hsa-miR-552-3p | TTC39A | NM_001080494 |
| hsa-miR-552-3p | TTC39A | NM_001144832 |
| hsa-miR-552-3p | ZMIZ1 | NM_020338 |
| hsa-miR-552-3p | ZNF345 | NM_001242472 |
| hsa-miR-552-3p | ZNF345 | NM_001242474 |
| hsa-miR-552-3p | ZNF345 | NM_001242475 |
| hsa-miR-552-3p | ZNF345 | NM_001242476 |
| hsa-miR-552-3p | ZNF345 | NM_003419 |
| hsa-miR-552-3p | ZNF704 | NM_001033723 |
| hsa-miR-708-5p | ALG9 | NM_001077690 |
| hsa-miR-708-5p | ALG9 | NM_001077691 |
| hsa-miR-708-5p | ALG9 | NM_001077692 |
| hsa-miR-708-5p | ALG9 | NM_024740 |
| hsa-miR-708-5p | ARAF | NM_001654 |
| hsa-miR-708-5p | CASP2 | NM_001224 |
| hsa-miR-708-5p | CASP2 | NM_032982 |
| hsa-miR-708-5p | CASP2 | NM_032983 |
| hsa-miR-708-5p | CCDC113 | NM_001142302 |
| hsa-miR-708-5p | CCDC113 | NM_014157 |
| hsa-miR-708-5p | CCND3 | NM_001136017 |
| hsa-miR-708-5p | CCND3 | NM_001136125 |
| hsa-miR-708-5p | CCND3 | NM_001136126 |
| hsa-miR-708-5p | CCND3 | NM_001760 |
| hsa-miR-708-5p | CDC42SE1 | NM_001038707 |
| hsa-miR-708-5p | CDC42SE1 | NM_020239 |
| hsa-miR-708-5p | CHL1 | NM_006614 |
| hsa-miR-708-5p | CNTFR | NM_001207011 |
| hsa-miR-708-5p | CNTFR | NM_001842 |
| hsa-miR-708-5p | CNTFR | NM_147164 |
| hsa-miR-708-5p | CREB1 | NM_004379 |
| hsa-miR-708-5p | CREB1 | NM_134442 |
| hsa-miR-708-5p | DCAF10 | NM_024345 |
| hsa-miR-708-5p | DCUN1D5 | NM_032299 |
| hsa-miR-708-5p | DKK3 | NM_001018057 |
| hsa-miR-708-5p | DKK3 | NM_013253 |
| hsa-miR-708-5p | DKK3 | NM_015881 |
| hsa-miR-708-5p | DRAM1 | NM_018370 |
| hsa-miR-708-5p | DYSF | NM_001130455 |
| hsa-miR-708-5p | DYSF | NM_001130976 |
| hsa-miR-708-5p | DYSF | NM_001130977 |
| hsa-miR-708-5p | DYSF | NM_001130978 |
| hsa-miR-708-5p | DYSF | NM_001130979 |
| hsa-miR-708-5p | DYSF | NM_001130980 |
| hsa-miR-708-5p | DYSF | NM_001130981 |
| hsa-miR-708-5p | DYSF | NM_001130982 |
| hsa-miR-708-5p | DYSF | NM_001130983 |
| hsa-miR-708-5p | DYSF | NM_001130984 |
| hsa-miR-708-5p | DYSF | NM_001130985 |
| hsa-miR-708-5p | DYSF | NM_001130986 |
| hsa-miR-708-5p | DYSF | NM_001130987 |
| hsa-miR-708-5p | DYSF | NM_003494 |
| hsa-miR-708-5p | E2F6 | NM_198256 |
| hsa-miR-708-5p | EFR3B | NM_014971 |
| hsa-miR-708-5p | EPDR1 | NM_017549 |
| hsa-miR-708-5p | ETF1 | NM_004730 |
| hsa-miR-708-5p | FAM135B | NM_015912 |
| hsa-miR-708-5p | FBXO40 | NM_016298 |
| hsa-miR-708-5p | FOXJ3 | NM_001198850 |
| hsa-miR-708-5p | FOXJ3 | NM_001198851 |
| hsa-miR-708-5p | FOXJ3 | NM_001198852 |
| hsa-miR-708-5p | FOXJ3 | NM_014947 |
| hsa-miR-708-5p | GPM6A | NM_005277 |
| hsa-miR-708-5p | GPM6A | NM_201591 |
| hsa-miR-708-5p | GPM6A | NM_201592 |
| hsa-miR-708-5p | GRIA4 | NM_000829 |
| hsa-miR-708-5p | GRIA4 | NM_001077243 |
| hsa-miR-708-5p | HEPH | NM_001130860 |
| hsa-miR-708-5p | HEPH | NM_014799 |
| hsa-miR-708-5p | HEPH | NM_138737 |
| hsa-miR-708-5p | HTRA2 | NM_013247 |
| hsa-miR-708-5p | HTRA2 | NM_145074 |
| hsa-miR-708-5p | IMPDH1 | NM_000883 |
| hsa-miR-708-5p | IMPDH1 | NM_001102605 |
| hsa-miR-708-5p | IMPDH1 | NM_001142573 |
| hsa-miR-708-5p | IMPDH1 | NM_001142574 |
| hsa-miR-708-5p | IMPDH1 | NM_001142575 |
| hsa-miR-708-5p | IMPDH1 | NM_001142576 |
| hsa-miR-708-5p | IMPDH1 | NM_183243 |
| hsa-miR-708-5p | KPNA4 | NM_002268 |
| hsa-miR-708-5p | LPPR5 | NM_001010861 |
| hsa-miR-708-5p | LPPR5 | NM_001037317 |
| hsa-miR-708-5p | LYPD3 | NM_014400 |
| hsa-miR-708-5p | MAP3K13 | NM_001242314 |
| hsa-miR-708-5p | MAP3K13 | NM_001242317 |
| hsa-miR-708-5p | MAP3K13 | NM_004721 |
| hsa-miR-708-5p | MPL | NM_005373 |
| hsa-miR-708-5p | N4BP1 | NM_153029 |
| hsa-miR-708-5p | NANOS1 | NM_199461 |
| hsa-miR-708-5p | NNAT | NM_005386 |
| hsa-miR-708-5p | NNAT | NM_181689 |
| hsa-miR-708-5p | OSBPL10 | NM_001174060 |
| hsa-miR-708-5p | OSBPL10 | NM_017784 |
| hsa-miR-708-5p | OTUB1 | NM_017670 |
| hsa-miR-708-5p | PAFAH1B2 | NM_002572 |
| hsa-miR-708-5p | PFKM | NM_000289 |
| hsa-miR-708-5p | PFKM | NM_001166686 |
| hsa-miR-708-5p | PFKM | NM_001166687 |
| hsa-miR-708-5p | PFKM | NM_001166688 |
| hsa-miR-708-5p | PPIE | NM_006112 |
| hsa-miR-708-5p | PRUNE2 | NM_015225 |
| hsa-miR-708-5p | PSAP | NM_002778 |
| hsa-miR-708-5p | RAB14 | NM_016322 |
| hsa-miR-708-5p | RAB8B | NM_016530 |
| hsa-miR-708-5p | RCVRN | NM_002903 |
| hsa-miR-708-5p | RNF165 | NM_152470 |
| hsa-miR-708-5p | RORA | NM_002943 |
| hsa-miR-708-5p | RORA | NM_134260 |
| hsa-miR-708-5p | RORA | NM_134261 |
| hsa-miR-708-5p | RORA | NM_134262 |
| hsa-miR-708-5p | SCAF4 | NM_001145444 |
| hsa-miR-708-5p | SCAF4 | NM_001145445 |
| hsa-miR-708-5p | SCAF4 | NM_020706 |
| hsa-miR-708-5p | SEMA4C | NM_017789 |
| hsa-miR-708-5p | SEMA7A | NM_001146029 |
| hsa-miR-708-5p | SEMA7A | NM_001146030 |
| hsa-miR-708-5p | SEMA7A | NM_003612 |
| hsa-miR-708-5p | SLC13A3 | NM_001011554 |
| hsa-miR-708-5p | SLC13A3 | NM_001193339 |
| hsa-miR-708-5p | SLC13A3 | NM_001193340 |
| hsa-miR-708-5p | SLC13A3 | NM_001193342 |
| hsa-miR-708-5p | SLC13A3 | NM_022829 |
| hsa-miR-708-5p | SLC31A1 | NM_001859 |
| hsa-miR-708-5p | SLC37A4 | NM_001164277 |
| hsa-miR-708-5p | SLC37A4 | NM_001164278 |
| hsa-miR-708-5p | SLC37A4 | NM_001164279 |
| hsa-miR-708-5p | SLC37A4 | NM_001467 |
| hsa-miR-708-5p | SLC44A5 | NM_001130058 |
| hsa-miR-708-5p | SLCO1C1 | NM_001145945 |
| hsa-miR-708-5p | SLCO1C1 | NM_017435 |
| hsa-miR-708-5p | SRPRB | NM_021203 |
| hsa-miR-708-5p | SSRP1 | NM_003146 |
| hsa-miR-708-5p | ST7 | NM_021908 |
| hsa-miR-708-5p | STK24 | NM_001032296 |
| hsa-miR-708-5p | STK24 | NM_003576 |
| hsa-miR-708-5p | STK4 | NM_006282 |
| hsa-miR-708-5p | TAGAP | NM_054114 |
| hsa-miR-708-5p | TAGAP | NM_152133 |
| hsa-miR-708-5p | TEX261 | NM_144582 |
| hsa-miR-708-5p | TLN2 | NM_015059 |
| hsa-miR-708-5p | TMEM167B | NM_020141 |
| hsa-miR-708-5p | TRNP1 | NM_001013642 |
| hsa-miR-708-5p | TTC39A | NM_001080494 |
| hsa-miR-708-5p | TTC39A | NM_001144832 |
| hsa-miR-708-5p | WIPF2 | NM_133264 |
| hsa-miR-708-5p | YWHAZ | NM_001135699 |
| hsa-miR-708-5p | YWHAZ | NM_001135700 |
| hsa-miR-708-5p | YWHAZ | NM_001135701 |
| hsa-miR-708-5p | YWHAZ | NM_001135702 |
| hsa-miR-708-5p | YWHAZ | NM_003406 |
| hsa-miR-708-5p | YWHAZ | NM_145690 |
| hsa-miR-708-5p | ZBED4 | NM_014838 |
| hsa-miR-877-5p | ADAMTSL1 | NM_001040272 |
| hsa-miR-877-5p | AR | NM_000044 |
| hsa-miR-877-5p | AR | NM_001011645 |
| hsa-miR-877-5p | ATXN1L | NM_001137675 |
| hsa-miR-877-5p | CAP1 | NM_001105530 |
| hsa-miR-877-5p | CAP1 | NM_006367 |
| hsa-miR-877-5p | CCDC34 | NM_080654 |
| hsa-miR-877-5p | CDC40 | NM_015891 |
| hsa-miR-877-5p | COL6A3 | NM_004369 |
| hsa-miR-877-5p | COL6A3 | NM_057166 |
| hsa-miR-877-5p | COL6A3 | NM_057167 |
| hsa-miR-877-5p | EID1 | NM_014335 |
| hsa-miR-877-5p | FXR2 | NM_004860 |
| hsa-miR-877-5p | HAND2 | NM_021973 |
| hsa-miR-877-5p | IGF2BP2 | NM_001007225 |
| hsa-miR-877-5p | IGF2BP2 | NM_006548 |
| hsa-miR-877-5p | ING3 | NM_019071 |
| hsa-miR-877-5p | MACROD2 | NM_001033087 |
| hsa-miR-877-5p | MACROD2 | NM_080676 |
| hsa-miR-877-5p | NCOR1 | NM_001190440 |
| hsa-miR-877-5p | NCOR1 | NM_006311 |
| hsa-miR-877-5p | PAIP1 | NM_006451 |
| hsa-miR-877-5p | PAIP1 | NM_182789 |
| hsa-miR-877-5p | PAIP1 | NM_183323 |
| hsa-miR-877-5p | PCDH19 | NM_001105243 |
| hsa-miR-877-5p | PCDH19 | NM_001184880 |
| hsa-miR-877-5p | PCDH19 | NM_020766 |
| hsa-miR-877-5p | PHF8 | NM_001184898 |
| hsa-miR-877-5p | SCN3A | NM_001081676 |
| hsa-miR-877-5p | SCN3A | NM_001081677 |
| hsa-miR-877-5p | SCN3A | NM_006922 |
| hsa-miR-877-5p | SLC16A14 | NM_152527 |
| hsa-miR-877-5p | SMG5 | NM_015327 |
| hsa-miR-877-5p | SORBS3 | NM_001018003 |
| hsa-miR-877-5p | SORBS3 | NM_005775 |
| hsa-miR-877-5p | TP53INP2 | NM_021202 |
| hsa-miR-877-5p | TRIM10 | NM_006778 |
| hsa-miR-877-5p | WHSC1 | NM_133334 |
| hsa-miR-877-5p | YLPM1 | NM_019589 |
| hsa-miR-877-5p | YTHDF1 | NM_017798 |
| hsa-miR-877-5p | ZNF174 | NM_003450 |

**Table S2：Putative target genes of DE-miRNAs.**
