# Supplementary material for: Screening and Functional Analysis of Hub MicroRNAs Related to Tumor Development in Colon Cancer
Source: Biomed Res Int. 2020 Jan 23;2020:3981931. doi: 10.1155/2020/3981931 (PMC6998761; doi:10.1155/2020/3981931)
Supplement: Supplementary 3 — Table S3: clinicopathological correlation analysis for GSE35834. [file 3981931.f3.docx]

**Table S3: Clinicopathological correlation analysis for GSE35834.**

| Characteristics | miR-17-5p expression | | | χ2 | p-Value | miR-182-5p expression | | | χ2 | p-Value | miR-200a-3p expression | | | χ2 | p-Value |
| --- | --- | --- | --- | --- | --- | --- | --- | --- | --- | --- | --- | --- | --- | --- | --- |
|  | High level (n = 26) | | Low level (n = 26) |  |  | High level (n = 26) | Low level (n = 26) | |  |  | High level (n = 26) | | Low level (n = 26) |  |  |
| Age |  | | | | | | | | | | | | | | |
| ≤60 | 13 | 7 | | 2.925 | 0.087 | 9 | | 11 | 0.325 | 0.569 | 7 | 13 | | 2.925 | 0.087 |
| >60 | 13 | 19 | |  |  | 17 | | 15 |  |  | 19 | 13 | |  |  |
| Gender |  | | | | | | | | | | | | | | |
| Female | 8 | 8 | | 0.000 | 1.000 | 10 | | 6 | 1.444 | 0.229 | 8 | 8 | | 0.000 | 1.000 |
| Male | 18 | 18 | |  |  | 16 | | 20 |  |  | 18 | 18 | |  |  |
| Grade |  | | | | | | | | | | | | | | |
| Low | 13 | 21 | | 5.438 | 0.020 | 20 | | 14 | 3.059 | 0.080 | 17 | 17 | | 0.000 | 1.000 |
| High | 13 | 5 | |  |  | 6 | | 12 |  |  | 9 | 9 | |  |  |
| TNM stage |  | | | | | | | | | | | | | | |
| Ⅰ+Ⅱ | 4 | 1 | | 1.991 | 0.158 | 3 | | 2 | 0.221 | 0.638 | 5 | 0 | | 5.532 | 0.019 |
| Ⅲ+Ⅳ | 22 | 25 | |  |  | 23 | | 24 |  |  | 21 | 26 | |  |  |
| Metastasis |  | | | | | | | | | | | | | | |
| Yes | 13 | 10 | | 0.702 | 0.402 | 12 | | 11 | 0.078 | 0.780 | 9 | 14 | | 1.949 | 0.163 |
| No | 13 | 16 | |  |  | 14 | | 15 |  |  | 17 | 12 | |  |  |
